# Recall Network: A Simple Brain-Inspired Algorithm for Classification

**DOI:** 10.1155/2022/9374946

**Published:** 2022-08-13

**Authors:** Zhaoning Tian, Ying Li, Zhenhua Li, Site Li

**Affiliations:** ^1^School of Computer, China University of Geosciences (Wuhan), Wuhan 430074, China; ^2^Apple Incorporated Company, Cupertino, CA 95014, USA

## Abstract

The latest development of neuroscience has deepened the understanding of the information-processing mechanisms in the human brain and inspired a couple of sophisticated computational methods, such as deep learning, memory networks, and hierarchical temporal memory. However, it remains a challenge to explore simpler models due to the high computational cost of the above-mentioned methods. This paper proposes recall network (RN), an intuitive and simple model, that initializes itself by constructing the network path derived from the correlation of features in the training dataset and then makes classification decisions by recalling the paths that are relevant to the features in the test set. The algorithm has been applied to 263 datasets available from UCI Machine Learning Repository, and the classification results of repeated 10-fold cross-validation experiments on Weka demonstrate its competitive performance with prestigious classification algorithms, such as ANN, J48, and KNN.

## 1. Introduction

Brain-inspired algorithms, like artificial neural networks, have shown great success in solving numerous problems in multiple fields for many years. Recently, the development of brain science and neuroscience has deepened the understanding of brain information-processing mechanisms and therefore inspires new generation computational models. For example, deep learning surpasses artificial neural networks (ANNs) in terms of both complexity and capability [1–3], and later in 2015, memory networks (MemNN) came with a long-term memory component [4, 5]; on the other hand, hierarchical temporal memory (HTM) builds a tree-shaped hierarchy of levels where the higher level gets input from results from the next lower level [6, 7]. Bin Hu etc. suggested that it is a feasible way to reconstruct cortical networks with dynamic activities instead of using only artificial computing networks [8].

Those algorithms have a trend of being more and more sophisticated. However, designing simpler models is still worth exploring due to the high computational cost of the above-mentioned methods. This work proposes a straightforward model called recall network (RN), which is a memory network that stores, marks, and then retrieves the previous paths. A RN consists of nodes and edges (path), where a node represents an attribute (feature) value, while an edge connects between two nodes and denotes the number of samples that has both attribute values. For each sample, if we connect its attribute values consecutively, we can form an end-to-end route. During the prediction phase, the network determines its type by plural edges along the route. For example, if a route consists of three edges with 2 YES and 1 NO, then the result will be YES.

This paper investigates the use and application of recall network in the realm of classification field. The objective of the study is to unveil the capabilities of this newly developed method on benchmark problems of classification. Compared with other classic approaches, the performance of the proposed algorithm is experimented and evaluated on Weka [9], a prestigious machine learning platform with the function of the fair comparison of algorithm performance. The results obtained verify the promising performance of the proposed algorithm.

The remaining sections of the paper are organized as follows. The second section introduces the proposed algorithm with particular emphasis on its application in the classification field. In the third section, the concept of RN is compared with other similar algorithms. Classification examples proving the accuracy of the propose algorithm are covered in the fourth section. The fifth section discusses the difference between RN and some powerful classification methods. A brief conclusion of the study is given in the last section.

## 2. Structure and Algorithms

### 2.1. Structure of a Recall Network

**Figure 1:**
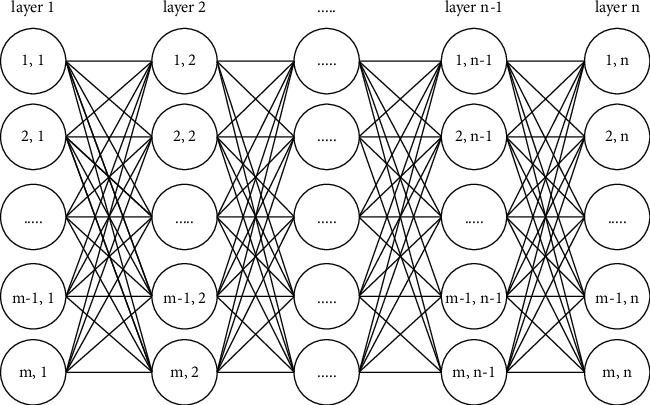
The diagram of a recall network.

A recall network is a group of connected layers, where every layer consists of similar nodes that represent the value for a certain attribute (see Figure 1). Also, each connection or edge, expressed as a set, stores relations between connected nodes of previous samples, for the purpose of determining the output value of the edge of the pair nodes by their plurality.

RN can be defined as an undirected graph *G* =  (*N*, *E*), consisting of the set *N* of nodes where nodes representing the same attribute are lined in one layer, and the set *E *of edges, where each edge connects two nodes from adjacent layers.

### 2.2. Mechanism of Recall Network

Each training sample, called an instance in Weka, is represented by a route from a node in the first layer to the last one. Routes are piled up in the training process, and during the prediction phase, each predicted sample, also a route, is voted by each edge whose value was accumulated in the training stage.

The above mechanism can be expressed as

(1) $$\begin{array}{c} f=\text{Mode}\left(e_{1},e_{2},e_{3},\ldots ,e_{n}\right),\\ e_{i}=\text{Mode}\left(e_{i_{1}},e_{i_{2}},e_{i_{3}},\ldots ,e_{i_{j}}\right), \end{array}$$

where *e*_1_, *e*_2_, *e*_3_,…, *e*_*n*_ are edges of the instance route from the 1st layer to the last one (*n*) and the Mode function is to get the mode of the edge set. In the same way, each edge *ei* gets the mode of plural connections of their adjacent nodes *e*_*i*1_, *e*_*i*2_, *e*_*i*3_,…, *e*_*ij*_.

The algorithm is simple to understand and cheap to implement, requiring only three easy steps: building a RN from instances (see Algorithm 1), training it by known data (see Algorithm 2), and using it to classify unknown samples (see Algorithm 3).

### 2.3. A Classification Example

To illustrate the proposed algorithm, an example is shown in Table 1. In this example, our alrogithm aims to predict whether a given weather condition is suitable for playing tennis.

**Figure 2:**
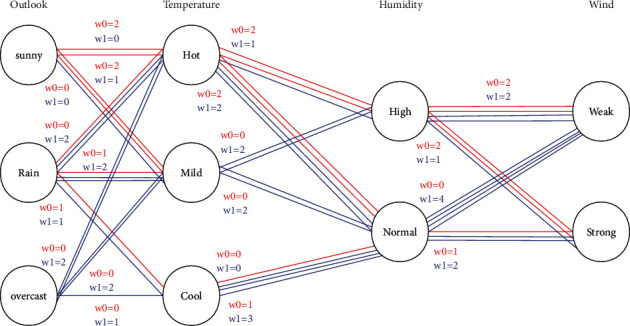
The RN graph corresponding to Table 1.

Figure 2 shows how a RN is created based on Table 1. Here, the RN consists of 4 layers (attributes) with Outlook, Temperature, Humidity, and Wind. Each node represents a possible value for a weather attribute, and nodes that shared the same attribute are arranged in a column. Each edge connects two nodes from adjacent columns. If a given day (a row in Table 1) is suitable for playing tennis (PlayTennis = YES), then we add one blue edge between each adjacent pair of nodes, in which the node should correctly represent the value of a weather attribute on that day. If a given day is not suitable for playing tennis (PlayTennis = NO), we add red edges with similar criteria.

To predict a new sample with following weather condition: Rain, Mild, High, Strong, first, we idenfity the set of 4 nodes that represent that weather condition in Figure 2. Then, we find all the edges (Rain-Mild, Mild-High, High-Strong) that connects each pair of nodes within that set. Finally, we group those edges by color and count them in Figure 2. In this example, we got 3 red edges and 5 blue edges and therefore, this sample is likely to be classified as PlayTennis = YES (5 blues win 3 reds). However, if we got the same number of red and blue edges, the system will choose the color of the first edge in the edge set.

### 2.4. Appropriated Problems

Apparently, the proposed approach can naturally be used for discrete values and categorical values, and datasets with missing or error values are tolerated.

For continuous values, it divides the data range into a couple of intervals, and each interval is regarded as a discrete value. As for the data missing case, the missing node will be skipped.

## 3. Conceptual Comparison of RN with Similar Algorithms

Though RN is a novel algorithm, its structure is still similar to artificial neural network (ANN) and the memory method like pheromone in ant colony optimization (ACO); after all, it is a simple, intuitive, and high readability brain-inspired algorithm.

### 3.1. Compared with the ANN

The RN shares the same biological motivation and similar network structure with ANN; however, there are three main differences.

1. The meaning of nodes is totally different. A node in ANN represents the summation of inputs while it works only as an identifier in RN.
2. Structure is more flexible in ANN but not in RN, since the number of RN layers is decided strictly by the number of attributes, and the number of nodes should equal the number of intervals of the attribute.
3. Though ANN is an effective algorithm in many fields, it is a “black box,” while RN is more explainable.

### 3.2. Compared with the MemNN

MemNN, strictly speaking, is more like a system consisting of a set of memory and inference modules than an independent algorithm. The idea that retrieves the most relevant memory is similar to RN; however, RN is much simpler: it does not need to compress and transform the input or use complex functions to score memories. On the contrary, RN just stores the input as it is and then returns the vote based on the majority marks of the same kind.

### 3.3. Compared with the HTM

Like HTM, RN simulates the mechanism of the cerebral cortex and memorizes patterns for solving problems, but RN has only one “level” and therefore spares the transformation process from lower levels to higher levels; moreover, RN uses a very primitive method—voting—to identify the patterns.

### 3.4. Compared with the ACO

RN is similar to ACO in the idea of “pheromone.” As long as a sample passes through a network route, each segment (edge) in the route will get a permanent mark, and such a mark will never decay along with the time, unlike ACO. We argue that RN more meets biological characteristics of the brain: memories learned by brain will hardly disappear completely, and they will be recalled when a similar event triggers even through a long period.

### 3.5. Comparison of the Mechanism, Computing Complexity, and Structure

To distinguish the RN further, we analyze RN in terms of mechanism, time complexity, space complexity, and structure (Table 2).

In a summary, RN is a distinctive brain-inspired algorithm. Although its network structure is similar to ANN, and its memory mechanisms is similar to MemNN, HTM, and ACO, RN is the simplest of them all.

## 4. Experiments and Results

### 4.1. Testing Tool

To evaluate the performance of the proposed algorithm, we chose Weka, a prestigious machine learning platform that gives a fair comparison among different algorithms. No doubt, Weka is one of the most popular tools to research machine learning algorithms. A number of scientists use this software to study classification problems, for example, Arora and Suman tested J48 and MLP on 5 UCI datasets [18]; Kiranmai and Laxmi classified power quality problems using Weka and studied the effect of attributes on classification accuracy [19]; Mhetre and Nagar adopted Weka to compare four classification algorithms (Naive Bayes, J48, ZeroR, and random tree) on education datasets [20]; Farhat et al. compared the performance of SVM, KNN, NB, logistic regression, decision tree, and random forest on intrusion detection data [21]; Villavicencio et al. studied J48 decision tree, random forest, SVM, KNN, and NB on the COVID-19 Symptoms and Presence dataset from Kaggle [22].

It can be seen from the above research that SVM, KNN, J48, NB, and OneR are competitive methods in the classification field. However, the above research works focus on limited field or limited datasets. In this paper, we used all available data to study the performance comparison.

### 4.2. Testing Data

The test problem includes 263 UCI datasets from UCI Machine Learning Repository. Although the repository offers 466 datasets in total, 121 are too big for Weka, 20 are missing, 53 are for natural language processing, and 9 are pictures. Somehow, the rest still represent a wide range of domains and data characteristics, whose detailed description is listed in Table 3. In a word, all available data in UCI Repository are used for testing.

### 4.3. Method

The RN is published on https://github.com/tianzhaoning/RRN/releases/download/RecallNetwork/RN.zip, and we then retrieved them back to Weka on a local machine to compare with others. In all experiments, 10 runs of 10-fold cross validation are used to average the results.

To illustrate the performance of the proposed method, we chose some competitive classification algorithms, such as Naive Bayes (NB), support vector machine (SVM), decision tree (J48), K-nearest neighbors (KNN), artificial neural network (ANN), and OneR (since it is also a very simple decision tree algorithm), on a bulk of benchmark data.

All algorithms use default parameters supplied by Weka, for the purpose of fair play. However, we applied two more parameters on SVM and one more on ANN besides the default setting to explore the effect under different configurations.

As for SVM, we took the default as the SVM1, changing the following parameters: degree, gamma, cachesize, cost, E-eps, and the *P* value (the value of the loss function in e-SVR). As for ANN, we took the default as ANN1, we change the learning rate and the momentum. The specific values of super-parameters are shown in Table 4.

In the process of statistical analysis, this paper adopts the Friedman test, which is a non-parametric test method using rank to realize whether there are significant differences in multiple population distributions, and ANOVA to test the remarkable mean difference among multiple groups. If both results are yes, we then use the Tukey HSD test to make pair-wise comparisons.

### 4.4. Performance Metrics

Totally, we evaluate the classification performance with the following indicators [23, 24]:

1. Accuracy (ACC): number of correctly predicted items divided by the total of the item to predict. It is the most important metric to measure classification performance. In this work, we use mean ACC: the average of each accuracy per dataset, which represents the average performance over all tasks.
2. Kappa statistic (*k*): a measure of how much better the classification results are compared to classification labels assigned by random chance. Generally, in terms of degree of agreement, *k* ≤ 0 is interpreted as indicating no agreement, 0.01–0.20 as none to slight, 0.21–0.40 as fair, 0.41–0.60 as moderate, 0.61–0.80 as substantial, and 0.81–1.00 as almost perfect agreement.
3. Root mean squared error (RMSE): the standard deviation of the residuals (prediction errors). This is another indicator of model accuracy.
4. Mean absolute error (MAE): the average difference between the prediction result and the true value. MAE can avoid the problem of error cancellation, so it can reflect the actual prediction error.
5. Relative absolute error (RAE): sum of the absolute value of the forecast errors divided by the sum of the absolute values of the mean errors. The RAE considers each error equally important. The better the model is, the closer its RAE is to 0.
6. Root relative squared error (RRSE): square root of the relative squared error (RSE) where RSE means the sum of squared errors of a predictive model normalized by the sum of squared mean errors. It tells how well a model performs relative to the average of the true values.
7. Running time: the executed time of an algorithm on data. It is an intuitive measurement of the time complexity in the real environment. In this paper, we use total time instead of running time per dataset, since Weka does not provide the detailed time on each experiment.

Generally, 10 runs of 10-fold cross validation must be used to average the above results [25] with the exception of run time, which uses the total time.

### 4.5. Results

**Figure 3:**
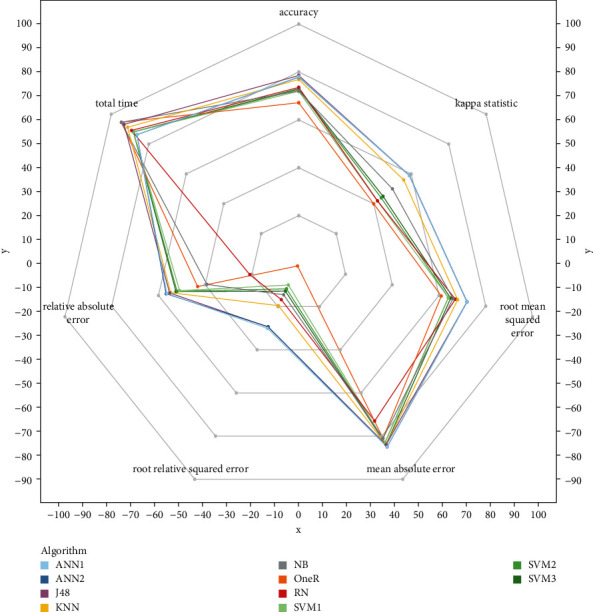
6 performance metrics of 10 models on 263 datasets.

Table 5 and Figure 3 show the average performance of each algorithm on all datasets. The detailed results are listed in Tables 6–11 which present the accuracy (ACC), kappa statistic (*k*), root mean squared error (RMSE), mean absolute error (MAE), relative absolute error (RAE), root relative squared error (RRSE), and their standard deviation of each model on each dataset, respectively.

Then, we used Friedman and ANOVA tests to compare the above 6 performance metrics of all models among 263 datasets, and the tests proved that all algorithms differed in both the distribution and the mean value at 95% confidence level (see Table 5). Then, the Tukey HSD test is employed to determine which pair has significant differences, and the detailed result is presented in Tables 12–17, where the result underlined means the corresponding pair is remarkably different.

**Figure 4:**
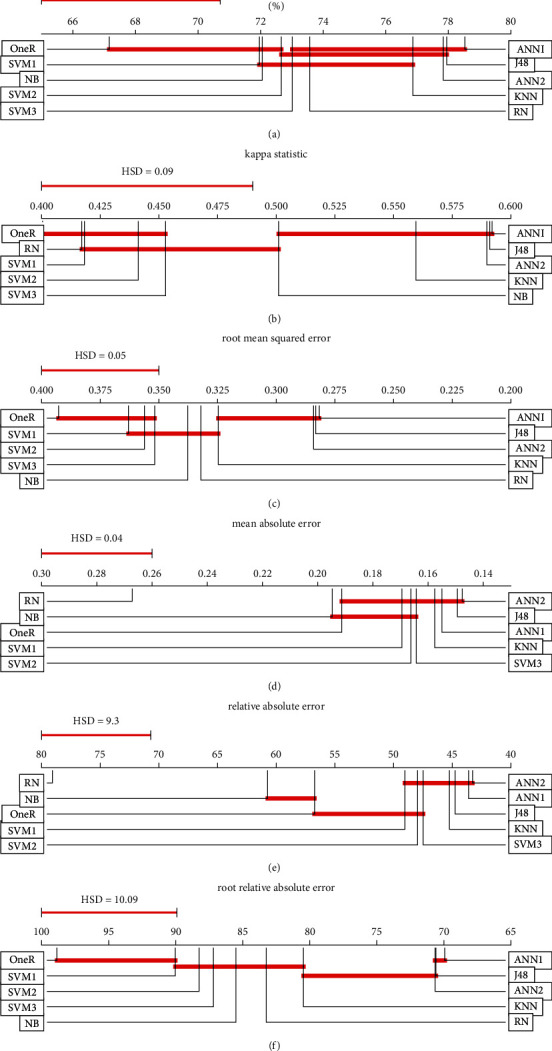
Tukey test results of 6 performance metrics.

Some findings of RN are summarized briefly as follows:

1. Figure 3 shows that the performance of RN is not significantly different from other prestigious classification algorithms.
2. As for average accuracy, the most important performance indicator, Table 5 shows that RN (73.56%) significantly outperforms OneR (67.16%) and is not statistically different from other algorithms (Figure 4(a)).
3. For kappa statistic, RN is different from ANN1, ANN2, J48, and KNN (Figure 4(b)); however, all algorithms range from 0.41 to 0.60 (except RN); therefore, they are in the same level: moderate.
4. About root mean squared error (RMSE) and root relative squared error (RRSE), RN outperforms OneR and lags behind ANN1, ANN2, and J48 (Figures 4(c) and 4(f)).
5. On mean absolute error (MAE) and relative absolute error (RAE), RN is weaker than others (Figures 4(d) and 4(e)).
6. With regard to running time, RN works faster than ANN1, ANN2, SVM1, SVM2, and SVM3, while it works slower than the remaining algorithms (Table 5).

In a word, though the proposed algorithm is simple and straightforward, it still shows the competitive performance with the above prestigious classification algorithms on core indicators.

## 5. Discussion

### 5.1. RN vs Ensemble Algorithms

As we all know, there are many powerful ensemble algorithms in the field of classification, such as random forest, random tree, and so on. However, these ensemble methods use multiple learning algorithms to achieve better predictive performance than any of the constituent learning algorithms alone. Therefore, it is not fair to compare ensemble methods with recall network, a simple and basic algorithm with a single classifier. it is not fair to compare ensemble methods with recall network, a simple and basic algorithm with a single classifier.

### 5.2. RN vs LSTM

In this paper, ANN shows the best performance on the classification task, but long short-term memory (LSTM) is a next-generation recurrent neural network and is better than traditional neural networks, let alone with the RN algorithm. Though the operation mechanism of both LSTM and RN is based on memory, the former obviously employs much more nodes and layers and has more complex structure and therefore takes a much longer running time, so the comparison with LSTM is not to be considered in this work.

### 5.3. Statistic Tests

In this paper, we employ three types of statistical methods. Friedman test is used to determine distribution differences of the result of the compared algorithms in the six evaluation indicators, while ANOVA is used to determine the mean difference of that. Then, we use the Tukey HSD test to find which pair has significant mean differences.

## 6. Conclusions

In this paper, a simple and effective classification algorithm is designed as a memory network that stores, marks, and then retrieves the previous paths. Though its structure is similar to artificial neural networks, and the memory mechanism is similar to memory networks, hierarchical temporal memory, and ant colony optimization, the proposed algorithm is still a naive and distinctive algorithm, and more interpretative than others.

To investigate the capabilities of this newly developed method in the realm of dataset classification, the RN is compared with other classic approaches on benchmark problems in Weka, and experiments show that this simple algorithm has no statistical difference with ANN, J48, KNN, and SVM in accuracy, and all compared algorithms are in the same level on kappa statistic except OneR, though RN performs poorly on RMSE, MAE, RAE, and RRSE.

There are several possible extensions to this work. On the one hand, the classification performance of RN can be improved further, since it is sensitive to the order of layers, so it could be archiving higher performance by exhaustively exploring all possible RN structures and then choosing the best one to fulfill the prediction; moreover, the technique of ensemble learning is also worthy to be used on bagging and boosting recall networks. On the other hand, RN can be also applied in other fields, for example, clustering based on RN could use Hamming distance to partition the routes into small groups.

## Figures and Tables

**Algorithm 1:**
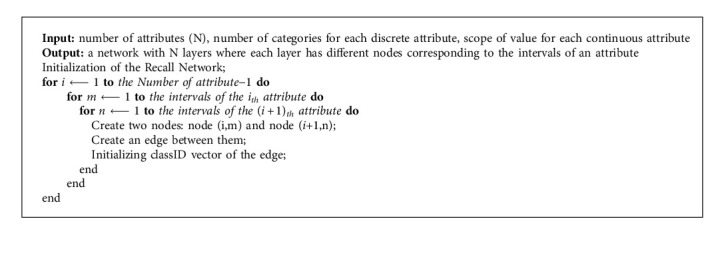
Building a recall network from instances.

**Algorithm 2:**
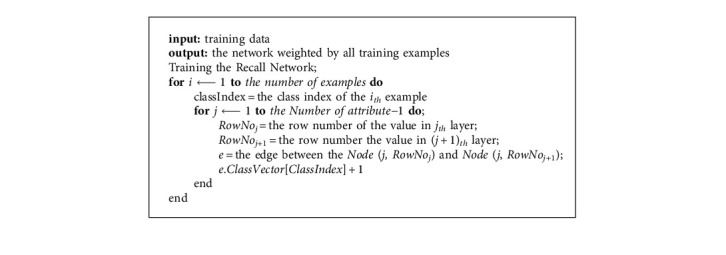
Teaching the recall network with the training dataset.

**Algorithm 3:**
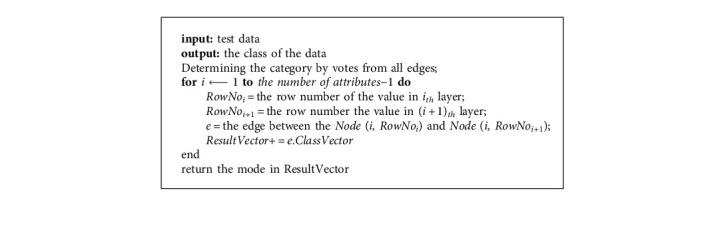
Predicting the class by a trained recall network.

**Table 1:** A classic example of playing tennis [10].

Weather					
Day	Outlook	Temperature	Humidity	Wind	PlayTennis
1	Sunny	Hot	High	Weak	No
2	Sunny	Hot	High	Strong	No
3	Overcast	Hot	High	Weak	Yes
4	Rain	Mild	High	Weak	Yes
5	Rain	Cool	Normal	Weak	Yes
6	Rain	Cool	Normal	Strong	No
*7*	Overcast	Cool	Normal	Strong	Yes
8	Sunny	Mild	High	Weak	No
9	Sunny	Cool	Normal	Weak	Yes
10	Rain	Mild	Normal	Weak	Yes
11	Sunny	Mild	Normal	Strong	Yes
12	Overcast	Mild	High	Strong	Yes
13	Overcast	Hot	Normal	Weak	Yes
14	Rain	Mild	High	Strong	No

**Table 2:** Comparison with similar algorithms in terms of mechanism, complexity, and structure (pictures of NN, MemNN, HTM, and ACO are copied from https://www.wikipedia.com; the time complexity and space complexity of ANN and MemNN are collected from [11–17]).

Algorithm	Mechanism	Time complexity	Space complexity	Structure
RN	Sum up the votes in an end-to-end route	*O* (*n* ∗ *d* ∗ *m*)	*O* (*n* ∗ *d*)	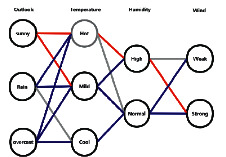

ANN	A linear combination of neurons and weights	*O*(∑_*l*=1_^*D*^*M*_*l*_^2^*∗K*_*l*_^2^*∗C*_*l*−1_*∗C*_*l*_)	*O*(∑_*l*=1_^*D*^*K*_*l*_^2^*∗C*_*l*−1_*∗C*_*l*_+∑_*l*=1_^*D*^*M*^2^*∗C*_*l*_)	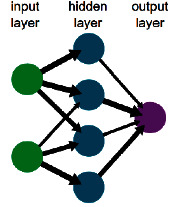

MemNN	Search the most relevant memory slot step by step until the answer is found	*O*(∑_*l*=1_^*D*^*n∗d*^2^)	*O*(∑_*l*=1_^*D*^*n*+*M*)	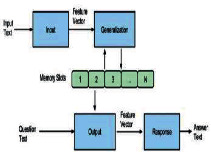

HTM	The lower level outputs the generated pattern to higher levels hierarchically	\	\	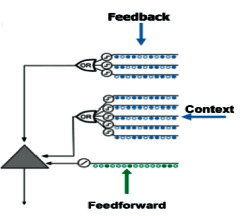

ACO	Good paths are encouraged to repeat while random exploration is still maintained	\	\	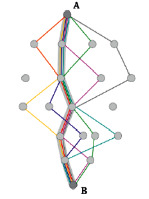

**Table 3:** Description of datasets.

No	Dataset	Examples	Attributes	Classes	Missingvalue	Numricvalue
1	Abalone	4177	9	3	N	Y
2	Absenteeism-at-work	740	20	2	N	Y
3	Acute-inflammation	120	7	2	N	Y
4	Acute-nephritis	120	7	2	N	Y
5	Adult	1628	15	2	N	Y
6	Aggregation	788	3	7	N	Y
7	Algerianforest	243	14	2	N	Y
8	Annealing	798	32	5	N	Y
9	Arrhythmia	452	263	13	N	Y
10	Au1 1000	1000	21	2	N	Y
11	Au4-2500	1000	101	3	N	Y
12	Au6-1000	1000	41	8	N	Y
13	Au6-250-drift-au6-cd1-500	750	41	8	N	Y
14	Au6-cd1-400	400	41	8	N	Y
15	Au7-300-drift-au7-cpd1-800	1100	13	5	N	Y
16	Au7-700	700	13	5	N	Y
17	Au7-cpd1-500	500	13	5	N	Y
18	Audiology-std	171	60	18	N	Y
19	Audit-risk	776	25	2	Y	Y
20	Autism-adolescent-data	104	21	2	Y	N
21	Autism-adult-data	704	21	2	Y	N
22	Autism-child-data	292	21	2	Y	N
23	Autos	205	26	7	Y	Y
24	Avila	4172	11	12	N	Y
25	Balance-scale	625	5	3	N	Y
26	Balloons	16	5	2	N	Y
27	Bank	4521	17	2	N	Y
28	Blood	748	5	2	N	Y
29	Breast-cancer-wisc-diag	569	31	2	N	Y
30	Breast-cancer-wisc-prog	198	34	2	N	Y
31	Breast-cancer-wisc	699	10	2	N	Y
32	Breast-cancer	286	10	2	N	Y
33	Breast-tissue	106	10	6	N	Y
34	Bupa	345	7	2	N	Y
35	Caesarean	80	6	2	N	N
36	Car	1728	7	4	N	Y
37	Cardiotocography-10classes	2126	22	10	N	Y
38	Cardiotocography-3classes	2126	22	3	N	Y
39	Cervical-cancer	72	20	2	N	Y
40	Chemicalcomposionofceramic	88	18	2	N	Y
41	Chess-krvk	2805	7	18	N	Y
42	Chess-krvkp	3196	37	2	N	Y
43	Congressional-voting	435	17	2	N	Y
44	Conn-bench-sonar-mines-rocks	208	61	2	N	Y
45	Conn-bench-vowel-deterding	528	12	11	N	Y
46	Connect-4	3377	43	2	N	Y
47	Connectionist	207	61	2	N	Y
48	Contrac	1473	10	3	N	Y
49	COVID-19	14	8	3	N	N
50	Credit-approval	690	16	2	N	Y
51	Crowdsource	2109	29	6	N	Y
52	Crx	690	16	2	Y	N
53	Cryother	90	7	2	N	Y
54	Cylinder-bands	512	36	2	N	Y
55	Dbworld-bodies	64	4703	2	N	N
56	Dbworld-bodies-stemmed	64	3722	2	N	N
57	Dbworld-subjects	64	243	2	N	N
58	Dbworld-subjects-stemmed	64	230	2	N	N
59	Dermatology	366	35	6	N	Y
60	Diabetes	768	9	2	N	Y
61	Diabetic	1151	20	2	N	Y
62	Divorce	170	55	2	N	N
63	Dota2train	926	116	2	N	N
64	Dow-jones-index	749	15	2	Y	Y
65	Dry-bean-dataset	3402	17	7	N	Y
66	Early-stage-diabetes-data-upload	520	17	2	N	N
67	Echocardiogram	131	11	2	N	Y
68	Ecoli	336	8	8	N	Y
69	Eegeyesate	14980	15	2	N	Y
70	Electrical	312	14	2	N	Y
71	Energy-y1	768	9	3	N	Y
72	Energy-y2	768	9	3	N	Y
73	Extentionofz-alizadehsani	303	59	2	N	Y
74	Fertility	100	10	2	N	Y
75	First-order	1529	57	2	N	Y
76	Flags	194	29	8	N	Y
77	Foresttypes	198	28	4	N	Y
78	Garments-worker-productivity	1197	14	5	Y	Y
79	Gender-name-dataset	4602	3	2	N	Y
80	Gesture-a1-raw	1747	20	5	N	Y
81	Gesture-a1-va3	1743	33	5	N	Y
82	Gesture-a2-raw	1264	20	5	N	Y
83	Gesture-a2-va3	1260	33	5	N	Y
84	Gesture-a3-raw	1834	20	5	N	Y
85	Gesture-a3-va3	1830	33	5	N	Y
86	Gesture-b1-raw	1073	20	6	N	Y
87	Gesture-b1-va3	1069	33	5	N	Y
88	Gesture-b3-raw	1424	20	5	N	Y
89	Gesture-b3-va3	1420	33	5	N	Y
90	Gesture-c1-raw	1111	20	5	N	Y
91	Gesture-c1-va3	1107	33	5	N	Y
92	Gesture-c3-raw	1448	20	5	N	Y
93	Gesture-c3-va3	1444	33	5	N	Y
94	Glass	214	10	6	N	Y
95	Go-track-tracks	163	9	2	N	Y
96	Haberman-survival	306	4	2	N	Y
97	Hayes-Roth	132	4	3	N	Y
98	Hcc-data	165	50	2	Y	N
99	Hcvdat	615	13	5	N	Y
100	Heart-Cleveland	303	14	5	N	Y
101	Heart-Hungarian	294	13	2	N	Y
102	Heart-Switzerland	123	13	5	N	Y
103	Heart-va	200	13	5	N	Y
104	Heart-failure-clinical-records-dataset	299	13	2	N	Y
105	Hepatitis	155	20	2	N	Y
106	Hill-valley	606	101	2	N	Y
107	Hiv1625data	1625	9	2	N	N
108	Hiv746data	746	9	2	N	N
109	Horse-colic	300	26	2	N	Y
110	Htru	17898	9	2	N	Y
111	Hypothyroid	3772	30	4	Y	N
112	Ibeacon-rssi-labeled	1420	14	105	N	Y
113	Ilpd-Indian-liver	583	10	2	N	Y
114	Image-segmentation	210	19	7	N	Y
115	Immunotherapy	90	8	2	N	Y
116	Impensdata	947	9	2	N	N
117	In-vehicle-coupon-recommendation	12684	24	2	Y	N
118	Indian	583	11	2	Y	Y
119	Ionosphere	351	34	2	N	Y
120	Iris	150	5	3	N	Y
121	Jain	372	3	2	N	Y
122	Jsbach-chorals-harmony	5665	15	2	N	N
123	Knowledge	172	6	4	N	Y
124	Lasvegastripadvisorreviews	504	19	7	N	N
125	Leaf	340	16	30	N	Y
126	Led-display	1000	8	10	N	Y
127	Lenses	24	5	3	N	Y
128	Letter	20000	17	26	N	Y
129	Libras	360	91	15	N	Y
130	Low-res-spect	531	101	9	N	Y
131	Lung-cancer	32	57	3	N	Y
132	Lymphography	148	19	4	N	Y
133	Magic	1902	11	2	N	Y
134	Mammographic	961	6	2	N	Y
135	Miniboone	1300	51	2	N	Y
136	Molec-biol-promoter	106	58	2	N	Y
137	Molec-biol-splice	3190	61	3	N	Y
138	Monks-1	124	7	2	N	Y
139	Monks-2	169	7	2	N	Y
140	Monks-3	122	7	2	N	Y
141	Mushroom	8124	22	2	N	Y
142	Musk-1	476	167	2	N	Y
143	Musk-2	3299	167	2	N	Y
144	Newdiagnosis	120	7	2	N	N
145	Nursery	12960	9	5	N	Y
146	Obesitydataset-raw-and-data-sinthetic	2111	17	7	N	N
147	Obs-network-dataset-2-aug27	1075	22	4	Y	Y
148	Occupancy-data	2665	7	2	N	Y
149	Occupancy-data2	9752	7	2	N	Y
150	Occupancy-data3	8143	7	2	N	Y
151	Old	2456	31	2	N	N
152	Online-shoppers-intention	12330	18	2	N	Y
153	Oocytes-merluccius-nucleus-4d	1022	42	2	N	Y
154	Oocytes-merluccius-states-2f	1022	26	3	N	Y
155	Oocytes-trisopterus-nucleus-2f	912	26	2	N	Y
156	Oocytes-trisopterus-states-5b	912	33	3	N	Y
157	Optdigits	5620	65	10	N	Y
158	Optical	3823	63	10	N	Y
159	Ozone	2536	73	2	N	Y
160	Page-blocks	5473	11	5	N	Y
161	Parkingbirmingham	3571	3	30	N	Y
162	Parkinsons	195	23	2	N	Y
163	Pasture	36	23	3	N	Y
164	Pbc	418	19	2	Y	Y
165	Pen	10992	17	10	N	Y
166	Pendigits	7494	17	10	N	Y
167	Pharynx	195	12	2	Y	N
168	Phishingwebsites	11055	31	2	N	N
169	Pima	768	9	2	N	Y
170	Pittsburg-bridges-rel-l	103	8	3	N	Y
171	Pittsburg-bridges-span	92	8	3	N	Y
172	Pittsburg-bridges-t-or-d	102	8	2	N	Y
173	Pittsburg-bridges-type	105	8	6	N	Y
174	Pittsburg-bridgesmaterial	106	8	3	N	Y
175	Planning	182	13	2	N	Y
176	Plant-margin	1600	65	100	N	Y
177	Plant-shape	1600	65	100	N	Y
178	Plant-texture	1599	65	100	N	Y
179	Poker-hand-training-true	25010	11	10	N	Y
180	Post-operative	90	9	3	N	Y
181	Primary-tumor	330	18	15	N	Y
182	Qsarbioconcentration	779	12	2	N	Y
183	Qsarbiodegradation	1055	42	2	N	Y
184	Qualitative-bankruptcy	250	7	2	N	N
185	Ringnorm	7400	21	2	N	Y
186	Risk-factors-cervical-cancer	858	36	2	Y	Y
187	Robotnavigation	5456	25	4	N	Y
188	Sapfile	131	22	3	N	N
189	Sat	6435	37	6	N	Y
190	Satellite	6435	37	6	N	Y
191	Scadi	70	206	7	N	N
192	Schillingdata	3272	9	2	N	N
193	Seeds	210	8	3	N	Y
194	Segment	2310	20	7	N	Y
195	Seismic-bumps	2584	19	2	N	Y
196	Semeion	1593	257	10	N	Y
197	Setapprocesst1	64	85	2	N	Y
198	Setapprocesst10	74	85	2	N	Y
199	Setapprocesst11	74	85	2	N	Y
200	Setapprocesst2	74	85	2	N	Y
201	Setapprocesst3	74	85	2	N	Y
202	Setapprocesst4	63	85	2	N	Y
203	Setapprocesst5	74	85	2	N	Y
204	Setapprocesst6	74	85	2	N	Y
205	Setapprocesst7	74	85	2	N	Y
206	Setapprocesst8	74	85	2	N	Y
207	Setapprocesst9	74	85	2	N	Y
208	Shillbiddingdataset	6321	9	2	N	Y
209	Shuttle-landing-control	15	7	2	N	N
210	Somervillehappinesssurvey2015	143	7	2	N	N
211	Sonar	208	61	2	N	Y
212	Soybean	307	36	18	N	Y
213	Spambase	4601	58	2	N	Y
214	Speaker-accent	329	13	6	N	Y
215	Spect	79	23	2	N	Y
216	Spectf	80	45	2	N	Y
217	Statlog-Australian-credit	690	15	2	N	Y
218	Statlog-German-credit	1000	25	2	N	Y
219	Statlog-heart	270	14	2	N	Y
220	Statlog-image	2310	19	7	N	Y
221	Statlog-landsat	4435	37	6	N	Y
222	Statlog-shuttle	43500	10	7	N	Y
223	Statlog-vehicle	846	19	4	N	Y
224	Steel-plates	1941	28	7	N	Y
225	Synthetic-control	600	61	6	N	Y
226	Teaching	151	6	3	N	Y
227	Thoraricsurgery	470	17	2	N	N
228	Thyroid	215	6	3	N	Y
229	Thyroid-train	3772	22	3	N	Y
230	Tic-tac-toe	958	10	2	N	Y
231	Titanic	2201	4	2	N	Y
232	Trains	10	30	2	N	Y
233	Transfusion	748	5	2	N	Y
234	Trial	776	17	2	Y	Y
235	Turkiye-student-evaluation	5820	34	3	N	Y
236	Unbalanced	856	33	2	N	Y
237	Urbanlandcover	168	148	9	N	Y
238	Userknowledgemodeling	252	6	4	N	Y
239	Vehicle	846	19	4	N	Y
240	Vertebral-column-2classes	310	7	2	N	Y
241	Vertebral-column-3classes	310	7	3	N	Y
242	Veteran	137	8	2	N	Y
243	Vowel	990	14	11	N	Y
244	Wall-following	5456	25	4	N	Y
245	Waveform-noise	5000	41	3	N	Y
246	Waveform	5000	22	3	N	Y
247	Wbc	683	10	2	N	N
248	Wdbc	569	31	2	N	Y
249	Weathernominal	14	5	2	N	N
250	Weathernumeric	14	5	2	N	N
251	Website-phishingdata	1353	10	3	N	N
252	Wholesalecustomersdata	440	8	2	N	Y
253	Wifi-localization	2000	8	4	N	Y
254	Wilt	4839	6	2	N	Y
255	Wine-quality-red	1599	12	6	N	Y
256	Wine-quality-white	4898	12	7	N	Y
257	Wine	178	14	3	N	Y
258	Yamilnaduelectricty	9156	4	20	N	Y
259	Yeast	1484	9	10	N	Y
260	Youtobe-kabita-preprocessing	4900	8	7	Y	Y
261	Youtobe-nisha-preprocessing	4900	8	7	N	Y
262	Z-alizadehsani	303	56	2	N	Y
263	Zoo	101	17	7	N	Y

**Table 4:** The parameters of SVM and ANN.

	Degree	Gamma	Cache	Cost	E-eps	*P* value
SVM1	3	0.5	40	1	0.001	0.1
SVM2	4	0.6	50	2	0.002	0.2
SVM3	5	0.7	60	3	0.001	0.3

	Learning rate			Momentum		

ANN1		0.3			0.2	
ANN2		0.4			0.3	

**Table 5:** Performance of all models among 263 datasets.

		Average accuracy (%)	Kappa statistic	Root mean squared error	Mean absolute error	Relative absolute error (%)	Root relative squared error (%)	Total running time (i7, 16G)
RN		73.57	0.42	0.33	0.27	79.08	83.25	3,780s
NB		72.05	0.50	0.34	0.19	60.74	85.51	60s
J48		78.53	0.59	0.28	0.16	44.76	70.59	180s
KNN		76.87	0.56	0.32	0.16	45.24	80.47	780s
SVM1		71.96	0.42	0.36	0.17	49.04	90.03	8,100s
SVM2		72.66	0.44	0.36	0.17	47.95	88.26	8,100s
SVM3		73.01	0.45	0.35	0.16	47.48	87.19	8,100s
ANN1		77.95	0.59	0.28	0.15	43.61	69.93	34.080s
ANN2		77.84	0.59	0.28	0.15	43.27	70.66	34.080s
OneR		67.16	0.40	0.39	0.19	56.74	98.86	60s

Friedman ranked test	Q (95% threshold: 21.67)	218.45	320.03	467.68	555.13	575.90	460.46	—

ANOVA	F (95% threshold: 1.88)	8.20	15.05	13.13	16.48	29.35	18.82	—

**Table 6:** The detailed experimental results on accuracy and standard deviation.

Dataset	RN	NB	J48	KNN	SVM1	SVM2	SVM3	ANN1	ANN2	OneR
Abalone	58.87 ± 2.79	57.65 ± 2.04	60.69 ± 1.97	57.94 ± 2.50	66.63 ± 2.47	66.98 ± 2.36	66.94 ± 2.25	65.55 ± 3.38	65.62 ± 4.13	60.95 ± 1.79
Absenteeism-at-work	98.92 ± 1.53	86.08 ± 3.61	99.73 ± 0.57	83.65 ± 2.88	84.73 ± 2.99	84.73 ± 2.99	84.73 ± 2.99	97.70 ± 1.11	96.89 ± 2.21	95.27 ± 1.72
Acute-inflammation	100.00 ± 0.00	100.00 ± 0.00	100.00 ± 0.00	100.00 ± 0.00	100.00 ± 0.00	100.00 ± 0.00	100.00 ± 0.00	100.00 ± 0.00	100.00 ± 0.00	79.17 ± 8.10
Acute-nephritis	100.00 ± 0.00	95.00 ± 7.03	100.00 ± 0.00	100.00 ± 0.00	100.00 ± 0.00	100.00 ± 0.00	100.00 ± 0.00	100.00 ± 0.00	100.00 ± 0.00	91.67 ± 7.86
Adult	76.04 ± 0.06	80.41 ± 2.49	81.76 ± 3.29	78.38 ± 3.59	83.29 ± 4.11	83.41 ± 4.15	83.35 ± 4.29	80.71 ± 3.27	80.10 ± 3.07	80.34 ± 1.30
Aggregation	84.64 ± 5.38	99.87 ± 0.40	99.62 ± 0.61	99.87 ± 0.41	99.87 ± 0.41	99.75 ± 0.54	99.75 ± 0.54	99.24 ± 0.89	99.24 ± 1.07	59.64 ± 2.89
Algerianforest	89.35 ± 7.86	94.67 ± 4.73	95.50 ± 4.53	88.10 ± 6.70	58.85 ± 1.69	61.73 ± 4.28	61.73 ± 4.28	95.92 ± 3.85	96.75 ± 4.58	97.15 ± 4.75
Annealing	76.19 ± 0.13	70.43 ± 4.45	96.50 ± 2.27	89.35 ± 2.13	88.98 ± 3.15	91.48 ± 2.54	92.11 ± 2.12	91.11 ± 3.57	90.86 ± 3.39	83.71 ± 0.09
Arrhythmia	54.20 ± 1.02	60.85 ± 7.44	64.38 ± 7.08	53.55 ± 3.26	61.72 ± 2.57	65.49 ± 3.47	64.60 ± 4.20	66.38 ± 5.17	56.26 ± 13.10	59.51 ± 2.65
Au1-1000	74.10 ± 0.32	72.80 ± 1.40	77.90 ± 4.43	66.70 ± 4.72	74.10 ± 0.32	74.10 ± 0.32	74.40 ± 0.84	68.80 ± 3.79	71.00 ± 3.68	74.10 ± 0.32
Au4-2500	62.60 ± 6.11	57.30 ± 4.30	72.40 ± 5.74	63.60 ± 4.35	61.50 ± 2.68	61.50 ± 2.68	61.50 ± 2.68	64.70 ± 4.88	64.90 ± 4.89	53.60 ± 4.27
Au6-1000	23.70 ± 1.34	18.70 ± 2.95	19.40 ± 4.03	13.40 ± 3.78	24.00 ± 0.00	24.00 ± 0.00	24.00 ± 0.00	14.60 ± 3.20	13.30 ± 4.14	24.30 ± 2.21
Au6-250-drift-au6-cd1-500	22.13 ± 2.45	20.40 ± 4.75	19.33 ± 4.37	16.27 ± 4.25	22.00 ± 0.70	22.00 ± 0.70	22.00 ± 0.70	14.93 ± 2.80	15.73 ± 3.31	24.53 ± 3.45
Au6-cd1-400	28.75 ± 6.69	25.75 ± 6.35	37.00 ± 6.85	17.75 ± 7.95	27.75 ± 0.79	27.75 ± 0.79	27.75 ± 0.79	21.00 ± 6.26	20.00 ± 6.01	31.75 ± 6.57
Au7-300-drift-au7-cpd1-800	28.64 ± 4.12	34.18 ± 3.72	37.91 ± 4.35	32.00 ± 4.26	28.36 ± 1.65	27.64 ± 1.93	27.64 ± 1.93	33.45 ± 3.58	35.36 ± 4.30	24.91 ± 3.10
Au7-700	41.00 ± 6.32	42.00 ± 5.35	52.29 ± 4.00	35.57 ± 5.57	37.29 ± 2.89	36.43 ± 2.36	36.43 ± 2.36	41.29 ± 5.24	43.57 ± 3.76	44.71 ± 3.81
Au7-cpd1-500	39.40 ± 2.50	38.20 ± 5.92	53.40 ± 4.62	37.80 ± 5.69	38.20 ± 1.14	38.60 ± 1.65	38.60 ± 1.65	39.40 ± 6.40	38.00 ± 5.66	40.20 ± 4.85
Audiology-std	28.07 ± 4.62	73.04 ± 10.91	76.05 ± 7.46	74.18 ± 11.92	63.14 ± 7.94	67.81 ± 10.16	70.75 ± 8.33	84.15 ± 6.29	83.01 ± 4.43	47.39 ± 2.12
Audit-risk	90.72 ± 3.88	94.59 ± 1.98	99.87 ± 0.41	97.81 ± 1.22	98.45 ± 1.33	98.71 ± 1.35	98.84 ± 1.41	96.91 ± 2.72	96.78 ± 2.72	100.00 ± 0.00
Autism-adolescent-data	80.82 ± 7.86	98.00 ± 4.22	100.00 ± 0.00	90.45 ± 6.39	96.18 ± 4.94	95.27 ± 4.99	96.18 ± 4.94	89.45 ± 10.34	89.45 ± 10.34	100.00 ± 0.00
Autism-adult-data	83.94 ± 3.42	97.01 ± 2.64	100.00 ± 0.00	94.89 ± 3.84	99.29 ± 1.00	99.57 ± 0.96	99.43 ± 1.00	100.00 ± 0.00	100.00 ± 0.00	100.00 ± 0.00
Autism-child-data	91.75 ± 5.20	98.97 ± 1.67	100.00 ± 0.00	88.34 ± 3.74	99.66 ± 1.09	100.00 ± 0.00	100.00 ± 0.00	99.66 ± 1.09	99.66 ± 1.09	100.00 ± 0.00
Autos	71.67 ± 10.00	56.62 ± 12.80	82.88 ± 7.79	76.43 ± 10.04	34.64 ± 4.34	35.62 ± 5.21	35.62 ± 5.21	77.00 ± 6.32	77.02 ± 4.82	62.36 ± 9.87
Avila	97.20 ± 0.76	27.16 ± 2.24	94.01 ± 1.24	75.60 ± 2.71	67.76 ± 2.13	69.94 ± 1.87	71.38 ± 2.31	61.72 ± 1.71	61.98 ± 3.05	71.45 ± 2.52
Balance-scale	79.67 ± 2.45	90.39 ± 1.72	76.65 ± 3.77	80.97 ± 4.45	90.40 ± 1.53	92.80 ± 0.86	93.60 ± 1.32	90.70 ± 3.79	91.02 ± 3.75	56.33 ± 4.39
Balloons	65.00 ± 41.16	60.00 ± 31.62	65.00 ± 33.75	75.00 ± 35.36	70.00 ± 34.96	80.00 ± 34.96	80.00 ± 34.96	75.00 ± 26.35	75.00 ± 26.35	45.00 ± 36.89
Bank	88.48 ± 0.06	83.70 ± 1.10	88.79 ± 1.14	86.09 ± 1.35	89.54 ± 0.61	89.69 ± 0.56	89.83 ± 1.06	88.59 ± 0.89	88.39 ± 1.65	88.56 ± 0.62
Blood	76.88 ± 1.34	74.47 ± 2.97	77.81 ± 3.77	69.91 ± 4.27	78.48 ± 2.09	79.01 ± 1.54	78.48 ± 1.44	78.21 ± 2.47	78.48 ± 2.80	76.34 ± 1.66
Breast-cancer-wisc-diag	93.68 ± 3.71	92.98 ± 4.30	93.33 ± 3.94	95.96 ± 1.87	97.54 ± 2.22	97.89 ± 1.99	98.24 ± 1.17	96.13 ± 2.00	96.48 ± 2.04	89.11 ± 4.11
Breast-cancer-wisc-prog	76.29 ± 2.10	67.66 ± 11.40	74.24 ± 7.29	72.79 ± 9.05	79.32 ± 6.01	78.26 ± 8.95	77.79 ± 9.57	73.79 ± 10.91	74.26 ± 13.10	70.11 ± 7.77
Breast-cancer-wisc	96.13 ± 2.44	95.85 ± 1.72	93.85 ± 3.16	95.56 ± 2.97	97.14 ± 1.91	96.99 ± 1.96	96.71 ± 1.91	94.85 ± 3.76	95.57 ± 2.07	92.71 ± 4.54
Breast-cancer	70.64 ± 1.49	72.03 ± 5.96	71.71 ± 4.22	70.28 ± 7.73	73.83 ± 5.25	73.13 ± 5.47	72.07 ± 5.25	72.45 ± 6.54	68.90 ± 2.76	70.32 ± 5.22
Breast-tissue	68.00 ± 13.75	66.36 ± 14.92	66.45 ± 15.65	71.64 ± 13.94	58.36 ± 14.47	67.82 ± 16.52	69.82 ± 16.67	64.09 ± 8.98	63.18 ± 15.76	54.55 ± 9.71
Bupa	67.55 ± 5.03	57.17 ± 8.53	62.87 ± 9.01	61.75 ± 4.95	58.86 ± 2.27	59.15 ± 2.45	59.15 ± 2.45	66.66 ± 7.58	69.29 ± 6.28	54.24 ± 7.87
Caesarian	73.75 ± 13.76	65.00 ± 21.08	57.50 ± 17.87	61.25 ± 18.11	55.00 ± 6.45	62.50 ± 11.79	58.75 ± 16.72	56.25 ± 19.76	56.25 ± 20.62	48.75 ± 18.11
Car	70.02 ± 0.17	84.32 ± 3.29	97.57 ± 1.01	96.59 ± 1.17	97.40 ± 1.13	98.09 ± 1.16	98.32 ± 0.96	93.23 ± 1.59	93.46 ± 1.71	70.02 ± 0.17
Cardiotocography-10classes	66.65 ± 2.68	70.93 ± 2.91	83.35 ± 2.43	78.65 ± 2.97	81.23 ± 2.09	82.41 ± 2.56	83.11 ± 2.07	82.92 ± 3.08	81.42 ± 3.06	47.60 ± 2.30
Cardiotocography-3classes	77.89 ± 0.23	82.08 ± 3.32	92.90 ± 2.51	92.05 ± 1.78	91.06 ± 1.93	92.05 ± 2.09	92.19 ± 1.75	92.19 ± 1.02	92.29 ± 2.33	81.42 ± 3.18
Cervical-cancer	76.61 ± 8.44	90.36 ± 8.92	84.64 ± 13.89	88.75 ± 11.23	70.89 ± 3.16	73.75 ± 7.05	73.75 ± 7.05	92.86 ± 10.10	92.86 ± 10.10	77.86 ± 9.07
Chemicalcomposionofceramic	100.00 ± 0.00	100.00 ± 0.00	98.75 ± 3.95	100.00 ± 0.00	45.56 ± 2.34	45.56 ± 2.34	45.56 ± 2.34	100.00 ± 0.00	100.00 ± 0.00	100.00 ± 0.00
Chess-krvk	27.84 ± 2.04	30.87 ± 2.03	51.87 ± 3.06	49.95 ± 3.30	46.02 ± 2.60	48.24 ± 2.24	49.77 ± 2.93	45.42 ± 2.55	43.89 ± 2.12	25.63 ± 2.11
Chess-krvkp	76.85 ± 1.29	85.54 ± 1.84	99.31 ± 0.51	95.28 ± 1.31	98.69 ± 0.91	99.19 ± 0.63	99.19 ± 0.53	99.19 ± 0.71	99.22 ± 0.69	66.46 ± 1.44
Congressional-voting	61.15 ± 1.12	57.67 ± 7.26	59.10 ± 3.13	59.56 ± 6.13	61.61 ± 4.05	61.14 ± 5.31	60.69 ± 5.89	59.75 ± 4.88	59.55 ± 5.00	62.98 ± 2.68
Conn-bench-sonar-mines-rocks	77.02 ± 7.19	68.36 ± 9.61	71.17 ± 7.10	86.57 ± 7.01	84.17 ± 7.06	87.02 ± 7.79	88.48 ± 7.85	80.81 ± 10.93	82.21 ± 8.70	63.45 ± 10.50
Conn-bench-vowel-deterding	75.57 ± 2.69	65.51 ± 5.57	79.54 ± 6.12	99.25 ± 1.32	94.13 ± 1.89	97.54 ± 1.79	98.49 ± 1.49	84.65 ± 2.64	82.76 ± 3.38	35.41 ± 4.94
Connect-4	74.53 ± 0.04	68.32 ± 2.04	79.98 ± 2.16	71.37 ± 2.71	74.39 ± 0.25	76.01 ± 0.95	77.17 ± 1.74	74.21 ± 2.00	75.93 ± 1.58	74.47 ± 0.14
Connectionist	78.69 ± 8.23	67.26 ± 11.21	74.33 ± 7.93	87.05 ± 5.86	61.40 ± 8.51	69.74 ± 11.51	72.67 ± 12.19	83.52 ± 9.07	83.55 ± 8.38	62.86 ± 6.95
Contrac	48.00 ± 3.06	48.89 ± 5.47	53.36 ± 3.41	43.25 ± 3.34	54.51 ± 2.22	54.38 ± 3.54	53.77 ± 3.87	54.51 ± 3.33	55.74 ± 3.31	48.00 ± 2.03
Covid-19	75.00 ± 35.36	60.00 ± 45.95	70.00 ± 42.16	60.00 ± 45.95	60.00 ± 39.44	55.00 ± 43.78	65.00 ± 41.16	65.00 ± 41.16	65.00 ± 41.16	80.00 ± 34.96
Credit-approval	82.03 ± 4.44	76.96 ± 3.83	84.93 ± 3.82	82.17 ± 5.64	85.36 ± 5.44	85.07 ± 4.83	84.64 ± 4.59	85.22 ± 2.72	84.20 ± 4.07	85.51 ± 4.48
Crowdsource	75.77 ± 1.42	81.41 ± 2.63	88.81 ± 1.49	95.31 ± 0.96	75.34 ± 1.06	75.34 ± 1.06	75.34 ± 1.06	92.60 ± 1.88	92.46 ± 2.22	78.52 ± 2.22
Crx	83.48 ± 4.89	77.68 ± 3.15	86.09 ± 3.75	81.16 ± 4.83	55.80 ± 3.57	56.52 ± 4.27	57.83 ± 5.13	83.04 ± 4.21	83.77 ± 2.88	85.51 ± 4.48
Cryother	90.00 ± 13.30	83.33 ± 15.93	93.33 ± 7.77	90.00 ± 8.20	82.22 ± 13.04	83.33 ± 13.09	83.33 ± 13.09	87.78 ± 9.73	86.67 ± 8.76	81.11 ± 10.54
Cylinder-bands	66.22 ± 3.19	67.59 ± 5.79	72.28 ± 6.11	69.16 ± 7.22	75.78 ± 3.85	79.09 ± 4.40	81.24 ± 3.14	73.05 ± 5.28	74.03 ± 5.50	66.61 ± 4.29
Dbworld-bodies	57.38 ± 9.15	75.48 ± 15.43	80.00 ± 16.81	59.29 ± 10.50	54.52 ± 5.55	54.52 ± 5.55	63.81 ± 12.69	/	/	86.43 ± 13.28
Dbworld-bodies-stemmed	57.38 ± 9.15	76.90 ± 12.45	86.67 ± 14.29	64.05 ± 14.74	54.52 ± 5.55	59.05 ± 9.17	73.81 ± 13.23	/	/	74.29 ± 20.32
Dbworld-sub jects	54.52 ± 5.55	89.52 ± 9.93	72.62 ± 12.15	80.24 ± 13.79	54.52 ± 5.55	54.52 ± 5.55	54.52 ± 5.55	89.52 ± 13.76	87.86 ± 15.49	62.62 ± 12.35
Dbworld-sub jects-stemmed	54.52 ± 5.55	86.43 ± 13.28	75.71 ± 17.45	83.33 ± 10.47	54.52 ± 5.55	54.52 ± 5.55	54.52 ± 5.55	87.62 ± 12.28	86.19 ± 11.49	60.95 ± 10.18
Dermatology	85.53 ± 4.82	97.54 ± 2.71	96.47 ± 3.38	95.35 ± 4.26	97.55 ± 2.37	97.83 ± 2.49	97.83 ± 2.79	98.36 ± 1.41	97.82 ± 1.71	49.73 ± 2.45
Diabetes	72.52 ± 4.14	76.31 ± 5.52	73.83 ± 5.66	70.17 ± 4.69	65.11 ± 0.36	65.11 ± 0.36	65.11 ± 0.36	75.40 ± 4.66	75.39 ± 4.38	71.48 ± 5.15
Diabetic	64.38 ± 4.52	56.82 ± 2.20	64.38 ± 4.58	61.33 ± 4.75	58.21 ± 4.78	59.43 ± 6.01	59.52 ± 5.71	72.03 ± 6.10	72.46 ± 5.82	53.26 ± 5.68
Divorce	97.65 ± 3.04	97.65 ± 3.04	95.29 ± 5.41	97.65 ± 3.04	97.65 ± 3.04	97.65 ± 3.04	97.65 ± 3.04	97.65 ± 3.04	97.65 ± 3.04	95.29 ± 5.41
Dota2train	52.70 ± 0.36	54.22 ± 3.46	54.12 ± 5.39	54.43 ± 4.15	52.70 ± 0.36	53.66 ± 3.61	52.59 ± 3.29	50.44 ± 3.50	52.60 ± 2.22	55.39 ± 3.63
Dow-jones-index	65.43 ± 4.15	50.34 ± 5.13	72.50 ± 4.09	54.61 ± 6.36	52.07 ± 0.22	52.07 ± 0.22	52.07 ± 0.22	54.60 ± 5.95	55.02 ± 4.73	56.48 ± 3.08
Dry-bean-dataset	93.30 ± 1.51	90.24 ± 1.50	93.06 ± 1.13	94.62 ± 1.12	61.52 ± 2.72	61.52 ± 2.72	61.52 ± 2.72	92.53 ± 1.66	92.65 ± 1.55	68.55 ± 1.91
Early-stage-diabetes-data-upload	84.04 ± 7.08	87.12 ± 3.01	95.96 ± 2.79	98.08 ± 2.22	94.23 ± 4.15	94.81 ± 4.06	95.38 ± 3.76	96.35 ± 2.93	96.15 ± 3.27	82.31 ± 4.33
Echocardiogram	68.68 ± 7.71	79.34 ± 10.97	78.57 ± 11.42	74.01 ± 13.23	81.65 ± 8.32	80.82 ± 11.09	80.82 ± 11.09	77.14 ± 12.43	77.86 ± 13.30	85.49 ± 6.73
Ecoli	71.76 ± 4.25	86.89 ± 5.32	83.93 ± 7.54	80.37 ± 6.38	86.59 ± 4.56	86.59 ± 4.33	87.18 ± 3.51	86.00 ± 5.28	84.82 ± 3.82	66.97 ± 6.83
Eegeyesate	67.94 ± 1.22	46.77 ± 3.26	84.50 ± 1.09	83.65 ± 2.03	55.12 ± 0.03	55.16 ± 0.06	55.16 ± 0.06	54.81 ± 3.76	55.07 ± 3.68	62.60 ± 1.18
Electrical	84.91 ± 7.28	97.46 ± 4.66	100.00 ± 0.00	91.32 ± 7.32	82.67 ± 7.52	82.67 ± 6.51	83.63 ± 6.21	98.71 ± 1.67	98.40 ± 1.69	99.68 ± 1.02
Energy-y1	81.25 ± 1.04	81.77 ± 3.33	97.53 ± 1.29	75.79 ± 4.02	88.67 ± 1.39	89.84 ± 1.60	89.84 ± 2.80	88.94 ± 1.74	88.02 ± 1.82	84.50 ± 1.89
Energy-y2	89.84 ± 3.06	82.82 ± 3.31	90.23 ± 2.77	76.31 ± 3.34	89.72 ± 2.06	90.76 ± 1.55	91.28 ± 1.50	91.01 ± 2.35	91.27 ± 2.82	88.41 ± 3.09
Extentionofz-alizadehsani	71.29 ± 1.47	93.40 ± 3.49	99.68 ± 1.02	90.45 ± 5.19	71.29 ± 1.47	71.29 ± 1.47	71.29 ± 1.47	98.03 ± 2.31	98.03 ± 2.31	86.49 ± 4.71
Fertility	88.00 ± 4.22	88.00 ± 4.22	85.00 ± 7.07	83.00 ± 11.60	88.00 ± 4.22	89.00 ± 5.68	89.00 ± 7.38	90.00 ± 9.43	88.00 ± 13.17	88.00 ± 4.22
First-order	90.45 ± 0.33	31.39 ± 3.84	100.00 ± 0.00	99.41 ± 0.48	90.45 ± 0.33	95.49 ± 1.46	97.97 ± 1.09	99.87 ± 0.41	99.87 ± 0.41	90.78 ± 1.71
Flags	47.97 ± 10.07	46.34 ± 7.61	60.95 ± 8.09	45.00 ± 8.94	52.61 ± 9.41	54.24 ± 10.31	53.24 ± 13.18	46.50 ± 12.51	49.00 ± 11.93	57.26 ± 7.25
Foresttypes	93.95 ± 4.58	94.92 ± 4.16	94.97 ± 5.77	95.45 ± 3.70	30.32 ± 2.45	32.34 ± 3.64	32.34 ± 3.64	96.92 ± 3.63	96.92 ± 3.63	82.26 ± 8.50
Garments-worker-productivity	44.44 ± 2.46	26.06 ± 2.62	47.04 ± 6.19	26.15 ± 3.50	35.50 ± 2.90	36.08 ± 2.91	36.84 ± 2.47	41.19 ± 5.54	41.11 ± 4.41	32.41 ± 3.71
Gender-name-dataset	60.24 ± 1.53	58.30 ± 6.51	60.23 ± 0.15	63.60 ± 1.70	64.19 ± 1.38	64.28 ± 2.02	64.34 ± 1.81	60.32 ± 0.10	58.26 ± 6.52	60.58 ± 1.04
Gesture-a1-raw	89.18 ± 2.11	74.13 ± 2.78	91.47 ± 2.11	96.34 ± 1.12	39.95 ± 0.30	39.95 ± 0.30	39.95 ± 0.30	91.53 ± 2.23	91.36 ± 1.41	96.74 ± 1.35
Gesture-a1-va3	68.10 ± 2.47	59.27 ± 3.52	66.16 ± 4.66	74.12 ± 2.76	39.82 ± 0.22	39.82 ± 0.22	39.82 ± 0.22	70.97 ± 1.66	70.57 ± 2.31	61.56 ± 2.06
Gesture-a2-raw	88.45 ± 1.91	68.67 ± 3.16	89.25 ± 3.08	94.63 ± 3.04	39.00 ± 0.27	39.00 ± 0.27	39.00 ± 0.27	89.48 ± 1.89	89.72 ± 2.25	94.70 ± 2.62
Gesture-a2-va3	61.98 ± 2.79	43.97 ± 8.73	57.06 ± 2.60	67.70 ± 4.13	38.81 ± 0.25	38.81 ± 0.25	38.81 ± 0.25	62.30 ± 3.43	61.35 ± 4.32	53.97 ± 4.10
Gesture-a3-raw	90.68 ± 1.99	61.45 ± 3.87	92.97 ± 1.22	96.29 ± 0.99	36.10 ± 0.24	36.10 ± 0.24	36.10 ± 0.24	89.53 ± 1.45	88.77 ± 2.50	95.31 ± 1.16
Gesture-a3-va3	60.38 ± 2.32	50.22 ± 2.53	75.96 ± 3.14	89.89 ± 1.77	35.96 ± 0.23	35.96 ± 0.23	35.96 ± 0.23	65.46 ± 3.50	66.45 ± 2.70	50.93 ± 2.39
Gesture-b1-raw	91.52 ± 2.94	48.56 ± 4.91	92.73 ± 2.78	94.87 ± 2.87	38.68 ± 0.65	38.86 ± 0.79	38.86 ± 0.79	87.51 ± 4.31	89.38 ± 1.98	91.42 ± 2.24
Gesture-b1-va3	61.19 ± 5.74	34.80 ± 7.18	69.88 ± 4.24	90.56 ± 2.69	38.54 ± 0.39	38.54 ± 0.39	38.54 ± 0.39	61.84 ± 3.40	63.70 ± 4.83	42.57 ± 4.35
Gesture-b3-raw	89.40 ± 2.92	57.65 ± 4.22	91.08 ± 3.43	95.51 ± 0.88	33.08 ± 0.34	33.08 ± 0.34	33.08 ± 0.34	88.41 ± 4.10	87.64 ± 3.40	95.36 ± 2.35
Gesture-b3-va3	46.20 ± 3.58	43.66 ± 3.96	57.39 ± 3.97	78.03 ± 2.93	33.03 ± 0.22	33.03 ± 0.22	33.03 ± 0.22	58.10 ± 5.47	55.92 ± 5.05	40.07 ± 5.49
Gesture-c1-raw	86.41 ± 3.53	63.64 ± 2.59	89.29 ± 2.26	94.33 ± 1.68	25.74 ± 0.45	25.74 ± 0.45	25.74 ± 0.45	87.58 ± 3.36	87.13 ± 3.58	93.79 ± 2.60
Gesture-c1-va3	52.84 ± 4.08	50.50 ± 3.89	57.81 ± 3.46	70.37 ± 3.45	25.47 ± 0.36	25.47 ± 0.36	25.47 ± 0.36	64.23 ± 3.13	62.51 ± 4.15	41.46 ± 4.63
Gesture-c3-raw	88.26 ± 2.66	60.08 ± 5.35	87.77 ± 3.54	93.02 ± 2.33	27.00 ± 0.22	27.00 ± 0.22	27.00 ± 0.22	85.91 ± 3.16	83.70 ± 2.24	94.34 ± 1.34
Gesture-c3-va3	48.13 ± 3.44	45.50 ± 2.38	54.91 ± 4.00	62.39 ± 4.71	26.94 ± 0.21	26.94 ± 0.21	26.94 ± 0.21	57.41 ± 4.08	56.10 ± 5.72	42.59 ± 2.76
Glass	65.80 ± 11.01	52.27 ± 9.05	67.29 ± 8.50	70.52 ± 8.94	72.88 ± 6.56	71.90 ± 8.50	70.97 ± 8.39	67.25 ± 9.72	68.20 ± 5.31	57.08 ± 8.98
Go-track-tracks	83.60 ± 10.36	82.87 ± 4.54	87.68 ± 7.71	90.92 ± 8.57	72.02 ± 12.71	71.99 ± 11.53	71.99 ± 11.53	84.12 ± 10.34	84.74 ± 8.99	75.55 ± 13.56
Haberman-survival	73.51 ± 3.08	75.13 ± 4.07	71.86 ± 4.08	67.68 ± 6.29	72.55 ± 4.65	71.25 ± 2.91	71.25 ± 2.91	72.85 ± 6.08	73.85 ± 6.62	72.88 ± 3.00
Hayes-roth	65.88 ± 10.45	74.84 ± 13.29	80.27 ± 6.51	81.04 ± 6.53	86.32 ± 7.10	81.04 ± 6.53	80.27 ± 6.51	71.04 ± 13.20	70.27 ± 13.54	43.96 ± 5.89
Hcc-data	63.68 ± 4.52	68.97 ± 8.63	60.70 ± 7.64	63.64 ± 8.02	61.84 ± 2.26	61.84 ± 2.26	61.84 ± 2.26	64.45 ± 14.82	64.41 ± 10.82	69.15 ± 11.07
Hcvdat	87.48 ± 1.05	91.71 ± 3.64	92.04 ± 2.21	90.57 ± 1.48	86.67 ± 0.63	86.67 ± 0.63	86.67 ± 0.63	93.00 ± 2.81	92.35 ± 3.00	88.62 ± 1.84
Heart-cleveland	54.15 ± 2.28	55.84 ± 10.42	52.55 ± 8.31	54.77 ± 6.51	57.51 ± 8.53	58.83 ± 9.07	57.53 ± 9.58	54.17 ± 6.97	54.49 ± 6.43	52.54 ± 7.34
Heart-hungarian	80.33 ± 7.12	80.97 ± 8.06	78.61 ± 6.72	77.55 ± 8.10	81.72 ± 7.63	82.38 ± 6.37	83.05 ± 6.50	77.86 ± 8.83	77.83 ± 7.95	78.98 ± 9.41
Heart-switzerland	43.78 ± 8.82	41.47 ± 11.31	41.41 ± 11.01	32.69 ± 14.13	39.04 ± 6.31	39.81 ± 5.62	41.54 ± 6.48	43.97 ± 14.45	44.68 ± 11.91	32.31 ± 12.00
Heart-va	29.00 ± 9.37	33.00 ± 10.06	33.00 ± 6.32	35.00 ± 7.82	34.50 ± 9.56	32.00 ± 8.56	30.00 ± 9.43	29.50 ± 10.39	28.50 ± 10.55	27.50 ± 9.79
Heart-failure-clinical-records-dataset	69.23 ± 2.10	76.94 ± 6.31	80.61 ± 5.13	65.54 ± 6.74	67.90 ± 1.62	67.90 ± 1.62	67.90 ± 1.62	73.56 ± 4.93	77.30 ± 9.22	85.61 ± 6.32
Hepatitis	79.37 ± 2.38	83.79 ± 8.93	79.96 ± 8.81	82.50 ± 5.54	83.17 ± 6.33	84.38 ± 9.31	84.38 ± 9.31	81.96 ± 9.33	78.75 ± 11.39	74.17 ± 6.82
Hill-valley	49.82 ± 5.84	48.67 ± 2.58	50.33 ± 1.11	51.81 ± 3.48	52.16 ± 6.58	52.15 ± 7.44	53.14 ± 6.99	56.14 ± 7.60	53.14 ± 2.72	47.51 ± 4.93
Hiv1625data	93.54 ± 1.84	93.72 ± 1.70	90.95 ± 2.44	92.06 ± 1.96	92.18 ± 2.00	93.41 ± 2.12	93.72 ± 2.33	95.26 ± 2.08	93.60 ± 2.31	79.32 ± 1.71
Hiv746data	92.37 ± 3.22	90.89 ± 3.68	82.58 ± 4.76	86.74 ± 3.36	89.55 ± 3.71	90.89 ± 2.80	91.03 ± 3.27	92.50 ± 2.51	92.50 ± 3.26	80.97 ± 4.35
Horse-colic	74.00 ± 3.06	72.67 ± 5.62	87.00 ± 5.08	76.67 ± 7.54	83.67 ± 4.83	84.67 ± 5.26	85.00 ± 5.72	79.33 ± 6.05	79.67 ± 7.93	82.33 ± 5.45
Htru	97.06 ± 0.49	94.50 ± 0.50	97.84 ± 0.51	97.14 ± 0.38	91.14 ± 0.14	91.48 ± 0.19	91.38 ± 0.18	97.97 ± 0.40	97.95 ± 0.36	97.60 ± 0.40
Hypothyroid	92.29 ± 0.08	95.28 ± 0.83	99.58 ± 0.29	91.52 ± 1.55	92.60 ± 0.31	92.74 ± 0.72	92.71 ± 0.78	93.82 ± 4.15	92.92 ± 4.46	96.24 ± 0.94
Ibeacon-rssi-labeled	24.15 ± 2.44	19.86 ± 2.15	26.34 ± 3.14	37.18 ± 3.84	34.15 ± 1.70	36.55 ± 2.81	37.75 ± 3.14	27.54 ± 3.58	27.54 ± 3.99	4.51 ± 1.73
Ilpd-indian-liver	71.53 ± 1.39	55.91 ± 5.24	66.06 ± 5.25	64.83 ± 5.89	71.36 ± 0.76	70.67 ± 1.74	70.83 ± 2.51	68.95 ± 4.78	68.95 ± 6.02	66.05 ± 2.99
Image-segmentation	88.10 ± 5.14	77.62 ± 5.52	89.05 ± 7.79	87.14 ± 4.52	87.14 ± 3.21	87.14 ± 4.52	86.67 ± 4.38	90.00 ± 4.74	90.00 ± 4.74	59.05 ± 8.75
Immunotherapy	78.89 ± 3.51	75.56 ± 10.21	82.22 ± 10.73	70.00 ± 16.60	78.89 ± 3.51	78.89 ± 3.51	78.89 ± 3.51	81.11 ± 11.77	78.89 ± 8.20	84.44 ± 9.37
Impensdata	85.64 ± 1.11	90.07 ± 2.47	84.27 ± 0.30	87.54 ± 2.46	84.27 ± 0.30	84.27 ± 0.30	84.27 ± 0.30	90.28 ± 2.24	89.33 ± 2.60	84.37 ± 2.08
In-vehicle-coupon-recommendation	57.36 ± 0.20	65.59 ± 1.54	70.84 ± 1.14	63.99 ± 1.53	68.89 ± 1.32	69.42 ± 1.31	69.84 ± 1.32	70.46 ± 1.04	71.29 ± 1.09	60.53 ± 0.88
Indian	71.70 ± 0.88	55.74 ± 5.17	68.97 ± 5.22	64.12 ± 6.81	72.38 ± 1.58	71.69 ± 1.34	71.69 ± 1.34	68.26 ± 5.88	67.91 ± 7.11	65.88 ± 2.93
Ionosphere	88.31 ± 3.17	82.62 ± 5.47	91.46 ± 3.27	86.33 ± 4.59	94.31 ± 2.30	94.89 ± 3.18	95.75 ± 3.00	90.32 ± 2.39	90.90 ± 3.45	80.92 ± 7.47
Iris	81.33 ± 11.24	93.33 ± 5.44	96.00 ± 5.62	95.33 ± 5.49	96.67 ± 3.51	96.00 ± 4.66	96.67 ± 3.51	97.33 ± 3.44	97.33 ± 3.44	92.00 ± 6.13
Jain	93.53 ± 4.26	94.35 ± 4.31	99.46 ± 1.71	100.00 ± 0.00	100.00 ± 0.00	100.00 ± 0.00	100.00 ± 0.00	95.15 ± 3.57	95.15 ± 3.33	95.16 ± 3.32
Jsbach-chorals-harmony	88.37 ± 0.05	96.86 ± 0.55	96.52 ± 0.50	97.49 ± 0.54	96.26 ± 0.61	96.91 ± 0.45	97.19 ± 0.68	97.56 ± 0.59	97.18 ± 0.87	95.96 ± 0.62
Knowledge	61.08 ± 7.48	87.19 ± 11.04	92.35 ± 6.82	75.00 ± 8.31	83.73 ± 9.90	88.92 ± 8.89	91.83 ± 7.91	91.83 ± 5.64	92.42 ± 5.53	82.52 ± 9.66
Lasvegastripadvisorreviews	14.67 ± 3.20	16.47 ± 4.40	16.30 ± 5.05	15.68 ± 3.96	13.47 ± 3.37	11.68 ± 4.80	14.07 ± 4.82	15.69 ± 6.51	16.07 ± 5.08	14.29 ± 3.25
Leaf	58.82 ± 5.88	72.06 ± 8.57	60.88 ± 8.09	3.24 ± 3.24	17.06 ± 4.11	24.12 ± 5.15	25.59 ± 5.55	50.88 ± 7.34	48.24 ± 7.99	23.82 ± 4.89
Led-display	70.00 ± 3.62	72.00 ± 2.75	71.10 ± 3.25	70.90 ± 3.28	71.90 ± 4.09	71.70 ± 3.80	71.40 ± 3.66	71.80 ± 3.46	72.00 ± 3.89	19.60 ± 1.26
Lenses	68.33 ± 33.75	68.33 ± 33.75	78.33 ± 33.38	86.67 ± 21.94	78.33 ± 29.45	83.33 ± 22.22	83.33 ± 22.22	71.67 ± 26.12	71.67 ± 26.12	66.67 ± 30.43
Letter	71.68 ± 0.73	64.01 ± 0.74	87.91 ± 0.65	95.90 ± 0.29	94.88 ± 0.25	96.00 ± 0.30	96.51 ± 0.22	82.45 ± 1.17	81.78 ± 1.08	17.25 ± 0.37
Libras	66.94 ± 9.66	63.33 ± 9.96	69.72 ± 10.43	85.83 ± 4.98	81.11 ± 4.10	85.28 ± 4.35	86.94 ± 3.94	80.56 ± 5.24	81.11 ± 5.37	21.11 ± 6.03
Low-res-spect	66.65 ± 3.88	80.42 ± 3.96	83.24 ± 3.40	83.79 ± 4.03	89.45 ± 2.41	89.64 ± 2.72	90.58 ± 2.53	92.47 ± 3.33	91.71 ± 2.55	73.83 ± 5.30
Lung-cancer	56.67 ± 21.08	66.67 ± 20.79	40.83 ± 26.77	50.83 ± 36.53	53.33 ± 21.94	59.17 ± 21.68	51.67 ± 25.09	46.67 ± 28.11	49.17 ± 24.36	47.50 ± 17.59
Lymphography	76.24 ± 7.04	82.38 ± 8.55	74.90 ± 8.12	75.67 ± 13.05	85.86 ± 9.25	85.10 ± 9.92	83.14 ± 8.96	81.00 ± 9.18	79.62 ± 8.77	75.67 ± 8.03
Magic	76.45 ± 1.75	73.34 ± 2.49	82.60 ± 2.56	79.65 ± 2.34	84.75 ± 2.15	85.02 ± 1.74	85.02 ± 1.42	84.28 ± 1.79	84.02 ± 1.85	71.87 ± 4.46
Mammographic	78.87 ± 3.27	78.46 ± 3.58	82.00 ± 2.11	75.54 ± 5.00	82.73 ± 2.13	82.32 ± 2.59	82.52 ± 2.64	81.69 ± 2.69	81.17 ± 2.53	81.89 ± 1.25
Miniboone	79.08 ± 1.98	29.15 ± 1.38	85.92 ± 2.43	83.54 ± 1.59	84.54 ± 2.47	85.62 ± 2.12	86.38 ± 1.85	84.15 ± 1.71	83.85 ± 1.81	81.77 ± 1.78
Molec-biol-promoter	88.55 ± 16.48	88.45 ± 10.93	74.45 ± 10.04	72.45 ± 12.08	82.64 ± 13.45	84.64 ± 11.94	84.64 ± 11.94	78.36 ± 10.92	77.45 ± 10.10	69.73 ± 11.09
Molec-biol-splice	51.88 ± 0.17	92.35 ± 1.71	92.63 ± 1.92	63.10 ± 2.08	87.12 ± 1.97	87.27 ± 1.71	86.74 ± 1.30	84.70 ± 2.26	84.33 ± 1.77	63.32 ± 2.27
Monks-1	91.86 ± 9.63	71.54 ± 11.51	96.79 ± 6.76	70.13 ± 10.31	80.64 ± 12.11	83.08 ± 10.93	83.14 ± 9.59	88.65 ± 14.95	89.55 ± 17.20	73.27 ± 9.82
Monks-2	62.13 ± 2.93	56.18 ± 10.96	76.91 ± 10.92	61.03 ± 10.94	58.01 ± 7.39	60.92 ± 12.54	64.49 ± 12.42	73.46 ± 12.38	74.56 ± 7.84	58.60 ± 4.55
Monks-3	93.40 ± 8.60	88.53 ± 5.76	93.40 ± 8.60	74.55 ± 16.38	90.19 ± 7.56	89.29 ± 7.96	89.29 ± 7.96	84.23 ± 10.77	89.23 ± 6.89	77.88 ± 3.84
Mushroom	90.52 ± 1.06	88.84 ± 1.14	100.00 ± 0.00	100.00 ± 0.00	100.00 ± 0.00	100.00 ± 0.00	100.00 ± 0.00	100.00 ± 0.00	100.00 ± 0.00	98.52 ± 0.48
Musk-1	84.25 ± 5.58	73.99 ± 6.97	85.11 ± 6.59	84.89 ± 4.56	90.77 ± 4.31	92.43 ± 2.86	93.69 ± 2.99	94.33 ± 2.60	93.49 ± 3.02	62.39 ± 8.95
Musk-2	87.81 ± 0.85	85.09 ± 1.67	95.42 ± 1.53	96.18 ± 0.96	96.09 ± 1.20	97.42 ± 0.95	98.06 ± 0.76	98.88 ± 0.69	98.73 ± 0.90	89.72 ± 1.26
Newdiagnosis	100.00 ± 0.00	100.00 ± 0.00	100.00 ± 0.00	100.00 ± 0.00	100.00 ± 0.00	100.00 ± 0.00	100.00 ± 0.00	100.00 ± 0.00	100.00 ± 0.00	77.50 ± 6.86
Nursery	88.17 ± 1.35	89.75 ± 0.98	99.49 ± 0.27	88.98 ± 0.91	98.24 ± 0.22	98.67 ± 0.21	98.94 ± 0.20	95.73 ± 0.88	94.77 ± 1.30	70.97 ± 1.03
Obesitydataset-raw-and-data-sinthetic	83.04 ± 2.29	67.41 ± 2.79	93.75 ± 1.94	82.05 ± 2.48	89.72 ± 2.33	91.71 ± 2.24	92.47 ± 2.22	94.37 ± 2.75	94.22 ± 1.46	67.08 ± 3.58
Obs-network-dataset-2-aug27	96.00 ± 1.70	71.82 ± 5.31	99.63 ± 1.17	99.07 ± 0.62	97.68 ± 0.65	97.68 ± 0.65	97.68 ± 0.65	96.56 ± 1.53	98.05 ± 1.19	90.69 ± 2.07
Occupancy-data	96.13 ± 0.92	95.53 ± 1.02	98.61 ± 0.81	99.14 ± 0.50	80.94 ± 2.01	82.10 ± 2.31	82.10 ± 2.31	97.94 ± 0.87	98.01 ± 0.90	98.69 ± 0.77
Occupancy-data2	98.41 ± 0.43	96.03 ± 0.55	99.44 ± 0.16	99.47 ± 0.23	83.55 ± 0.50	85.65 ± 0.59	85.65 ± 0.59	99.43 ± 0.21	99.42 ± 0.15	99.37 ± 0.16
Occupancy-data3	98.40 ± 0.63	97.70 ± 0.55	99.51 ± 0.10	99.46 ± 0.23	82.91 ± 0.53	84.51 ± 0.61	84.53 ± 0.61	98.76 ± 0.37	98.77 ± 0.30	99.37 ± 0.18
Old	88.60 ± 1.80	94.05 ± 0.97	94.99 ± 1.72	97.68 ± 0.82	95.07 ± 0.93	95.19 ± 1.27	95.48 ± 1.19	96.95 ± 0.97	96.86 ± 1.12	88.68 ± 1.62
Online-shoppers-intention	84.53 ± 0.03	81.60 ± 1.30	89.53 ± 0.53	81.53 ± 0.57	84.50 ± 0.06	84.45 ± 0.13	84.40 ± 0.15	89.38 ± 0.85	88.86 ± 1.21	88.23 ± 0.98
Oocytes-merluccius-nucleus-4d	72.02 ± 2.48	59.98 ± 4.09	75.64 ± 2.43	72.22 ± 3.90	77.30 ± 2.90	80.13 ± 2.51	81.01 ± 3.02	81.41 ± 2.04	80.62 ± 4.38	66.83 ± 5.20
Oocytes-merluccius-states-2f	88.55 ± 3.21	84.64 ± 4.04	90.51 ± 3.40	91.09 ± 2.84	91.68 ± 2.59	92.07 ± 2.10	92.76 ± 1.97	91.78 ± 1.69	91.29 ± 2.76	81.89 ± 4.69
Oocytes-trisopterus-nucleus-2f	g64.46 ± 5.21	53.07 ± 5.55	72.16 ± 6.85	74.34 ± 5.80	81.15 ± 3.67	83.78 ± 3.41	83.34 ± 2.76	83.11 ± 3.97	82.12 ± 4.25	59.43 ± 5.98
Oocytes-trisopterus-states-5b	g83.13 ± 4.78	75.78 ± 4.23	88.60 ± 2.55	90.90 ± 3.69	91.78 ± 3.40	92.33 ± 2.74	92.77 ± 2.32	93.32 ± 2.68	93.32 ± 2.97	80.04 ± 2.74
Optdigits	88.83 ± 1.19	91.33 ± 1.01	90.69 ± 1.44	98.61 ± 0.64	73.13 ± 1.91	74.88 ± 2.08	74.88 ± 2.08	98.26 ± 0.71	98.43 ± 0.54	27.03 ± 1.23
Optical	88.47 ± 1.57	91.68 ± 1.31	89.72 ± 1.39	98.46 ± 0.57	98.59 ± 0.55	98.74 ± 0.60	98.72 ± 0.58	98.27 ± 0.54	98.20 ± 0.75	27.70 ± 1.30
Ozone	97.12 ± 0.19	71.10 ± 3.68	95.78 ± 0.53	95.11 ± 0.67	97.12 ± 0.19	97.12 ± 0.19	97.12 ± 0.19	96.22 ± 0.97	96.65 ± 0.89	96.85 ± 0.45
Page-blocks	92.84 ± 0.89	89.99 ± 1.43	96.88 ± 0.41	96.02 ± 0.60	96.18 ± 0.75	96.44 ± 0.75	96.47 ± 0.76	96.22 ± 0.70	96.20 ± 0.55	93.75 ± 0.60
Parkingbirmingham	81.18 ± 1.94	76.17 ± 2.07	100.00 ± 0.00	97.45 ± 0.67	58.69 ± 3.78	61.30 ± 3.91	61.30 ± 3.91	86.42 ± 1.54	87.88 ± 1.56	100.00 ± 0.00
Parkinsons	84.63 ± 5.40	69.34 ± 11.51	80.50 ± 3.33	96.39 ± 2.49	87.71 ± 6.39	89.79 ± 6.29	91.34 ± 5.80	90.63 ± 7.79	89.66 ± 8.93	86.18 ± 7.59
Pasture	71.67 ± 24.91	74.17 ± 23.72	78.33 ± 20.49	71.67 ± 27.55	28.33 ± 4.30	28.33 ± 4.30	28.33 ± 4.30	76.67 ± 25.09	74.17 ± 26.48	63.33 ± 26.41
Pbc	75.35 ± 4.83	77.72 ± 7.42	77.24 ± 8.31	59.58 ± 8.77	61.48 ± 0.79	61.48 ± 0.79	61.48 ± 0.79	71.06 ± 6.04	73.67 ± 4.02	72.25 ± 9.13
Pen	87.26 ± 0.61	85.75 ± 1.18	96.56 ± 0.53	99.36 ± 0.17	13.49 ± 0.86	14.47 ± 0.95	14.47 ± 0.95	94.62 ± 0.63	94.40 ± 0.43	38.95 ± 1.54
Pendigits	86.55 ± 1.02	87.99 ± 1.46	96.14 ± 0.75	99.43 ± 0.24	99.61 ± 0.19	99.67 ± 0.16	99.65 ± 0.17	95.17 ± 0.67	95.05 ± 0.54	39.74 ± 1.98
Pharynx	76.45 ± 2.19	68.24 ± 10.48	76.45 ± 2.19	64.08 ± 10.16	75.42 ± 8.61	73.29 ± 10.60	70.26 ± 10.68	71.79 ± 3.64	70.95 ± 16.41	27.13 ± 5.60
Phishingwebsites	85.07 ± 0.99	92.98 ± 0.64	95.88 ± 0.44	97.18 ± 0.45	94.50 ± 0.52	95.00 ± 0.50	95.26 ± 0.46	96.78 ± 0.38	96.83 ± 0.48	88.89 ± 0.56
Pima	72.00 ± 3.77	76.31 ± 5.55	73.83 ± 5.66	70.17 ± 4.69	75.66 ± 4.08	75.13 ± 4.01	75.00 ± 3.43	75.40 ± 4.66	75.39 ± 4.38	71.48 ± 5.15
Pittsburg-bridges-rel-l	69.09 ± 8.02	66.91 ± 11.95	63.00 ± 14.10	71.09 ± 16.91	67.18 ± 12.34	68.09 ± 12.39	72.82 ± 11.10	61.36 ± 14.27	66.18 ± 10.75	71.00 ± 10.59
Pittsburg-bridges-span	59.78 ± 8.98	67.33 ± 13.93	60.78 ± 13.17	55.00 ± 18.05	65.11 ± 14.63	64.00 ± 16.18	64.00 ± 16.18	66.22 ± 9.91	68.44 ± 13.31	54.67 ± 14.02
Pittsburg-bridges-t-or-d	86.36 ± 4.73	86.45 ± 9.83	83.55 ± 10.78	83.55 ± 11.76	86.45 ± 7.74	88.45 ± 8.44	89.45 ± 7.90	85.64 ± 12.49	83.64 ± 14.10	85.27 ± 9.69
Pittsburg-bridges-type	55.91 ± 11.28	55.91 ± 11.62	61.73 ± 9.31	57.82 ± 11.76	53.91 ± 12.05	61.27 ± 17.65	62.45 ± 13.32	59.64 ± 17.82	59.64 ± 17.30	56.09 ± 5.40
Pittsburg-bridgesmaterial	84.00 ± 7.75	84.00 ± 7.50	87.73 ± 8.07	83.73 ± 11.42	85.91 ± 6.39	85.00 ± 6.24	84.00 ± 9.64	83.18 ± 11.03	79.36 ± 10.14	86.82 ± 4.74
Planning	72.02 ± 2.57	68.13 ± 6.28	71.46 ± 1.60	65.94 ± 9.32	71.46 ± 1.60	70.32 ± 5.96	65.91 ± 6.93	61.49 ± 7.03	58.71 ± 9.93	62.05 ± 8.60
Plant-margin	63.13 ± 4.31	84.69 ± 2.59	48.06 ± 4.50	74.00 ± 2.78	82.81 ± 2.27	84.75 ± 2.80	85.13 ± 2.82	83.06 ± 2.23	83.63 ± 3.24	7.69 ± 2.17
Plant-shape	49.81 ± 4.42	53.19 ± 2.88	46.13 ± 3.40	64.69 ± 3.35	48.94 ± 1.62	56.75 ± 3.13	60.06 ± 3.35	64.75 ± 3.79	65.00 ± 3.35	8.56 ± 1.62
Plant-texture	62.85 ± 2.93	74.42 ± 3.13	51.97 ± 3.48	80.11 ± 2.84	83.30 ± 2.50	85.55 ± 2.29	86.06 ± 2.35	83.80 ± 1.74	83.11 ± 1.91	3.44 ± 0.90
Poker-hand-training-true	49.95 ± 0.02	49.95 ± 0.02	55.91 ± 2.04	47.03 ± 0.86	58.71 ± 1.00	57.57 ± 0.93	56.45 ± 1.11	53.97 ± 0.61	51.90 ± 2.03	49.95 ± 0.02
Post-operative	71.11 ± 5.74	65.56 ± 6.31	71.11 ± 5.74	61.11 ± 10.80	71.11 ± 5.74	68.89 ± 7.03	66.67 ± 9.07	56.67 ± 13.30	56.67 ± 16.10	68.89 ± 7.03
Primary-tumor	26.06 ± 2.12	43.03 ± 10.18	43.33 ± 9.04	35.15 ± 8.35	46.67 ± 10.90	44.85 ± 10.57	43.94 ± 11.97	40.91 ± 7.99	41.21 ± 8.48	27.88 ± 2.78
Qsarbioconcentration	74.84 ± 0.63	73.69 ± 1.80	73.29 ± 3.81	61.62 ± 5.75	74.84 ± 0.70	73.30 ± 1.50	70.60 ± 2.89	70.99 ± 4.07	71.63 ± 3.05	72.79 ± 3.27
Qsarbiodegradation	69.95 ± 2.16	75.83 ± 3.23	83.51 ± 3.46	83.60 ± 2.24	85.88 ± 2.62	86.25 ± 2.17	85.59 ± 2.49	86.92 ± 2.30	87.21 ± 2.46	76.69 ± 3.95
Qualitative-bankruptcy	99.20 ± 2.53	99.20 ± 2.53	98.00 ± 2.83	100.00 ± 0.00	98.80 ± 2.70	98.80 ± 2.70	99.60 ± 1.26	99.20 ± 2.53	99.20 ± 2.53	98.40 ± 2.80
Ringnorm	79.14 ± 1.43	98.64 ± 0.47	91.61 ± 0.27	75.16 ± 1.58	98.59 ± 0.41	98.47 ± 0.41	98.35 ± 0.48	91.95 ± 1.47	91.70 ± 0.86	64.43 ± 0.97
Risk-factors-cervical-cancer	93.59 ± 0.60	88.69 ± 1.91	95.10 ± 1.97	94.40 ± 2.06	93.59 ± 0.60	93.47 ± 0.80	93.94 ± 1.62	94.76 ± 2.00	94.52 ± 2.27	96.15 ± 1.91
Robotnavigation	91.81 ± 1.00	52.51 ± 2.01	99.60 ± 0.23	88.25 ± 1.22	90.03 ± 1.14	91.18 ± 0.98	91.99 ± 0.77	88.21 ± 1.86	88.20 ± 2.44	75.37 ± 1.90
Sapfile	48.85 ± 11.47	54.18 ± 10.31	45.71 ± 14.95	41.98 ± 8.97	48.85 ± 8.89	49.67 ± 6.83	49.67 ± 8.54	48.08 ± 9.46	47.31 ± 12.30	41.92 ± 12.36
Sat	85.10 ± 0.87	79.60 ± 1.23	86.03 ± 0.98	90.55 ± 0.79	26.14 ± 0.53	27.32 ± 0.47	27.32 ± 0.47	89.71 ± 1.60	88.98 ± 1.11	59.75 ± 1.30
Satelite	85.07 ± 0.82	79.56 ± 1.10	86.34 ± 0.65	90.58 ± 1.11	26.12 ± 0.57	27.40 ± 0.71	27.40 ± 0.71	89.82 ± 0.66	89.26 ± 0.87	59.97 ± 1.65
Scadi	58.57 ± 8.11	82.86 ± 9.04	80.00 ± 13.80	80.00 ± 13.80	75.71 ± 9.64	78.57 ± 13.88	81.43 ± 15.13	80.00 ± 9.99	80.00 ± 9.99	58.57 ± 10.54
Schillingdata	86.86 ± 0.24	93.86 ± 0.74	86.74 ± 0.15	88.45 ± 1.00	86.74 ± 0.15	89.00 ± 1.03	91.63 ± 0.66	92.67 ± 2.08	91.01 ± 3.38	86.58 ± 0.44
Seeds	86.67 ± 6.66	91.43 ± 4.38	91.90 ± 5.96	94.29 ± 2.01	93.81 ± 3.92	93.81 ± 3.92	93.81 ± 3.92	95.24 ± 3.17	96.19 ± 3.01	82.38 ± 10.05
Segment	90.30 ± 2.26	80.22 ± 1.89	96.93 ± 1.09	97.14 ± 0.62	65.37 ± 2.88	67.01 ± 2.40	66.97 ± 2.47	96.06 ± 1.20	96.28 ± 0.89	63.85 ± 2.78
Seismic-bumps	93.42 ± 0.01	86.73 ± 1.89	93.34 ± 0.24	89.39 ± 1.46	93.42 ± 0.01	93.42 ± 0.01	93.42 ± 0.01	92.45 ± 0.93	92.30 ± 0.92	92.96 ± 0.55
Semeion	78.02 ± 3.36	85.94 ± 2.72	75.83 ± 2.39	91.46 ± 1.92	95.60 ± 1.07	96.17 ± 0.69	96.11 ± 0.72	92.65 ± 1.57	93.09 ± 1.66	19.59 ± 0.47
Setapprocesst1	68.33 ± 8.70	58.57 ± 22.11	62.86 ± 20.40	65.24 ± 10.72	63.81 ± 8.38	68.57 ± 10.64	66.90 ± 9.29	64.76 ± 20.48	65.00 ± 19.92	72.14 ± 23.19
Setapprocesst10	66.43 ± 5.51	44.64 ± 15.99	58.21 ± 14.12	60.54 ± 16.51	66.43 ± 5.51	66.43 ± 5.51	66.43 ± 5.51	51.25 ± 27.07	51.25 ± 21.95	62.14 ± 8.07
Setapprocesst11	66.43 ± 5.51	52.50 ± 24.67	65.71 ± 18.55	58.04 ± 16.28	66.43 ± 5.51	66.43 ± 5.51	66.43 ± 5.51	61.43 ± 15.71	64.11 ± 20.11	56.96 ± 10.60
Setapprocesst2	67.86 ± 8.16	66.79 ± 15.27	72.14 ± 19.71	62.68 ± 21.57	66.43 ± 5.51	66.43 ± 5.51	66.43 ± 5.51	62.68 ± 20.16	65.18 ± 20.81	81.25 ± 16.71
Setapprocesst3	66.43 ± 5.51	65.18 ± 12.69	60.54 ± 15.76	66.25 ± 13.40	66.43 ± 5.51	66.43 ± 5.51	66.43 ± 5.51	66.25 ± 13.40	66.25 ± 9.43	53.04 ± 16.45
Setapprocesst4	71.43 ± 6.64	47.62 ± 26.15	55.24 ± 15.22	60.24 ± 18.58	71.43 ± 6.64	71.43 ± 6.64	71.43 ± 6.64	55.00 ± 21.89	56.90 ± 17.37	72.62 ± 11.68
Setapprocesst5	69.29 ± 7.28	45.89 ± 16.52	53.39 ± 18.00	59.64 ± 17.60	65.18 ± 7.54	63.93 ± 8.94	63.93 ± 8.94	59.82 ± 13.58	62.50 ± 14.68	65.71 ± 24.88
Setapprocesst6	66.43 ± 5.51	67.50 ± 16.68	64.82 ± 13.15	64.29 ± 14.51	66.43 ± 5.51	66.43 ± 5.51	66.43 ± 5.51	62.68 ± 17.46	62.68 ± 14.64	60.18 ± 20.15
Setapprocesst7	67.86 ± 8.16	74.82 ± 14.86	60.71 ± 19.94	64.11 ± 15.09	66.43 ± 5.51	66.43 ± 5.51	66.43 ± 5.51	70.54 ± 21.15	71.96 ± 18.29	52.86 ± 15.18
Setapprocesst8	66.43 ± 5.51	68.93 ± 14.02	70.18 ± 15.13	63.21 ± 15.59	66.43 ± 5.51	66.43 ± 5.51	66.43 ± 5.51	66.61 ± 16.37	65.36 ± 18.16	49.46 ± 11.45
Setapprocesst9	66.43 ± 5.51	64.64 ± 16.62	69.29 ± 16.49	58.39 ± 9.93	66.43 ± 5.51	66.43 ± 5.51	66.43 ± 5.51	70.18 ± 20.26	67.68 ± 17.22	50.71 ± 16.54
Shillbiddingdataset	90.95 ± 0.43	96.71 ± 0.43	99.62 ± 0.15	99.22 ± 0.28	99.54 ± 0.17	99.56 ± 0.21	99.65 ± 0.19	99.68 ± 0.15	99.72 ± 0.19	97.28 ± 0.54
Shuttle-landing-control	95.00 ± 15.81	80.00 ± 34.96	95.00 ± 15.81	95.00 ± 15.81	95.00 ± 15.81	95.00 ± 15.81	95.00 ± 15.81	85.00 ± 33.75	80.00 ± 34.96	85.00 ± 33.75
Somervillehappinesssurvey2015	57.33 ± 12.50	59.38 ± 9.99	64.29 ± 17.09	58.95 ± 12.80	60.05 ± 10.86	58.71 ± 10.31	58.76 ± 11.69	58.86 ± 9.38	59.57 ± 11.65	65.67 ± 15.87
Sonar	76.55 ± 7.06	67.88 ± 9.29	71.17 ± 7.10	86.57 ± 7.01	65.90 ± 5.43	70.26 ± 9.01	73.60 ± 9.54	82.29 ± 10.70	81.76 ± 9.68	62.50 ± 11.07
Soybean	69.10 ± 6.56	91.86 ± 5.60	87.26 ± 5.16	87.62 ± 4.79	90.22 ± 6.00	92.17 ± 4.45	91.53 ± 4.69	89.88 ± 6.85	90.84 ± 6.47	30.63 ± 2.45
Spambase	77.42 ± 1.19	79.11 ± 1.78	92.96 ± 1.19	90.78 ± 1.10	93.39 ± 0.93	93.50 ± 1.10	93.41 ± 0.92	91.20 ± 1.27	89.11 ± 6.86	78.35 ± 1.99
Speaker-accent	51.36 ± 2.52	58.07 ± 10.22	69.92 ± 9.58	80.56 ± 8.92	58.36 ± 4.00	62.31 ± 4.31	62.31 ± 4.31	79.64 ± 8.20	75.40 ± 7.29	50.45 ± 4.64
Spect	70.89 ± 8.34	72.14 ± 17.46	70.89 ± 13.18	55.71 ± 15.72	64.64 ± 11.19	64.46 ± 11.74	63.21 ± 9.46	55.71 ± 17.79	56.96 ± 14.57	72.14 ± 9.82
Spectf	68.75 ± 12.15	75.00 ± 11.79	73.75 ± 12.43	67.50 ± 18.82	67.50 ± 6.45	67.50 ± 6.45	76.25 ± 13.76	71.25 ± 14.49	72.50 ± 15.37	71.25 ± 8.44
Statlog-australian-credit	67.68 ± 0.70	59.28 ± 5.53	66.96 ± 7.13	55.80 ± 3.29	67.54 ± 1.83	64.35 ± 6.38	63.91 ± 5.61	62.75 ± 5.59	61.01 ± 4.50	65.22 ± 6.34
Statlog-german-credit	70.00 ± 0.00	75.60 ± 5.21	73.90 ± 4.68	67.80 ± 3.94	76.10 ± 4.12	77.30 ± 3.74	77.30 ± 4.42	70.90 ± 3.93	72.20 ± 3.36	70.90 ± 2.28
Statlog-heart	83.70 ± 7.45	83.33 ± 7.25	76.30 ± 9.75	75.19 ± 8.56	82.96 ± 5.30	80.00 ± 8.41	79.63 ± 9.60	78.15 ± 7.08	80.00 ± 8.76	71.11 ± 6.25
Statlog-image	90.22 ± 2.15	80.09 ± 2.02	96.93 ± 1.09	97.14 ± 0.62	94.29 ± 0.88	95.15 ± 0.95	95.45 ± 0.90	96.10 ± 0.91	96.15 ± 1.05	63.68 ± 2.46
Statlog-landsat	85.95 ± 1.49	79.55 ± 2.20	86.13 ± 1.81	90.15 ± 1.70	89.47 ± 1.59	90.19 ± 1.38	90.26 ± 1.61	89.00 ± 1.26	88.88 ± 1.74	60.45 ± 1.68
Statlog-shuttle	84.19 ± 0.43	91.64 ± 0.89	99.96 ± 0.03	99.94 ± 0.04	99.75 ± 0.05	99.80 ± 0.05	99.80 ± 0.04	99.69 ± 0.04	99.71 ± 0.07	94.69 ± 0.30
Statlog-vehicle	66.55 ± 2.71	44.80 ± 3.40	72.23 ± 5.93	69.86 ± 4.47	76.72 ± 3.88	80.26 ± 2.60	80.50 ± 2.36	82.28 ± 3.15	80.75 ± 4.62	51.19 ± 5.09
Steel-plates	61.62 ± 2.95	60.28 ± 3.84	76.04 ± 3.30	71.97 ± 2.73	75.53 ± 2.77	76.97 ± 2.27	76.87 ± 2.92	72.70 ± 2.86	72.65 ± 3.17	48.17 ± 3.88
Synthetic-control	89.00 ± 2.74	94.67 ± 3.50	91.67 ± 3.51	96.50 ± 2.14	99.33 ± 0.86	99.50 ± 0.81	99.50 ± 0.81	99.33 ± 0.86	99.33 ± 0.86	57.00 ± 3.83
Teaching	50.25 ± 11.28	54.21 ± 8.37	59.58 ± 11.57	62.29 ± 13.54	56.17 ± 14.70	61.50 ± 12.68	62.17 ± 12.37	54.29 ± 11.11	52.29 ± 9.41	46.38 ± 10.93
Thoraricsurgery	85.11 ± 0.00	78.51 ± 9.27	84.47 ± 1.03	77.23 ± 5.94	85.11 ± 0.00	84.47 ± 1.03	84.47 ± 1.03	79.15 ± 4.23	79.79 ± 5.42	83.40 ± 1.68
Thyroid	85.19 ± 7.42	96.73 ± 3.84	92.06 ± 6.32	97.21 ± 2.40	75.87 ± 5.01	77.23 ± 4.51	77.23 ± 4.51	96.75 ± 3.10	96.75 ± 3.10	90.69 ± 8.54
Thyroid-train	92.47 ± 0.13	95.63 ± 1.10	99.71 ± 0.32	92.13 ± 1.32	95.10 ± 0.68	95.68 ± 0.60	96.26 ± 0.63	96.58 ± 1.05	96.00 ± 1.30	96.47 ± 0.78
Tic-tac-toe	65.34 ± 0.43	69.62 ± 3.56	93.84 ± 2.44	100.00 ± 0.00	98.64 ± 1.40	99.27 ± 1.00	99.69 ± 0.71	97.81 ± 1.04	97.91 ± 1.21	69.93 ± 2.39
Titanic	78.05 ± 2.46	75.37 ± 2.86	79.05 ± 1.78	79.05 ± 1.78	78.46 ± 2.19	78.92 ± 2.01	78.92 ± 2.01	78.15 ± 2.26	77.42 ± 2.19	77.60 ± 2.02
Trains	70.00 ± 48.30	60.00 ± 51.64	70.00 ± 48.30	60.00 ± 51.64	60.00 ± 51.64	70.00 ± 48.30	70.00 ± 48.30	80.00 ± 42.16	80.00 ± 42.16	0.00 ± 0.00
Transfusion	76.88 ± 1.34	75.40 ± 3.44	77.81 ± 3.77	70.45 ± 4.45	75.27 ± 2.89	72.99 ± 3.06	71.92 ± 3.72	78.21 ± 2.47	78.48 ± 2.80	76.34 ± 1.66
Trial	94.08 ± 2.90	90.34 ± 3.48	100.00 ± 0.00	99.74 ± 0.82	99.10 ± 0.87	99.36 ± 0.68	99.36 ± 0.68	99.23 ± 0.90	99.36 ± 0.91	100.00 ± 0.00
Turkiye-student-evaluation	61.87 ± 0.05	86.99 ± 1.52	99.97 ± 0.07	90.41 ± 1.58	95.50 ± 0.52	96.27 ± 0.59	96.27 ± 0.59	100.00 ± 0.00	100.00 ± 0.00	99.97 ± 0.07
Unbalanced	98.60 ± 0.49	90.77 ± 4.44	98.60 ± 0.49	97.66 ± 1.57	98.60 ± 0.49	98.37 ± 0.60	98.13 ± 0.81	98.13 ± 1.12	97.90 ± 1.08	98.60 ± 0.49
Urbanlandcover	80.18 ± 10.26	78.49 ± 9.22	79.08 ± 7.80	76.65 ± 11.07	17.28 ± 1.99	17.28 ± 1.99	17.28 ± 1.99	77.21 ± 12.43	77.21 ± 12.43	49.23 ± 14.54
Userknowledgemodeling	63.91 ± 7.90	89.34 ± 5.79	94.02 ± 3.41	80.58 ± 10.14	77.74 ± 6.42	85.29 ± 4.31	85.69 ± 3.93	91.29 ± 3.59	93.25 ± 4.58	85.31 ± 7.80
Vehicle	69.27 ± 4.29	44.80 ± 4.72	72.47 ± 5.87	69.86 ± 4.47	30.50 ± 2.54	31.32 ± 3.07	31.32 ± 3.07	*81.70 ± 3.82*	81.21 ± 4.43	51.90 ± 5.04
Vertebral-column-2classes	74.52 ± 8.93	77.74 ± 7.04	81.61 ± 7.91	81.94 ± 5.09	*85.48 ± 5.32*	84.52 ± 6.23	84.84 ± 5.70	84.52 ± 6.77	84.19 ± 6.17	73.87 ± 7.67
Vertebral-column-3classes	70.00 ± 10.32	83.23 ± 5.85	82.26 ± 6.13	78.71 ± 4.86	83.87 ± 5.89	84.84 ± 6.98	84.19 ± 6.71	*85.16 ± 4.86*	84.52 ± 6.04	75.16 ± 6.98
Veteran	71.48 ± 2.70	72.09 ± 8.96	72.20 ± 6.80	60.66 ± 8.86	70.77 ± 1.06	70.77 ± 1.06	70.77 ± 1.06	60.60 ± 14.50	67.14 ± 15.64	*73.57 ± 7.61*
Vowel	62.42 ± 3.43	63.74 ± 4.43	81.52 ± 4.72	*99.29 ± 0.83*	85.05 ± 2.60	91.11 ± 2.01	92.93 ± 2.08	92.83 ± 3.45	93.54 ± 2.39	32.02 ± 4.21
Wall-following	91.86 ± 1.22	52.47 ± 1.38	*99.65 ± 0.25*	88.18 ± 1.21	89.00 ± 1.41	90.60 ± 1.58	91.35 ± 1.30	87.48 ± 1.40	87.99 ± 1.95	75.64 ± 1.43
Waveform-noise	67.18 ± 2.20	80.00 ± 1.93	75.16 ± 1.37	73.62 ± 1.27	*86.14 ± 1.51*	85.38 ± 1.28	84.90 ± 1.41	83.54 ± 1.64	83.08 ± 1.43	53.72 ± 2.75
Waveform	70.94 ± 1.45	81.02 ± 1.38	75.88 ± 1.31	76.90 ± 2.01	*86.42 ± 1.36*	85.66 ± 1.38	85.46 ± 1.43	83.84 ± 1.15	83.16 ± 1.28	52.54 ± 1.64
Wbc	96.92 ± 2.02	97.51 ± 1.39	92.98 ± 2.02	95.76 ± 2.52	97.66 ± 1.58	97.66 ± 1.58	*97.80 ± 1.73*	95.76 ± 1.59	96.19 ± 1.85	91.06 ± 2.91
Wdbc	93.68 ± 3.71	92.98 ± 4.30	93.33 ± 3.94	95.96 ± 1.87	62.74 ± 0.73	62.74 ± 0.73	62.74 ± 0.73	*96.66 ± 1.76*	95.96 ± 2.35	89.81 ± 3.17
Weathernominal	65.00 ± 41.16	60.00 ± 39.44	55.00 ± 43.78	65.00 ± 41.16	70.00 ± 34.96	60.00 ± 39.44	*75.00 ± 35.36*	*75.00 ± 35.36*	*75.00 ± 35.36*	45.00 ± 43.78
Weathernumeric	55.00 ± 43.78	70.00 ± 34.96	70.00 ± 42.16	*80.00 ± 34.96*	70.00 ± 34.96	50.00 ± 47.14	50.00 ± 47.14	75.00 ± 42.49	75.00 ± 42.49	45.00 ± 43.78
Website-phishingdata	82.41 ± 2.24	84.11 ± 1.90	*90.76 ± 2.08*	88.32 ± 2.77	85.66 ± 1.27	86.18 ± 1.20	87.65 ± 2.05	88.77 ± 1.94	89.80 ± 2.46	81.74 ± 1.63
Wholesalecustomersdata	89.55 ± 2.20	89.09 ± 3.83	90.23 ± 3.04	87.95 ± 4.80	67.73 ± 0.96	67.73 ± 0.96	67.73 ± 0.96	*91.59 ± 3.40*	90.91 ± 4.01	90.45 ± 3.68
Wifi-localization	92.60 ± 1.68	98.20 ± 0.71	97.15 ± 0.63	*98.30 ± 0.89*	79.05 ± 2.49	80.45 ± 2.58	80.45 ± 2.58	97.90 ± 0.39	97.95 ± 0.69	79.00 ± 4.45
Wilt	94.61 ± 0.07	89.17 ± 1.82	*98.20 ± 0.46*	94.88 ± 0.58	94.77 ± 0.14	94.77 ± 0.14	94.77 ± 0.14	97.85 ± 0.87	98.12 ± 0.58	94.46 ± 0.21
Wine-quality-red	61.98 ± 3.57	55.10 ± 4.59	61.16 ± 2.98	*64.79 ± 2.73*	62.79 ± 2.48	63.17 ± 2.68	63.29 ± 2.70	60.48 ± 3.81	60.60 ± 3.07	54.66 ± 3.11
Wine-quality-white	57.17 ± 1.97	44.10 ± 1.20	58.35 ± 2.80	*65.40 ± 2.50*	57.00 ± 2.28	57.86 ± 2.47	58.11 ± 2.64	55.25 ± 2.32	54.61 ± 1.46	45.75 ± 2.15
Wine	96.11 ± 5.89	96.63 ± 5.38	93.86 ± 5.52	94.97 ± 4.11	*98.89 ± 2.34*	98.30 ± 2.74	98.30 ± 2.74	97.19 ± 3.96	97.19 ± 3.96	76.96 ± 9.24
Yamilnaduelectricty	19.88 ± 1.29	6.65 ± 0.37	14.06 ± 1.09	*21.21 ± 1.31*	6.65 ± 0.55	6.82 ± 0.56	6.70 ± 0.61	6.40 ± 0.72	6.40 ± 0.69	7.45 ± 1.12
Yeast	46.83 ± 2.58	57.81 ± 2.65	55.99 ± 4.85	52.29 ± 2.39	60.24 ± 4.08	60.31 ± 4.24	*60.44 ± 4.50*	59.43 ± 3.49	58.82 ± 3.39	40.02 ± 3.39
Youtobe-kabita-preprocessing	33.18 ± 2.32	26.61 ± 1.02	34.18 ± 1.77	31.94 ± 1.55	*37.33 ± 2.37*	36.22 ± 2.19	35.53 ± 2.10	33.49 ± 1.29	33.67 ± 2.04	32.37 ± 2.01
Youtobe-nisha-preprocessing	37.02 ± 1.82	33.14 ± 1.90	38.51 ± 1.25	35.49 ± 1.84	*40.45 ± 2.13*	39.22 ± 2.10	38.82 ± 2.22	37.49 ± 1.95	36.22 ± 2.37	31.22 ± 1.32
Z-alizadehsani	71.29 ± 1.47	79.87 ± 6.59	79.25 ± 8.32	78.26 ± 7.02	71.29 ± 1.47	71.29 ± 1.47	71.29 ± 1.47	*82.25 ± 7.86*	81.59 ± 7.34	70.66 ± 5.28
Zoo	91.18 ± 8.52	*96.18 ± 6.54*	92.18 ± 8.94	*96.18 ± 6.54*	94.18 ± 8.11	94.18 ± 8.11	94.18 ± 8.11	95.18 ± 6.65	95.18 ± 6.65	73.27 ± 10.54
Average acc (rank)	73.57 (5)	72.05 (8)	78.53 (1)	76.87 (4)	71.96 (9)	72.66 (7)	73.01 (6)	77.95 (2)	77.84 (3)	67.16 (10)
Average acc std (rank)	4.85 (4)	6.41 (10)	5.86 (6)	6.07 (7)	4.20 (1)	4.52 (2)	4.58 (3)	6.10 (8)	6.18 (9)	5.63 (5)

**Table 7:** The detailed experimental results on kappa statistic and standard deviation.

Dataset	RN	NB	J48	KNN	SVM1	SVM2	SVM3	ANN1	ANN2	OneR
Abalone	0.38 ± 0.04	0.36 ± 0.03	0.41 ± 0.03	0.37 ± 0.04	0.50 ± 0.04	0.50 ± 0.04	0.50 ± 0.03	0.48 ± 0.05	0.48 ± 0.06	0.41 ± 0.03
Absenteeism-at-work	0.98 ± 0.03	0.71 ± 0.08	0.99 ± 0.01	0.67 ± 0.06	0.67 ± 0.07	0.67 ± 0.07	0.67 ± 0.07	0.95 ± 0.02	0.94 ± 0.05	0.90 ± 0.04
Acute-inflammation	1.00 ± 0.00	1.00 ± 0.00	1.00 ± 0.00	1.00 ± 0.00	1.00 ± 0.00	1.00 ± 0.00	1.00 ± 0.00	1.00 ± 0.00	1.00 ± 0.00	0.58 ± 0.16
Acute-nephritis	1.00 ± 0.00	0.89 ± 0.15	1.00 ± 0.00	1.00 ± 0.00	1.00 ± 0.00	1.00 ± 0.00	1.00 ± 0.00	1.00 ± 0.00	1.00 ± 0.00	0.84 ± 0.15
Adult	0.00 ± 0.00	0.34 ± 0.09	0.47 ± 0.10	0.41 ± 0.10	0.47 ± 0.13	0.49 ± 0.14	0.50 ± 0.14	0.44 ± 0.08	0.42 ± 0.08	0.26 ± 0.07
Aggregation	0.81 ± 0.07	1.00 ± 0.01	1.00 ± 0.01	1.00 ± 0.01	1.00 ± 0.01	1.00 ± 0.01	1.00 ± 0.01	0.99 ± 0.01	0.99 ± 0.01	0.47 ± 0.04
Algerianforest	0.78 ± 0.17	0.89 ± 0.10	0.91 ± 0.09	0.76 ± 0.14	0.06 ± 0.05	0.14 ± 0.09	0.14 ± 0.09	0.92 ± 0.08	0.93 ± 0.09	0.94 ± 0.10
Annealing	0.00 ± 0.00	0.50 ± 0.05	0.91 ± 0.05	0.74 ± 0.06	0.67 ± 0.10	0.76 ± 0.08	0.78 ± 0.06	0.78 ± 0.08	0.77 ± 0.08	0.44 ± 0.00
Arrhythmia	0.00 ± 0.00	0.43 ± 0.11	0.46 ± 0.10	0.24 ± 0.05	0.25 ± 0.05	0.35 ± 0.08	0.35 ± 0.08	0.46 ± 0.09	0.30 ± 0.12	0.21 ± 0.06
Au1-1000	0.00 ± 0.00	0.04 ± 0.06	0.35 ± 0.12	0.15 ± 0.11	0.00 ± 0.00	0.00 ± 0.00	0.02 ± 0.04	0.17 ± 0.12	0.22 ± 0.11	0.00 ± 0.00
Au4-2500	0.30 ± 0.11	0.22 ± 0.08	0.51 ± 0.10	0.35 ± 0.08	0.29 ± 0.05	0.29 ± 0.05	0.29 ± 0.05	0.37 ± 0.08	0.37 ± 0.09	0.15 ± 0.08
Au6-1000	0.00 ± 0.02	0.02 ± 0.04	0.06 ± 0.05	-0.01 ± 0.04	0.00 ± 0.00	0.00 ± 0.00	0.00 ± 0.00	0.00 ± 0.04	-0.01 ± 0.05	0.08 ± 0.03
Au6-250-drift-au6-cd1-500	0.02 ± 0.03	0.04 ± 0.05	0.05 ± 0.05	0.01 ± 0.05	0.00 ± 0.00	0.00 ± 0.00	0.00 ± 0.00	-0.00 ± 0.03	0.01 ± 0.04	0.08 ± 0.04
Au6-cd1-400	0.03 ± 0.09	0.08 ± 0.07	0.24 ± 0.08	0.01 ± 0.09	0.00 ± 0.00	0.00 ± 0.00	0.00 ± 0.00	0.04 ± 0.08	0.03 ± 0.08	0.11 ± 0.08
Au7-300-drift-au7-cpd1-800	0.05 ± 0.05	0.15 ± 0.05	0.21 ± 0.06	0.14 ± 0.05	0.01 ± 0.02	0.01 ± 0.03	0.01 ± 0.03	0.15 ± 0.05	0.17 ± 0.06	0.03 ± 0.04
Au7-700	0.11 ± 0.10	0.14 ± 0.08	0.28 ± 0.06	0.03 ± 0.08	0.04 ± 0.04	0.03 ± 0.04	0.03 ± 0.04	0.11 ± 0.08	0.15 ± 0.05	0.17 ± 0.06
Au7-cpd1-500	0.03 ± 0.04	0.10 ± 0.08	0.37 ± 0.06	0.17 ± 0.08	-0.00 ± 0.01	0.01 ± 0.03	0.01 ± 0.03	0.18 ± 0.08	0.16 ± 0.07	0.12 ± 0.07
Audiology-std	0.03 ± 0.04	0.69 ± 0.12	0.72 ± 0.09	0.70 ± 0.14	0.55 ± 0.10	0.61 ± 0.13	0.65 ± 0.10	0.81 ± 0.08	0.80 ± 0.05	0.30 ± 0.02
Audit-risk	0.80 ± 0.09	0.88 ± 0.04	1.00 ± 0.01	0.95 ± 0.03	0.97 ± 0.03	0.97 ± 0.03	0.98 ± 0.03	0.93 ± 0.06	0.93 ± 0.06	1.00 ± 0.00
Autism-adolescent-data	0.55 ± 0.20	0.96 ± 0.09	1.00 ± 0.00	0.79 ± 0.15	0.92 ± 0.11	0.89 ± 0.11	0.92 ± 0.11	0.78 ± 0.22	0.78 ± 0.22	1.00 ± 0.00
Autism-adult-data	0.49 ± 0.13	0.93 ± 0.06	1.00 ± 0.00	0.87 ± 0.09	0.98 ± 0.03	0.99 ± 0.02	0.99 ± 0.03	1.00 ± 0.00	1.00 ± 0.00	1.00 ± 0.00
Autism-child-data	0.83 ± 0.10	0.98 ± 0.03	1.00 ± 0.00	0.77 ± 0.07	0.99 ± 0.02	1.00 ± 0.00	1.00 ± 0.00	0.99 ± 0.02	0.99 ± 0.02	1.00 ± 0.00
Autos	0.62 ± 0.13	0.45 ± 0.16	0.78 ± 0.10	0.70 ± 0.13	0.03 ± 0.06	0.05 ± 0.07	0.05 ± 0.07	0.70 ± 0.08	0.70 ± 0.06	0.49 ± 0.13
Avila	0.96 ± 0.01	0.18 ± 0.02	0.92 ± 0.02	0.68 ± 0.03	0.56 ± 0.03	0.59 ± 0.03	0.61 ± 0.03	0.48 ± 0.03	0.49 ± 0.04	0.62 ± 0.03
Balance-scale	0.62 ± 0.05	0.82 ± 0.03	0.58 ± 0.06	0.67 ± 0.07	0.82 ± 0.03	0.87 ± 0.02	0.89 ± 0.02	0.84 ± 0.06	0.84 ± 0.06	0.19 ± 0.08
Balloons	0.50 ± 0.53	0.30 ± 0.48	0.40 ± 0.52	0.60 ± 0.52	0.50 ± 0.53	0.70 ± 0.48	0.70 ± 0.48	0.50 ± 0.53	0.50 ± 0.53	0.10 ± 0.57
Bank	0.00 ± 0.00	0.31 ± 0.06	0.36 ± 0.07	0.22 ± 0.06	0.27 ± 0.06	0.31 ± 0.05	0.34 ± 0.08	0.35 ± 0.08	0.37 ± 0.08	0.20 ± 0.07
Blood	0.05 ± 0.07	0.12 ± 0.08	0.34 ± 0.12	0.11 ± 0.11	0.20 ± 0.10	0.26 ± 0.05	0.26 ± 0.05	0.28 ± 0.09	0.30 ± 0.10	0.06 ± 0.08
Breast-cancer-wisc-diag	0.86 ± 0.08	0.85 ± 0.09	0.86 ± 0.08	0.91 ± 0.04	0.95 ± 0.05	0.95 ± 0.04	0.96 ± 0.02	0.92 ± 0.04	0.92 ± 0.04	0.77 ± 0.09
Breast-cancer-wisc-prog	0.00 ± 0.00	0.21 ± 0.29	0.29 ± 0.18	0.23 ± 0.29	0.20 ± 0.27	0.24 ± 0.34	0.24 ± 0.33	0.26 ± 0.26	0.27 ± 0.29	0.01 ± 0.21
Breast-cancer-wisc	0.91 ± 0.05	0.91 ± 0.04	0.86 ± 0.07	0.90 ± 0.07	0.94 ± 0.04	0.93 ± 0.04	0.93 ± 0.04	0.89 ± 0.08	0.90 ± 0.05	0.83 ± 0.11
Breast-cancer	0.01 ± 0.05	0.23 ± 0.18	0.21 ± 0.12	0.27 ± 0.18	0.23 ± 0.15	0.24 ± 0.16	0.22 ± 0.14	0.28 ± 0.18	0.18 ± 0.10	0.16 ± 0.13
Breast-tissue	0.61 ± 0.16	0.59 ± 0.18	0.59 ± 0.19	0.65 ± 0.17	0.49 ± 0.17	0.61 ± 0.20	0.63 ± 0.20	0.56 ± 0.11	0.55 ± 0.19	0.44 ± 0.12
Bupa	0.29 ± 0.11	0.19 ± 0.15	0.22 ± 0.18	0.21 ± 0.10	0.03 ± 0.05	0.03 ± 0.05	0.03 ± 0.05	0.30 ± 0.15	0.34 ± 0.14	0.05 ± 0.16
Caesarian	0.44 ± 0.31	0.31 ± 0.38	0.17 ± 0.35	0.25 ± 0.33	-0.02 ± 0.09	0.20 ± 0.25	0.16 ± 0.29	0.11 ± 0.41	0.11 ± 0.44	-0.00 ± 0.37
Car	0.00 ± 0.00	0.63 ± 0.09	0.95 ± 0.02	0.93 ± 0.03	0.94 ± 0.02	0.96 ± 0.02	0.96 ± 0.02	0.85 ± 0.03	0.86 ± 0.04	0.00 ± 0.00
Cardiotocography-10classes	0.58 ± 0.03	0.66 ± 0.03	0.80 ± 0.03	0.74 ± 0.03	0.77 ± 0.03	0.79 ± 0.03	0.80 ± 0.03	0.80 ± 0.04	0.78 ± 0.04	0.34 ± 0.03
Cardiotocography-3classes	0.00 ± 0.01	0.60 ± 0.06	0.81 ± 0.07	0.78 ± 0.05	0.75 ± 0.05	0.78 ± 0.06	0.78 ± 0.05	0.78 ± 0.03	0.78 ± 0.07	0.38 ± 0.12
Cervical-cancer	0.24 ± 0.30	0.76 ± 0.23	0.64 ± 0.33	0.71 ± 0.28	0.00 ± 0.00	0.12 ± 0.25	0.12 ± 0.25	0.82 ± 0.26	0.82 ± 0.26	0.37 ± 0.28
Chemicalcomposionofceramic	1.00 ± 0.00	1.00 ± 0.00	0.97 ± 0.08	1.00 ± 0.00	0.00 ± 0.00	0.00 ± 0.00	0.00 ± 0.00	1.00 ± 0.00	1.00 ± 0.00	1.00 ± 0.00
Chess-krvk	0.15 ± 0.02	0.21 ± 0.02	0.46 ± 0.03	0.44 ± 0.04	0.39 ± 0.03	0.42 ± 0.03	0.43 ± 0.03	0.38 ± 0.03	0.36 ± 0.02	0.13 ± 0.02
Chess-krvkp	0.53 ± 0.03	0.71 ± 0.04	0.99 ± 0.01	0.91 ± 0.03	0.97 ± 0.02	0.98 ± 0.01	0.98 ± 0.01	0.98 ± 0.01	0.98 ± 0.01	0.32 ± 0.03
Congressional-voting	-0.00 ± 0.01	-0.02 ± 0.16	-0.03 ± 0.05	0.02 ± 0.13	0.04 ± 0.10	0.05 ± 0.12	0.04 ± 0.13	0.06 ± 0.17	0.04 ± 0.17	0.06 ± 0.07
Conn-bench-sonar-mines-rocks	0.53 ± 0.15	0.38 ± 0.18	0.42 ± 0.14	0.73 ± 0.14	0.68 ± 0.14	0.74 ± 0.16	0.77 ± 0.16	0.61 ± 0.22	0.64 ± 0.17	0.27 ± 0.21
Conn-bench-vowel-deterding	0.73 ± 0.03	0.62 ± 0.06	0.77 ± 0.07	0.99 ± 0.01	0.94 ± 0.02	0.97 ± 0.02	0.98 ± 0.02	0.83 ± 0.03	0.81 ± 0.04	0.29 ± 0.05
Connect-4	0.00 ± 0.00	0.13 ± 0.07	0.45 ± 0.05	0.25 ± 0.07	0.00 ± 0.01	0.11 ± 0.04	0.21 ± 0.06	0.29 ± 0.05	0.33 ± 0.05	0.00 ± 0.00
Connectionist	0.56 ± 0.18	0.36 ± 0.22	0.48 ± 0.15	0.74 ± 0.12	0.19 ± 0.17	0.37 ± 0.24	0.44 ± 0.25	0.67 ± 0.18	0.67 ± 0.17	0.25 ± 0.14
Contrac	0.12 ± 0.06	0.23 ± 0.08	0.27 ± 0.06	0.12 ± 0.05	0.28 ± 0.04	0.29 ± 0.05	0.28 ± 0.06	0.31 ± 0.05	0.32 ± 0.05	0.15 ± 0.04
Covid-19	0.60 ± 0.52	0.53 ± 0.50	0.63 ± 0.48	0.50 ± 0.55	0.40 ± 0.52	0.43 ± 0.50	0.53 ± 0.50	0.53 ± 0.50	0.53 ± 0.50	0.73 ± 0.44
Credit-approval	0.63 ± 0.09	0.52 ± 0.08	0.70 ± 0.08	0.64 ± 0.12	0.71 ± 0.11	0.70 ± 0.10	0.69 ± 0.09	0.70 ± 0.05	0.68 ± 0.08	0.71 ± 0.09
Crowdsource	0.29 ± 0.06	0.64 ± 0.05	0.76 ± 0.03	0.90 ± 0.02	0.27 ± 0.04	0.27 ± 0.04	0.27 ± 0.04	0.84 ± 0.04	0.84 ± 0.05	0.53 ± 0.05
Crx	0.66 ± 0.10	0.53 ± 0.07	0.72 ± 0.08	0.62 ± 0.10	0.03 ± 0.08	0.09 ± 0.11	0.12 ± 0.13	0.66 ± 0.09	0.67 ± 0.06	0.71 ± 0.09
Cryother	0.79 ± 0.28	0.66 ± 0.33	0.87 ± 0.16	0.80 ± 0.16	0.64 ± 0.26	0.66 ± 0.26	0.66 ± 0.26	0.75 ± 0.20	0.73 ± 0.18	0.62 ± 0.21
Cylinder-bands	0.16 ± 0.10	0.23 ± 0.15	0.41 ± 0.12	0.34 ± 0.16	0.46 ± 0.10	0.55 ± 0.11	0.60 ± 0.07	0.43 ± 0.11	0.45 ± 0.12	0.23 ± 0.10
Dbworld-bodies	0.07 ± 0.15	0.48 ± 0.34	0.61 ± 0.33	0.11 ± 0.22	0.00 ± 0.00	0.00 ± 0.00	0.22 ± 0.28	/	/	0.72 ± 0.27
Dbworld-bodies-stemmed	0.07 ± 0.15	0.51 ± 0.26	0.74 ± 0.27	0.24 ± 0.30	0.00 ± 0.00	0.11 ± 0.17	0.44 ± 0.29	/	/	0.51 ± 0.37
Dbworld-subjects	0.00 ± 0.00	0.79 ± 0.19	0.48 ± 0.21	0.61 ± 0.27	0.00 ± 0.00	0.00 ± 0.00	0.00 ± 0.00	0.79 ± 0.27	0.76 ± 0.31	0.20 ± 0.26
Dbworld-subjects-stemmed	0.00 ± 0.00	0.73 ± 0.26	0.54 ± 0.33	0.68 ± 0.20	0.00 ± 0.00	0.00 ± 0.00	0.00 ± 0.00	0.76 ± 0.24	0.73 ± 0.23	0.25 ± 0.19
Dermatology	0.81 ± 0.06	0.97 ± 0.03	0.96 ± 0.04	0.94 ± 0.05	0.97 ± 0.03	0.97 ± 0.03	0.97 ± 0.03	0.98 ± 0.02	0.97 ± 0.02	0.33 ± 0.04
Diabetes	0.29 ± 0.13	0.46 ± 0.13	0.41 ± 0.13	0.33 ± 0.12	0.00 ± 0.00	0.00 ± 0.00	0.00 ± 0.00	0.45 ± 0.10	0.45 ± 0.12	0.32 ± 0.15
Diabetic	0.28 ± 0.09	0.17 ± 0.04	0.29 ± 0.09	0.23 ± 0.10	0.13 ± 0.10	0.16 ± 0.13	0.16 ± 0.12	0.44 ± 0.12	0.45 ± 0.12	0.06 ± 0.11
Divorce	0.95 ± 0.06	0.95 ± 0.06	0.90 ± 0.11	0.95 ± 0.06	0.95 ± 0.06	0.95 ± 0.06	0.95 ± 0.06	0.95 ± 0.06	0.95 ± 0.06	0.90 ± 0.11
Dota2train	0.00 ± 0.00	0.08 ± 0.07	0.08 ± 0.11	0.09 ± 0.08	0.00 ± 0.00	0.03 ± 0.08	0.03 ± 0.07	-0.00 ± 0.04	0.01 ± 0.04	0.08 ± 0.08
Dow-jones-index	0.30 ± 0.08	-0.01 ± 0.10	0.45 ± 0.08	0.09 ± 0.13	0.00 ± 0.00	0.00 ± 0.00	0.00 ± 0.00	0.09 ± 0.11	0.09 ± 0.09	0.13 ± 0.06
Dry-bean-dataset	0.92 ± 0.02	0.88 ± 0.02	0.92 ± 0.01	0.93 ± 0.01	0.50 ± 0.04	0.50 ± 0.04	0.50 ± 0.04	0.91 ± 0.02	0.91 ± 0.02	0.61 ± 0.02
Early-stage-diabetes-data-upload	0.64 ± 0.16	0.73 ± 0.06	0.92 ± 0.06	0.96 ± 0.05	0.88 ± 0.09	0.89 ± 0.08	0.90 ± 0.08	0.92 ± 0.06	0.92 ± 0.07	0.65 ± 0.08
Echocardiogram	0.12 ± 0.21	0.53 ± 0.26	0.52 ± 0.22	0.43 ± 0.26	0.54 ± 0.22	0.53 ± 0.28	0.53 ± 0.28	0.52 ± 0.20	0.52 ± 0.25	0.63 ± 0.18
Ecoli	0.58 ± 0.07	0.82 ± 0.07	0.78 ± 0.10	0.73 ± 0.08	0.81 ± 0.06	0.81 ± 0.06	0.82 ± 0.05	0.81 ± 0.07	0.79 ± 0.05	0.53 ± 0.10
Eegeyesate	0.32 ± 0.03	0.01 ± 0.02	0.69 ± 0.02	0.67 ± 0.04	0.00 ± 0.00	0.00 ± 0.00	0.00 ± 0.00	0.06 ± 0.04	0.08 ± 0.05	0.22 ± 0.03
Electrical	0.62 ± 0.19	0.94 ± 0.11	1.00 ± 0.00	0.80 ± 0.16	0.58 ± 0.20	0.59 ± 0.17	0.62 ± 0.16	0.97 ± 0.04	0.97 ± 0.04	0.99 ± 0.02
Energy-y1	0.68 ± 0.02	0.70 ± 0.05	0.96 ± 0.02	0.62 ± 0.06	0.81 ± 0.02	0.83 ± 0.03	0.84 ± 0.05	0.82 ± 0.03	0.80 ± 0.03	0.74 ± 0.03
Energy-y2	0.84 ± 0.05	0.72 ± 0.05	0.84 ± 0.04	0.62 ± 0.05	0.84 ± 0.03	0.85 ± 0.02	0.86 ± 0.02	0.86 ± 0.04	0.86 ± 0.04	0.81 ± 0.05
Extentionofz-alizadehsani	0.00 ± 0.00	0.84 ± 0.09	0.99 ± 0.03	0.78 ± 0.11	0.00 ± 0.00	0.00 ± 0.00	0.00 ± 0.00	0.95 ± 0.05	0.95 ± 0.05	0.71 ± 0.09
Fertility	0.00 ± 0.00	0.00 ± 0.00	-0.03 ± 0.06	0.14 ± 0.39	0.00 ± 0.00	0.10 ± 0.32	0.19 ± 0.43	0.43 ± 0.46	0.43 ± 0.47	0.00 ± 0.00
First-order	0.00 ± 0.00	0.03 ± 0.03	1.00 ± 0.00	0.97 ± 0.03	0.00 ± 0.00	0.66 ± 0.14	0.87 ± 0.07	0.99 ± 0.02	0.99 ± 0.02	0.18 ± 0.20
Flags	0.28 ± 0.14	0.34 ± 0.08	0.51 ± 0.10	0.31 ± 0.11	0.38 ± 0.11	0.41 ± 0.12	0.40 ± 0.15	0.34 ± 0.14	0.37 ± 0.13	0.45 ± 0.09
Foresttypes	0.92 ± 0.06	0.93 ± 0.06	0.93 ± 0.08	0.94 ± 0.05	0.01 ± 0.02	0.04 ± 0.04	0.04 ± 0.04	0.96 ± 0.05	0.96 ± 0.05	0.76 ± 0.11
Garments-worker-productivity	0.23 ± 0.03	0.07 ± 0.04	0.29 ± 0.08	0.02 ± 0.05	0.09 ± 0.04	0.10 ± 0.04	0.11 ± 0.04	0.20 ± 0.07	0.20 ± 0.05	0.08 ± 0.05
Gender-name-dataset	0.04 ± 0.04	0.00 ± 0.01	0.00 ± 0.00	0.16 ± 0.04	0.15 ± 0.03	0.16 ± 0.05	0.16 ± 0.04	0.00 ± 0.00	0.00 ± 0.00	0.08 ± 0.03
Gesture-a1-raw	0.83 ± 0.03	0.61 ± 0.04	0.87 ± 0.03	0.95 ± 0.02	0.00 ± 0.00	0.00 ± 0.00	0.00 ± 0.00	0.87 ± 0.03	0.87 ± 0.02	0.95 ± 0.02
Gesture-a1-va3	0.48 ± 0.04	0.41 ± 0.05	0.50 ± 0.07	0.62 ± 0.04	0.00 ± 0.00	0.00 ± 0.00	0.00 ± 0.00	0.55 ± 0.03	0.55 ± 0.03	0.39 ± 0.03
Gesture-a2-raw	0.83 ± 0.03	0.56 ± 0.04	0.85 ± 0.04	0.92 ± 0.04	0.00 ± 0.00	0.00 ± 0.00	0.00 ± 0.00	0.85 ± 0.03	0.85 ± 0.03	0.92 ± 0.04
Gesture-a2-va3	0.40 ± 0.04	0.25 ± 0.08	0.38 ± 0.03	0.53 ± 0.06	0.00 ± 0.00	0.00 ± 0.00	0.00 ± 0.00	0.43 ± 0.05	0.43 ± 0.06	0.30 ± 0.06
Gesture-a3-raw	0.87 ± 0.03	0.49 ± 0.05	0.90 ± 0.02	0.95 ± 0.01	0.00 ± 0.00	0.00 ± 0.00	0.00 ± 0.00	0.86 ± 0.02	0.85 ± 0.03	0.94 ± 0.02
Gesture-a3-va3	0.42 ± 0.04	0.31 ± 0.04	0.68 ± 0.04	0.86 ± 0.02	0.00 ± 0.00	0.00 ± 0.00	0.00 ± 0.00	0.51 ± 0.05	0.53 ± 0.04	0.30 ± 0.03
Gesture-b1-raw	0.88 ± 0.04	0.33 ± 0.07	0.90 ± 0.04	0.93 ± 0.04	0.01 ± 0.01	0.01 ± 0.01	0.01 ± 0.01	0.83 ± 0.06	0.85 ± 0.03	0.88 ± 0.03
Gesture-b1-va3	0.43 ± 0.09	0.16 ± 0.08	0.59 ± 0.06	0.87 ± 0.04	0.00 ± 0.00	0.00 ± 0.00	0.00 ± 0.00	0.47 ± 0.05	0.49 ± 0.07	0.18 ± 0.06
Gesture-b3-raw	0.86 ± 0.04	0.45 ± 0.05	0.88 ± 0.04	0.94 ± 0.01	0.00 ± 0.00	0.00 ± 0.00	0.00 ± 0.00	0.85 ± 0.05	0.84 ± 0.05	0.94 ± 0.03
Gesture-b3-va3	0.25 ± 0.05	0.30 ± 0.05	0.44 ± 0.05	0.71 ± 0.04	0.00 ± 0.00	0.00 ± 0.00	0.00 ± 0.00	0.45 ± 0.07	0.42 ± 0.07	0.19 ± 0.08
Gesture-c1-raw	0.83 ± 0.04	0.54 ± 0.03	0.86 ± 0.03	0.93 ± 0.02	0.00 ± 0.00	0.00 ± 0.00	0.00 ± 0.00	0.84 ± 0.04	0.84 ± 0.05	0.92 ± 0.03
Gesture-c1-va3	0.39 ± 0.05	0.38 ± 0.05	0.46 ± 0.05	0.62 ± 0.04	0.00 ± 0.00	0.00 ± 0.00	0.00 ± 0.00	0.54 ± 0.04	0.52 ± 0.05	0.25 ± 0.06
Gesture-c3-raw	0.85 ± 0.03	0.49 ± 0.07	0.84 ± 0.05	0.91 ± 0.03	0.00 ± 0.00	0.00 ± 0.00	0.00 ± 0.00	0.82 ± 0.04	0.79 ± 0.03	0.93 ± 0.02
Gesture-c3-va3	0.31 ± 0.05	0.30 ± 0.03	0.42 ± 0.05	0.51 ± 0.06	0.00 ± 0.00	0.00 ± 0.00	0.00 ± 0.00	0.45 ± 0.05	0.43 ± 0.07	0.25 ± 0.04
Glass	0.52 ± 0.16	0.36 ± 0.13	0.56 ± 0.11	0.60 ± 0.11	0.61 ± 0.10	0.60 ± 0.12	0.59 ± 0.12	0.54 ± 0.13	0.55 ± 0.08	0.38 ± 0.13
Go-track-tracks	0.67 ± 0.21	0.65 ± 0.09	0.75 ± 0.16	0.82 ± 0.17	0.44 ± 0.26	0.44 ± 0.24	0.44 ± 0.24	0.68 ± 0.21	0.69 ± 0.18	0.52 ± 0.26
Haberman-survival	0.03 ± 0.11	0.19 ± 0.16	0.17 ± 0.16	0.09 ± 0.16	0.11 ± 0.14	0.09 ± 0.10	0.09 ± 0.10	0.20 ± 0.19	0.23 ± 0.20	0.18 ± 0.15
Hayes-roth	0.47 ± 0.16	0.62 ± 0.20	0.70 ± 0.10	0.71 ± 0.10	0.79 ± 0.11	0.71 ± 0.10	0.70 ± 0.10	0.56 ± 0.20	0.54 ± 0.21	0.10 ± 0.11
Hcc-data	0.05 ± 0.13	0.32 ± 0.20	0.16 ± 0.16	0.24 ± 0.19	0.00 ± 0.00	0.00 ± 0.00	0.00 ± 0.00	0.24 ± 0.32	0.23 ± 0.24	0.33 ± 0.24
Hcvdat	0.10 ± 0.11	0.66 ± 0.16	0.66 ± 0.09	0.52 ± 0.11	0.00 ± 0.00	0.00 ± 0.00	0.00 ± 0.00	0.69 ± 0.14	0.67 ± 0.13	0.44 ± 0.13
Heart-cleveland	0.00 ± 0.01	0.31 ± 0.14	0.23 ± 0.10	0.29 ± 0.09	0.28 ± 0.14	0.32 ± 0.14	0.30 ± 0.14	0.27 ± 0.09	0.28 ± 0.10	0.10 ± 0.11
Heart-hungarian	0.52 ± 0.18	0.59 ± 0.17	0.52 ± 0.15	0.51 ± 0.18	0.59 ± 0.17	0.60 ± 0.15	0.62 ± 0.14	0.52 ± 0.18	0.51 ± 0.17	0.53 ± 0.21
Heart-switzerland	0.10 ± 0.12	0.18 ± 0.15	0.15 ± 0.17	0.06 ± 0.19	0.06 ± 0.10	0.10 ± 0.10	0.13 ± 0.11	0.19 ± 0.21	0.20 ± 0.17	-0.01 ± 0.18
Heart-va	0.05 ± 0.12	0.14 ± 0.13	0.12 ± 0.09	0.15 ± 0.10	0.13 ± 0.13	0.11 ± 0.11	0.09 ± 0.12	0.09 ± 0.13	0.07 ± 0.14	0.04 ± 0.13
Heart-failure-clinical-records-dataset	0.05 ± 0.07	0.41 ± 0.17	0.54 ± 0.14	0.15 ± 0.18	0.00 ± 0.00	0.00 ± 0.00	0.00 ± 0.00	0.37 ± 0.12	0.47 ± 0.21	0.65 ± 0.17
Hepatitis	0.00 ± 0.00	0.51 ± 0.30	0.36 ± 0.33	0.41 ± 0.23	0.35 ± 0.29	0.42 ± 0.40	0.42 ± 0.40	0.40 ± 0.35	0.29 ± 0.34	-0.04 ± 0.19
Hill-valley	-0.00 ± 0.12	-0.02 ± 0.05	-0.00 ± 0.02	0.04 ± 0.07	0.04 ± 0.13	0.04 ± 0.15	0.06 ± 0.14	0.13 ± 0.15	0.07 ± 0.05	-0.05 ± 0.10
Hiv1625data	0.80 ± 0.06	0.82 ± 0.05	0.73 ± 0.08	0.78 ± 0.05	0.76 ± 0.07	0.81 ± 0.07	0.82 ± 0.07	0.87 ± 0.06	0.82 ± 0.07	0.32 ± 0.08
Hiv746data	0.85 ± 0.07	0.82 ± 0.07	0.65 ± 0.10	0.73 ± 0.07	0.79 ± 0.08	0.82 ± 0.06	0.82 ± 0.07	0.85 ± 0.05	0.85 ± 0.07	0.61 ± 0.09
Horse-colic	0.34 ± 0.09	0.43 ± 0.12	0.71 ± 0.12	0.50 ± 0.15	0.63 ± 0.11	0.66 ± 0.12	0.67 ± 0.13	0.56 ± 0.13	0.56 ± 0.18	0.59 ± 0.12
Htru	0.80 ± 0.04	0.71 ± 0.02	0.86 ± 0.03	0.83 ± 0.02	0.06 ± 0.03	0.15 ± 0.03	0.15 ± 0.03	0.87 ± 0.02	0.87 ± 0.02	0.85 ± 0.02
Hypothyroid	0.00 ± 0.00	0.60 ± 0.08	0.97 ± 0.02	0.36 ± 0.08	0.08 ± 0.06	0.16 ± 0.09	0.17 ± 0.09	0.56 ± 0.12	0.51 ± 0.13	0.76 ± 0.06
Ibeacon-rssi-labeled	0.23 ± 0.03	0.19 ± 0.02	0.25 ± 0.03	0.36 ± 0.04	0.33 ± 0.02	0.36 ± 0.03	0.37 ± 0.03	0.27 ± 0.04	0.27 ± 0.04	0.03 ± 0.02
Ilpd-indian-liver	0.02 ± 0.06	0.25 ± 0.06	0.01 ± 0.11	0.18 ± 0.11	0.00 ± 0.00	0.02 ± 0.08	0.07 ± 0.07	0.09 ± 0.12	0.10 ± 0.12	0.04 ± 0.10
Image-segmentation	0.86 ± 0.06	0.74 ± 0.06	0.87 ± 0.09	0.85 ± 0.05	0.85 ± 0.04	0.85 ± 0.05	0.84 ± 0.05	0.88 ± 0.06	0.88 ± 0.06	0.52 ± 0.10
Immunotherapy	0.00 ± 0.00	0.13 ± 0.33	0.37 ± 0.39	0.06 ± 0.45	0.00 ± 0.00	0.00 ± 0.00	0.00 ± 0.00	0.37 ± 0.38	0.34 ± 0.28	0.49 ± 0.30
Impensdata	0.15 ± 0.09	0.58 ± 0.11	0.00 ± 0.00	0.42 ± 0.12	0.00 ± 0.00	0.00 ± 0.00	0.00 ± 0.00	0.63 ± 0.09	0.51 ± 0.16	0.15 ± 0.07
In-vehicle-coupon-recommendation	0.01 ± 0.00	0.29 ± 0.03	0.39 ± 0.02	0.28 ± 0.03	0.36 ± 0.03	0.37 ± 0.03	0.38 ± 0.03	0.39 ± 0.02	0.41 ± 0.02	0.20 ± 0.05
Indian	0.02 ± 0.04	0.25 ± 0.06	0.18 ± 0.14	0.17 ± 0.11	0.05 ± 0.08	0.03 ± 0.07	0.03 ± 0.07	0.13 ± 0.14	0.13 ± 0.17	0.04 ± 0.10
Ionosphere	0.73 ± 0.08	0.64 ± 0.11	0.81 ± 0.07	0.68 ± 0.11	0.87 ± 0.05	0.89 ± 0.07	0.91 ± 0.07	0.78 ± 0.06	0.79 ± 0.08	0.58 ± 0.17
Iris	0.72 ± 0.17	0.90 ± 0.08	0.94 ± 0.08	0.93 ± 0.08	0.95 ± 0.05	0.94 ± 0.07	0.95 ± 0.05	0.96 ± 0.05	0.96 ± 0.05	0.88 ± 0.09
Jain	0.82 ± 0.13	0.86 ± 0.11	0.99 ± 0.04	1.00 ± 0.00	1.00 ± 0.00	1.00 ± 0.00	1.00 ± 0.00	0.87 ± 0.09	0.87 ± 0.09	0.86 ± 0.10
Jsbach-chorals-harmony	0.00 ± 0.00	0.84 ± 0.03	0.82 ± 0.03	0.88 ± 0.03	0.79 ± 0.04	0.84 ± 0.02	0.85 ± 0.04	0.88 ± 0.03	0.86 ± 0.05	0.79 ± 0.03
Knowledge	0.44 ± 0.10	0.82 ± 0.16	0.89 ± 0.09	0.64 ± 0.12	0.77 ± 0.14	0.84 ± 0.13	0.88 ± 0.11	0.89 ± 0.08	0.89 ± 0.08	0.75 ± 0.14
Lasvegastripadvisorreviews	-0.01 ± 0.04	0.03 ± 0.05	0.02 ± 0.06	0.01 ± 0.04	-0.02 ± 0.04	-0.04 ± 0.05	-0.01 ± 0.06	0.01 ± 0.08	0.02 ± 0.06	-0.00 ± 0.04
Leaf	0.57 ± 0.06	0.71 ± 0.09	0.59 ± 0.08	-0.00 ± 0.03	0.14 ± 0.04	0.21 ± 0.05	0.23 ± 0.06	0.49 ± 0.08	0.46 ± 0.08	0.21 ± 0.05
Led-display	0.67 ± 0.04	0.69 ± 0.03	0.68 ± 0.04	0.68 ± 0.04	0.69 ± 0.05	0.69 ± 0.04	0.68 ± 0.04	0.69 ± 0.04	0.69 ± 0.04	0.10 ± 0.01
Lenses	0.50 ± 0.53	0.50 ± 0.53	0.70 ± 0.42	0.75 ± 0.42	0.65 ± 0.47	0.70 ± 0.42	0.70 ± 0.42	0.50 ± 0.47	0.50 ± 0.47	0.53 ± 0.38
Letter	0.71 ± 0.01	0.63 ± 0.01	0.87 ± 0.01	0.96 ± 0.00	0.95 ± 0.00	0.96 ± 0.00	0.96 ± 0.00	0.82 ± 0.01	0.81 ± 0.01	0.14 ± 0.00
Libras	0.65 ± 0.10	0.61 ± 0.11	0.68 ± 0.11	0.85 ± 0.05	0.80 ± 0.04	0.84 ± 0.05	0.86 ± 0.04	0.79 ± 0.06	0.80 ± 0.06	0.15 ± 0.06
Low-res-spect	0.40 ± 0.08	0.72 ± 0.05	0.74 ± 0.05	0.75 ± 0.06	0.84 ± 0.04	0.84 ± 0.04	0.86 ± 0.04	0.88 ± 0.05	0.87 ± 0.04	0.60 ± 0.08
Lung-cancer	0.32 ± 0.34	0.48 ± 0.34	0.09 ± 0.42	0.27 ± 0.56	0.25 ± 0.35	0.36 ± 0.34	0.26 ± 0.40	0.18 ± 0.46	0.23 ± 0.39	0.19 ± 0.30
Lymphography	0.51 ± 0.16	0.65 ± 0.17	0.52 ± 0.16	0.53 ± 0.25	0.72 ± 0.18	0.71 ± 0.19	0.67 ± 0.17	0.62 ± 0.19	0.60 ± 0.19	0.53 ± 0.17
Magic	0.42 ± 0.04	0.35 ± 0.06	0.61 ± 0.05	0.55 ± 0.05	0.65 ± 0.05	0.65 ± 0.04	0.65 ± 0.03	0.65 ± 0.05	0.64 ± 0.04	0.37 ± 0.10
Mammographic	0.57 ± 0.07	0.57 ± 0.07	0.64 ± 0.04	0.50 ± 0.10	0.65 ± 0.04	0.64 ± 0.05	0.65 ± 0.05	0.63 ± 0.06	0.62 ± 0.06	0.63 ± 0.03
Miniboone	0.30 ± 0.09	0.02 ± 0.01	0.64 ± 0.06	0.58 ± 0.05	0.54 ± 0.08	0.59 ± 0.07	0.61 ± 0.06	0.56 ± 0.05	0.56 ± 0.05	0.49 ± 0.06
Molec-biol-promoter	0.77 ± 0.33	0.77 ± 0.22	0.49 ± 0.20	0.45 ± 0.24	0.65 ± 0.27	0.69 ± 0.24	0.69 ± 0.24	0.56 ± 0.22	0.54 ± 0.21	0.39 ± 0.22
Molec-biol-splice	0.00 ± 0.00	0.88 ± 0.03	0.88 ± 0.03	0.43 ± 0.03	0.79 ± 0.03	0.79 ± 0.03	0.79 ± 0.02	0.75 ± 0.04	0.75 ± 0.03	0.41 ± 0.03
Monks-1	0.84 ± 0.19	0.43 ± 0.23	0.93 ± 0.14	0.41 ± 0.20	0.61 ± 0.24	0.66 ± 0.22	0.66 ± 0.19	0.77 ± 0.30	0.79 ± 0.34	0.46 ± 0.20
Monks-2	0.00 ± 0.00	-0.09 ± 0.21	0.50 ± 0.24	0.15 ± 0.24	-0.00 ± 0.14	0.12 ± 0.27	0.21 ± 0.26	0.43 ± 0.26	0.45 ± 0.17	-0.04 ± 0.07
Monks-3	0.87 ± 0.17	0.77 ± 0.12	0.87 ± 0.17	0.49 ± 0.33	0.80 ± 0.15	0.79 ± 0.16	0.79 ± 0.16	0.68 ± 0.22	0.78 ± 0.14	0.56 ± 0.08
Mushroom	0.81 ± 0.02	0.78 ± 0.02	1.00 ± 0.00	1.00 ± 0.00	1.00 ± 0.00	1.00 ± 0.00	1.00 ± 0.00	1.00 ± 0.00	1.00 ± 0.00	0.97 ± 0.01
Musk-1	0.67 ± 0.12	0.48 ± 0.13	0.70 ± 0.13	0.70 ± 0.09	0.81 ± 0.09	0.85 ± 0.06	0.87 ± 0.06	0.88 ± 0.05	0.87 ± 0.06	0.22 ± 0.18
Musk-2	0.33 ± 0.07	0.50 ± 0.06	0.83 ± 0.06	0.86 ± 0.03	0.84 ± 0.05	0.90 ± 0.04	0.92 ± 0.03	0.96 ± 0.03	0.95 ± 0.03	0.54 ± 0.07
Newdiagnosis	1.00 ± 0.00	1.00 ± 0.00	1.00 ± 0.00	1.00 ± 0.00	1.00 ± 0.00	1.00 ± 0.00	1.00 ± 0.00	1.00 ± 0.00	1.00 ± 0.00	0.55 ± 0.14
Nursery	0.82 ± 0.02	0.85 ± 0.01	0.99 ± 0.00	0.84 ± 0.01	0.97 ± 0.00	0.98 ± 0.00	0.98 ± 0.00	0.94 ± 0.01	0.92 ± 0.02	0.57 ± 0.02
Obesitydataset-raw-and-data-sinthetic	0.80 ± 0.03	0.62 ± 0.03	0.93 ± 0.02	0.79 ± 0.03	0.88 ± 0.03	0.90 ± 0.03	0.91 ± 0.03	0.93 ± 0.03	0.93 ± 0.02	0.62 ± 0.04
Obs-network-dataset-2-aug27	0.94 ± 0.03	0.60 ± 0.07	0.99 ± 0.02	0.99 ± 0.01	0.97 ± 0.01	0.97 ± 0.01	0.97 ± 0.01	0.95 ± 0.02	0.97 ± 0.02	0.86 ± 0.03
Occupancy-data	0.92 ± 0.02	0.91 ± 0.02	0.97 ± 0.02	0.98 ± 0.01	0.54 ± 0.06	0.57 ± 0.06	0.57 ± 0.06	0.96 ± 0.02	0.96 ± 0.02	0.97 ± 0.02
Occupancy-data2	0.95 ± 0.01	0.89 ± 0.01	0.98 ± 0.00	0.98 ± 0.01	0.30 ± 0.03	0.42 ± 0.03	0.42 ± 0.03	0.98 ± 0.01	0.98 ± 0.00	0.98 ± 0.00
Occupancy-data3	0.95 ± 0.02	0.93 ± 0.02	0.99 ± 0.00	0.98 ± 0.01	0.28 ± 0.03	0.37 ± 0.03	0.37 ± 0.03	0.96 ± 0.01	0.96 ± 0.01	0.98 ± 0.01
Old	0.76 ± 0.04	0.88 ± 0.02	0.90 ± 0.03	0.95 ± 0.02	0.90 ± 0.02	0.90 ± 0.03	0.91 ± 0.02	0.94 ± 0.02	0.94 ± 0.02	0.77 ± 0.03
Online-shoppers-intention	0.00 ± 0.00	0.42 ± 0.03	0.57 ± 0.02	0.26 ± 0.03	0.00 ± 0.00	0.00 ± 0.01	0.00 ± 0.01	0.56 ± 0.03	0.55 ± 0.06	0.52 ± 0.04
Oocytes-merluccius-nucleus-4d	0.24 ± 0.07	0.20 ± 0.08	0.42 ± 0.08	0.39 ± 0.08	0.40 ± 0.09	0.49 ± 0.07	0.52 ± 0.08	0.58 ± 0.04	0.56 ± 0.10	0.16 ± 0.14
Oocytes-merluccius-states-2f	0.74 ± 0.08	0.70 ± 0.07	0.80 ± 0.07	0.81 ± 0.06	0.82 ± 0.05	0.83 ± 0.04	0.84 ± 0.04	0.82 ± 0.03	0.81 ± 0.06	0.62 ± 0.10
Oocytes-trisopterus-nucleus-2f	0.23 ± 0.11	0.13 ± 0.10	0.43 ± 0.13	0.48 ± 0.11	0.60 ± 0.08	0.66 ± 0.07	0.65 ± 0.06	0.65 ± 0.08	0.63 ± 0.09	0.16 ± 0.13
Oocytes-trisopterus-states-5b	0.65 ± 0.10	0.51 ± 0.09	0.77 ± 0.05	0.82 ± 0.08	0.83 ± 0.07	0.84 ± 0.06	0.85 ± 0.05	0.87 ± 0.05	0.87 ± 0.06	0.58 ± 0.06
Optdigits	0.88 ± 0.01	0.90 ± 0.01	0.90 ± 0.02	0.98 ± 0.01	0.70 ± 0.02	0.72 ± 0.02	0.72 ± 0.02	0.98 ± 0.01	0.98 ± 0.01	0.19 ± 0.01
Optical	0.87 ± 0.02	0.91 ± 0.01	0.89 ± 0.02	0.98 ± 0.01	0.98 ± 0.01	0.99 ± 0.01	0.99 ± 0.01	0.98 ± 0.01	0.98 ± 0.01	0.20 ± 0.01
Ozone	0.00 ± 0.00	0.09 ± 0.03	0.20 ± 0.13	0.16 ± 0.12	0.00 ± 0.00	0.00 ± 0.00	0.00 ± 0.00	0.30 ± 0.18	0.32 ± 0.16	-0.00 ± 0.01
Page-blocks	0.45 ± 0.09	0.55 ± 0.05	0.83 ± 0.02	0.78 ± 0.03	0.77 ± 0.05	0.79 ± 0.05	0.80 ± 0.05	0.78 ± 0.04	0.78 ± 0.03	0.64 ± 0.03
Parkingbirmingham	0.80 ± 0.02	0.75 ± 0.02	1.00 ± 0.00	0.97 ± 0.01	0.57 ± 0.04	0.60 ± 0.04	0.60 ± 0.04	0.86 ± 0.02	0.87 ± 0.02	1.00 ± 0.00
Parkinsons	0.45 ± 0.26	0.40 ± 0.19	0.47 ± 0.16	0.91 ± 0.07	0.59 ± 0.23	0.67 ± 0.21	0.73 ± 0.19	0.76 ± 0.19	0.72 ± 0.23	0.57 ± 0.25
Pasture	0.59 ± 0.34	0.62 ± 0.35	0.66 ± 0.34	0.58 ± 0.41	0.00 ± 0.00	0.00 ± 0.00	0.00 ± 0.00	0.64 ± 0.40	0.60 ± 0.42	0.47 ± 0.36
Pbc	0.43 ± 0.12	0.51 ± 0.16	0.50 ± 0.19	0.21 ± 0.18	0.00 ± 0.00	0.00 ± 0.00	0.00 ± 0.00	0.39 ± 0.11	0.43 ± 0.08	0.39 ± 0.19
Pen	0.86 ± 0.01	0.84 ± 0.01	0.96 ± 0.01	0.99 ± 0.00	0.03 ± 0.01	0.05 ± 0.01	0.05 ± 0.01	0.94 ± 0.01	0.94 ± 0.00	0.32 ± 0.02
Pendigits	0.85 ± 0.01	0.87 ± 0.02	0.96 ± 0.01	0.99 ± 0.00	1.00 ± 0.00	1.00 ± 0.00	1.00 ± 0.00	0.95 ± 0.01	0.94 ± 0.01	0.33 ± 0.02
Pharynx	0.00 ± 0.00	-0.06 ± 0.19	0.00 ± 0.00	-0.08 ± 0.24	0.07 ± 0.22	0.09 ± 0.29	0.04 ± 0.27	0.01 ± 0.16	0.02 ± 0.06	-0.01 ± 0.08
Phishingwebsites	0.69 ± 0.02	0.86 ± 0.01	0.92 ± 0.01	0.94 ± 0.01	0.89 ± 0.01	0.90 ± 0.01	0.90 ± 0.01	0.93 ± 0.01	0.94 ± 0.01	0.77 ± 0.01
Pima	0.28 ± 0.12	0.46 ± 0.13	0.41 ± 0.13	0.33 ± 0.12	0.44 ± 0.09	0.43 ± 0.09	0.43 ± 0.08	0.45 ± 0.10	0.45 ± 0.12	0.32 ± 0.15
Pittsburg-bridges-rel-l	0.39 ± 0.19	0.42 ± 0.24	0.33 ± 0.27	0.51 ± 0.29	0.37 ± 0.26	0.40 ± 0.26	0.51 ± 0.20	0.35 ± 0.26	0.41 ± 0.21	0.46 ± 0.21
Pittsburg-bridges-span	0.24 ± 0.18	0.44 ± 0.25	0.32 ± 0.25	0.29 ± 0.28	0.39 ± 0.25	0.38 ± 0.28	0.37 ± 0.28	0.42 ± 0.18	0.46 ± 0.22	0.12 ± 0.23
Pittsburg-bridges-t-or-d	0.00 ± 0.00	0.19 ± 0.40	0.09 ± 0.34	0.30 ± 0.43	0.09 ± 0.32	0.24 ± 0.45	0.31 ± 0.45	0.30 ± 0.53	0.29 ± 0.54	0.38 ± 0.43
Pittsburg-bridges-type	0.32 ± 0.17	0.41 ± 0.16	0.48 ± 0.13	0.44 ± 0.16	0.34 ± 0.17	0.47 ± 0.24	0.49 ± 0.19	0.47 ± 0.22	0.46 ± 0.21	0.36 ± 0.09
Pittsburg-bridgesmaterial	0.50 ± 0.28	0.59 ± 0.17	0.65 ± 0.23	0.61 ± 0.25	0.62 ± 0.15	0.60 ± 0.13	0.59 ± 0.21	0.60 ± 0.18	0.52 ± 0.16	0.62 ± 0.14
Planning	0.03 ± 0.08	-0.01 ± 0.15	0.00 ± 0.00	0.12 ± 0.20	0.00 ± 0.00	0.01 ± 0.15	-0.05 ± 0.14	0.00 ± 0.18	-0.07 ± 0.20	-0.08 ± 0.18
Plant-margin	0.63 ± 0.04	0.85 ± 0.03	0.48 ± 0.05	0.74 ± 0.03	0.83 ± 0.02	0.85 ± 0.03	0.85 ± 0.03	0.83 ± 0.02	0.83 ± 0.03	0.07 ± 0.02
Plant-shape	0.49 ± 0.04	0.53 ± 0.03	0.46 ± 0.03	0.64 ± 0.03	0.48 ± 0.02	0.56 ± 0.03	0.60 ± 0.03	0.64 ± 0.04	0.65 ± 0.03	0.08 ± 0.02
Plant-texture	0.62 ± 0.03	0.74 ± 0.03	0.51 ± 0.04	0.80 ± 0.03	0.83 ± 0.03	0.85 ± 0.02	0.86 ± 0.02	0.84 ± 0.02	0.83 ± 0.02	0.03 ± 0.01
Poker-hand-training-true	0.00 ± 0.00	0.00 ± 0.00	0.20 ± 0.03	0.06 ± 0.01	0.22 ± 0.02	0.21 ± 0.02	0.20 ± 0.02	0.11 ± 0.02	0.08 ± 0.03	0.00 ± 0.00
Post-operative	0.00 ± 0.00	-0.03 ± 0.14	0.00 ± 0.00	-0.06 ± 0.24	0.00 ± 0.00	-0.02 ± 0.10	-0.02 ± 0.16	-0.10 ± 0.22	-0.09 ± 0.27	-0.03 ± 0.07
Primary-tumor	0.01 ± 0.02	0.36 ± 0.11	0.35 ± 0.10	0.27 ± 0.09	0.37 ± 0.13	0.36 ± 0.12	0.35 ± 0.14	0.33 ± 0.09	0.33 ± 0.10	0.08 ± 0.03
Qsarbioconcentration	-0.00 ± 0.01	-0.01 ± 0.04	-0.03 ± 0.06	0.01 ± 0.15	-0.00 ± 0.01	-0.02 ± 0.05	-0.05 ± 0.06	-0.03 ± 0.08	0.00 ± 0.07	-0.01 ± 0.08
Qsarbiodegradation	0.15 ± 0.07	0.52 ± 0.06	0.63 ± 0.08	0.64 ± 0.05	0.68 ± 0.06	0.69 ± 0.05	0.67 ± 0.06	0.70 ± 0.05	0.71 ± 0.06	0.46 ± 0.09
Qualitative-bankruptcy	0.98 ± 0.05	0.98 ± 0.05	0.96 ± 0.06	1.00 ± 0.00	0.97 ± 0.06	0.97 ± 0.06	0.99 ± 0.03	0.98 ± 0.05	0.98 ± 0.05	0.97 ± 0.06
Ringnorm	0.58 ± 0.03	0.97 ± 0.01	0.83 ± 0.01	0.50 ± 0.03	0.97 ± 0.01	0.97 ± 0.01	0.97 ± 0.01	0.84 ± 0.03	0.83 ± 0.02	0.29 ± 0.02
Risk-factors-cervical-cancer	0.00 ± 0.00	0.41 ± 0.09	0.59 ± 0.19	0.49 ± 0.17	0.00 ± 0.00	0.02 ± 0.09	0.23 ± 0.21	0.53 ± 0.19	0.50 ± 0.22	0.73 ± 0.13
Robotnavigation	0.88 ± 0.02	0.36 ± 0.03	0.99 ± 0.00	0.82 ± 0.02	0.85 ± 0.02	0.87 ± 0.01	0.88 ± 0.01	0.82 ± 0.03	0.82 ± 0.04	0.61 ± 0.03
Sapfile	0.15 ± 0.18	0.30 ± 0.15	0.16 ± 0.21	0.09 ± 0.11	0.14 ± 0.14	0.17 ± 0.11	0.19 ± 0.13	0.19 ± 0.14	0.17 ± 0.19	0.05 ± 0.20
Sat	0.81 ± 0.01	0.75 ± 0.02	0.83 ± 0.01	0.88 ± 0.01	0.03 ± 0.01	0.05 ± 0.01	0.05 ± 0.01	0.87 ± 0.02	0.86 ± 0.01	0.50 ± 0.02
Satelite	0.81 ± 0.01	0.75 ± 0.01	0.83 ± 0.01	0.88 ± 0.01	0.03 ± 0.01	0.05 ± 0.01	0.05 ± 0.01	0.87 ± 0.01	0.87 ± 0.01	0.51 ± 0.02
Scadi	0.34 ± 0.11	0.76 ± 0.13	0.72 ± 0.19	0.72 ± 0.19	0.66 ± 0.12	0.70 ± 0.19	0.74 ± 0.21	0.72 ± 0.14	0.72 ± 0.14	0.41 ± 0.12
Schillingdata	0.02 ± 0.02	0.71 ± 0.04	0.00 ± 0.00	0.43 ± 0.06	0.00 ± 0.00	0.27 ± 0.09	0.53 ± 0.05	0.69 ± 0.06	0.60 ± 0.10	0.01 ± 0.02
Seeds	0.80 ± 0.10	0.87 ± 0.07	0.88 ± 0.09	0.91 ± 0.03	0.91 ± 0.06	0.91 ± 0.06	0.91 ± 0.06	0.93 ± 0.05	0.94 ± 0.05	0.74 ± 0.15
Segment	0.89 ± 0.03	0.77 ± 0.02	0.96 ± 0.01	0.97 ± 0.01	0.60 ± 0.03	0.62 ± 0.03	0.61 ± 0.03	0.95 ± 0.01	0.96 ± 0.01	0.58 ± 0.03
Seismic-bumps	0.00 ± 0.00	0.22 ± 0.09	-0.00 ± 0.00	0.11 ± 0.08	0.00 ± 0.00	0.00 ± 0.00	0.00 ± 0.00	0.06 ± 0.09	0.07 ± 0.06	0.00 ± 0.03
Semeion	0.76 ± 0.04	0.84 ± 0.03	0.73 ± 0.03	0.91 ± 0.02	0.95 ± 0.01	0.96 ± 0.01	0.96 ± 0.01	0.92 ± 0.02	0.92 ± 0.02	0.11 ± 0.00
Setapprocesst1	0.06 ± 0.19	0.16 ± 0.41	0.17 ± 0.45	0.15 ± 0.24	-0.08 ± 0.13	0.07 ± 0.31	0.03 ± 0.24	0.15 ± 0.47	0.13 ± 0.49	0.38 ± 0.47
Setapprocesst10	0.00 ± 0.00	0.01 ± 0.28	0.05 ± 0.32	0.09 ± 0.40	0.00 ± 0.00	0.00 ± 0.00	0.00 ± 0.00	-0.03 ± 0.50	-0.01 ± 0.41	0.00 ± 0.20
Setapprocesst11	0.00 ± 0.00	0.08 ± 0.37	0.24 ± 0.44	0.08 ± 0.33	0.00 ± 0.00	0.00 ± 0.00	0.00 ± 0.00	0.20 ± 0.29	0.29 ± 0.33	-0.03 ± 0.21
Setapprocesst2	0.06 ± 0.19	0.25 ± 0.36	0.34 ± 0.50	0.14 ± 0.48	0.00 ± 0.00	0.00 ± 0.00	0.00 ± 0.00	0.18 ± 0.40	0.24 ± 0.41	0.53 ± 0.44
Setapprocesst3	0.00 ± 0.00	0.22 ± 0.30	0.12 ± 0.35	0.18 ± 0.38	0.00 ± 0.00	0.00 ± 0.00	0.00 ± 0.00	0.23 ± 0.32	0.18 ± 0.28	-0.02 ± 0.35
Setapprocesst4	0.00 ± 0.00	0.01 ± 0.46	-0.12 ± 0.30	-0.02 ± 0.41	0.00 ± 0.00	0.00 ± 0.00	0.00 ± 0.00	-0.04 ± 0.43	-0.07 ± 0.39	0.23 ± 0.34
Setapprocesst5	0.10 ± 0.21	0.01 ± 0.30	-0.11 ± 0.32	0.06 ± 0.34	-0.02 ± 0.07	-0.05 ± 0.10	-0.05 ± 0.10	0.06 ± 0.22	0.11 ± 0.26	0.25 ± 0.46
Setapprocesst6	0.00 ± 0.00	0.24 ± 0.36	0.14 ± 0.37	0.09 ± 0.35	0.00 ± 0.00	0.00 ± 0.00	0.00 ± 0.00	0.11 ± 0.42	0.10 ± 0.37	0.05 ± 0.40
Setapprocesst7	0.06 ± 0.19	0.40 ± 0.38	0.14 ± 0.45	0.09 ± 0.36	0.00 ± 0.00	0.00 ± 0.00	0.00 ± 0.00	0.32 ± 0.46	0.33 ± 0.43	-0.04 ± 0.34
Setapprocesst8	0.00 ± 0.00	0.35 ± 0.25	0.37 ± 0.32	0.08 ± 0.39	0.00 ± 0.00	0.00 ± 0.00	0.00 ± 0.00	0.23 ± 0.39	0.21 ± 0.42	-0.15 ± 0.20
Setapprocesst9	0.00 ± 0.00	0.27 ± 0.34	0.35 ± 0.36	-0.04 ± 0.23	0.00 ± 0.00	0.00 ± 0.00	0.00 ± 0.00	0.32 ± 0.42	0.27 ± 0.36	-0.08 ± 0.30
Shillbiddingdataset	0.25 ± 0.06	0.85 ± 0.02	0.98 ± 0.01	0.96 ± 0.01	0.98 ± 0.01	0.98 ± 0.01	0.98 ± 0.01	0.98 ± 0.01	0.99 ± 0.01	0.87 ± 0.02
Shuttle-landing-control	0.90 ± 0.32	0.70 ± 0.48	0.90 ± 0.32	0.90 ± 0.32	0.90 ± 0.32	0.90 ± 0.32	0.90 ± 0.32	0.80 ± 0.42	0.70 ± 0.48	0.80 ± 0.42
Somervillehappinesssurvey2015	0.12 ± 0.26	0.18 ± 0.20	0.28 ± 0.34	0.16 ± 0.25	0.19 ± 0.22	0.16 ± 0.20	0.16 ± 0.23	0.17 ± 0.19	0.18 ± 0.23	0.31 ± 0.32
Sonar	0.52 ± 0.15	0.37 ± 0.18	0.42 ± 0.14	0.73 ± 0.14	0.29 ± 0.11	0.39 ± 0.18	0.46 ± 0.19	0.64 ± 0.21	0.63 ± 0.19	0.25 ± 0.22
Soybean	0.65 ± 0.07	0.91 ± 0.06	0.86 ± 0.06	0.86 ± 0.05	0.89 ± 0.07	0.91 ± 0.05	0.91 ± 0.05	0.89 ± 0.08	0.90 ± 0.07	0.22 ± 0.04
Spambase	0.48 ± 0.03	0.59 ± 0.03	0.85 ± 0.03	0.81 ± 0.02	0.86 ± 0.02	0.86 ± 0.02	0.86 ± 0.02	0.81 ± 0.03	0.78 ± 0.13	0.54 ± 0.04
Speaker-accent	0.04 ± 0.04	0.47 ± 0.12	0.57 ± 0.13	0.73 ± 0.12	0.22 ± 0.08	0.32 ± 0.10	0.32 ± 0.10	0.71 ± 0.11	0.64 ± 0.12	0.13 ± 0.08
Spect	0.14 ± 0.23	0.36 ± 0.42	0.19 ± 0.40	-0.08 ± 0.36	0.04 ± 0.28	0.06 ± 0.34	0.05 ± 0.31	-0.05 ± 0.44	-0.03 ± 0.38	0.19 ± 0.28
Spectf	0.38 ± 0.24	0.50 ± 0.24	0.47 ± 0.25	0.35 ± 0.38	0.35 ± 0.13	0.35 ± 0.13	0.53 ± 0.28	0.42 ± 0.29	0.45 ± 0.31	0.43 ± 0.17
Statlog-australian-credit	-0.00 ± 0.01	0.09 ± 0.08	0.15 ± 0.15	0.00 ± 0.06	0.02 ± 0.04	-0.00 ± 0.12	0.02 ± 0.12	0.06 ± 0.13	0.04 ± 0.08	0.09 ± 0.15
Statlog-german-credit	0.00 ± 0.00	0.39 ± 0.14	0.35 ± 0.12	0.23 ± 0.10	0.36 ± 0.11	0.41 ± 0.09	0.42 ± 0.11	0.30 ± 0.07	0.32 ± 0.08	0.12 ± 0.06
Statlog-heart	0.66 ± 0.16	0.66 ± 0.15	0.52 ± 0.20	0.50 ± 0.17	0.65 ± 0.10	0.60 ± 0.16	0.59 ± 0.19	0.56 ± 0.14	0.60 ± 0.17	0.41 ± 0.12
Statlog-image	0.89 ± 0.03	0.77 ± 0.02	0.96 ± 0.01	0.97 ± 0.01	0.93 ± 0.01	0.94 ± 0.01	0.95 ± 0.01	0.95 ± 0.01	0.96 ± 0.01	0.58 ± 0.03
Statlog-landsat	0.82 ± 0.02	0.75 ± 0.03	0.83 ± 0.02	0.88 ± 0.02	0.87 ± 0.02	0.88 ± 0.02	0.88 ± 0.02	0.86 ± 0.02	0.86 ± 0.02	0.51 ± 0.02
Statlog-shuttle	0.38 ± 0.02	0.75 ± 0.03	1.00 ± 0.00	1.00 ± 0.00	0.99 ± 0.00	0.99 ± 0.00	0.99 ± 0.00	0.99 ± 0.00	0.99 ± 0.00	0.86 ± 0.01
Statlog-vehicle	0.55 ± 0.04	0.27 ± 0.04	0.63 ± 0.08	0.60 ± 0.06	0.69 ± 0.05	0.74 ± 0.03	0.74 ± 0.03	0.76 ± 0.04	0.74 ± 0.06	0.35 ± 0.07
Steel-plates	0.46 ± 0.04	0.52 ± 0.05	0.69 ± 0.04	0.64 ± 0.03	0.68 ± 0.04	0.70 ± 0.03	0.70 ± 0.04	0.65 ± 0.04	0.65 ± 0.04	0.30 ± 0.05
Synthetic-control	0.87 ± 0.03	0.94 ± 0.04	0.90 ± 0.04	0.96 ± 0.03	0.99 ± 0.01	0.99 ± 0.01	0.99 ± 0.01	0.99 ± 0.01	0.99 ± 0.01	0.48 ± 0.05
Teaching	0.25 ± 0.17	0.31 ± 0.13	0.39 ± 0.17	0.43 ± 0.20	0.34 ± 0.22	0.42 ± 0.19	0.43 ± 0.18	0.31 ± 0.17	0.28 ± 0.14	0.20 ± 0.16
Thoraricsurgery	0.00 ± 0.00	0.05 ± 0.15	0.00 ± 0.04	0.04 ± 0.17	0.00 ± 0.00	-0.01 ± 0.02	-0.01 ± 0.02	0.11 ± 0.13	0.15 ± 0.20	-0.01 ± 0.04
Thyroid	0.61 ± 0.21	0.92 ± 0.09	0.83 ± 0.14	0.94 ± 0.05	0.27 ± 0.19	0.34 ± 0.17	0.34 ± 0.17	0.93 ± 0.07	0.93 ± 0.07	0.79 ± 0.20
Thyroid-train	0.00 ± 0.00	0.63 ± 0.10	0.98 ± 0.02	0.39 ± 0.10	0.51 ± 0.10	0.60 ± 0.08	0.67 ± 0.08	0.72 ± 0.10	0.68 ± 0.09	0.78 ± 0.05
Tic-tac-toe	0.00 ± 0.00	0.27 ± 0.08	0.86 ± 0.05	1.00 ± 0.00	0.97 ± 0.03	0.98 ± 0.02	0.99 ± 0.02	0.95 ± 0.02	0.95 ± 0.03	0.34 ± 0.05
Titanic	0.40 ± 0.07	0.42 ± 0.06	0.43 ± 0.05	0.43 ± 0.05	0.43 ± 0.06	0.43 ± 0.06	0.43 ± 0.06	0.44 ± 0.06	0.42 ± 0.06	0.44 ± 0.05
Trains	0.70 ± 0.48	0.60 ± 0.52	0.70 ± 0.48	0.60 ± 0.52	0.60 ± 0.52	0.70 ± 0.48	0.70 ± 0.48	0.80 ± 0.42	0.80 ± 0.42	0.00 ± 0.00
Transfusion	0.05 ± 0.07	0.16 ± 0.10	0.34 ± 0.12	0.12 ± 0.12	0.08 ± 0.11	0.06 ± 0.09	0.04 ± 0.10	0.28 ± 0.09	0.30 ± 0.10	0.06 ± 0.08
Trial	0.87 ± 0.07	0.80 ± 0.07	1.00 ± 0.00	0.99 ± 0.02	0.98 ± 0.02	0.99 ± 0.01	0.99 ± 0.01	0.98 ± 0.02	0.99 ± 0.02	1.00 ± 0.00
Turkiye-student-evaluation	0.00 ± 0.00	0.75 ± 0.03	1.00 ± 0.00	0.82 ± 0.03	0.91 ± 0.01	0.93 ± 0.01	0.93 ± 0.01	1.00 ± 0.00	1.00 ± 0.00	1.00 ± 0.00
Unbalanced	0.00 ± 0.00	0.07 ± 0.13	0.00 ± 0.00	0.16 ± 0.36	0.00 ± 0.00	-0.00 ± 0.00	-0.01 ± 0.01	-0.00 ± 0.01	0.04 ± 0.16	0.00 ± 0.00
Urbanlandcover	0.77 ± 0.12	0.75 ± 0.11	0.76 ± 0.09	0.73 ± 0.13	0.00 ± 0.00	0.00 ± 0.00	0.00 ± 0.00	0.74 ± 0.14	0.74 ± 0.14	0.42 ± 0.16
Userknowledgemodeling	0.48 ± 0.11	0.85 ± 0.08	0.92 ± 0.05	0.73 ± 0.14	0.67 ± 0.10	0.79 ± 0.06	0.79 ± 0.06	0.88 ± 0.05	0.90 ± 0.07	0.79 ± 0.11
Vehicle	0.59 ± 0.06	0.27 ± 0.06	0.63 ± 0.08	0.60 ± 0.06	0.07 ± 0.03	0.08 ± 0.04	0.08 ± 0.04	*0.76 ± 0.05*	0.75 ± 0.06	0.36 ± 0.07
Vertebral-column-2classes	0.34 ± 0.23	0.55 ± 0.13	0.57 ± 0.16	0.60 ± 0.11	*0.66 ± 0.13*	0.64 ± 0.15	0.65 ± 0.14	0.63 ± 0.17	0.62 ± 0.16	0.43 ± 0.15
Vertebral-column-3classes	0.50 ± 0.18	0.73 ± 0.09	0.72 ± 0.09	0.66 ± 0.08	0.74 ± 0.09	0.76 ± 0.11	0.75 ± 0.10	*0.77 ± 0.07*	0.76 ± 0.09	0.59 ± 0.11
Veteran	0.03 ± 0.10	0.22 ± 0.21	*0.36 ± 0.17*	0.07 ± 0.21	0.00 ± 0.00	0.00 ± 0.00	0.00 ± 0.00	0.06 ± 0.34	0.19 ± 0.35	0.35 ± 0.18
Vowel	0.59 ± 0.04	0.60 ± 0.05	0.80 ± 0.05	*0.99 ± 0.01*	0.84 ± 0.03	0.90 ± 0.02	0.92 ± 0.02	0.92 ± 0.04	0.93 ± 0.03	0.25 ± 0.05
Wall-following	0.88 ± 0.02	0.36 ± 0.02	*0.99 ± 0.00*	0.82 ± 0.02	0.83 ± 0.02	0.86 ± 0.02	0.87 ± 0.02	0.81 ± 0.02	0.82 ± 0.03	0.61 ± 0.02
Waveform-noise	0.51 ± 0.03	0.70 ± 0.03	0.63 ± 0.02	0.60 ± 0.02	*0.79 ± 0.02*	0.78 ± 0.02	0.77 ± 0.02	0.75 ± 0.02	0.75 ± 0.02	0.31 ± 0.04
Waveform	0.56 ± 0.02	0.72 ± 0.02	0.64 ± 0.02	0.65 ± 0.03	*0.80 ± 0.02*	0.78 ± 0.02	0.78 ± 0.02	0.76 ± 0.02	0.75 ± 0.02	0.29 ± 0.02
Wbc	0.93 ± 0.04	*0.95 ± 0.03*	0.84 ± 0.05	0.91 ± 0.06	*0.95 ± 0.03*	*0.95 ± 0.03*	*0.95 ± 0.04*	0.91 ± 0.03	0.92 ± 0.04	0.80 ± 0.07
Wdbc	0.86 ± 0.08	0.85 ± 0.09	0.86 ± 0.08	0.91 ± 0.04	0.00 ± 0.00	0.00 ± 0.00	0.00 ± 0.00	*0.93 ± 0.04*	0.91 ± 0.05	0.78 ± 0.07
Weathernominal	0.40 ± 0.70	0.40 ± 0.52	0.30 ± 0.67	0.40 ± 0.70	0.50 ± 0.53	0.40 ± 0.52	*0.60 ± 0.52*	*0.60 ± 0.52*	*0.60 ± 0.52*	0.20 ± 0.63
Weathernumeric	0.30 ± 0.67	0.50 ± 0.53	0.50 ± 0.71	*0.70 ± 0.48*	0.50 ± 0.53	0.20 ± 0.79	0.20 ± 0.79	*0.70 ± 0.48*	*0.70 ± 0.48*	0.20 ± 0.63
Website-phishingdata	0.67 ± 0.04	0.71 ± 0.04	*0.84 ± 0.04*	0.79 ± 0.05	0.73 ± 0.02	0.74 ± 0.02	0.78 ± 0.04	0.80 ± 0.04	0.82 ± 0.04	0.66 ± 0.03
Wholesalecustomersdata	0.76 ± 0.05	0.75 ± 0.09	0.78 ± 0.06	0.72 ± 0.11	0.00 ± 0.00	0.00 ± 0.00	0.00 ± 0.00	*0.81 ± 0.07*	0.79 ± 0.09	0.78 ± 0.08
Wifi-localization	0.90 ± 0.02	*0.98 ± 0.01*	0.96 ± 0.01	*0.98 ± 0.01*	0.72 ± 0.03	0.74 ± 0.03	0.74 ± 0.03	0.97 ± 0.01	0.97 ± 0.01	0.72 ± 0.06
Wilt	0.00 ± 0.00	0.30 ± 0.08	*0.82 ± 0.05*	0.42 ± 0.07	0.06 ± 0.04	0.06 ± 0.04	0.06 ± 0.04	0.75 ± 0.13	0.79 ± 0.08	0.01 ± 0.03
Wine-quality-red	0.36 ± 0.06	0.31 ± 0.06	0.39 ± 0.05	*0.45 ± 0.04*	0.39 ± 0.04	0.39 ± 0.04	0.40 ± 0.04	0.36 ± 0.06	0.36 ± 0.04	0.24 ± 0.05
Wine-quality-white	0.27 ± 0.04	0.22 ± 0.02	0.38 ± 0.04	*0.49 ± 0.04*	0.30 ± 0.04	0.32 ± 0.04	0.33 ± 0.04	0.28 ± 0.04	0.27 ± 0.03	0.13 ± 0.03
Wine	0.94 ± 0.09	0.95 ± 0.08	0.91 ± 0.08	0.92 ± 0.06	*0.98 ± 0.04*	0.97 ± 0.04	0.97 ± 0.04	0.96 ± 0.06	0.96 ± 0.06	0.65 ± 0.14
Yamilnaduelectricty	0.15 ± 0.01	0.00 ± 0.00	0.09 ± 0.01	*0.17 ± 0.01*	0.00 ± 0.01	0.00 ± 0.01	0.00 ± 0.01	0.00 ± 0.01	0.00 ± 0.01	0.01 ± 0.01
Yeast	0.27 ± 0.04	0.45 ± 0.03	0.43 ± 0.06	0.38 ± 0.03	*0.48 ± 0.06*	*0.48 ± 0.06*	*0.48 ± 0.06*	0.47 ± 0.05	0.46 ± 0.04	0.18 ± 0.05
Youtobe-kabita-preprocessing	0.22 ± 0.03	0.14 ± 0.01	0.23 ± 0.02	0.21 ± 0.02	*0.27 ± 0.03*	0.26 ± 0.03	0.25 ± 0.02	0.22 ± 0.01	0.23 ± 0.02	0.21 ± 0.02
Youtobe-nisha-preprocessing	0.27 ± 0.02	0.22 ± 0.02	0.28 ± 0.01	0.25 ± 0.02	*0.31 ± 0.02*	0.29 ± 0.02	0.29 ± 0.03	0.27 ± 0.02	0.26 ± 0.03	0.20 ± 0.02
Z-alizadehsani	0.00 ± 0.00	0.54 ± 0.15	0.47 ± 0.21	0.50 ± 0.13	0.00 ± 0.00	0.00 ± 0.00	0.00 ± 0.00	*0.57 ± 0.18*	0.56 ± 0.17	0.30 ± 0.21
Zoo	0.88 ± 0.12	*0.95 ± 0.08*	0.90 ± 0.12	*0.95 ± 0.08*	0.92 ± 0.11	0.92 ± 0.11	0.92 ± 0.11	0.94 ± 0.09	0.94 ± 0.09	0.65 ± 0.14
Average k (rank)	0.4171 (9)	0.5010 (5)	0.5910 (2)	0.5595 (4)	0.4182 (8)	0.4412 (7)	0.4528 (6)	0.5920 (1)	0.5898 (3)	0.4004 (10)
Average k std (rank)	0.0828 (4)	0.1118 (7)	0.1067 (6)	0.1151 (9)	0.0694 (1)	0.0798 (2)	0.0808 (3)	0.1150 (8)	0.1157 (10)	0.1061 (5)

**Table 8:** The detailed experimental results on root mean squared error and standard deviation.

Dataset	RN	NB	J48	KNN	SVM1	SVM2	SVM3	ANN1	ANN2	OneR
Abalone	0.41 ± 0.01	0.46 ± 0.02	0.45 ± 0.01	0.53 ± 0.02	0.47 ± 0.02	0.47 ± 0.02	0.47 ± 0.02	0.39 ± 0.01	0.39 ± 0.01	0.51 ± 0.01
Absenteeism-at-work	0.32 ± 0.01	0.33 ± 0.04	0.03 ± 0.05	0.40 ± 0.04	0.39 ± 0.04	0.39 ± 0.04	0.39 ± 0.04	0.14 ± 0.03	0.15 ± 0.04	0.21 ± 0.04
Acute-inflammatioactn	0.25 ± 0.02	0.15 ± 0.03	0.00 ± 0.00	0.01 ± 0.00	0.00 ± 0.00	0.00 ± 0.00	0.00 ± 0.00	0.01 ± 0.00	0.00 ± 0.00	0.45 ± 0.10
Acute-nephritis	0.22 ± 0.01	0.14 ± 0.13	0.00 ± 0.00	0.01 ± 0.00	0.00 ± 0.00	0.00 ± 0.00	0.00 ± 0.00	0.01 ± 0.00	0.00 ± 0.00	0.22 ± 0.20
Adult	0.40 ± 0.00	0.40 ± 0.03	0.38 ± 0.03	0.46 ± 0.04	0.41 ± 0.05	0.40 ± 0.05	0.41 ± 0.05	0.38 ± 0.03	0.39 ± 0.03	0.44 ± 0.01
Aggregation	0.14 ± 0.02	0.02 ± 0.01	0.02 ± 0.03	0.01 ± 0.02	0.01 ± 0.02	0.01 ± 0.03	0.01 ± 0.03	0.06 ± 0.02	0.05 ± 0.02	0.34 ± 0.01
Algerianforest	0.33 ± 0.03	0.19 ± 0.14	0.16 ± 0.13	0.33 ± 0.10	0.64 ± 0.01	0.62 ± 0.04	0.62 ± 0.04	0.14 ± 0.10	0.12 ± 0.11	0.09 ± 0.15
Annealing	0.26 ± 0.00	0.31 ± 0.03	0.11 ± 0.04	0.20 ± 0.02	0.21 ± 0.03	0.18 ± 0.03	0.18 ± 0.02	0.17 ± 0.04	0.16 ± 0.04	0.26 ± 0.00
Arrhythmia	0.22 ± 0.00	0.24 ± 0.02	0.22 ± 0.02	0.26 ± 0.01	0.24 ± 0.01	0.23 ± 0.01	0.23 ± 0.01	0.20 ± 0.02	0.23 ± 0.03	0.25 ± 0.01
Au1-1000	0.43 ± 0.00	0.43 ± 0.01	0.43 ± 0.04	0.51 ± 0.04	0.51 ± 0.00	0.51 ± 0.00	0.51 ± 0.01	0.53 ± 0.03	0.51 ± 0.04	0.51 ± 0.00
Au4-2500	0.42 ± 0.00	0.43 ± 0.01	0.41 ± 0.04	0.49 ± 0.03	0.51 ± 0.02	0.51 ± 0.02	0.51 ± 0.02	0.44 ± 0.03	0.44 ± 0.03	0.56 ± 0.03
Au6-1000	0.33 ± 0.00	0.34 ± 0.00	0.43 ± 0.01	0.46 ± 0.01	0.44 ± 0.00	0.44 ± 0.00	0.44 ± 0.00	0.43 ± 0.01	0.43 ± 0.01	0.43 ± 0.01
Au6-250-drift-au6-cd1-500	0.33 ± 0.00	0.34 ± 0.01	0.43 ± 0.01	0.45 ± 0.01	0.44 ± 0.00	0.44 ± 0.00	0.44 ± 0.00	0.42 ± 0.01	0.42 ± 0.01	0.43 ± 0.01
Au6-cd1-400	0.32 ± 0.00	0.34 ± 0.01	0.37 ± 0.02	0.45 ± 0.02	0.42 ± 0.00	0.42 ± 0.00	0.42 ± 0.00	0.40 ± 0.02	0.40 ± 0.01	0.41 ± 0.02
Au7-300-drift-au7-cpd1-800	0.39 ± 0.00	0.39 ± 0.01	0.46 ± 0.02	0.52 ± 0.02	0.54 ± 0.01	0.54 ± 0.01	0.54 ± 0.01	0.42 ± 0.01	0.42 ± 0.01	0.55 ± 0.01
Au7-700	0.36 ± 0.00	0.36 ± 0.01	0.41 ± 0.02	0.51 ± 0.02	0.50 ± 0.01	0.50 ± 0.01	0.50 ± 0.01	0.41 ± 0.01	0.41 ± 0.01	0.47 ± 0.02
Au7-cpd1-500	0.38 ± 0.00	0.38 ± 0.01	0.40 ± 0.02	0.50 ± 0.02	0.50 ± 0.00	0.50 ± 0.01	0.50 ± 0.01	0.45 ± 0.02	0.45 ± 0.03	0.49 ± 0.02
Audiology-std	0.21 ± 0.00	0.16 ± 0.03	0.15 ± 0.03	0.16 ± 0.04	0.20 ± 0.02	0.19 ± 0.03	0.18 ± 0.03	0.12 ± 0.02	0.13 ± 0.02	0.24 ± 0.00
Audit-risk	0.30 ± 0.02	0.22 ± 0.05	0.01 ± 0.04	0.14 ± 0.06	0.10 ± 0.08	0.09 ± 0.08	0.07 ± 0.08	0.15 ± 0.05	0.16 ± 0.05	0.00 ± 0.00
Autism-adolescent-data	0.39 ± 0.03	0.11 ± 0.07	0.00 ± 0.00	0.27 ± 0.15	0.12 ± 0.16	0.15 ± 0.16	0.12 ± 0.16	0.23 ± 0.18	0.23 ± 0.18	0.00 ± 0.00
Autism-adult-data	0.34 ± 0.01	0.12 ± 0.06	0.00 ± 0.00	0.21 ± 0.08	0.05 ± 0.07	0.03 ± 0.06	0.04 ± 0.07	0.01 ± 0.00	0.01 ± 0.00	0.00 ± 0.00
Autism-child-data	0.41 ± 0.01	0.10 ± 0.04	0.00 ± 0.00	0.33 ± 0.06	0.02 ± 0.06	0.00 ± 0.00	0.00 ± 0.00	0.03 ± 0.04	0.04 ± 0.04	0.00 ± 0.00
Autos	0.26 ± 0.01	0.31 ± 0.05	0.20 ± 0.04	0.25 ± 0.06	0.43 ± 0.01	0.43 ± 0.02	0.43 ± 0.02	0.23 ± 0.03	0.23 ± 0.03	0.33 ± 0.04
Avila	0.14 ± 0.00	0.29 ± 0.00	0.09 ± 0.01	0.20 ± 0.01	0.23 ± 0.01	0.22 ± 0.01	0.22 ± 0.01	0.21 ± 0.00	0.21 ± 0.01	0.22 ± 0.01
Balance-scale	0.34 ± 0.01	0.28 ± 0.01	0.37 ± 0.03	0.33 ± 0.04	0.25 ± 0.02	0.22 ± 0.01	0.21 ± 0.02	0.20 ± 0.03	0.21 ± 0.03	0.54 ± 0.03
Balloons	0.42 ± 0.11	0.50 ± 0.03	0.51 ± 0.19	0.37 ± 0.10	0.38 ± 0.41	0.24 ± 0.40	0.24 ± 0.40	0.38 ± 0.33	0.37 ± 0.34	0.65 ± 0.37
Bank	0.31 ± 0.00	0.35 ± 0.01	0.31 ± 0.01	0.37 ± 0.02	0.32 ± 0.01	0.32 ± 0.01	0.32 ± 0.02	0.30 ± 0.01	0.30 ± 0.02	0.34 ± 0.01
Blood	0.42 ± 0.01	0.43 ± 0.02	0.40 ± 0.02	0.53 ± 0.04	0.46 ± 0.02	0.46 ± 0.02	0.46 ± 0.02	0.39 ± 0.01	0.39 ± 0.01	0.49 ± 0.02
Breast-cancer-wisc-diag	0.28 ± 0.02	0.24 ± 0.11	0.24 ± 0.10	0.20 ± 0.05	0.13 ± 0.08	0.12 ± 0.08	0.12 ± 0.07	0.17 ± 0.05	0.17 ± 0.08	0.32 ± 0.06
Breast-cancer-wisc-prog	0.42 ± 0.02	0.52 ± 0.10	0.47 ± 0.08	0.51 ± 0.10	0.45 ± 0.07	0.46 ± 0.10	0.46 ± 0.10	0.46 ± 0.09	0.47 ± 0.10	0.54 ± 0.07
Breast-cancer-wisc	0.20 ± 0.04	0.20 ± 0.05	0.23 ± 0.07	0.19 ± 0.09	0.15 ± 0.08	0.15 ± 0.09	0.17 ± 0.07	0.19 ± 0.10	0.19 ± 0.07	0.26 ± 0.09
Breast-cancer	0.43 ± 0.02	0.45 ± 0.05	0.46 ± 0.03	0.54 ± 0.07	0.51 ± 0.05	0.52 ± 0.06	0.53 ± 0.05	0.48 ± 0.06	0.51 ± 0.04	0.54 ± 0.05
Breast-tissue	0.29 ± 0.03	0.29 ± 0.08	0.30 ± 0.09	0.28 ± 0.10	0.37 ± 0.08	0.32 ± 0.09	0.29 ± 0.13	0.28 ± 0.04	0.29 ± 0.05	0.39 ± 0.04
Bupa	0.47 ± 0.01	0.51 ± 0.04	0.54 ± 0.06	0.62 ± 0.04	0.64 ± 0.02	0.64 ± 0.02	0.64 ± 0.02	0.47 ± 0.04	0.47 ± 0.04	0.67 ± 0.06
Caesarian	0.46 ± 0.05	0.47 ± 0.10	0.49 ± 0.05	0.58 ± 0.12	0.67 ± 0.05	0.61 ± 0.09	0.63 ± 0.12	0.60 ± 0.15	0.60 ± 0.13	0.70 ± 0.15
Car	0.32 ± 0.00	0.23 ± 0.01	0.10 ± 0.03	0.12 ± 0.02	0.11 ± 0.03	0.09 ± 0.03	0.09 ± 0.03	0.15 ± 0.02	0.15 ± 0.02	0.39 ± 0.00
Cardiotocography-10classes	0.24 ± 0.00	0.22 ± 0.01	0.17 ± 0.01	0.21 ± 0.01	0.19 ± 0.01	0.19 ± 0.01	0.18 ± 0.01	0.17 ± 0.01	0.18 ± 0.01	0.32 ± 0.01
Cardiotocography-3classes	0.31 ± 0.00	0.33 ± 0.03	0.21 ± 0.04	0.23 ± 0.03	0.24 ± 0.03	0.23 ± 0.03	0.23 ± 0.03	0.21 ± 0.02	0.20 ± 0.03	0.35 ± 0.03
Cervical-cancer	0.38 ± 0.03	0.23 ± 0.17	0.30 ± 0.22	0.26 ± 0.22	0.54 ± 0.03	0.51 ± 0.07	0.51 ± 0.07	0.16 ± 0.16	0.16 ± 0.16	0.46 ± 0.10
Chemicalcomposionofceramic	0.25 ± 0.02	0.00 ± 0.00	0.04 ± 0.11	0.01 ± 0.00	0.74 ± 0.02	0.74 ± 0.02	0.74 ± 0.02	0.01 ± 0.01	0.01 ± 0.00	0.00 ± 0.00
Chess-krvk	0.22 ± 0.00	0.21 ± 0.00	0.21 ± 0.01	0.23 ± 0.01	0.24 ± 0.01	0.24 ± 0.01	0.24 ± 0.01	0.20 ± 0.00	0.20 ± 0.00	0.29 ± 0.00
Chess-krvkp	0.47 ± 0.00	0.32 ± 0.02	0.07 ± 0.04	0.23 ± 0.02	0.11 ± 0.04	0.08 ± 0.04	0.09 ± 0.03	0.07 ± 0.03	0.07 ± 0.04	0.58 ± 0.01
Congressional-voting	0.48 ± 0.00	0.48 ± 0.04	0.49 ± 0.02	0.50 ± 0.05	0.62 ± 0.03	0.62 ± 0.04	0.63 ± 0.05	0.49 ± 0.04	0.49 ± 0.04	0.61 ± 0.02
Conn-bench-sonar-mines-rocks	0.46 ± 0.01	0.53 ± 0.07	0.52 ± 0.06	0.35 ± 0.10	0.39 ± 0.10	0.33 ± 0.14	0.31 ± 0.14	0.38 ± 0.14	0.36 ± 0.14	0.60 ± 0.09
Conn-bench-vowel-deterding	0.22 ± 0.00	0.20 ± 0.01	0.18 ± 0.03	0.02 ± 0.03	0.10 ± 0.02	0.06 ± 0.03	0.04 ± 0.04	0.15 ± 0.02	0.16 ± 0.02	0.34 ± 0.01
Connect-4	0.43 ± 0.00	0.48 ± 0.01	0.41 ± 0.02	0.53 ± 0.03	0.51 ± 0.00	0.49 ± 0.01	0.48 ± 0.02	0.47 ± 0.02	0.47 ± 0.02	0.51 ± 0.00
Connectionist	0.46 ± 0.01	0.53 ± 0.10	0.49 ± 0.07	0.35 ± 0.08	0.62 ± 0.07	0.54 ± 0.13	0.49 ± 0.19	0.36 ± 0.11	0.36 ± 0.11	0.61 ± 0.06
Contrac	0.45 ± 0.00	0.46 ± 0.02	0.47 ± 0.02	0.61 ± 0.02	0.55 ± 0.01	0.55 ± 0.02	0.55 ± 0.02	0.43 ± 0.01	0.43 ± 0.01	0.59 ± 0.01
Covid-19	0.38 ± 0.09	0.35 ± 0.19	0.33 ± 0.31	0.40 ± 0.27	0.39 ± 0.35	0.42 ± 0.37	0.34 ± 0.37	0.31 ± 0.29	0.31 ± 0.30	0.20 ± 0.32
Credit-approval	0.42 ± 0.01	0.43 ± 0.04	0.36 ± 0.04	0.42 ± 0.07	0.38 ± 0.07	0.38 ± 0.06	0.39 ± 0.06	0.35 ± 0.04	0.37 ± 0.04	0.38 ± 0.06
Crowdsource	0.22 ± 0.00	0.22 ± 0.02	0.19 ± 0.01	0.12 ± 0.01	0.29 ± 0.01	0.29 ± 0.01	0.29 ± 0.01	0.15 ± 0.02	0.15 ± 0.02	0.27 ± 0.01
Crx	0.42 ± 0.01	0.43 ± 0.03	0.33 ± 0.04	0.43 ± 0.06	0.66 ± 0.03	0.66 ± 0.03	0.65 ± 0.04	0.38 ± 0.05	0.37 ± 0.03	0.38 ± 0.06
Cryother	0.34 ± 0.05	0.31 ± 0.16	0.20 ± 0.15	0.26 ± 0.18	0.39 ± 0.18	0.37 ± 0.18	0.37 ± 0.18	0.28 ± 0.13	0.32 ± 0.12	0.42 ± 0.12
Cylinder-bands	0.46 ± 0.01	0.54 ± 0.06	0.50 ± 0.06	0.55 ± 0.06	0.49 ± 0.04	0.46 ± 0.05	0.43 ± 0.04	0.48 ± 0.05	0.48 ± 0.04	0.58 ± 0.04
Dbworld-bodies	0.49 ± 0.01	0.46 ± 0.20	0.36 ± 0.25	0.62 ± 0.08	0.67 ± 0.04	0.67 ± 0.04	0.59 ± 0.11	/	/	0.28 ± 0.25
Dbworld-bodies-stemmed	0.49 ± 0.01	0.45 ± 0.18	0.28 ± 0.22	0.58 ± 0.12	0.67 ± 0.04	0.64 ± 0.07	0.50 ± 0.12	/	/	0.44 ± 0.27
Dbworld-subjects	0.49 ± 0.01	0.28 ± 0.15	0.44 ± 0.10	0.42 ± 0.15	0.67 ± 0.04	0.67 ± 0.04	0.67 ± 0.04	0.25 ± 0.20	0.25 ± 0.23	0.60 ± 0.10
Dbworld-subjects-stemmed	0.49 ± 0.01	0.27 ± 0.16	0.39 ± 0.17	0.37 ± 0.14	0.67 ± 0.04	0.67 ± 0.04	0.67 ± 0.04	0.26 ± 0.20	0.27 ± 0.20	0.62 ± 0.08
Dermatology	0.27 ± 0.00	0.07 ± 0.05	0.09 ± 0.06	0.10 ± 0.07	0.07 ± 0.06	0.06 ± 0.06	0.06 ± 0.07	0.07 ± 0.03	0.07 ± 0.03	0.41 ± 0.01
Diabetes	0.43 ± 0.02	0.41 ± 0.04	0.44 ± 0.05	0.54 ± 0.04	0.59 ± 0.00	0.59 ± 0.00	0.59 ± 0.00	0.42 ± 0.03	0.42 ± 0.04	0.53 ± 0.05
Diabetic	0.47 ± 0.01	0.65 ± 0.02	0.51 ± 0.03	0.62 ± 0.04	0.65 ± 0.04	0.64 ± 0.05	0.63 ± 0.05	0.43 ± 0.04	0.43 ± 0.04	0.68 ± 0.04
Divorce	0.15 ± 0.06	0.10 ± 0.13	0.17 ± 0.14	0.09 ± 0.11	0.10 ± 0.13	0.10 ± 0.13	0.10 ± 0.13	0.08 ± 0.10	0.08 ± 0.11	0.16 ± 0.15
Dota2train	0.50 ± 0.00	0.52 ± 0.02	0.60 ± 0.04	0.62 ± 0.03	0.69 ± 0.00	0.68 ± 0.03	0.69 ± 0.02	0.65 ± 0.07	0.64 ± 0.08	0.67 ± 0.03
Dow-jones-index	0.47 ± 0.01	0.55 ± 0.03	0.46 ± 0.03	0.67 ± 0.05	0.69 ± 0.00	0.69 ± 0.00	0.69 ± 0.00	0.53 ± 0.03	0.54 ± 0.03	0.66 ± 0.02
Dry-bean-dataset	0.14 ± 0.00	0.16 ± 0.01	0.13 ± 0.01	0.12 ± 0.01	0.33 ± 0.01	0.33 ± 0.01	0.33 ± 0.01	0.13 ± 0.01	0.13 ± 0.01	0.30 ± 0.01
Early-stage-diabetes-data-upload	0.39 ± 0.01	0.32 ± 0.05	0.19 ± 0.06	0.11 ± 0.09	0.22 ± 0.11	0.20 ± 0.11	0.18 ± 0.12	0.15 ± 0.07	0.16 ± 0.08	0.42 ± 0.05
Echocardiogram	0.43 ± 0.03	0.40 ± 0.15	0.42 ± 0.11	0.49 ± 0.11	0.40 ± 0.15	0.41 ± 0.17	0.41 ± 0.17	0.42 ± 0.11	0.42 ± 0.12	0.37 ± 0.09
Ecoli	0.24 ± 0.01	0.16 ± 0.03	0.18 ± 0.05	0.22 ± 0.04	0.18 ± 0.03	0.18 ± 0.03	0.18 ± 0.03	0.17 ± 0.02	0.18 ± 0.02	0.29 ± 0.03
Eegeyesate	0.46 ± 0.00	0.70 ± 0.06	0.38 ± 0.01	0.40 ± 0.03	0.67 ± 0.00	0.67 ± 0.00	0.67 ± 0.00	0.50 ± 0.01	0.50 ± 0.02	0.61 ± 0.01
Electrical	0.35 ± 0.04	0.14 ± 0.07	0.00 ± 0.00	0.27 ± 0.12	0.41 ± 0.09	0.41 ± 0.08	0.40 ± 0.08	0.08 ± 0.07	0.08 ± 0.07	0.02 ± 0.06
Energy-y1	0.31 ± 0.00	0.30 ± 0.02	0.12 ± 0.03	0.40 ± 0.03	0.27 ± 0.02	0.26 ± 0.02	0.26 ± 0.04	0.24 ± 0.01	0.25 ± 0.02	0.32 ± 0.02
Energy-y2	0.29 ± 0.01	0.27 ± 0.03	0.22 ± 0.03	0.40 ± 0.03	0.26 ± 0.03	0.25 ± 0.02	0.24 ± 0.02	0.21 ± 0.02	0.21 ± 0.03	0.28 ± 0.04
Extentionofz-alizadehsani	0.42 ± 0.01	0.23 ± 0.07	0.02 ± 0.06	0.30 ± 0.08	0.54 ± 0.01	0.54 ± 0.01	0.54 ± 0.01	0.09 ± 0.09	0.09 ± 0.09	0.36 ± 0.07
Fertility	0.32 ± 0.05	0.33 ± 0.05	0.36 ± 0.07	0.38 ± 0.17	0.34 ± 0.06	0.31 ± 0.12	0.29 ± 0.17	0.29 ± 0.13	0.30 ± 0.15	0.34 ± 0.06
First-order	0.29 ± 0.00	0.82 ± 0.02	0.00 ± 0.00	0.06 ± 0.05	0.31 ± 0.01	0.21 ± 0.04	0.13 ± 0.06	0.02 ± 0.03	0.02 ± 0.03	0.30 ± 0.03
Flags	0.29 ± 0.01	0.33 ± 0.02	0.30 ± 0.04	0.36 ± 0.03	0.34 ± 0.03	0.34 ± 0.04	0.34 ± 0.05	0.33 ± 0.04	0.32 ± 0.03	0.33 ± 0.03
Foresttypes	0.28 ± 0.01	0.13 ± 0.09	0.12 ± 0.10	0.13 ± 0.08	0.59 ± 0.01	0.58 ± 0.02	0.58 ± 0.02	0.10 ± 0.07	0.10 ± 0.07	0.29 ± 0.07
Garments-worker-productivity	0.37 ± 0.00	0.41 ± 0.01	0.41 ± 0.02	0.54 ± 0.01	0.51 ± 0.01	0.51 ± 0.01	0.50 ± 0.01	0.39 ± 0.01	0.39 ± 0.01	0.52 ± 0.01
Gender-name-dataset	0.49 ± 0.00	0.61 ± 0.01	0.49 ± 0.00	0.48 ± 0.01	0.60 ± 0.01	0.60 ± 0.02	0.60 ± 0.02	0.49 ± 0.00	0.49 ± 0.00	0.63 ± 0.01
Gesture-a1-raw	0.20 ± 0.01	0.29 ± 0.02	0.18 ± 0.02	0.12 ± 0.02	0.49 ± 0.00	0.49 ± 0.00	0.49 ± 0.00	0.17 ± 0.02	0.17 ± 0.01	0.11 ± 0.03
Gesture-a1-va3	0.31 ± 0.01	0.38 ± 0.02	0.35 ± 0.03	0.32 ± 0.02	0.49 ± 0.00	0.49 ± 0.00	0.49 ± 0.00	0.30 ± 0.01	0.31 ± 0.02	0.39 ± 0.01
Gesture-a2-raw	0.21 ± 0.00	0.33 ± 0.02	0.20 ± 0.03	0.14 ± 0.05	0.49 ± 0.00	0.49 ± 0.00	0.49 ± 0.00	0.18 ± 0.02	0.18 ± 0.02	0.14 ± 0.03
Gesture-a2-va3	0.33 ± 0.01	0.44 ± 0.04	0.40 ± 0.01	0.36 ± 0.02	0.49 ± 0.00	0.49 ± 0.00	0.49 ± 0.00	0.35 ± 0.02	0.35 ± 0.02	0.43 ± 0.02
Gesture-a3-raw	0.18 ± 0.01	0.35 ± 0.01	0.16 ± 0.01	0.12 ± 0.02	0.51 ± 0.00	0.51 ± 0.00	0.51 ± 0.00	0.19 ± 0.01	0.19 ± 0.02	0.14 ± 0.02
Gesture-a3-va3	0.33 ± 0.00	0.43 ± 0.01	0.30 ± 0.02	0.20 ± 0.02	0.51 ± 0.00	0.51 ± 0.00	0.51 ± 0.00	0.32 ± 0.01	0.32 ± 0.01	0.44 ± 0.01
Gesture-b1-raw	0.18 ± 0.01	0.38 ± 0.02	0.15 ± 0.03	0.13 ± 0.04	0.45 ± 0.00	0.45 ± 0.00	0.45 ± 0.00	0.18 ± 0.02	0.17 ± 0.01	0.17 ± 0.02
Gesture-b1-va3	0.33 ± 0.01	0.49 ± 0.03	0.34 ± 0.02	0.19 ± 0.03	0.50 ± 0.00	0.50 ± 0.00	0.50 ± 0.00	0.33 ± 0.01	0.33 ± 0.02	0.48 ± 0.02
Gesture-b3-raw	0.23 ± 0.01	0.36 ± 0.02	0.18 ± 0.03	0.13 ± 0.01	0.52 ± 0.00	0.52 ± 0.00	0.52 ± 0.00	0.20 ± 0.04	0.20 ± 0.03	0.13 ± 0.03
Gesture-b3-va3	0.37 ± 0.01	0.45 ± 0.02	0.40 ± 0.02	0.30 ± 0.02	0.52 ± 0.00	0.52 ± 0.00	0.52 ± 0.00	0.35 ± 0.02	0.37 ± 0.02	0.49 ± 0.02
Gesture-c1-raw	0.23 ± 0.01	0.34 ± 0.01	0.20 ± 0.02	0.15 ± 0.02	0.55 ± 0.00	0.55 ± 0.00	0.55 ± 0.00	0.20 ± 0.03	0.20 ± 0.03	0.15 ± 0.03
Gesture-c1-va3	0.35 ± 0.01	0.41 ± 0.02	0.39 ± 0.02	0.34 ± 0.02	0.55 ± 0.00	0.55 ± 0.00	0.55 ± 0.00	0.34 ± 0.02	0.35 ± 0.01	0.48 ± 0.02
Gesture-c3-raw	0.22 ± 0.01	0.36 ± 0.02	0.21 ± 0.03	0.16 ± 0.03	0.54 ± 0.00	0.54 ± 0.00	0.54 ± 0.00	0.21 ± 0.02	0.23 ± 0.01	0.15 ± 0.02
Gesture-c3-va3	0.36 ± 0.01	0.44 ± 0.01	0.40 ± 0.02	0.39 ± 0.02	0.54 ± 0.00	0.54 ± 0.00	0.54 ± 0.00	0.36 ± 0.02	0.37 ± 0.02	0.48 ± 0.01
Glass	0.29 ± 0.01	0.36 ± 0.03	0.31 ± 0.04	0.31 ± 0.05	0.30 ± 0.04	0.30 ± 0.04	0.31 ± 0.04	0.28 ± 0.03	0.28 ± 0.02	0.38 ± 0.04
Go-track-tracks	0.37 ± 0.04	0.38 ± 0.06	0.30 ± 0.14	0.24 ± 0.18	0.52 ± 0.11	0.52 ± 0.11	0.52 ± 0.11	0.32 ± 0.09	0.32 ± 0.09	0.48 ± 0.14
Haberman-survival	0.43 ± 0.01	0.43 ± 0.04	0.43 ± 0.02	0.57 ± 0.06	0.52 ± 0.05	0.54 ± 0.03	0.54 ± 0.03	0.43 ± 0.03	0.43 ± 0.03	0.52 ± 0.03
Hayes-roth	0.34 ± 0.02	0.36 ± 0.02	0.28 ± 0.05	0.21 ± 0.05	0.28 ± 0.11	0.35 ± 0.06	0.36 ± 0.06	0.34 ± 0.10	0.34 ± 0.10	0.61 ± 0.03
Hcc-data	0.46 ± 0.01	0.53 ± 0.07	0.54 ± 0.06	0.60 ± 0.07	0.62 ± 0.02	0.62 ± 0.02	0.62 ± 0.02	0.54 ± 0.11	0.56 ± 0.09	0.54 ± 0.12
Hcvdat	0.19 ± 0.01	0.17 ± 0.04	0.17 ± 0.03	0.19 ± 0.02	0.23 ± 0.01	0.23 ± 0.01	0.23 ± 0.01	0.14 ± 0.03	0.16 ± 0.03	0.21 ± 0.02
Heart-cleveland	0.33 ± 0.01	0.34 ± 0.04	0.40 ± 0.04	0.42 ± 0.03	0.41 ± 0.04	0.40 ± 0.05	0.41 ± 0.05	0.38 ± 0.03	0.38 ± 0.04	0.43 ± 0.03
Heart-hungarian	0.39 ± 0.03	0.39 ± 0.08	0.41 ± 0.07	0.46 ± 0.09	0.42 ± 0.09	0.41 ± 0.08	0.40 ± 0.09	0.42 ± 0.08	0.42 ± 0.06	0.45 ± 0.11
Heart-switzerland	0.37 ± 0.01	0.40 ± 0.03	0.45 ± 0.05	0.51 ± 0.05	0.49 ± 0.03	0.49 ± 0.02	0.48 ± 0.03	0.42 ± 0.05	0.42 ± 0.04	0.52 ± 0.05
Heart-va	0.39 ± 0.01	0.42 ± 0.03	0.48 ± 0.02	0.50 ± 0.03	0.51 ± 0.04	0.52 ± 0.03	0.53 ± 0.04	0.47 ± 0.03	0.48 ± 0.03	0.54 ± 0.04
Heart-failure-clinical-records-dataset	0.44 ± 0.01	0.41 ± 0.04	0.41 ± 0.06	0.58 ± 0.06	0.57 ± 0.01	0.57 ± 0.01	0.57 ± 0.01	0.48 ± 0.05	0.45 ± 0.09	0.37 ± 0.08
Hepatitis	0.38 ± 0.02	0.37 ± 0.10	0.41 ± 0.12	0.41 ± 0.07	0.40 ± 0.08	0.35 ± 0.19	0.35 ± 0.19	0.39 ± 0.11	0.42 ± 0.12	0.50 ± 0.07
Hill-valley	0.51 ± 0.01	0.70 ± 0.05	0.50 ± 0.00	0.69 ± 0.03	0.69 ± 0.05	0.69 ± 0.05	0.68 ± 0.05	0.48 ± 0.02	0.49 ± 0.02	0.72 ± 0.03
Hiv1625data	0.25 ± 0.02	0.22 ± 0.04	0.27 ± 0.03	0.27 ± 0.02	0.28 ± 0.04	0.25 ± 0.04	0.25 ± 0.05	0.20 ± 0.05	0.24 ± 0.05	0.45 ± 0.02
Hiv746data	0.30 ± 0.03	0.26 ± 0.05	0.37 ± 0.04	0.34 ± 0.04	0.32 ± 0.06	0.30 ± 0.05	0.29 ± 0.06	0.25 ± 0.05	0.26 ± 0.06	0.43 ± 0.06
Horse-colic	0.43 ± 0.01	0.47 ± 0.06	0.34 ± 0.08	0.48 ± 0.08	0.40 ± 0.07	0.39 ± 0.07	0.38 ± 0.08	0.44 ± 0.05	0.42 ± 0.10	0.42 ± 0.06
Htru	0.16 ± 0.01	0.23 ± 0.01	0.14 ± 0.02	0.17 ± 0.01	0.30 ± 0.00	0.29 ± 0.00	0.29 ± 0.00	0.13 ± 0.01	0.13 ± 0.01	0.15 ± 0.01
Hypothyroid	0.18 ± 0.00	0.14 ± 0.01	0.04 ± 0.02	0.21 ± 0.02	0.19 ± 0.00	0.19 ± 0.01	0.19 ± 0.01	0.15 ± 0.04	0.17 ± 0.04	0.14 ± 0.02
Ibeacon-rssi-labeled	0.09 ± 0.00	0.10 ± 0.00	0.09 ± 0.00	0.09 ± 0.00	0.11 ± 0.00	0.11 ± 0.00	0.11 ± 0.00	0.09 ± 0.00	0.09 ± 0.00	0.13 ± 0.00
Ilpd-indian-liver	0.43 ± 0.01	0.65 ± 0.04	0.47 ± 0.03	0.59 ± 0.05	0.54 ± 0.01	0.54 ± 0.02	0.54 ± 0.02	0.42 ± 0.01	0.42 ± 0.01	0.58 ± 0.03
Image-segmentation	0.23 ± 0.01	0.24 ± 0.03	0.16 ± 0.08	0.19 ± 0.04	0.19 ± 0.02	0.19 ± 0.04	0.19 ± 0.04	0.14 ± 0.03	0.15 ± 0.03	0.34 ± 0.04
Immunotherapy	0.38 ± 0.02	0.40 ± 0.08	0.37 ± 0.13	0.50 ± 0.21	0.46 ± 0.04	0.46 ± 0.04	0.46 ± 0.04	0.37 ± 0.10	0.39 ± 0.11	0.37 ± 0.16
Impensdata	0.30 ± 0.01	0.26 ± 0.03	0.36 ± 0.00	0.33 ± 0.03	0.40 ± 0.00	0.40 ± 0.00	0.40 ± 0.00	0.29 ± 0.03	0.31 ± 0.04	0.39 ± 0.03
In-vehicle-coupon-recommendation	0.48 ± 0.00	0.47 ± 0.01	0.46 ± 0.01	0.56 ± 0.01	0.56 ± 0.01	0.55 ± 0.01	0.55 ± 0.01	0.52 ± 0.01	0.51 ± 0.01	0.63 ± 0.01
Indian	0.43 ± 0.01	0.65 ± 0.04	0.48 ± 0.04	0.60 ± 0.06	0.53 ± 0.02	0.53 ± 0.01	0.53 ± 0.01	0.43 ± 0.02	0.43 ± 0.02	0.58 ± 0.03
Ionosphere	0.30 ± 0.02	0.39 ± 0.06	0.28 ± 0.06	0.36 ± 0.07	0.23 ± 0.05	0.21 ± 0.09	0.19 ± 0.09	0.29 ± 0.03	0.28 ± 0.05	0.42 ± 0.11
Iris	0.28 ± 0.04	0.14 ± 0.08	0.11 ± 0.12	0.13 ± 0.13	0.11 ± 0.11	0.11 ± 0.12	0.11 ± 0.11	0.10 ± 0.09	0.10 ± 0.09	0.19 ± 0.14
Jain	0.20 ± 0.06	0.18 ± 0.06	0.02 ± 0.07	0.00 ± 0.00	0.00 ± 0.00	0.00 ± 0.00	0.00 ± 0.00	0.18 ± 0.05	0.18 ± 0.05	0.19 ± 0.11
Jsbach-chorals-harmony	0.26 ± 0.00	0.16 ± 0.01	0.17 ± 0.01	0.14 ± 0.01	0.19 ± 0.02	0.18 ± 0.01	0.17 ± 0.02	0.15 ± 0.02	0.16 ± 0.03	0.20 ± 0.02
Knowledge	0.36 ± 0.02	0.25 ± 0.07	0.15 ± 0.10	0.34 ± 0.06	0.27 ± 0.09	0.20 ± 0.13	0.16 ± 0.13	0.17 ± 0.08	0.17 ± 0.07	0.29 ± 0.08
Lasvegastripadvisorreviews	0.35 ± 0.00	0.39 ± 0.01	0.42 ± 0.01	0.49 ± 0.01	0.50 ± 0.01	0.50 ± 0.01	0.50 ± 0.01	0.45 ± 0.01	0.45 ± 0.02	0.49 ± 0.01
Leaf	0.15 ± 0.00	0.12 ± 0.02	0.15 ± 0.02	0.24 ± 0.00	0.24 ± 0.01	0.22 ± 0.01	0.22 ± 0.01	0.15 ± 0.01	0.16 ± 0.01	0.23 ± 0.01
Led-display	0.26 ± 0.00	0.20 ± 0.01	0.21 ± 0.01	0.21 ± 0.01	0.24 ± 0.02	0.24 ± 0.02	0.24 ± 0.02	0.20 ± 0.01	0.21 ± 0.01	0.40 ± 0.00
Lenses	0.33 ± 0.10	0.42 ± 0.12	0.23 ± 0.26	0.19 ± 0.18	0.24 ± 0.31	0.21 ± 0.27	0.21 ± 0.27	0.29 ± 0.24	0.31 ± 0.25	0.39 ± 0.29
Letter	0.17 ± 0.00	0.14 ± 0.00	0.09 ± 0.00	0.06 ± 0.00	0.06 ± 0.00	0.06 ± 0.00	0.05 ± 0.00	0.11 ± 0.00	0.11 ± 0.00	0.25 ± 0.00
Libras	0.21 ± 0.01	0.21 ± 0.03	0.19 ± 0.03	0.13 ± 0.03	0.16 ± 0.02	0.14 ± 0.02	0.13 ± 0.02	0.15 ± 0.02	0.14 ± 0.02	0.32 ± 0.01
Low-res-spect	0.21 ± 0.00	0.21 ± 0.02	0.19 ± 0.02	0.19 ± 0.02	0.15 ± 0.02	0.15 ± 0.02	0.14 ± 0.02	0.12 ± 0.02	0.13 ± 0.02	0.24 ± 0.02
Lung-cancer	0.44 ± 0.02	0.45 ± 0.14	0.55 ± 0.14	0.48 ± 0.27	0.52 ± 0.20	0.49 ± 0.20	0.53 ± 0.21	0.54 ± 0.11	0.54 ± 0.12	0.58 ± 0.10
Lymphography	0.32 ± 0.01	0.26 ± 0.06	0.33 ± 0.07	0.32 ± 0.13	0.24 ± 0.11	0.26 ± 0.08	0.28 ± 0.07	0.27 ± 0.06	0.27 ± 0.07	0.34 ± 0.06
Magic	0.40 ± 0.01	0.49 ± 0.02	0.38 ± 0.02	0.45 ± 0.03	0.39 ± 0.03	0.39 ± 0.02	0.39 ± 0.02	0.34 ± 0.03	0.36 ± 0.03	0.53 ± 0.04
Mammographic	0.40 ± 0.01	0.41 ± 0.04	0.36 ± 0.02	0.47 ± 0.05	0.41 ± 0.03	0.42 ± 0.03	0.42 ± 0.03	0.37 ± 0.02	0.38 ± 0.03	0.43 ± 0.01
Miniboone	0.36 ± 0.01	0.84 ± 0.01	0.36 ± 0.03	0.40 ± 0.02	0.39 ± 0.03	0.38 ± 0.03	0.37 ± 0.03	0.34 ± 0.02	0.34 ± 0.02	0.43 ± 0.02
Molec-biol-promoter	0.45 ± 0.02	0.27 ± 0.14	0.48 ± 0.10	0.51 ± 0.12	0.40 ± 0.14	0.36 ± 0.17	0.36 ± 0.17	0.42 ± 0.10	0.43 ± 0.10	0.54 ± 0.10
Molec-biol-splice	0.44 ± 0.00	0.20 ± 0.02	0.21 ± 0.03	0.50 ± 0.01	0.29 ± 0.02	0.29 ± 0.02	0.30 ± 0.01	0.29 ± 0.02	0.30 ± 0.02	0.49 ± 0.02
Monks-1	0.40 ± 0.03	0.43 ± 0.08	0.09 ± 0.13	0.54 ± 0.10	0.39 ± 0.22	0.36 ± 0.21	0.38 ± 0.16	0.22 ± 0.22	0.18 ± 0.24	0.51 ± 0.10
Monks-2	0.49 ± 0.02	0.50 ± 0.03	0.44 ± 0.10	0.61 ± 0.09	0.65 ± 0.06	0.62 ± 0.11	0.59 ± 0.10	0.45 ± 0.10	0.44 ± 0.07	0.64 ± 0.04
Monks-3	0.39 ± 0.04	0.32 ± 0.06	0.20 ± 0.16	0.47 ± 0.15	0.26 ± 0.19	0.29 ± 0.17	0.29 ± 0.17	0.32 ± 0.16	0.28 ± 0.12	0.47 ± 0.04
Mushroom	0.34 ± 0.01	0.32 ± 0.02	0.00 ± 0.00	0.00 ± 0.00	0.00 ± 0.00	0.00 ± 0.00	0.00 ± 0.00	0.00 ± 0.00	0.00 ± 0.00	0.12 ± 0.02
Musk-1	0.41 ± 0.01	0.49 ± 0.07	0.37 ± 0.09	0.38 ± 0.06	0.30 ± 0.08	0.27 ± 0.06	0.24 ± 0.06	0.21 ± 0.05	0.21 ± 0.05	0.61 ± 0.07
Musk-2	0.29 ± 0.01	0.37 ± 0.02	0.20 ± 0.03	0.19 ± 0.02	0.20 ± 0.03	0.16 ± 0.03	0.14 ± 0.03	0.10 ± 0.04	0.10 ± 0.04	0.32 ± 0.02
Newdiagnosis	0.25 ± 0.02	0.15 ± 0.04	0.00 ± 0.00	0.00 ± 0.00	0.00 ± 0.00	0.00 ± 0.00	0.00 ± 0.00	0.01 ± 0.00	0.01 ± 0.00	0.47 ± 0.08
Nursery	0.33 ± 0.00	0.19 ± 0.01	0.04 ± 0.01	0.18 ± 0.01	0.08 ± 0.01	0.07 ± 0.01	0.07 ± 0.01	0.10 ± 0.02	0.12 ± 0.02	0.34 ± 0.01
Obesitydataset-raw-and-data-sinthetic	0.28 ± 0.00	0.25 ± 0.01	0.13 ± 0.02	0.23 ± 0.02	0.17 ± 0.02	0.15 ± 0.02	0.15 ± 0.02	0.12 ± 0.03	0.12 ± 0.01	0.31 ± 0.02
Obs-network-dataset-2-aug27	0.21 ± 0.01	0.36 ± 0.03	0.01 ± 0.03	0.06 ± 0.03	0.11 ± 0.02	0.11 ± 0.02	0.11 ± 0.02	0.11 ± 0.03	0.09 ± 0.03	0.21 ± 0.03
Occupancy-data	0.17 ± 0.02	0.20 ± 0.02	0.11 ± 0.03	0.09 ± 0.03	0.44 ± 0.02	0.42 ± 0.03	0.42 ± 0.03	0.13 ± 0.03	0.12 ± 0.03	0.11 ± 0.03
Occupancy-data2	0.14 ± 0.01	0.19 ± 0.01	0.07 ± 0.01	0.07 ± 0.01	0.41 ± 0.01	0.38 ± 0.01	0.38 ± 0.01	0.07 ± 0.01	0.07 ± 0.01	0.08 ± 0.01
Occupancy-data3	0.12 ± 0.01	0.14 ± 0.02	0.07 ± 0.01	0.07 ± 0.02	0.41 ± 0.01	0.39 ± 0.01	0.39 ± 0.01	0.09 ± 0.01	0.09 ± 0.01	0.08 ± 0.01
Old	0.42 ± 0.00	0.21 ± 0.02	0.20 ± 0.03	0.13 ± 0.03	0.22 ± 0.02	0.22 ± 0.03	0.21 ± 0.03	0.15 ± 0.03	0.15 ± 0.03	0.34 ± 0.02
Online-shoppers-intention	0.34 ± 0.00	0.39 ± 0.01	0.30 ± 0.01	0.43 ± 0.01	0.39 ± 0.00	0.39 ± 0.00	0.40 ± 0.00	0.28 ± 0.01	0.29 ± 0.02	0.34 ± 0.01
Oocytes-merluccius-nucleus-4d	0.43 ± 0.01	0.59 ± 0.04	0.44 ± 0.02	0.53 ± 0.04	0.48 ± 0.03	0.44 ± 0.03	0.43 ± 0.04	0.38 ± 0.03	0.39 ± 0.04	0.57 ± 0.04
Oocytes-merluccius-states-2f	0.25 ± 0.02	0.31 ± 0.04	0.24 ± 0.04	0.24 ± 0.04	0.23 ± 0.04	0.23 ± 0.03	0.22 ± 0.03	0.21 ± 0.02	0.22 ± 0.03	0.34 ± 0.04
Oocytes-trisopterus-nucleus-2f	0.46 ± 0.01	0.64 ± 0.04	0.49 ± 0.05	0.50 ± 0.06	0.43 ± 0.04	0.40 ± 0.04	0.41 ± 0.03	0.36 ± 0.04	0.39 ± 0.04	0.64 ± 0.05
Oocytes-trisopterus-states-5b	0.29 ± 0.03	0.39 ± 0.03	0.26 ± 0.04	0.24 ± 0.05	0.23 ± 0.05	0.22 ± 0.04	0.22 ± 0.04	0.19 ± 0.04	0.19 ± 0.04	0.36 ± 0.03
Optdigits	0.27 ± 0.00	0.13 ± 0.01	0.13 ± 0.01	0.05 ± 0.01	0.23 ± 0.01	0.22 ± 0.01	0.22 ± 0.01	0.05 ± 0.01	0.05 ± 0.01	0.38 ± 0.00
Optical	0.27 ± 0.00	0.12 ± 0.01	0.14 ± 0.01	0.05 ± 0.01	0.05 ± 0.01	0.05 ± 0.01	0.05 ± 0.01	0.05 ± 0.01	0.06 ± 0.01	0.38 ± 0.00
Ozone	0.17 ± 0.01	0.53 ± 0.03	0.20 ± 0.01	0.22 ± 0.02	0.17 ± 0.01	0.17 ± 0.01	0.17 ± 0.01	0.18 ± 0.02	0.17 ± 0.02	0.18 ± 0.01
Page-blocks	0.14 ± 0.01	0.19 ± 0.01	0.11 ± 0.01	0.13 ± 0.01	0.12 ± 0.01	0.12 ± 0.01	0.12 ± 0.01	0.11 ± 0.01	0.11 ± 0.01	0.16 ± 0.01
Parkingbirmingham	0.09 ± 0.01	0.10 ± 0.00	0.00 ± 0.00	0.04 ± 0.01	0.17 ± 0.01	0.16 ± 0.01	0.16 ± 0.01	0.08 ± 0.00	0.08 ± 0.00	0.00 ± 0.00
Parkinsons	0.34 ± 0.03	0.54 ± 0.11	0.42 ± 0.05	0.16 ± 0.11	0.34 ± 0.09	0.29 ± 0.13	0.27 ± 0.12	0.28 ± 0.11	0.26 ± 0.14	0.36 ± 0.11
Pasture	0.41 ± 0.04	0.34 ± 0.24	0.30 ± 0.23	0.33 ± 0.26	0.69 ± 0.02	0.69 ± 0.02	0.69 ± 0.02	0.30 ± 0.24	0.31 ± 0.25	0.43 ± 0.25
Pbc	0.43 ± 0.02	0.43 ± 0.07	0.41 ± 0.07	0.63 ± 0.07	0.62 ± 0.01	0.62 ± 0.01	0.62 ± 0.01	0.50 ± 0.05	0.48 ± 0.03	0.52 ± 0.09
Pen	0.22 ± 0.00	0.16 ± 0.01	0.08 ± 0.01	0.04 ± 0.00	0.42 ± 0.00	0.41 ± 0.00	0.41 ± 0.00	0.10 ± 0.01	0.10 ± 0.00	0.35 ± 0.00
Pendigits	0.22 ± 0.00	0.15 ± 0.01	0.09 ± 0.01	0.03 ± 0.01	0.03 ± 0.01	0.02 ± 0.01	0.02 ± 0.01	0.09 ± 0.01	0.09 ± 0.01	0.35 ± 0.01
Pharynx	0.43 ± 0.02	0.46 ± 0.03	0.42 ± 0.01	0.59 ± 0.09	0.49 ± 0.08	0.51 ± 0.10	0.54 ± 0.09	0.49 ± 0.04	0.51 ± 0.13	0.85 ± 0.03
Phishingwebsites	0.44 ± 0.00	0.23 ± 0.01	0.19 ± 0.01	0.14 ± 0.01	0.23 ± 0.01	0.22 ± 0.01	0.22 ± 0.01	0.16 ± 0.01	0.16 ± 0.01	0.33 ± 0.01
Pima	0.43 ± 0.02	0.42 ± 0.04	0.44 ± 0.05	0.54 ± 0.04	0.49 ± 0.04	0.50 ± 0.04	0.50 ± 0.03	0.42 ± 0.03	0.42 ± 0.04	0.53 ± 0.05
Pittsburg-bridges-rel-l	0.40 ± 0.03	0.40 ± 0.08	0.44 ± 0.08	0.40 ± 0.17	0.46 ± 0.10	0.45 ± 0.10	0.42 ± 0.10	0.45 ± 0.09	0.43 ± 0.09	0.43 ± 0.09
Pittsburg-bridges-span	0.41 ± 0.02	0.38 ± 0.07	0.43 ± 0.07	0.51 ± 0.11	0.47 ± 0.10	0.48 ± 0.12	0.48 ± 0.12	0.41 ± 0.05	0.41 ± 0.07	0.54 ± 0.08
Pittsburg-bridges-t-or-d	0.33 ± 0.06	0.30 ± 0.10	0.37 ± 0.13	0.37 ± 0.17	0.34 ± 0.14	0.30 ± 0.17	0.28 ± 0.16	0.29 ± 0.21	0.31 ± 0.22	0.34 ± 0.19
Pittsburg-bridges-type	0.33 ± 0.02	0.32 ± 0.04	0.33 ± 0.04	0.36 ± 0.06	0.39 ± 0.05	0.35 ± 0.08	0.35 ± 0.07	0.32 ± 0.07	0.34 ± 0.07	0.38 ± 0.02
Pittsburg-bridgesmaterial	0.30 ± 0.03	0.30 ± 0.07	0.25 ± 0.08	0.30 ± 0.13	0.30 ± 0.07	0.31 ± 0.06	0.30 ± 0.13	0.30 ± 0.08	0.32 ± 0.08	0.29 ± 0.05
Planning	0.45 ± 0.01	0.49 ± 0.04	0.45 ± 0.01	0.58 ± 0.08	0.53 ± 0.01	0.54 ± 0.05	0.58 ± 0.06	0.56 ± 0.06	0.60 ± 0.08	0.61 ± 0.07
Plant-margin	0.09 ± 0.00	0.05 ± 0.00	0.10 ± 0.00	0.07 ± 0.00	0.06 ± 0.00	0.06 ± 0.01	0.05 ± 0.01	0.05 ± 0.00	0.05 ± 0.00	0.14 ± 0.00
Plant-shape	0.09 ± 0.00	0.09 ± 0.00	0.10 ± 0.00	0.08 ± 0.00	0.10 ± 0.00	0.09 ± 0.00	0.09 ± 0.00	0.07 ± 0.00	0.07 ± 0.00	0.14 ± 0.00
Plant-texture	0.10 ± 0.00	0.07 ± 0.00	0.09 ± 0.00	0.06 ± 0.00	0.06 ± 0.00	0.05 ± 0.00	0.05 ± 0.00	0.05 ± 0.00	0.05 ± 0.00	0.14 ± 0.00
Poker-hand-training-true	0.24 ± 0.00	0.24 ± 0.00	0.27 ± 0.01	0.32 ± 0.00	0.29 ± 0.00	0.29 ± 0.00	0.30 ± 0.00	0.24 ± 0.00	0.24 ± 0.00	0.32 ± 0.00
Post-operative	0.38 ± 0.03	0.41 ± 0.04	0.38 ± 0.03	0.51 ± 0.06	0.44 ± 0.04	0.45 ± 0.05	0.47 ± 0.07	0.45 ± 0.08	0.48 ± 0.08	0.45 ± 0.05
Primary-tumor	0.23 ± 0.00	0.22 ± 0.02	0.24 ± 0.01	0.28 ± 0.02	0.27 ± 0.03	0.27 ± 0.03	0.27 ± 0.03	0.25 ± 0.02	0.25 ± 0.02	0.31 ± 0.01
Qsarbioconcentration	0.43 ± 0.00	0.44 ± 0.01	0.45 ± 0.04	0.62 ± 0.05	0.50 ± 0.01	0.52 ± 0.01	0.54 ± 0.03	0.47 ± 0.03	0.48 ± 0.02	0.52 ± 0.03
Qsarbiodegradation	0.42 ± 0.01	0.47 ± 0.03	0.38 ± 0.03	0.40 ± 0.03	0.37 ± 0.03	0.37 ± 0.03	0.38 ± 0.03	0.33 ± 0.02	0.33 ± 0.03	0.48 ± 0.04
Qualitative-bankruptcy	0.16 ± 0.02	0.03 ± 0.05	0.09 ± 0.11	0.01 ± 0.01	0.05 ± 0.10	0.05 ± 0.10	0.02 ± 0.06	0.02 ± 0.06	0.02 ± 0.06	0.07 ± 0.11
Ringnorm	0.37 ± 0.01	0.10 ± 0.01	0.28 ± 0.00	0.50 ± 0.02	0.12 ± 0.02	0.12 ± 0.02	0.13 ± 0.02	0.27 ± 0.02	0.27 ± 0.01	0.60 ± 0.01
Risk-factors-cervical-cancer	0.23 ± 0.01	0.31 ± 0.03	0.19 ± 0.04	0.23 ± 0.04	0.25 ± 0.01	0.26 ± 0.02	0.24 ± 0.03	0.20 ± 0.04	0.21 ± 0.04	0.19 ± 0.05
Robotnavigation	0.22 ± 0.01	0.44 ± 0.01	0.04 ± 0.01	0.24 ± 0.01	0.22 ± 0.01	0.21 ± 0.01	0.20 ± 0.01	0.23 ± 0.02	0.22 ± 0.02	0.35 ± 0.01
Sapfile	0.45 ± 0.01	0.46 ± 0.05	0.54 ± 0.07	0.60 ± 0.04	0.58 ± 0.05	0.58 ± 0.04	0.58 ± 0.05	0.54 ± 0.05	0.54 ± 0.07	0.62 ± 0.06
Sat	0.22 ± 0.00	0.26 ± 0.01	0.21 ± 0.01	0.18 ± 0.01	0.50 ± 0.00	0.49 ± 0.00	0.49 ± 0.00	0.17 ± 0.01	0.18 ± 0.01	0.37 ± 0.01
Satelite	0.22 ± 0.00	0.26 ± 0.01	0.21 ± 0.01	0.18 ± 0.01	0.50 ± 0.00	0.49 ± 0.00	0.49 ± 0.00	0.17 ± 0.00	0.18 ± 0.01	0.37 ± 0.01
Scadi	0.28 ± 0.01	0.20 ± 0.08	0.20 ± 0.08	0.21 ± 0.09	0.26 ± 0.05	0.23 ± 0.10	0.20 ± 0.12	0.22 ± 0.06	0.22 ± 0.07	0.34 ± 0.04
Schillingdata	0.29 ± 0.00	0.21 ± 0.01	0.34 ± 0.00	0.31 ± 0.01	0.36 ± 0.00	0.33 ± 0.02	0.29 ± 0.01	0.24 ± 0.03	0.29 ± 0.04	0.37 ± 0.01
Seeds	0.28 ± 0.03	0.21 ± 0.09	0.21 ± 0.10	0.19 ± 0.03	0.18 ± 0.10	0.18 ± 0.10	0.18 ± 0.10	0.14 ± 0.05	0.14 ± 0.05	0.33 ± 0.09
Segment	0.21 ± 0.00	0.23 ± 0.01	0.09 ± 0.02	0.09 ± 0.01	0.31 ± 0.01	0.31 ± 0.01	0.31 ± 0.01	0.10 ± 0.01	0.09 ± 0.01	0.32 ± 0.01
Seismic-bumps	0.25 ± 0.00	0.34 ± 0.02	0.25 ± 0.00	0.32 ± 0.02	0.26 ± 0.00	0.26 ± 0.00	0.26 ± 0.00	0.25 ± 0.01	0.26 ± 0.01	0.27 ± 0.01
Semeion	0.29 ± 0.00	0.16 ± 0.02	0.21 ± 0.01	0.13 ± 0.01	0.09 ± 0.01	0.09 ± 0.01	0.09 ± 0.01	0.11 ± 0.01	0.11 ± 0.01	0.40 ± 0.00
Setapprocesst1	0.44 ± 0.03	0.61 ± 0.19	0.56 ± 0.14	0.57 ± 0.09	0.60 ± 0.07	0.55 ± 0.10	0.57 ± 0.09	0.54 ± 0.21	0.54 ± 0.21	0.45 ± 0.28
Setapprocesst10	0.47 ± 0.03	0.73 ± 0.10	0.62 ± 0.11	0.60 ± 0.15	0.58 ± 0.05	0.58 ± 0.05	0.58 ± 0.05	0.63 ± 0.20	0.63 ± 0.17	0.61 ± 0.07
Setapprocesst11	0.47 ± 0.03	0.63 ± 0.19	0.55 ± 0.16	0.63 ± 0.12	0.58 ± 0.05	0.58 ± 0.05	0.58 ± 0.05	0.57 ± 0.12	0.54 ± 0.13	0.65 ± 0.08
Setapprocesst2	0.47 ± 0.03	0.54 ± 0.14	0.43 ± 0.24	0.56 ± 0.23	0.58 ± 0.05	0.58 ± 0.05	0.58 ± 0.05	0.56 ± 0.15	0.54 ± 0.16	0.37 ± 0.23
Setapprocesst3	0.47 ± 0.02	0.58 ± 0.11	0.60 ± 0.14	0.56 ± 0.12	0.58 ± 0.05	0.58 ± 0.05	0.58 ± 0.05	0.55 ± 0.11	0.56 ± 0.08	0.68 ± 0.12
Setapprocesst4	0.46 ± 0.03	0.66 ± 0.21	0.64 ± 0.11	0.60 ± 0.16	0.53 ± 0.07	0.53 ± 0.07	0.53 ± 0.07	0.61 ± 0.17	0.62 ± 0.13	0.51 ± 0.11
Setapprocesst5	0.46 ± 0.03	0.71 ± 0.12	0.64 ± 0.16	0.61 ± 0.14	0.59 ± 0.06	0.60 ± 0.07	0.60 ± 0.07	0.58 ± 0.11	0.57 ± 0.10	0.53 ± 0.25
Setapprocesst6	0.46 ± 0.01	0.54 ± 0.15	0.55 ± 0.11	0.58 ± 0.12	0.58 ± 0.05	0.58 ± 0.05	0.58 ± 0.05	0.55 ± 0.18	0.56 ± 0.16	0.61 ± 0.16
Setapprocesst7	0.47 ± 0.03	0.47 ± 0.17	0.56 ± 0.23	0.58 ± 0.13	0.58 ± 0.05	0.58 ± 0.05	0.58 ± 0.05	0.46 ± 0.20	0.47 ± 0.20	0.68 ± 0.11
Setapprocesst8	0.46 ± 0.03	0.54 ± 0.13	0.49 ± 0.15	0.57 ± 0.20	0.58 ± 0.05	0.58 ± 0.05	0.58 ± 0.05	0.52 ± 0.20	0.51 ± 0.21	0.71 ± 0.08
Setapprocesst9	0.47 ± 0.02	0.56 ± 0.14	0.52 ± 0.15	0.63 ± 0.07	0.58 ± 0.05	0.58 ± 0.05	0.58 ± 0.05	0.46 ± 0.21	0.52 ± 0.21	0.69 ± 0.12
Shillbiddingdataset	0.21 ± 0.00	0.17 ± 0.01	0.06 ± 0.01	0.09 ± 0.02	0.07 ± 0.01	0.06 ± 0.02	0.06 ± 0.02	0.05 ± 0.01	0.05 ± 0.02	0.16 ± 0.02
Shuttle-landing-control	0.19 ± 0.22	0.27 ± 0.32	0.14 ± 0.20	0.16 ± 0.23	0.07 ± 0.22	0.07 ± 0.22	0.07 ± 0.22	0.19 ± 0.31	0.19 ± 0.32	0.17 ± 0.37
Somervillehappinesssurvey2015	0.49 ± 0.03	0.52 ± 0.06	0.48 ± 0.10	0.56 ± 0.11	0.63 ± 0.08	0.64 ± 0.08	0.64 ± 0.09	0.59 ± 0.09	0.59 ± 0.10	0.57 ± 0.13
Sonar	0.46 ± 0.01	0.53 ± 0.07	0.52 ± 0.06	0.35 ± 0.10	0.58 ± 0.05	0.54 ± 0.08	0.51 ± 0.09	0.37 ± 0.15	0.37 ± 0.15	0.61 ± 0.09
Soybean	0.20 ± 0.00	0.08 ± 0.04	0.11 ± 0.02	0.11 ± 0.02	0.10 ± 0.03	0.09 ± 0.03	0.09 ± 0.03	0.09 ± 0.03	0.09 ± 0.02	0.28 ± 0.00
Spambase	0.42 ± 0.00	0.45 ± 0.02	0.26 ± 0.02	0.30 ± 0.02	0.26 ± 0.02	0.25 ± 0.02	0.26 ± 0.02	0.26 ± 0.02	0.29 ± 0.07	0.46 ± 0.02
Speaker-accent	0.31 ± 0.01	0.32 ± 0.04	0.30 ± 0.05	0.25 ± 0.06	0.37 ± 0.02	0.35 ± 0.02	0.35 ± 0.02	0.23 ± 0.03	0.25 ± 0.04	0.41 ± 0.02
Spect	0.45 ± 0.04	0.48 ± 0.18	0.48 ± 0.10	0.63 ± 0.11	0.59 ± 0.10	0.59 ± 0.11	0.60 ± 0.08	0.60 ± 0.14	0.60 ± 0.15	0.52 ± 0.10
Spectf	0.47 ± 0.02	0.46 ± 0.14	0.49 ± 0.12	0.54 ± 0.15	0.57 ± 0.06	0.57 ± 0.06	0.45 ± 0.19	0.48 ± 0.19	0.48 ± 0.14	0.53 ± 0.08
Statlog-australian-credit	0.46 ± 0.00	0.50 ± 0.03	0.51 ± 0.06	0.66 ± 0.02	0.57 ± 0.02	0.60 ± 0.05	0.60 ± 0.05	0.53 ± 0.03	0.54 ± 0.03	0.59 ± 0.05
Statlog-german-credit	0.45 ± 0.00	0.42 ± 0.04	0.47 ± 0.05	0.57 ± 0.03	0.49 ± 0.04	0.47 ± 0.04	0.47 ± 0.05	0.51 ± 0.03	0.50 ± 0.03	0.54 ± 0.02
Statlog-heart	0.41 ± 0.02	0.36 ± 0.07	0.45 ± 0.09	0.48 ± 0.11	0.41 ± 0.06	0.44 ± 0.10	0.44 ± 0.11	0.43 ± 0.06	0.41 ± 0.09	0.53 ± 0.06
Statlog-image	0.20 ± 0.00	0.23 ± 0.01	0.09 ± 0.02	0.09 ± 0.01	0.13 ± 0.01	0.12 ± 0.01	0.11 ± 0.01	0.09 ± 0.01	0.09 ± 0.02	0.32 ± 0.01
Statlog-landsat	0.22 ± 0.00	0.26 ± 0.01	0.21 ± 0.01	0.18 ± 0.02	0.19 ± 0.01	0.18 ± 0.01	0.18 ± 0.02	0.18 ± 0.01	0.18 ± 0.02	0.36 ± 0.01
Statlog-shuttle	0.17 ± 0.00	0.13 ± 0.01	0.01 ± 0.01	0.01 ± 0.01	0.03 ± 0.00	0.02 ± 0.00	0.02 ± 0.00	0.03 ± 0.00	0.03 ± 0.00	0.12 ± 0.00
Statlog-vehicle	0.36 ± 0.01	0.46 ± 0.01	0.33 ± 0.03	0.39 ± 0.03	0.34 ± 0.03	0.31 ± 0.02	0.31 ± 0.02	0.26 ± 0.02	0.27 ± 0.03	0.49 ± 0.03
Steel-plates	0.27 ± 0.00	0.32 ± 0.01	0.25 ± 0.02	0.28 ± 0.01	0.26 ± 0.02	0.26 ± 0.01	0.26 ± 0.02	0.25 ± 0.01	0.26 ± 0.02	0.38 ± 0.01
Synthetic-control	0.27 ± 0.01	0.13 ± 0.04	0.16 ± 0.03	0.10 ± 0.04	0.03 ± 0.04	0.02 ± 0.04	0.02 ± 0.04	0.04 ± 0.02	0.04 ± 0.02	0.38 ± 0.02
Teaching	0.45 ± 0.02	0.45 ± 0.03	0.46 ± 0.06	0.48 ± 0.08	0.53 ± 0.09	0.50 ± 0.08	0.50 ± 0.08	0.45 ± 0.04	0.46 ± 0.03	0.60 ± 0.06
Thoraricsurgery	0.35 ± 0.00	0.39 ± 0.07	0.37 ± 0.02	0.47 ± 0.06	0.39 ± 0.00	0.39 ± 0.01	0.39 ± 0.01	0.42 ± 0.05	0.41 ± 0.06	0.41 ± 0.02
Thyroid	0.27 ± 0.05	0.10 ± 0.09	0.19 ± 0.11	0.11 ± 0.09	0.40 ± 0.04	0.39 ± 0.04	0.39 ± 0.04	0.12 ± 0.08	0.12 ± 0.08	0.21 ± 0.14
Thyroid-train	0.20 ± 0.00	0.15 ± 0.02	0.03 ± 0.03	0.23 ± 0.02	0.18 ± 0.01	0.17 ± 0.01	0.16 ± 0.01	0.13 ± 0.03	0.15 ± 0.02	0.15 ± 0.02
Tic-tac-toe	0.46 ± 0.00	0.43 ± 0.01	0.23 ± 0.04	0.00 ± 0.00	0.09 ± 0.07	0.06 ± 0.07	0.02 ± 0.05	0.14 ± 0.03	0.13 ± 0.05	0.55 ± 0.02
Titanic	0.41 ± 0.01	0.42 ± 0.02	0.40 ± 0.02	0.39 ± 0.01	0.46 ± 0.02	0.46 ± 0.02	0.46 ± 0.02	0.40 ± 0.02	0.41 ± 0.02	0.47 ± 0.02
Trains	0.46 ± 0.08	0.40 ± 0.52	0.28 ± 0.46	0.42 ± 0.42	0.40 ± 0.52	0.30 ± 0.48	0.30 ± 0.48	0.28 ± 0.37	0.27 ± 0.37	1.00 ± 0.00
Transfusion	0.42 ± 0.01	0.43 ± 0.02	0.40 ± 0.02	0.53 ± 0.04	0.50 ± 0.03	0.52 ± 0.03	0.53 ± 0.03	0.39 ± 0.01	0.39 ± 0.01	0.49 ± 0.02
Trial	0.34 ± 0.01	0.30 ± 0.06	0.00 ± 0.00	0.02 ± 0.05	0.07 ± 0.06	0.06 ± 0.06	0.06 ± 0.06	0.06 ± 0.06	0.05 ± 0.06	0.00 ± 0.00
Turkiye-student-evaluation	0.40 ± 0.00	0.28 ± 0.02	0.01 ± 0.01	0.25 ± 0.02	0.17 ± 0.01	0.16 ± 0.01	0.16 ± 0.01	0.00 ± 0.00	0.00 ± 0.00	0.01 ± 0.01
Unbalanced	0.12 ± 0.02	0.27 ± 0.06	0.12 ± 0.02	0.14 ± 0.06	0.12 ± 0.02	0.13 ± 0.02	0.13 ± 0.03	0.13 ± 0.02	0.13 ± 0.02	0.12 ± 0.02
Urbanlandcover	0.26 ± 0.01	0.21 ± 0.05	0.21 ± 0.04	0.22 ± 0.06	0.43 ± 0.01	0.43 ± 0.01	0.43 ± 0.01	0.19 ± 0.06	0.19 ± 0.06	0.33 ± 0.05
Userknowledgemodeling	0.36 ± 0.02	0.23 ± 0.05	0.15 ± 0.06	0.30 ± 0.09	0.33 ± 0.05	0.27 ± 0.04	0.27 ± 0.04	0.17 ± 0.05	0.15 ± 0.07	0.26 ± 0.08
Vehicle	0.35 ± 0.01	0.46 ± 0.02	0.33 ± 0.03	0.39 ± 0.03	0.59 ± 0.01	0.59 ± 0.01	0.59 ± 0.01	*0.26 ± 0.02*	0.27 ± 0.03	0.49 ± 0.03
Vertebral-column-2classes	0.40 ± 0.04	0.42 ± 0.09	0.37 ± 0.07	0.42 ± 0.06	0.37 ± 0.08	0.38 ± 0.09	0.38 ± 0.08	*0.32 ± 0.06*	*0.32 ± 0.06*	0.51 ± 0.08
Vertebral-column-3classes	0.36 ± 0.02	0.28 ± 0.04	0.31 ± 0.04	0.37 ± 0.04	0.32 ± 0.06	0.31 ± 0.07	0.32 ± 0.06	*0.26 ± 0.04*	*0.26 ± 0.04*	0.40 ± 0.06
Veteran	*0.43 ± 0.01*	0.46 ± 0.07	0.45 ± 0.06	0.62 ± 0.07	0.54 ± 0.01	0.54 ± 0.01	0.54 ± 0.01	0.54 ± 0.12	0.51 ± 0.12	0.51 ± 0.08
Vowel	0.24 ± 0.00	0.21 ± 0.01	0.17 ± 0.02	*0.03 ± 0.03*	0.16 ± 0.01	0.13 ± 0.01	0.11 ± 0.02	0.11 ± 0.02	0.10 ± 0.02	0.35 ± 0.01
Wall-following	0.23 ± 0.01	0.44 ± 0.01	*0.04 ± 0.02*	0.24 ± 0.01	0.23 ± 0.02	0.22 ± 0.02	0.21 ± 0.02	0.23 ± 0.01	0.23 ± 0.02	0.35 ± 0.01
Waveform-noise	0.41 ± 0.00	0.34 ± 0.01	0.39 ± 0.01	0.42 ± 0.01	*0.30 ± 0.02*	0.31 ± 0.01	0.32 ± 0.02	0.31 ± 0.02	0.32 ± 0.02	0.56 ± 0.02
Waveform	0.39 ± 0.00	0.33 ± 0.01	0.38 ± 0.01	0.39 ± 0.02	*0.30 ± 0.02*	0.31 ± 0.01	0.31 ± 0.02	*0.30 ± 0.01*	0.32 ± 0.01	0.56 ± 0.01
Wbc	0.18 ± 0.03	0.14 ± 0.06	0.23 ± 0.02	0.18 ± 0.06	0.13 ± 0.08	0.13 ± 0.08	*0.12 ± 0.09*	0.19 ± 0.04	0.18 ± 0.05	0.30 ± 0.05
Wdbc	0.28 ± 0.02	0.24 ± 0.11	0.24 ± 0.10	0.20 ± 0.05	0.61 ± 0.01	0.61 ± 0.01	0.61 ± 0.01	*0.16 ± 0.05*	0.17 ± 0.07	0.32 ± 0.05
Weathernominal	0.49 ± 0.16	0.44 ± 0.17	0.42 ± 0.37	0.52 ± 0.25	0.38 ± 0.41	0.48 ± 0.43	*0.31 ± 0.41*	*0.31 ± 0.40*	*0.31 ± 0.41*	0.61 ± 0.44
Weathernumeric	0.57 ± 0.15	0.49 ± 0.20	*0.27 ± 0.36*	*0.27 ± 0.34*	0.38 ± 0.41	0.54 ± 0.48	0.54 ± 0.48	0.31 ± 0.41	0.31 ± 0.41	0.61 ± 0.44
Website-phishingdata	0.35 ± 0.00	0.28 ± 0.02	*0.22 ± 0.02*	0.25 ± 0.02	0.31 ± 0.01	0.30 ± 0.01	0.29 ± 0.02	0.25 ± 0.02	0.25 ± 0.03	0.35 ± 0.02
Wholesalecustomersdata	0.29 ± 0.02	0.30 ± 0.05	0.29 ± 0.04	0.34 ± 0.07	0.57 ± 0.01	0.57 ± 0.01	0.57 ± 0.01	*0.25 ± 0.05*	0.26 ± 0.05	0.30 ± 0.06
Wifi-localization	0.26 ± 0.01	*0.09 ± 0.02*	0.11 ± 0.01	*0.09 ± 0.02*	0.32 ± 0.02	0.31 ± 0.02	0.31 ± 0.02	*0.09 ± 0.01*	*0.09 ± 0.01*	0.32 ± 0.03
Wilt	0.22 ± 0.00	0.27 ± 0.01	0.13 ± 0.02	0.23 ± 0.01	0.23 ± 0.00	0.23 ± 0.00	0.23 ± 0.00	*0.12 ± 0.03*	*0.12 ± 0.02*	0.24 ± 0.00
Wine-quality-red	*0.29 ± 0.01*	0.32 ± 0.01	0.33 ± 0.01	0.34 ± 0.01	0.35 ± 0.01	0.35 ± 0.01	0.35 ± 0.01	0.30 ± 0.01	0.30 ± 0.01	0.39 ± 0.01
Wine-quality-white	*0.29 ± 0.00*	0.32 ± 0.00	0.32 ± 0.01	0.31 ± 0.01	0.35 ± 0.01	0.35 ± 0.01	0.35 ± 0.01	*0.29 ± 0.00*	*0.29 ± 0.00*	0.39 ± 0.01
Wine	0.27 ± 0.02	0.09 ± 0.10	0.17 ± 0.11	0.15 ± 0.10	*0.04 ± 0.08*	0.06 ± 0.09	0.06 ± 0.09	0.09 ± 0.08	0.09 ± 0.08	0.38 ± 0.08
Yamilnaduelectricty	*0.21 ± 0.00*	0.22 ± 0.00	0.25 ± 0.00	0.28 ± 0.00	0.31 ± 0.00	0.31 ± 0.00	0.31 ± 0.00	0.22 ± 0.00	0.22 ± 0.00	0.30 ± 0.00
Yeast	0.26 ± 0.00	*0.24 ± 0.01*	0.27 ± 0.01	0.31 ± 0.01	0.28 ± 0.01	0.28 ± 0.01	0.28 ± 0.02	*0.24 ± 0.01*	*0.24 ± 0.01*	0.35 ± 0.01
Youtobe-kabita-preprocessing	*0.33 ± 0.00*	0.37 ± 0.00	0.35 ± 0.01	0.40 ± 0.00	0.42 ± 0.01	0.43 ± 0.01	0.43 ± 0.01	*0.33 ± 0.00*	*0.33 ± 0.00*	0.44 ± 0.01
Youtobe-nisha-preprocessing	*0.33 ± 0.00*	0.36 ± 0.00	0.35 ± 0.01	0.39 ± 0.01	0.41 ± 0.01	0.42 ± 0.01	0.42 ± 0.01	*0.33 ± 0.00*	*0.33 ± 0.00*	0.44 ± 0.00
Z-alizadehsani	0.43 ± 0.01	0.39 ± 0.07	0.43 ± 0.09	0.46 ± 0.07	0.54 ± 0.01	0.54 ± 0.01	0.54 ± 0.01	*0.37 ± 0.09*	0.39 ± 0.07	0.54 ± 0.05
Zoo	0.23 ± 0.02	*0.06 ± 0.09*	0.10 ± 0.10	*0.06 ± 0.07*	0.08 ± 0.11	0.08 ± 0.11	0.08 ± 0.11	0.07 ± 0.07	0.07 ± 0.07	0.27 ± 0.06
Average rmse (rank)	0.3321 (5)	0.3377 (6)	0.2832 (2)	0.3247 (4)	0.3630 (9)	0.3562 (8)	0.3518 (7)	0.2817 (1)	0.2841 (3)	0.3928 (10)
Average rmse std (rank)	0.0152 (1)	0.0496 (4)	0.0531 (7)	0.0570 (9)	0.0442 (2)	0.0478 (3)	0.0497 (5)	0.0554 (8)	0.0573 (10)	0.0513 (6)

**Table 9:** The detailed experimental results on mean absolute error and standard deviation.

Dataset	RN	NB	J48	KNN	SVM1	SVM2	SVM3	ANN1	ANN2	OneR
Abalone	0.34 ± 0.01	0.29 ± 0.01	0.29 ± 0.01	0.28 ± 0.02	0.22 ± 0.02	0.22 ± 0.02	0.22 ± 0.01	0.29 ± 0.01	0.29 ± 0.01	0.26 ± 0.01
Absenteeism-at-work	0.31 ± 0.01	0.16 ± 0.03	0.00 ± 0.01	0.16 ± 0.03	0.15 ± 0.03	0.15 ± 0.03	0.15 ± 0.03	0.04 ± 0.01	0.04 ± 0.02	0.05 ± 0.02
Acute-inflammation	0.23 ± 0.02	0.10 ± 0.03	0.00 ± 0.00	0.01 ± 0.00	0.00 ± 0.00	0.00 ± 0.00	0.00 ± 0.00	0.01 ± 0.00	0.00 ± 0.00	0.21 ± 0.08
Acute-nephritis	0.21 ± 0.01	0.05 ± 0.05	0.00 ± 0.00	0.01 ± 0.00	0.00 ± 0.00	0.00 ± 0.00	0.00 ± 0.00	0.01 ± 0.00	0.00 ± 0.00	0.08 ± 0.08
Adult	0.34 ± 0.00	0.20 ± 0.02	0.22 ± 0.03	0.22 ± 0.04	0.17 ± 0.04	0.17 ± 0.04	0.17 ± 0.04	0.21 ± 0.03	0.21 ± 0.03	0.20 ± 0.01
Aggregation	0.04 ± 0.01	0.00 ± 0.00	0.00 ± 0.00	0.00 ± 0.00	0.00 ± 0.00	0.00 ± 0.00	0.00 ± 0.00	0.02 ± 0.00	0.01 ± 0.01	0.12 ± 0.01
Algerianforest	0.30 ± 0.02	0.06 ± 0.05	0.05 ± 0.04	0.12 ± 0.07	0.41 ± 0.02	0.38 ± 0.04	0.38 ± 0.04	0.04 ± 0.03	0.04 ± 0.04	0.03 ± 0.05
Annealing	0.15 ± 0.00	0.12 ± 0.01	0.02 ± 0.01	0.04 ± 0.01	0.04 ± 0.01	0.03 ± 0.01	0.03 ± 0.01	0.04 ± 0.01	0.04 ± 0.01	0.07 ± 0.00
Arrhythmia	0.10 ± 0.00	0.06 ± 0.01	0.06 ± 0.01	0.07 ± 0.00	0.06 ± 0.00	0.05 ± 0.01	0.05 ± 0.01	0.05 ± 0.01	0.07 ± 0.02	0.06 ± 0.00
Au1-1000	0.38 ± 0.00	0.36 ± 0.01	0.30 ± 0.03	0.32 ± 0.04	0.26 ± 0.00	0.26 ± 0.00	0.26 ± 0.01	0.31 ± 0.04	0.29 ± 0.04	0.26 ± 0.00
Au4-2500	0.36 ± 0.00	0.32 ± 0.01	0.20 ± 0.03	0.24 ± 0.03	0.26 ± 0.02	0.26 ± 0.02	0.26 ± 0.02	0.24 ± 0.03	0.24 ± 0.03	0.31 ± 0.03
Au6-1000	0.21 ± 0.00	0.21 ± 0.00	0.20 ± 0.01	0.22 ± 0.01	0.19 ± 0.00	0.19 ± 0.00	0.19 ± 0.00	0.21 ± 0.01	0.22 ± 0.01	0.19 ± 0.01
Au6-250-drift-au6-cd1-500	0.21 ± 0.00	0.21 ± 0.00	0.20 ± 0.01	0.21 ± 0.01	0.19 ± 0.00	0.19 ± 0.00	0.19 ± 0.00	0.21 ± 0.00	0.21 ± 0.01	0.19 ± 0.01
Au6-cd1-400	0.21 ± 0.00	0.20 ± 0.01	0.16 ± 0.02	0.21 ± 0.02	0.18 ± 0.00	0.18 ± 0.00	0.18 ± 0.00	0.20 ± 0.01	0.20 ± 0.01	0.17 ± 0.02
Au7-300-drift-au7-cpd1-800	0.31 ± 0.00	0.29 ± 0.00	0.26 ± 0.01	0.27 ± 0.02	0.29 ± 0.01	0.29 ± 0.01	0.29 ± 0.01	0.28 ± 0.01	0.28 ± 0.01	0.30 ± 0.01
Au7-700	0.26 ± 0.00	0.24 ± 0.01	0.20 ± 0.01	0.26 ± 0.02	0.25 ± 0.01	0.25 ± 0.01	0.25 ± 0.01	0.24 ± 0.01	0.24 ± 0.01	0.22 ± 0.02
Au7-cpd1-500	0.30 ± 0.00	0.28 ± 0.01	0.21 ± 0.02	0.25 ± 0.02	0.25 ± 0.00	0.25 ± 0.01	0.25 ± 0.01	0.25 ± 0.02	0.25 ± 0.02	0.24 ± 0.02
Audiology-std	0.09 ± 0.00	0.03 ± 0.01	0.03 ± 0.01	0.04 ± 0.01	0.04 ± 0.01	0.04 ± 0.01	0.03 ± 0.01	0.02 ± 0.01	0.03 ± 0.01	0.06 ± 0.00
Audit-risk	0.28 ± 0.01	0.05 ± 0.02	0.00 ± 0.00	0.02 ± 0.01	0.02 ± 0.01	0.01 ± 0.01	0.01 ± 0.01	0.04 ± 0.02	0.04 ± 0.02	0.00 ± 0.00
Autism-adolescent-data	0.37 ± 0.03	0.04 ± 0.03	0.00 ± 0.00	0.10 ± 0.06	0.04 ± 0.05	0.05 ± 0.05	0.04 ± 0.05	0.10 ± 0.09	0.10 ± 0.09	0.00 ± 0.00
Autism-adult-data	0.29 ± 0.01	0.03 ± 0.02	0.00 ± 0.00	0.05 ± 0.04	0.01 ± 0.01	0.00 ± 0.01	0.01 ± 0.01	0.00 ± 0.00	0.00 ± 0.00	0.00 ± 0.00
Autism-child-data	0.40 ± 0.01	0.04 ± 0.02	0.00 ± 0.00	0.12 ± 0.04	0.00 ± 0.01	0.00 ± 0.00	0.00 ± 0.00	0.01 ± 0.01	0.01 ± 0.01	0.00 ± 0.00
Autos	0.17 ± 0.01	0.13 ± 0.03	0.06 ± 0.02	0.07 ± 0.03	0.19 ± 0.01	0.18 ± 0.01	0.18 ± 0.01	0.07 ± 0.02	0.07 ± 0.01	0.11 ± 0.03
Avila	0.07 ± 0.00	0.13 ± 0.00	0.01 ± 0.00	0.04 ± 0.00	0.05 ± 0.00	0.05 ± 0.00	0.05 ± 0.00	0.08 ± 0.00	0.08 ± 0.00	0.05 ± 0.00
Balance-scale	0.28 ± 0.01	0.21 ± 0.01	0.19 ± 0.02	0.13 ± 0.03	0.06 ± 0.01	0.05 ± 0.01	0.04 ± 0.01	0.08 ± 0.01	0.08 ± 0.01	0.29 ± 0.03
Balloons	0.41 ± 0.12	0.50 ± 0.03	0.46 ± 0.19	0.34 ± 0.12	0.30 ± 0.35	0.20 ± 0.35	0.20 ± 0.35	0.28 ± 0.23	0.27 ± 0.24	0.55 ± 0.37
Bank	0.20 ± 0.00	0.18 ± 0.01	0.14 ± 0.01	0.14 ± 0.01	0.10 ± 0.01	0.10 ± 0.01	0.10 ± 0.01	0.13 ± 0.01	0.13 ± 0.02	0.11 ± 0.01
Blood	0.35 ± 0.01	0.29 ± 0.02	0.30 ± 0.01	0.32 ± 0.04	0.22 ± 0.02	0.21 ± 0.02	0.22 ± 0.01	0.30 ± 0.02	0.30 ± 0.02	0.24 ± 0.02
Breast-cancer-wisc-diag	0.25 ± 0.02	0.07 ± 0.04	0.07 ± 0.04	0.04 ± 0.02	0.02 ± 0.02	0.02 ± 0.02	0.02 ± 0.01	0.04 ± 0.02	0.04 ± 0.02	0.11 ± 0.04
Breast-cancer-wisc-prog	0.36 ± 0.01	0.34 ± 0.09	0.29 ± 0.06	0.27 ± 0.09	0.21 ± 0.06	0.22 ± 0.09	0.22 ± 0.10	0.27 ± 0.09	0.27 ± 0.11	0.30 ± 0.08
Breast-cancer-wisc	0.14 ± 0.02	0.04 ± 0.02	0.07 ± 0.03	0.05 ± 0.03	0.03 ± 0.02	0.03 ± 0.02	0.03 ± 0.02	0.06 ± 0.03	0.05 ± 0.02	0.07 ± 0.05
Breast-cancer	0.39 ± 0.01	0.32 ± 0.04	0.37 ± 0.03	0.30 ± 0.08	0.26 ± 0.05	0.27 ± 0.05	0.28 ± 0.05	0.32 ± 0.06	0.33 ± 0.04	0.30 ± 0.05
Breast-tissue	0.19 ± 0.02	0.11 ± 0.04	0.11 ± 0.05	0.11 ± 0.04	0.14 ± 0.05	0.11 ± 0.06	0.10 ± 0.06	0.14 ± 0.02	0.14 ± 0.03	0.15 ± 0.03
Bupa	0.45 ± 0.01	0.46 ± 0.03	0.40 ± 0.08	0.38 ± 0.05	0.41 ± 0.02	0.41 ± 0.02	0.41 ± 0.02	0.37 ± 0.04	0.36 ± 0.04	0.46 ± 0.08
Caesarian	0.43 ± 0.05	0.41 ± 0.10	0.46 ± 0.06	0.47 ± 0.14	0.45 ± 0.06	0.38 ± 0.12	0.41 ± 0.17	0.44 ± 0.18	0.44 ± 0.17	0.51 ± 0.18
Car	0.22 ± 0.00	0.12 ± 0.01	0.01 ± 0.00	0.02 ± 0.01	0.01 ± 0.01	0.01 ± 0.01	0.01 ± 0.00	0.04 ± 0.01	0.04 ± 0.01	0.15 ± 0.00
Cardiotocography-10classes	0.14 ± 0.00	0.06 ± 0.00	0.04 ± 0.00	0.04 ± 0.01	0.04 ± 0.00	0.04 ± 0.01	0.03 ± 0.00	0.04 ± 0.01	0.04 ± 0.00	0.10 ± 0.00
Cardiotocography-3classes	0.22 ± 0.00	0.12 ± 0.02	0.06 ± 0.02	0.05 ± 0.01	0.06 ± 0.01	0.05 ± 0.01	0.05 ± 0.01	0.06 ± 0.01	0.06 ± 0.01	0.12 ± 0.02
Cervical-cancer	0.34 ± 0.03	0.10 ± 0.07	0.16 ± 0.12	0.12 ± 0.11	0.29 ± 0.03	0.26 ± 0.07	0.26 ± 0.07	0.08 ± 0.08	0.07 ± 0.08	0.22 ± 0.09
Chemicalcomposionofceramic	0.24 ± 0.02	0.00 ± 0.00	0.01 ± 0.04	0.01 ± 0.00	0.54 ± 0.02	0.54 ± 0.02	0.54 ± 0.02	0.01 ± 0.00	0.00 ± 0.00	0.00 ± 0.00
Chess-krvk	0.10 ± 0.00	0.09 ± 0.00	0.06 ± 0.00	0.06 ± 0.00	0.06 ± 0.00	0.06 ± 0.00	0.06 ± 0.00	0.07 ± 0.00	0.07 ± 0.00	0.08 ± 0.00
Chess-krvkp	0.47 ± 0.00	0.21 ± 0.02	0.01 ± 0.00	0.10 ± 0.01	0.01 ± 0.01	0.01 ± 0.01	0.01 ± 0.01	0.01 ± 0.01	0.01 ± 0.01	0.34 ± 0.01
Congressional-voting	0.47 ± 0.00	0.45 ± 0.04	0.46 ± 0.03	0.45 ± 0.05	0.38 ± 0.04	0.39 ± 0.05	0.39 ± 0.06	0.43 ± 0.03	0.43 ± 0.03	0.37 ± 0.03
Conn-bench-sonar-mines-rocks	0.45 ± 0.01	0.31 ± 0.09	0.29 ± 0.07	0.14 ± 0.07	0.16 ± 0.07	0.13 ± 0.08	0.12 ± 0.08	0.20 ± 0.09	0.18 ± 0.09	0.37 ± 0.10
Conn-bench-vowel-deterding	0.11 ± 0.00	0.08 ± 0.01	0.04 ± 0.01	0.01 ± 0.00	0.01 ± 0.00	0.00 ± 0.00	0.00 ± 0.00	0.04 ± 0.01	0.04 ± 0.01	0.12 ± 0.01
Connect-4	0.38 ± 0.00	0.38 ± 0.01	0.24 ± 0.02	0.29 ± 0.03	0.26 ± 0.00	0.24 ± 0.01	0.23 ± 0.02	0.27 ± 0.02	0.26 ± 0.02	0.26 ± 0.00
Connectionist	0.45 ± 0.01	0.32 ± 0.11	0.27 ± 0.07	0.13 ± 0.06	0.39 ± 0.09	0.30 ± 0.12	0.27 ± 0.12	0.17 ± 0.08	0.17 ± 0.08	0.37 ± 0.07
Contrac	0.42 ± 0.00	0.37 ± 0.02	0.34 ± 0.02	0.38 ± 0.02	0.30 ± 0.01	0.30 ± 0.02	0.31 ± 0.03	0.35 ± 0.01	0.34 ± 0.01	0.35 ± 0.01
Covid-19	0.32 ± 0.08	0.29 ± 0.17	0.24 ± 0.24	0.33 ± 0.23	0.27 ± 0.26	0.30 ± 0.29	0.23 ± 0.27	0.23 ± 0.23	0.22 ± 0.23	0.13 ± 0.23
Credit-approval	0.41 ± 0.01	0.23 ± 0.03	0.19 ± 0.03	0.18 ± 0.06	0.15 ± 0.05	0.15 ± 0.05	0.15 ± 0.05	0.16 ± 0.03	0.17 ± 0.04	0.14 ± 0.04
Crowdsource	0.12 ± 0.00	0.06 ± 0.01	0.04 ± 0.00	0.02 ± 0.00	0.08 ± 0.00	0.08 ± 0.00	0.08 ± 0.00	0.03 ± 0.01	0.03 ± 0.01	0.07 ± 0.01
Crx	0.41 ± 0.01	0.22 ± 0.03	0.19 ± 0.03	0.19 ± 0.05	0.44 ± 0.04	0.43 ± 0.04	0.42 ± 0.05	0.18 ± 0.04	0.17 ± 0.02	0.14 ± 0.04
Cryother	0.32 ± 0.05	0.18 ± 0.11	0.11 ± 0.07	0.11 ± 0.08	0.18 ± 0.13	0.17 ± 0.13	0.17 ± 0.13	0.14 ± 0.08	0.17 ± 0.09	0.19 ± 0.11
Cylinder-bands	0.45 ± 0.01	0.33 ± 0.06	0.29 ± 0.06	0.31 ± 0.07	0.24 ± 0.04	0.21 ± 0.04	0.19 ± 0.03	0.27 ± 0.05	0.26 ± 0.05	0.33 ± 0.04
Dbworld-bodies	0.49 ± 0.01	0.24 ± 0.15	0.21 ± 0.16	0.41 ± 0.10	0.45 ± 0.06	0.45 ± 0.06	0.36 ± 0.13	/	/	0.14 ± 0.13
Dbworld-bodies-stemmed	0.48 ± 0.01	0.23 ± 0.13	0.15 ± 0.13	0.36 ± 0.14	0.45 ± 0.06	0.41 ± 0.09	0.26 ± 0.13	/	/	0.26 ± 0.20
Dbworld-subjects	0.49 ± 0.01	0.17 ± 0.10	0.30 ± 0.10	0.25 ± 0.12	0.45 ± 0.06	0.45 ± 0.06	0.45 ± 0.06	0.13 ± 0.12	0.13 ± 0.14	0.37 ± 0.12
Dbworld-subjects-stemmed	0.49 ± 0.01	0.17 ± 0.11	0.27 ± 0.14	0.19 ± 0.09	0.45 ± 0.06	0.45 ± 0.06	0.45 ± 0.06	0.14 ± 0.12	0.14 ± 0.11	0.39 ± 0.10
Dermatology	0.19 ± 0.00	0.01 ± 0.01	0.02 ± 0.01	0.02 ± 0.01	0.01 ± 0.01	0.01 ± 0.01	0.01 ± 0.01	0.01 ± 0.00	0.01 ± 0.00	0.17 ± 0.01
Diabetes	0.39 ± 0.02	0.28 ± 0.03	0.32 ± 0.05	0.30 ± 0.05	0.35 ± 0.00	0.35 ± 0.00	0.35 ± 0.00	0.30 ± 0.02	0.29 ± 0.02	0.29 ± 0.05
Diabetic	0.46 ± 0.01	0.43 ± 0.02	0.38 ± 0.03	0.39 ± 0.05	0.42 ± 0.05	0.41 ± 0.06	0.40 ± 0.06	0.33 ± 0.03	0.33 ± 0.03	0.47 ± 0.06
Divorce	0.10 ± 0.03	0.02 ± 0.03	0.06 ± 0.06	0.03 ± 0.03	0.02 ± 0.03	0.02 ± 0.03	0.02 ± 0.03	0.02 ± 0.03	0.02 ± 0.03	0.05 ± 0.05
Dota2train	0.50 ± 0.00	0.47 ± 0.02	0.47 ± 0.04	0.45 ± 0.03	0.47 ± 0.00	0.46 ± 0.04	0.47 ± 0.03	0.50 ± 0.03	0.48 ± 0.02	0.45 ± 0.04
Dow-jones-index	0.45 ± 0.02	0.50 ± 0.03	0.29 ± 0.04	0.45 ± 0.06	0.48 ± 0.00	0.48 ± 0.00	0.48 ± 0.00	0.46 ± 0.03	0.45 ± 0.03	0.44 ± 0.03
Dry-bean-dataset	0.06 ± 0.00	0.03 ± 0.00	0.02 ± 0.00	0.02 ± 0.00	0.11 ± 0.01	0.11 ± 0.01	0.11 ± 0.01	0.03 ± 0.00	0.03 ± 0.00	0.09 ± 0.01
Early-stage-diabetes-data-upload	0.37 ± 0.01	0.15 ± 0.03	0.05 ± 0.03	0.02 ± 0.02	0.06 ± 0.04	0.05 ± 0.04	0.05 ± 0.04	0.04 ± 0.02	0.04 ± 0.03	0.18 ± 0.04
Echocardiogram	0.40 ± 0.03	0.22 ± 0.11	0.24 ± 0.10	0.26 ± 0.13	0.18 ± 0.08	0.19 ± 0.11	0.19 ± 0.11	0.24 ± 0.12	0.23 ± 0.12	0.15 ± 0.07
Ecoli	0.14 ± 0.00	0.04 ± 0.01	0.05 ± 0.02	0.05 ± 0.02	0.03 ± 0.01	0.03 ± 0.01	0.03 ± 0.01	0.05 ± 0.01	0.05 ± 0.01	0.08 ± 0.02
Eegeyesate	0.45 ± 0.00	0.53 ± 0.02	0.17 ± 0.01	0.16 ± 0.02	0.45 ± 0.00	0.45 ± 0.00	0.45 ± 0.00	0.49 ± 0.01	0.48 ± 0.01	0.37 ± 0.01
Electrical	0.30 ± 0.03	0.06 ± 0.03	0.00 ± 0.00	0.09 ± 0.07	0.17 ± 0.08	0.17 ± 0.07	0.16 ± 0.06	0.02 ± 0.02	0.02 ± 0.01	0.00 ± 0.01
Energy-y1	0.25 ± 0.00	0.13 ± 0.01	0.02 ± 0.01	0.16 ± 0.03	0.08 ± 0.01	0.07 ± 0.01	0.07 ± 0.02	0.09 ± 0.01	0.09 ± 0.01	0.10 ± 0.01
Energy-y2	0.23 ± 0.00	0.11 ± 0.01	0.09 ± 0.01	0.16 ± 0.02	0.07 ± 0.01	0.06 ± 0.01	0.06 ± 0.01	0.08 ± 0.01	0.08 ± 0.02	0.08 ± 0.02
Extentionofz-alizadehsani	0.38 ± 0.01	0.08 ± 0.04	0.01 ± 0.01	0.10 ± 0.05	0.29 ± 0.01	0.29 ± 0.01	0.29 ± 0.01	0.02 ± 0.02	0.02 ± 0.02	0.14 ± 0.05
Fertility	0.21 ± 0.03	0.20 ± 0.03	0.22 ± 0.04	0.18 ± 0.11	0.12 ± 0.04	0.11 ± 0.06	0.11 ± 0.07	0.13 ± 0.08	0.13 ± 0.10	0.12 ± 0.04
First-order	0.17 ± 0.00	0.68 ± 0.04	0.00 ± 0.00	0.01 ± 0.00	0.10 ± 0.00	0.05 ± 0.01	0.02 ± 0.01	0.00 ± 0.00	0.00 ± 0.00	0.09 ± 0.02
Flags	0.18 ± 0.00	0.13 ± 0.01	0.11 ± 0.02	0.14 ± 0.02	0.12 ± 0.02	0.11 ± 0.03	0.12 ± 0.03	0.14 ± 0.03	0.13 ± 0.03	0.11 ± 0.02
Foresttypes	0.23 ± 0.01	0.03 ± 0.02	0.03 ± 0.03	0.03 ± 0.02	0.35 ± 0.01	0.34 ± 0.02	0.34 ± 0.02	0.03 ± 0.02	0.02 ± 0.02	0.09 ± 0.04
Garments-worker-productivity	0.29 ± 0.00	0.29 ± 0.01	0.22 ± 0.02	0.30 ± 0.01	0.26 ± 0.01	0.26 ± 0.01	0.25 ± 0.01	0.26 ± 0.01	0.26 ± 0.01	0.27 ± 0.01
Gender-name-dataset	0.47 ± 0.00	0.42 ± 0.04	0.48 ± 0.00	0.41 ± 0.01	0.36 ± 0.01	0.36 ± 0.02	0.36 ± 0.02	0.48 ± 0.01	0.48 ± 0.01	0.39 ± 0.01
Gesture-a1-raw	0.12 ± 0.00	0.11 ± 0.01	0.04 ± 0.01	0.02 ± 0.00	0.24 ± 0.00	0.24 ± 0.00	0.24 ± 0.00	0.04 ± 0.01	0.04 ± 0.00	0.01 ± 0.01
Gesture-a1-va3	0.21 ± 0.01	0.16 ± 0.01	0.14 ± 0.02	0.10 ± 0.01	0.24 ± 0.00	0.24 ± 0.00	0.24 ± 0.00	0.14 ± 0.01	0.14 ± 0.01	0.15 ± 0.01
Gesture-a2-raw	0.13 ± 0.00	0.13 ± 0.01	0.05 ± 0.01	0.02 ± 0.01	0.24 ± 0.00	0.24 ± 0.00	0.24 ± 0.00	0.05 ± 0.01	0.05 ± 0.01	0.02 ± 0.01
Gesture-a2-va3	0.23 ± 0.01	0.23 ± 0.03	0.18 ± 0.01	0.13 ± 0.02	0.24 ± 0.00	0.24 ± 0.00	0.24 ± 0.00	0.18 ± 0.01	0.18 ± 0.02	0.18 ± 0.02
Gesture-a3-raw	0.10 ± 0.00	0.16 ± 0.01	0.03 ± 0.00	0.02 ± 0.00	0.26 ± 0.00	0.26 ± 0.00	0.26 ± 0.00	0.05 ± 0.01	0.05 ± 0.01	0.02 ± 0.00
Gesture-a3-va3	0.24 ± 0.00	0.20 ± 0.01	0.10 ± 0.01	0.04 ± 0.01	0.26 ± 0.00	0.26 ± 0.00	0.26 ± 0.00	0.17 ± 0.01	0.16 ± 0.01	0.20 ± 0.01
Gesture-b1-raw	0.10 ± 0.01	0.18 ± 0.01	0.03 ± 0.01	0.02 ± 0.01	0.20 ± 0.00	0.20 ± 0.00	0.20 ± 0.00	0.05 ± 0.01	0.04 ± 0.01	0.03 ± 0.01
Gesture-b1-va3	0.24 ± 0.01	0.26 ± 0.03	0.13 ± 0.02	0.04 ± 0.01	0.25 ± 0.00	0.25 ± 0.00	0.25 ± 0.00	0.18 ± 0.01	0.17 ± 0.02	0.23 ± 0.02
Gesture-b3-raw	0.15 ± 0.01	0.18 ± 0.01	0.04 ± 0.01	0.02 ± 0.00	0.27 ± 0.00	0.27 ± 0.00	0.27 ± 0.00	0.06 ± 0.01	0.06 ± 0.01	0.02 ± 0.01
Gesture-b3-va3	0.28 ± 0.00	0.23 ± 0.01	0.18 ± 0.01	0.09 ± 0.01	0.27 ± 0.00	0.27 ± 0.00	0.27 ± 0.00	0.19 ± 0.01	0.19 ± 0.01	0.24 ± 0.02
Gesture-c1-raw	0.15 ± 0.01	0.15 ± 0.01	0.05 ± 0.01	0.02 ± 0.01	0.30 ± 0.00	0.30 ± 0.00	0.30 ± 0.00	0.06 ± 0.01	0.06 ± 0.01	0.02 ± 0.01
Gesture-c1-va3	0.26 ± 0.01	0.20 ± 0.01	0.17 ± 0.01	0.12 ± 0.01	0.30 ± 0.00	0.30 ± 0.00	0.30 ± 0.00	0.16 ± 0.01	0.16 ± 0.01	0.23 ± 0.02
Gesture-c3-raw	0.14 ± 0.01	0.16 ± 0.02	0.05 ± 0.01	0.03 ± 0.01	0.29 ± 0.00	0.29 ± 0.00	0.29 ± 0.00	0.07 ± 0.01	0.07 ± 0.01	0.02 ± 0.01
Gesture-c3-va3	0.27 ± 0.01	0.22 ± 0.01	0.19 ± 0.02	0.15 ± 0.02	0.29 ± 0.00	0.29 ± 0.00	0.29 ± 0.00	0.19 ± 0.01	0.20 ± 0.02	0.23 ± 0.01
Glass	0.18 ± 0.01	0.18 ± 0.02	0.12 ± 0.02	0.10 ± 0.03	0.09 ± 0.02	0.09 ± 0.03	0.10 ± 0.03	0.13 ± 0.02	0.12 ± 0.01	0.14 ± 0.03
Go-track-tracks	0.34 ± 0.04	0.19 ± 0.05	0.14 ± 0.07	0.10 ± 0.08	0.28 ± 0.13	0.28 ± 0.12	0.28 ± 0.12	0.19 ± 0.06	0.18 ± 0.05	0.24 ± 0.14
Haberman-survival	0.37 ± 0.01	0.33 ± 0.03	0.36 ± 0.03	0.34 ± 0.07	0.27 ± 0.05	0.29 ± 0.03	0.29 ± 0.03	0.35 ± 0.03	0.35 ± 0.03	0.27 ± 0.03
Hayes-roth	0.27 ± 0.02	0.29 ± 0.02	0.13 ± 0.03	0.09 ± 0.04	0.09 ± 0.05	0.13 ± 0.04	0.13 ± 0.04	0.22 ± 0.07	0.21 ± 0.07	0.37 ± 0.04
Hcc-data	0.45 ± 0.01	0.33 ± 0.06	0.43 ± 0.06	0.37 ± 0.08	0.38 ± 0.02	0.38 ± 0.02	0.38 ± 0.02	0.36 ± 0.12	0.36 ± 0.10	0.31 ± 0.11
Hcvdat	0.08 ± 0.00	0.04 ± 0.01	0.03 ± 0.01	0.04 ± 0.01	0.05 ± 0.00	0.05 ± 0.00	0.05 ± 0.00	0.03 ± 0.01	0.03 ± 0.01	0.05 ± 0.01
Heart-cleveland	0.24 ± 0.01	0.18 ± 0.03	0.21 ± 0.02	0.18 ± 0.03	0.17 ± 0.03	0.16 ± 0.04	0.17 ± 0.04	0.19 ± 0.02	0.18 ± 0.02	0.19 ± 0.03
Heart-hungarian	0.36 ± 0.02	0.19 ± 0.07	0.27 ± 0.05	0.23 ± 0.08	0.18 ± 0.08	0.18 ± 0.06	0.17 ± 0.06	0.23 ± 0.08	0.23 ± 0.07	0.21 ± 0.09
Heart-switzerland	0.28 ± 0.01	0.26 ± 0.02	0.26 ± 0.04	0.27 ± 0.05	0.24 ± 0.03	0.24 ± 0.02	0.23 ± 0.03	0.24 ± 0.05	0.23 ± 0.04	0.27 ± 0.05
Heart-va	0.30 ± 0.00	0.28 ± 0.02	0.28 ± 0.02	0.26 ± 0.03	0.26 ± 0.04	0.27 ± 0.03	0.28 ± 0.04	0.29 ± 0.02	0.29 ± 0.03	0.29 ± 0.04
Heart-failure-clinical-records-dataset	0.41 ± 0.01	0.25 ± 0.04	0.24 ± 0.05	0.35 ± 0.07	0.32 ± 0.02	0.32 ± 0.02	0.32 ± 0.02	0.27 ± 0.05	0.24 ± 0.08	0.14 ± 0.06
Hepatitis	0.30 ± 0.02	0.18 ± 0.08	0.24 ± 0.07	0.18 ± 0.05	0.17 ± 0.06	0.16 ± 0.09	0.16 ± 0.09	0.20 ± 0.09	0.22 ± 0.11	0.26 ± 0.07
Hill-valley	0.50 ± 0.01	0.51 ± 0.02	0.50 ± 0.00	0.48 ± 0.03	0.48 ± 0.07	0.48 ± 0.07	0.47 ± 0.07	0.46 ± 0.02	0.45 ± 0.02	0.52 ± 0.05
Hiv1625data	0.18 ± 0.01	0.07 ± 0.02	0.13 ± 0.02	0.11 ± 0.01	0.08 ± 0.02	0.07 ± 0.02	0.06 ± 0.02	0.05 ± 0.02	0.07 ± 0.02	0.21 ± 0.02
Hiv746data	0.25 ± 0.02	0.11 ± 0.03	0.25 ± 0.03	0.17 ± 0.03	0.10 ± 0.04	0.09 ± 0.03	0.09 ± 0.03	0.08 ± 0.02	0.08 ± 0.03	0.19 ± 0.04
Horse-colic	0.41 ± 0.01	0.27 ± 0.06	0.16 ± 0.04	0.24 ± 0.07	0.16 ± 0.05	0.15 ± 0.05	0.15 ± 0.06	0.23 ± 0.05	0.22 ± 0.07	0.18 ± 0.05
Htru	0.06 ± 0.00	0.06 ± 0.00	0.03 ± 0.00	0.03 ± 0.00	0.09 ± 0.00	0.09 ± 0.00	0.09 ± 0.00	0.03 ± 0.00	0.03 ± 0.00	0.02 ± 0.00
Hypothyroid	0.07 ± 0.00	0.04 ± 0.00	0.00 ± 0.00	0.04 ± 0.01	0.04 ± 0.00	0.04 ± 0.00	0.04 ± 0.00	0.04 ± 0.02	0.04 ± 0.03	0.02 ± 0.00
Ibeacon-rssi-labeled	0.02 ± 0.00	0.02 ± 0.00	0.01 ± 0.00	0.01 ± 0.00	0.01 ± 0.00	0.01 ± 0.00	0.01 ± 0.00	0.02 ± 0.00	0.02 ± 0.00	0.02 ± 0.00
Ilpd-indian-liver	0.39 ± 0.01	0.44 ± 0.05	0.39 ± 0.03	0.35 ± 0.06	0.29 ± 0.01	0.29 ± 0.02	0.29 ± 0.03	0.34 ± 0.03	0.34 ± 0.03	0.34 ± 0.03
Image-segmentation	0.15 ± 0.01	0.06 ± 0.02	0.04 ± 0.02	0.04 ± 0.01	0.04 ± 0.01	0.04 ± 0.01	0.04 ± 0.01	0.04 ± 0.01	0.04 ± 0.01	0.12 ± 0.03
Immunotherapy	0.30 ± 0.02	0.31 ± 0.06	0.23 ± 0.08	0.30 ± 0.16	0.21 ± 0.04	0.21 ± 0.04	0.21 ± 0.04	0.19 ± 0.08	0.20 ± 0.08	0.16 ± 0.09
Impensdata	0.21 ± 0.01	0.13 ± 0.02	0.27 ± 0.00	0.16 ± 0.02	0.16 ± 0.00	0.16 ± 0.00	0.16 ± 0.00	0.10 ± 0.02	0.11 ± 0.03	0.16 ± 0.02
In-vehicle-coupon-recommendation	0.48 ± 0.00	0.41 ± 0.01	0.38 ± 0.01	0.36 ± 0.01	0.31 ± 0.01	0.31 ± 0.01	0.30 ± 0.01	0.30 ± 0.01	0.29 ± 0.01	0.39 ± 0.01
Indian	0.38 ± 0.01	0.44 ± 0.05	0.33 ± 0.04	0.36 ± 0.07	0.28 ± 0.02	0.28 ± 0.01	0.28 ± 0.01	0.35 ± 0.03	0.34 ± 0.03	0.34 ± 0.03
Ionosphere	0.24 ± 0.02	0.17 ± 0.05	0.09 ± 0.03	0.14 ± 0.05	0.06 ± 0.02	0.05 ± 0.03	0.04 ± 0.03	0.10 ± 0.02	0.10 ± 0.03	0.19 ± 0.07
Iris	0.17 ± 0.02	0.04 ± 0.03	0.03 ± 0.03	0.04 ± 0.04	0.02 ± 0.02	0.03 ± 0.03	0.02 ± 0.02	0.03 ± 0.03	0.03 ± 0.02	0.05 ± 0.04
Jain	0.08 ± 0.03	0.07 ± 0.03	0.01 ± 0.02	0.00 ± 0.00	0.00 ± 0.00	0.00 ± 0.00	0.00 ± 0.00	0.07 ± 0.02	0.07 ± 0.02	0.05 ± 0.03
Jsbach-chorals-harmony	0.17 ± 0.00	0.05 ± 0.01	0.05 ± 0.01	0.03 ± 0.00	0.04 ± 0.01	0.03 ± 0.00	0.03 ± 0.01	0.03 ± 0.01	0.03 ± 0.01	0.04 ± 0.01
Knowledge	0.27 ± 0.01	0.13 ± 0.04	0.04 ± 0.03	0.13 ± 0.04	0.08 ± 0.05	0.06 ± 0.04	0.04 ± 0.04	0.05 ± 0.02	0.05 ± 0.03	0.09 ± 0.05
Lasvegastripadvisorreviews	0.24 ± 0.00	0.24 ± 0.01	0.24 ± 0.01	0.24 ± 0.01	0.25 ± 0.01	0.25 ± 0.01	0.25 ± 0.01	0.24 ± 0.01	0.24 ± 0.01	0.24 ± 0.01
Leaf	0.05 ± 0.00	0.02 ± 0.00	0.03 ± 0.00	0.06 ± 0.00	0.06 ± 0.00	0.05 ± 0.00	0.05 ± 0.00	0.04 ± 0.00	0.04 ± 0.00	0.05 ± 0.00
Led-display	0.15 ± 0.00	0.08 ± 0.00	0.08 ± 0.00	0.08 ± 0.00	0.06 ± 0.01	0.06 ± 0.01	0.06 ± 0.01	0.08 ± 0.00	0.07 ± 0.00	0.16 ± 0.00
Lenses	0.28 ± 0.07	0.37 ± 0.09	0.15 ± 0.19	0.13 ± 0.11	0.14 ± 0.20	0.11 ± 0.15	0.11 ± 0.15	0.19 ± 0.15	0.20 ± 0.16	0.22 ± 0.20
Letter	0.07 ± 0.00	0.03 ± 0.00	0.01 ± 0.00	0.00 ± 0.00	0.00 ± 0.00	0.00 ± 0.00	0.00 ± 0.00	0.02 ± 0.00	0.02 ± 0.00	0.06 ± 0.00
Libras	0.10 ± 0.00	0.05 ± 0.01	0.04 ± 0.01	0.02 ± 0.01	0.03 ± 0.01	0.02 ± 0.01	0.02 ± 0.01	0.03 ± 0.01	0.03 ± 0.01	0.11 ± 0.01
Low-res-spect	0.11 ± 0.00	0.04 ± 0.01	0.04 ± 0.01	0.04 ± 0.01	0.02 ± 0.01	0.02 ± 0.01	0.02 ± 0.01	0.02 ± 0.01	0.02 ± 0.01	0.06 ± 0.01
Lung-cancer	0.41 ± 0.02	0.25 ± 0.11	0.39 ± 0.16	0.34 ± 0.22	0.31 ± 0.15	0.27 ± 0.14	0.32 ± 0.17	0.35 ± 0.14	0.35 ± 0.15	0.35 ± 0.12
Lymphography	0.23 ± 0.00	0.10 ± 0.03	0.14 ± 0.04	0.13 ± 0.06	0.07 ± 0.05	0.07 ± 0.05	0.08 ± 0.04	0.10 ± 0.04	0.10 ± 0.03	0.12 ± 0.04
Magic	0.34 ± 0.01	0.28 ± 0.02	0.22 ± 0.02	0.20 ± 0.02	0.15 ± 0.02	0.15 ± 0.02	0.15 ± 0.01	0.19 ± 0.02	0.20 ± 0.02	0.28 ± 0.04
Mammographic	0.36 ± 0.01	0.24 ± 0.03	0.25 ± 0.02	0.26 ± 0.05	0.17 ± 0.02	0.18 ± 0.03	0.17 ± 0.03	0.25 ± 0.02	0.26 ± 0.03	0.18 ± 0.01
Miniboone	0.30 ± 0.00	0.71 ± 0.01	0.14 ± 0.02	0.17 ± 0.02	0.15 ± 0.02	0.14 ± 0.02	0.14 ± 0.02	0.21 ± 0.03	0.21 ± 0.03	0.18 ± 0.02
Molec-biol-promoter	0.45 ± 0.02	0.13 ± 0.08	0.27 ± 0.10	0.28 ± 0.12	0.17 ± 0.13	0.15 ± 0.12	0.15 ± 0.12	0.23 ± 0.09	0.23 ± 0.09	0.30 ± 0.11
Molec-biol-splice	0.40 ± 0.00	0.07 ± 0.01	0.06 ± 0.01	0.25 ± 0.01	0.09 ± 0.01	0.08 ± 0.01	0.09 ± 0.01	0.11 ± 0.01	0.11 ± 0.01	0.24 ± 0.02
Monks-1	0.39 ± 0.03	0.37 ± 0.08	0.04 ± 0.07	0.31 ± 0.09	0.19 ± 0.12	0.17 ± 0.11	0.17 ± 0.10	0.14 ± 0.14	0.12 ± 0.16	0.27 ± 0.10
Monks-2	0.47 ± 0.02	0.48 ± 0.03	0.27 ± 0.10	0.39 ± 0.11	0.42 ± 0.07	0.39 ± 0.13	0.36 ± 0.12	0.29 ± 0.09	0.27 ± 0.08	0.41 ± 0.05
Monks-3	0.37 ± 0.03	0.27 ± 0.04	0.11 ± 0.08	0.25 ± 0.15	0.10 ± 0.08	0.11 ± 0.08	0.11 ± 0.08	0.17 ± 0.10	0.13 ± 0.06	0.22 ± 0.04
Mushroom	0.33 ± 0.00	0.12 ± 0.01	0.00 ± 0.00	0.00 ± 0.00	0.00 ± 0.00	0.00 ± 0.00	0.00 ± 0.00	0.00 ± 0.00	0.00 ± 0.00	0.01 ± 0.00
Musk-1	0.40 ± 0.01	0.26 ± 0.07	0.16 ± 0.07	0.15 ± 0.05	0.09 ± 0.04	0.08 ± 0.03	0.06 ± 0.03	0.07 ± 0.02	0.07 ± 0.02	0.38 ± 0.09
Musk-2	0.20 ± 0.00	0.15 ± 0.02	0.05 ± 0.01	0.04 ± 0.01	0.04 ± 0.01	0.03 ± 0.01	0.02 ± 0.01	0.01 ± 0.01	0.01 ± 0.01	0.10 ± 0.01
Newdiagnosis	0.23 ± 0.02	0.10 ± 0.03	0.00 ± 0.00	0.00 ± 0.00	0.00 ± 0.00	0.00 ± 0.00	0.00 ± 0.00	0.01 ± 0.00	0.00 ± 0.00	0.22 ± 0.07
Nursery	0.24 ± 0.00	0.08 ± 0.00	0.00 ± 0.00	0.05 ± 0.00	0.01 ± 0.00	0.01 ± 0.00	0.00 ± 0.00	0.02 ± 0.01	0.03 ± 0.01	0.12 ± 0.00
Obesitydataset-raw-and-data-sinthetic	0.19 ± 0.00	0.11 ± 0.00	0.02 ± 0.01	0.05 ± 0.01	0.03 ± 0.01	0.02 ± 0.01	0.02 ± 0.01	0.02 ± 0.01	0.02 ± 0.00	0.09 ± 0.01
Obs-network-dataset-2-aug27	0.15 ± 0.01	0.14 ± 0.03	0.00 ± 0.01	0.01 ± 0.00	0.01 ± 0.00	0.01 ± 0.00	0.01 ± 0.00	0.03 ± 0.01	0.02 ± 0.01	0.05 ± 0.01
Occupancy-data	0.09 ± 0.01	0.05 ± 0.01	0.02 ± 0.01	0.01 ± 0.01	0.19 ± 0.02	0.18 ± 0.02	0.18 ± 0.02	0.04 ± 0.01	0.03 ± 0.01	0.01 ± 0.01
Occupancy-data2	0.07 ± 0.00	0.04 ± 0.00	0.01 ± 0.00	0.01 ± 0.00	0.16 ± 0.01	0.14 ± 0.01	0.14 ± 0.01	0.01 ± 0.00	0.01 ± 0.00	0.01 ± 0.00
Occupancy-data3	0.05 ± 0.00	0.02 ± 0.01	0.01 ± 0.00	0.01 ± 0.00	0.17 ± 0.01	0.15 ± 0.01	0.15 ± 0.01	0.01 ± 0.00	0.01 ± 0.00	0.01 ± 0.00
Old	0.42 ± 0.00	0.08 ± 0.01	0.06 ± 0.01	0.03 ± 0.01	0.05 ± 0.01	0.05 ± 0.01	0.05 ± 0.01	0.03 ± 0.01	0.03 ± 0.01	0.11 ± 0.02
Online-shoppers-intention	0.24 ± 0.00	0.23 ± 0.01	0.14 ± 0.00	0.18 ± 0.01	0.15 ± 0.00	0.16 ± 0.00	0.16 ± 0.00	0.13 ± 0.01	0.14 ± 0.02	0.12 ± 0.01
Oocytes-merluccius-nucleus-4d	0.39 ± 0.01	0.40 ± 0.04	0.29 ± 0.04	0.28 ± 0.04	0.23 ± 0.03	0.20 ± 0.03	0.19 ± 0.03	0.20 ± 0.03	0.21 ± 0.03	0.33 ± 0.05
Oocytes-merluccius-states-2f	0.17 ± 0.01	0.10 ± 0.02	0.07 ± 0.02	0.06 ± 0.02	0.06 ± 0.02	0.05 ± 0.01	0.05 ± 0.01	0.06 ± 0.01	0.06 ± 0.01	0.12 ± 0.03
Oocytes-trisopterus-nucleus-2f	0.44 ± 0.01	0.46 ± 0.05	0.30 ± 0.05	0.26 ± 0.06	0.19 ± 0.04	0.16 ± 0.03	0.17 ± 0.03	0.18 ± 0.03	0.19 ± 0.03	0.41 ± 0.06
Oocytes-trisopterus-states-5b	0.20 ± 0.02	0.16 ± 0.03	0.09 ± 0.02	0.06 ± 0.02	0.05 ± 0.02	0.05 ± 0.02	0.05 ± 0.02	0.05 ± 0.02	0.05 ± 0.02	0.13 ± 0.02
Optdigits	0.16 ± 0.00	0.02 ± 0.00	0.02 ± 0.00	0.00 ± 0.00	0.05 ± 0.00	0.05 ± 0.00	0.05 ± 0.00	0.01 ± 0.00	0.00 ± 0.00	0.15 ± 0.00
Optical	0.16 ± 0.00	0.02 ± 0.00	0.02 ± 0.00	0.00 ± 0.00	0.00 ± 0.00	0.00 ± 0.00	0.00 ± 0.00	0.01 ± 0.00	0.01 ± 0.00	0.14 ± 0.00
Ozone	0.06 ± 0.00	0.29 ± 0.04	0.05 ± 0.01	0.05 ± 0.01	0.03 ± 0.00	0.03 ± 0.00	0.03 ± 0.00	0.04 ± 0.01	0.03 ± 0.01	0.03 ± 0.00
Page-blocks	0.05 ± 0.00	0.04 ± 0.01	0.02 ± 0.00	0.02 ± 0.00	0.02 ± 0.00	0.01 ± 0.00	0.01 ± 0.00	0.02 ± 0.00	0.02 ± 0.00	0.02 ± 0.00
Parkingbirmingham	0.01 ± 0.00	0.02 ± 0.00	0.00 ± 0.00	0.00 ± 0.00	0.03 ± 0.00	0.03 ± 0.00	0.03 ± 0.00	0.02 ± 0.00	0.02 ± 0.00	0.00 ± 0.00
Parkinsons	0.28 ± 0.03	0.31 ± 0.11	0.20 ± 0.03	0.04 ± 0.02	0.12 ± 0.06	0.10 ± 0.06	0.09 ± 0.06	0.12 ± 0.06	0.11 ± 0.07	0.14 ± 0.08
Pasture	0.37 ± 0.04	0.18 ± 0.15	0.16 ± 0.13	0.21 ± 0.17	0.48 ± 0.03	0.48 ± 0.03	0.48 ± 0.03	0.19 ± 0.16	0.20 ± 0.16	0.24 ± 0.18
Pbc	0.40 ± 0.01	0.24 ± 0.06	0.29 ± 0.06	0.40 ± 0.09	0.39 ± 0.01	0.39 ± 0.01	0.39 ± 0.01	0.29 ± 0.05	0.27 ± 0.03	0.28 ± 0.09
Pen	0.12 ± 0.00	0.03 ± 0.00	0.01 ± 0.00	0.00 ± 0.00	0.17 ± 0.00	0.17 ± 0.00	0.17 ± 0.00	0.01 ± 0.00	0.01 ± 0.00	0.12 ± 0.00
Pendigits	0.12 ± 0.00	0.03 ± 0.00	0.01 ± 0.00	0.00 ± 0.00	0.00 ± 0.00	0.00 ± 0.00	0.00 ± 0.00	0.01 ± 0.00	0.01 ± 0.00	0.12 ± 0.00
Pharynx	0.36 ± 0.02	0.39 ± 0.04	0.36 ± 0.01	0.36 ± 0.10	0.25 ± 0.09	0.27 ± 0.11	0.30 ± 0.11	0.29 ± 0.04	0.30 ± 0.16	0.73 ± 0.06
Phishingwebsites	0.43 ± 0.00	0.09 ± 0.01	0.06 ± 0.00	0.03 ± 0.01	0.05 ± 0.01	0.05 ± 0.01	0.05 ± 0.00	0.03 ± 0.00	0.03 ± 0.00	0.11 ± 0.01
Pima	0.39 ± 0.01	0.28 ± 0.03	0.32 ± 0.05	0.30 ± 0.05	0.24 ± 0.04	0.25 ± 0.04	0.25 ± 0.03	0.30 ± 0.02	0.29 ± 0.02	0.29 ± 0.05
Pittsburg-bridges-rel-l	0.34 ± 0.02	0.26 ± 0.06	0.31 ± 0.07	0.20 ± 0.11	0.22 ± 0.08	0.21 ± 0.08	0.18 ± 0.07	0.27 ± 0.07	0.26 ± 0.07	0.19 ± 0.07
Pittsburg-bridges-span	0.35 ± 0.01	0.26 ± 0.05	0.30 ± 0.07	0.30 ± 0.10	0.23 ± 0.10	0.24 ± 0.11	0.24 ± 0.11	0.24 ± 0.04	0.22 ± 0.07	0.30 ± 0.09
Pittsburg-bridges-t-or-d	0.22 ± 0.04	0.20 ± 0.07	0.21 ± 0.09	0.17 ± 0.12	0.14 ± 0.08	0.12 ± 0.08	0.11 ± 0.08	0.15 ± 0.12	0.16 ± 0.13	0.15 ± 0.10
Pittsburg-bridges-type	0.23 ± 0.01	0.17 ± 0.02	0.17 ± 0.02	0.15 ± 0.04	0.15 ± 0.04	0.13 ± 0.06	0.13 ± 0.04	0.15 ± 0.05	0.15 ± 0.05	0.15 ± 0.02
Pittsburg-bridgesmaterial	0.21 ± 0.02	0.13 ± 0.05	0.13 ± 0.04	0.12 ± 0.07	0.09 ± 0.04	0.10 ± 0.04	0.11 ± 0.06	0.13 ± 0.06	0.15 ± 0.06	0.09 ± 0.03
Planning	0.41 ± 0.01	0.43 ± 0.03	0.41 ± 0.01	0.34 ± 0.09	0.29 ± 0.02	0.30 ± 0.06	0.34 ± 0.07	0.41 ± 0.05	0.43 ± 0.08	0.38 ± 0.09
Plant-margin	0.02 ± 0.00	0.00 ± 0.00	0.01 ± 0.00	0.01 ± 0.00	0.00 ± 0.00	0.00 ± 0.00	0.00 ± 0.00	0.00 ± 0.00	0.00 ± 0.00	0.02 ± 0.00
Plant-shape	0.02 ± 0.00	0.01 ± 0.00	0.01 ± 0.00	0.01 ± 0.00	0.01 ± 0.00	0.01 ± 0.00	0.01 ± 0.00	0.01 ± 0.00	0.01 ± 0.00	0.02 ± 0.00
Plant-texture	0.02 ± 0.00	0.01 ± 0.00	0.01 ± 0.00	0.01 ± 0.00	0.00 ± 0.00	0.00 ± 0.00	0.00 ± 0.00	0.00 ± 0.00	0.00 ± 0.00	0.02 ± 0.00
Poker-hand-training-true	0.11 ± 0.00	0.11 ± 0.00	0.10 ± 0.00	0.11 ± 0.00	0.08 ± 0.00	0.08 ± 0.00	0.09 ± 0.00	0.11 ± 0.00	0.11 ± 0.00	0.10 ± 0.00
Post-operative	0.28 ± 0.02	0.30 ± 0.04	0.28 ± 0.02	0.29 ± 0.07	0.19 ± 0.04	0.21 ± 0.05	0.22 ± 0.06	0.28 ± 0.07	0.29 ± 0.08	0.21 ± 0.05
Primary-tumor	0.11 ± 0.00	0.08 ± 0.01	0.09 ± 0.01	0.09 ± 0.01	0.07 ± 0.01	0.07 ± 0.01	0.07 ± 0.02	0.08 ± 0.01	0.08 ± 0.01	0.10 ± 0.00
Qsarbioconcentration	0.38 ± 0.00	0.38 ± 0.02	0.39 ± 0.02	0.38 ± 0.06	0.25 ± 0.01	0.27 ± 0.02	0.29 ± 0.03	0.39 ± 0.02	0.39 ± 0.02	0.27 ± 0.03
Qsarbiodegradation	0.39 ± 0.01	0.24 ± 0.03	0.19 ± 0.03	0.16 ± 0.02	0.14 ± 0.03	0.14 ± 0.02	0.14 ± 0.02	0.14 ± 0.02	0.14 ± 0.02	0.23 ± 0.04
Qualitative-bankruptcy	0.13 ± 0.01	0.01 ± 0.02	0.02 ± 0.03	0.00 ± 0.00	0.01 ± 0.03	0.01 ± 0.03	0.00 ± 0.01	0.01 ± 0.02	0.01 ± 0.02	0.02 ± 0.03
Ringnorm	0.30 ± 0.01	0.02 ± 0.00	0.09 ± 0.00	0.25 ± 0.02	0.01 ± 0.00	0.02 ± 0.00	0.02 ± 0.00	0.08 ± 0.01	0.09 ± 0.01	0.36 ± 0.01
Risk-factors-cervical-cancer	0.12 ± 0.01	0.11 ± 0.01	0.06 ± 0.02	0.06 ± 0.02	0.06 ± 0.01	0.07 ± 0.01	0.06 ± 0.02	0.05 ± 0.02	0.05 ± 0.02	0.04 ± 0.02
Robotnavigation	0.16 ± 0.00	0.24 ± 0.01	0.00 ± 0.00	0.06 ± 0.01	0.05 ± 0.01	0.04 ± 0.00	0.04 ± 0.00	0.07 ± 0.01	0.06 ± 0.01	0.12 ± 0.01
Sapfile	0.41 ± 0.01	0.33 ± 0.04	0.40 ± 0.08	0.41 ± 0.06	0.34 ± 0.06	0.34 ± 0.05	0.34 ± 0.06	0.35 ± 0.05	0.35 ± 0.07	0.39 ± 0.08
Sat	0.14 ± 0.00	0.07 ± 0.00	0.05 ± 0.00	0.03 ± 0.00	0.25 ± 0.00	0.24 ± 0.00	0.24 ± 0.00	0.04 ± 0.00	0.04 ± 0.00	0.13 ± 0.00
Satelite	0.14 ± 0.00	0.07 ± 0.00	0.05 ± 0.00	0.03 ± 0.00	0.25 ± 0.00	0.24 ± 0.00	0.24 ± 0.00	0.04 ± 0.00	0.04 ± 0.00	0.13 ± 0.01
Scadi	0.18 ± 0.01	0.05 ± 0.02	0.07 ± 0.03	0.08 ± 0.04	0.07 ± 0.03	0.06 ± 0.04	0.05 ± 0.04	0.06 ± 0.03	0.06 ± 0.03	0.12 ± 0.03
Schillingdata	0.19 ± 0.00	0.10 ± 0.01	0.23 ± 0.00	0.15 ± 0.01	0.13 ± 0.00	0.11 ± 0.01	0.08 ± 0.01	0.07 ± 0.02	0.09 ± 0.03	0.13 ± 0.00
Seeds	0.19 ± 0.03	0.06 ± 0.03	0.07 ± 0.04	0.04 ± 0.01	0.04 ± 0.03	0.04 ± 0.03	0.04 ± 0.03	0.04 ± 0.01	0.04 ± 0.01	0.12 ± 0.07
Segment	0.13 ± 0.00	0.06 ± 0.00	0.01 ± 0.00	0.01 ± 0.00	0.10 ± 0.01	0.09 ± 0.01	0.09 ± 0.01	0.02 ± 0.00	0.01 ± 0.00	0.10 ± 0.01
Seismic-bumps	0.12 ± 0.00	0.14 ± 0.02	0.12 ± 0.00	0.11 ± 0.01	0.07 ± 0.00	0.07 ± 0.00	0.07 ± 0.00	0.10 ± 0.01	0.09 ± 0.01	0.07 ± 0.01
Semeion	0.17 ± 0.00	0.03 ± 0.01	0.05 ± 0.00	0.02 ± 0.00	0.01 ± 0.00	0.01 ± 0.00	0.01 ± 0.00	0.02 ± 0.00	0.02 ± 0.00	0.16 ± 0.00
Setapprocesst1	0.40 ± 0.03	0.42 ± 0.19	0.38 ± 0.18	0.35 ± 0.10	0.36 ± 0.08	0.31 ± 0.11	0.33 ± 0.09	0.36 ± 0.19	0.37 ± 0.19	0.28 ± 0.23
Setapprocesst10	0.44 ± 0.02	0.56 ± 0.15	0.42 ± 0.14	0.40 ± 0.16	0.34 ± 0.06	0.34 ± 0.06	0.34 ± 0.06	0.47 ± 0.24	0.47 ± 0.21	0.38 ± 0.08
Setapprocesst11	0.44 ± 0.02	0.45 ± 0.25	0.36 ± 0.17	0.42 ± 0.16	0.34 ± 0.06	0.34 ± 0.06	0.34 ± 0.06	0.39 ± 0.15	0.35 ± 0.16	0.43 ± 0.11
Setapprocesst2	0.44 ± 0.02	0.33 ± 0.15	0.27 ± 0.18	0.38 ± 0.21	0.34 ± 0.06	0.34 ± 0.06	0.34 ± 0.06	0.38 ± 0.17	0.35 ± 0.17	0.19 ± 0.17
Setapprocesst3	0.44 ± 0.01	0.35 ± 0.12	0.40 ± 0.15	0.34 ± 0.13	0.34 ± 0.06	0.34 ± 0.06	0.34 ± 0.06	0.35 ± 0.12	0.35 ± 0.08	0.47 ± 0.16
Setapprocesst4	0.41 ± 0.03	0.51 ± 0.24	0.45 ± 0.14	0.40 ± 0.18	0.29 ± 0.07	0.29 ± 0.07	0.29 ± 0.07	0.45 ± 0.19	0.43 ± 0.16	0.27 ± 0.12
Setapprocesst5	0.43 ± 0.03	0.53 ± 0.17	0.47 ± 0.17	0.41 ± 0.17	0.35 ± 0.08	0.36 ± 0.09	0.36 ± 0.09	0.41 ± 0.13	0.39 ± 0.13	0.34 ± 0.25
Setapprocesst6	0.44 ± 0.01	0.33 ± 0.16	0.35 ± 0.12	0.36 ± 0.14	0.34 ± 0.06	0.34 ± 0.06	0.34 ± 0.06	0.37 ± 0.15	0.37 ± 0.14	0.40 ± 0.20
Setapprocesst7	0.44 ± 0.03	0.26 ± 0.14	0.39 ± 0.20	0.36 ± 0.15	0.34 ± 0.06	0.34 ± 0.06	0.34 ± 0.06	0.28 ± 0.18	0.29 ± 0.18	0.47 ± 0.15
Setapprocesst8	0.43 ± 0.02	0.32 ± 0.14	0.30 ± 0.15	0.37 ± 0.15	0.34 ± 0.06	0.34 ± 0.06	0.34 ± 0.06	0.34 ± 0.16	0.34 ± 0.17	0.51 ± 0.11
Setapprocesst9	0.44 ± 0.02	0.35 ± 0.15	0.32 ± 0.16	0.42 ± 0.10	0.34 ± 0.06	0.34 ± 0.06	0.34 ± 0.06	0.29 ± 0.18	0.33 ± 0.16	0.49 ± 0.17
Shillbiddingdataset	0.12 ± 0.00	0.04 ± 0.00	0.01 ± 0.00	0.01 ± 0.00	0.00 ± 0.00	0.00 ± 0.00	0.00 ± 0.00	0.00 ± 0.00	0.00 ± 0.00	0.03 ± 0.01
Shuttle-landing-control	0.16 ± 0.17	0.23 ± 0.29	0.12 ± 0.13	0.14 ± 0.19	0.05 ± 0.16	0.05 ± 0.16	0.05 ± 0.16	0.16 ± 0.27	0.16 ± 0.29	0.15 ± 0.34
Somervillehappinesssurvey2015	0.47 ± 0.03	0.44 ± 0.06	0.41 ± 0.10	0.42 ± 0.10	0.40 ± 0.11	0.41 ± 0.10	0.41 ± 0.12	0.42 ± 0.11	0.43 ± 0.11	0.34 ± 0.16
Sonar	0.45 ± 0.01	0.31 ± 0.09	0.29 ± 0.07	0.14 ± 0.07	0.34 ± 0.05	0.30 ± 0.09	0.26 ± 0.10	0.19 ± 0.10	0.19 ± 0.09	0.37 ± 0.11
Soybean	0.09 ± 0.00	0.01 ± 0.01	0.02 ± 0.01	0.02 ± 0.01	0.01 ± 0.01	0.01 ± 0.00	0.01 ± 0.01	0.02 ± 0.01	0.02 ± 0.01	0.08 ± 0.00
Spambase	0.40 ± 0.00	0.21 ± 0.02	0.09 ± 0.01	0.09 ± 0.01	0.07 ± 0.01	0.06 ± 0.01	0.07 ± 0.01	0.11 ± 0.01	0.13 ± 0.06	0.22 ± 0.02
Speaker-accent	0.21 ± 0.01	0.15 ± 0.03	0.11 ± 0.03	0.07 ± 0.03	0.14 ± 0.01	0.13 ± 0.01	0.13 ± 0.01	0.08 ± 0.02	0.09 ± 0.02	0.17 ± 0.02
Spect	0.42 ± 0.03	0.31 ± 0.14	0.38 ± 0.09	0.49 ± 0.12	0.35 ± 0.11	0.36 ± 0.12	0.37 ± 0.09	0.46 ± 0.17	0.47 ± 0.16	0.28 ± 0.10
Spectf	0.47 ± 0.02	0.25 ± 0.12	0.28 ± 0.11	0.33 ± 0.18	0.32 ± 0.06	0.32 ± 0.06	0.24 ± 0.14	0.30 ± 0.14	0.29 ± 0.13	0.29 ± 0.08
Statlog-australian-credit	0.43 ± 0.00	0.46 ± 0.02	0.41 ± 0.05	0.44 ± 0.03	0.32 ± 0.02	0.36 ± 0.06	0.36 ± 0.06	0.41 ± 0.03	0.41 ± 0.03	0.35 ± 0.06
Statlog-german-credit	0.41 ± 0.00	0.29 ± 0.03	0.31 ± 0.04	0.32 ± 0.04	0.24 ± 0.04	0.23 ± 0.04	0.23 ± 0.04	0.30 ± 0.04	0.28 ± 0.03	0.29 ± 0.02
Statlog-heart	0.40 ± 0.02	0.19 ± 0.06	0.28 ± 0.08	0.25 ± 0.08	0.17 ± 0.05	0.20 ± 0.08	0.20 ± 0.10	0.22 ± 0.06	0.21 ± 0.08	0.29 ± 0.06
Statlog-image	0.12 ± 0.00	0.06 ± 0.00	0.01 ± 0.00	0.01 ± 0.00	0.02 ± 0.00	0.01 ± 0.00	0.01 ± 0.00	0.02 ± 0.00	0.02 ± 0.00	0.10 ± 0.01
Statlog-landsat	0.14 ± 0.00	0.07 ± 0.01	0.05 ± 0.01	0.03 ± 0.01	0.04 ± 0.01	0.03 ± 0.00	0.03 ± 0.01	0.04 ± 0.00	0.04 ± 0.01	0.13 ± 0.01
Statlog-shuttle	0.07 ± 0.00	0.03 ± 0.00	0.00 ± 0.00	0.00 ± 0.00	0.00 ± 0.00	0.00 ± 0.00	0.00 ± 0.00	0.00 ± 0.00	0.00 ± 0.00	0.02 ± 0.00
Statlog-vehicle	0.29 ± 0.01	0.28 ± 0.01	0.14 ± 0.02	0.15 ± 0.02	0.12 ± 0.02	0.10 ± 0.01	0.10 ± 0.01	0.10 ± 0.01	0.10 ± 0.02	0.24 ± 0.03
Steel-plates	0.17 ± 0.00	0.12 ± 0.01	0.07 ± 0.01	0.08 ± 0.01	0.07 ± 0.01	0.07 ± 0.01	0.07 ± 0.01	0.08 ± 0.01	0.08 ± 0.01	0.15 ± 0.01
Synthetic-control	0.19 ± 0.00	0.02 ± 0.01	0.03 ± 0.01	0.01 ± 0.01	0.00 ± 0.00	0.00 ± 0.00	0.00 ± 0.00	0.01 ± 0.00	0.01 ± 0.00	0.14 ± 0.01
Teaching	0.40 ± 0.02	0.38 ± 0.03	0.29 ± 0.07	0.25 ± 0.09	0.29 ± 0.10	0.26 ± 0.08	0.25 ± 0.08	0.36 ± 0.03	0.36 ± 0.03	0.36 ± 0.07
Thoraricsurgery	0.25 ± 0.00	0.28 ± 0.08	0.25 ± 0.00	0.23 ± 0.06	0.15 ± 0.00	0.16 ± 0.01	0.16 ± 0.01	0.24 ± 0.04	0.22 ± 0.04	0.17 ± 0.02
Thyroid	0.18 ± 0.03	0.02 ± 0.02	0.06 ± 0.04	0.03 ± 0.02	0.16 ± 0.03	0.15 ± 0.03	0.15 ± 0.03	0.03 ± 0.02	0.03 ± 0.02	0.06 ± 0.06
Thyroid-train	0.09 ± 0.00	0.04 ± 0.01	0.00 ± 0.00	0.05 ± 0.01	0.03 ± 0.00	0.03 ± 0.00	0.02 ± 0.00	0.03 ± 0.01	0.03 ± 0.01	0.02 ± 0.01
Tic-tac-toe	0.44 ± 0.00	0.37 ± 0.01	0.07 ± 0.02	0.00 ± 0.00	0.01 ± 0.01	0.01 ± 0.01	0.00 ± 0.01	0.03 ± 0.01	0.02 ± 0.01	0.30 ± 0.02
Titanic	0.37 ± 0.01	0.31 ± 0.02	0.31 ± 0.01	0.31 ± 0.01	0.22 ± 0.02	0.21 ± 0.02	0.21 ± 0.02	0.32 ± 0.02	0.32 ± 0.03	0.22 ± 0.02
Trains	0.46 ± 0.08	0.40 ± 0.52	0.28 ± 0.46	0.42 ± 0.42	0.40 ± 0.52	0.30 ± 0.48	0.30 ± 0.48	0.28 ± 0.37	0.27 ± 0.37	1.00 ± 0.00
Transfusion	0.35 ± 0.01	0.29 ± 0.02	0.30 ± 0.01	0.32 ± 0.04	0.25 ± 0.03	0.27 ± 0.03	0.28 ± 0.04	0.30 ± 0.02	0.30 ± 0.02	0.24 ± 0.02
Trial	0.32 ± 0.02	0.10 ± 0.03	0.00 ± 0.00	0.00 ± 0.01	0.01 ± 0.01	0.01 ± 0.01	0.01 ± 0.01	0.01 ± 0.01	0.01 ± 0.01	0.00 ± 0.00
Turkiye-student-evaluation	0.34 ± 0.00	0.10 ± 0.01	0.00 ± 0.00	0.06 ± 0.01	0.03 ± 0.00	0.02 ± 0.00	0.02 ± 0.00	0.00 ± 0.00	0.00 ± 0.00	0.00 ± 0.00
Unbalanced	0.03 ± 0.00	0.11 ± 0.04	0.03 ± 0.00	0.02 ± 0.02	0.01 ± 0.00	0.02 ± 0.01	0.02 ± 0.01	0.03 ± 0.01	0.02 ± 0.01	0.01 ± 0.00
Urbanlandcover	0.16 ± 0.01	0.05 ± 0.02	0.05 ± 0.01	0.06 ± 0.02	0.18 ± 0.00	0.18 ± 0.00	0.18 ± 0.00	0.05 ± 0.02	0.05 ± 0.02	0.11 ± 0.03
Userknowledgemodeling	0.27 ± 0.02	0.11 ± 0.02	0.04 ± 0.02	0.10 ± 0.05	0.11 ± 0.03	0.07 ± 0.02	0.07 ± 0.02	0.05 ± 0.02	0.04 ± 0.02	0.07 ± 0.04
Vehicle	0.29 ± 0.01	0.28 ± 0.01	0.14 ± 0.02	0.15 ± 0.02	0.35 ± 0.01	0.34 ± 0.02	0.34 ± 0.02	0.10 ± 0.01	0.10 ± 0.02	0.24 ± 0.03
Vertebral-column-2classes	0.34 ± 0.04	0.22 ± 0.07	0.21 ± 0.06	0.18 ± 0.05	0.15 ± 0.05	0.15 ± 0.06	0.15 ± 0.06	0.18 ± 0.04	0.18 ± 0.04	0.26 ± 0.08
Vertebral-column-3classes	0.28 ± 0.02	0.14 ± 0.02	0.13 ± 0.04	0.15 ± 0.03	0.11 ± 0.04	0.10 ± 0.05	0.11 ± 0.04	0.13 ± 0.02	0.13 ± 0.02	0.17 ± 0.05
Veteran	0.39 ± 0.01	0.31 ± 0.05	0.34 ± 0.04	0.40 ± 0.09	0.29 ± 0.01	0.29 ± 0.01	0.29 ± 0.01	0.39 ± 0.12	0.34 ± 0.12	0.26 ± 0.08
Vowel	0.13 ± 0.00	0.09 ± 0.01	0.04 ± 0.01	0.00 ± 0.00	0.03 ± 0.00	0.02 ± 0.00	0.01 ± 0.00	0.02 ± 0.01	0.02 ± 0.00	0.12 ± 0.01
Wall-following	0.16 ± 0.00	0.24 ± 0.00	0.00 ± 0.00	0.06 ± 0.01	0.05 ± 0.01	0.05 ± 0.01	0.04 ± 0.01	0.07 ± 0.01	0.07 ± 0.01	0.12 ± 0.01
Waveform-noise	0.37 ± 0.00	0.14 ± 0.01	0.17 ± 0.01	0.18 ± 0.01	0.09 ± 0.01	0.10 ± 0.01	0.10 ± 0.01	0.11 ± 0.01	0.11 ± 0.01	0.31 ± 0.02
Waveform	0.34 ± 0.00	0.13 ± 0.01	0.17 ± 0.01	0.15 ± 0.01	0.09 ± 0.01	0.10 ± 0.01	0.10 ± 0.01	0.11 ± 0.01	0.11 ± 0.01	0.32 ± 0.01
Wbc	0.10 ± 0.01	0.03 ± 0.01	0.09 ± 0.02	0.05 ± 0.02	0.02 ± 0.02	0.02 ± 0.02	0.02 ± 0.02	0.04 ± 0.01	0.04 ± 0.02	0.09 ± 0.03
Wdbc	0.25 ± 0.02	0.07 ± 0.04	0.07 ± 0.04	0.04 ± 0.02	0.37 ± 0.01	0.37 ± 0.01	0.37 ± 0.01	0.03 ± 0.02	0.04 ± 0.02	0.10 ± 0.03
Weathernominal	0.45 ± 0.15	0.42 ± 0.16	0.36 ± 0.34	0.48 ± 0.24	0.30 ± 0.35	0.40 ± 0.39	0.25 ± 0.35	0.25 ± 0.35	0.25 ± 0.35	0.55 ± 0.44
Weathernumeric	0.55 ± 0.16	0.45 ± 0.18	0.24 ± 0.33	0.24 ± 0.30	0.30 ± 0.35	0.50 ± 0.47	0.50 ± 0.47	0.28 ± 0.39	0.29 ± 0.40	0.55 ± 0.44
Website-phishingdata	0.29 ± 0.00	0.14 ± 0.01	0.08 ± 0.01	0.09 ± 0.01	0.10 ± 0.01	0.09 ± 0.01	0.08 ± 0.01	0.09 ± 0.01	0.08 ± 0.01	0.12 ± 0.01
Wholesalecustomersdata	0.23 ± 0.02	0.12 ± 0.03	0.13 ± 0.03	0.12 ± 0.05	0.32 ± 0.01	0.32 ± 0.01	0.32 ± 0.01	0.11 ± 0.03	0.11 ± 0.03	0.10 ± 0.04
Wifi-localization	0.20 ± 0.00	0.01 ± 0.00	0.02 ± 0.00	0.01 ± 0.00	0.10 ± 0.01	0.10 ± 0.01	0.10 ± 0.01	0.01 ± 0.00	0.01 ± 0.00	0.11 ± 0.02
Wilt	0.10 ± 0.00	0.17 ± 0.01	0.02 ± 0.00	0.05 ± 0.01	0.05 ± 0.00	0.05 ± 0.00	0.05 ± 0.00	0.03 ± 0.00	0.02 ± 0.00	0.06 ± 0.00
Wine-quality-red	0.18 ± 0.00	0.18 ± 0.01	0.14 ± 0.01	0.12 ± 0.01	0.12 ± 0.01	0.12 ± 0.01	0.12 ± 0.01	0.17 ± 0.01	0.16 ± 0.01	0.15 ± 0.01
Wine-quality-white	0.17 ± 0.00	0.17 ± 0.00	0.13 ± 0.01	0.10 ± 0.01	0.12 ± 0.01	0.12 ± 0.01	0.12 ± 0.01	0.16 ± 0.00	0.16 ± 0.00	0.15 ± 0.01
Wine	0.23 ± 0.01	0.02 ± 0.03	0.05 ± 0.03	0.04 ± 0.03	0.01 ± 0.02	0.01 ± 0.02	0.01 ± 0.02	0.02 ± 0.02	0.02 ± 0.02	0.15 ± 0.06
Yamilnaduelectricty	0.09 ± 0.00	0.09 ± 0.00	0.09 ± 0.00	0.08 ± 0.00	0.09 ± 0.00	0.09 ± 0.00	0.09 ± 0.00	0.09 ± 0.00	0.09 ± 0.00	0.09 ± 0.00
Yeast	0.14 ± 0.00	0.10 ± 0.00	0.10 ± 0.01	0.10 ± 0.00	0.08 ± 0.01	0.08 ± 0.01	0.08 ± 0.01	0.10 ± 0.00	0.10 ± 0.00	0.12 ± 0.01
Youtobe-kabita-preprocessing	0.23 ± 0.00	0.21 ± 0.00	0.21 ± 0.00	0.20 ± 0.00	0.18 ± 0.01	0.18 ± 0.01	0.18 ± 0.01	0.22 ± 0.00	0.21 ± 0.00	0.19 ± 0.01
Youtobe-nisha-preprocessing	0.22 ± 0.00	0.21 ± 0.00	0.20 ± 0.00	0.20 ± 0.00	0.17 ± 0.01	0.17 ± 0.01	0.17 ± 0.01	0.21 ± 0.00	0.21 ± 0.00	0.20 ± 0.00
Z-alizadehsani	0.39 ± 0.01	0.20 ± 0.06	0.24 ± 0.08	0.22 ± 0.07	0.29 ± 0.01	0.29 ± 0.01	0.29 ± 0.01	0.17 ± 0.07	0.18 ± 0.06	0.29 ± 0.05
Zoo	0.14 ± 0.01	0.01 ± 0.02	0.02 ± 0.02	0.02 ± 0.01	0.02 ± 0.02	0.02 ± 0.02	0.02 ± 0.02	0.02 ± 0.02	0.02 ± 0.02	0.08 ± 0.03
Average mae (rank)	0.2672 (10)	0.1947 (9)	0.1550 (3)	0.1576 (4)	0.1695 (7)	0.1662 (6)	0.1643 (5)	0.1494 (2)	0.1476 (1)	0.1913 (8)
Average mae std (rank)	0.0120 (1)	0.0374 (6)	0.0372 (5)	0.0431 (9)	0.0318 (2)	0.0345 (3)	0.0350 (4)	0.0400 (7)	0.0413 (8)	0.0438 (10)

**Table 10:** The detailed experimental results on relative absolute error and standard deviation.

Dataset	RN	NB	J48	KNN	SVM1	SVM2	SVM3	ANN1	ANN2	OneR
Abalone	75.92 ± 1.42	64.51 ± 3.17	64.97 ± 1.91	63.17 ± 3.75	50.10 ± 3.70	49.56 ± 3.54	49.63 ± 3.38	65.50 ± 2.15	64.96 ± 2.45	58.61 ± 2.68
Absenteeism-at-work	62.76 ± 1.26	33.47 ± 5.17	0.73 ± 1.25	33.50 ± 5.85	31.11 ± 6.09	31.11 ± 6.09	31.11 ± 6.09	7.25 ± 2.91	7.28 ± 3.18	9.63 ± 3.49
Acute-inflammation	46.10 ± 4.81	19.62 ± 5.03	0.00 ± 0.00	1.64 ± 0.10	0.00 ± 0.00	0.00 ± 0.00	0.00 ± 0.00	1.10 ± 0.10	0.79 ± 0.08	41.67 ± 16.20
Acute-nephritis	43.11 ± 2.40	10.82 ± 11.20	0.00 ± 0.00	1.66 ± 0.10	0.00 ± 0.00	0.00 ± 0.00	0.00 ± 0.00	1.12 ± 0.14	0.90 ± 0.18	17.13 ± 16.15
Adult	94.23 ± 1.18	55.36 ± 6.78	60.24 ± 7.54	59.42 ± 9.83	45.84 ± 11.29	45.50 ± 11.36	45.67 ± 11.76	58.87 ± 7.66	58.37 ± 7.45	53.92 ± 3.56
Aggregation	17.56 ± 6.47	1.06 ± 0.54	0.48 ± 0.78	1.23 ± 0.52	0.16 ± 0.52	0.33 ± 0.69	0.33 ± 0.69	7.37 ± 2.22	6.05 ± 2.49	51.54 ± 3.72
Algerianforest	61.49 ± 4.72	12.11 ± 10.35	9.21 ± 8.93	24.91 ± 13.53	83.64 ± 3.36	77.76 ± 8.52	77.76 ± 8.52	8.33 ± 6.21	7.25 ± 7.42	5.78 ± 9.62
Annealing	91.67 ± 0.60	76.69 ± 9.34	10.96 ± 5.44	27.17 ± 5.51	27.39 ± 7.85	21.16 ± 6.34	19.61 ± 5.29	25.69 ± 8.98	24.05 ± 6.96	40.47 ± 0.10
Arrhythmia	96.40 ± 0.43	56.91 ± 10.57	56.76 ± 10.48	70.08 ± 4.75	56.02 ± 3.65	50.52 ± 5.06	51.82 ± 6.11	50.80 ± 7.65	66.27 ± 20.92	59.26 ± 3.73
Au1-1000	99.01 ± 0.36	92.85 ± 2.65	77.84 ± 7.92	84.06 ± 11.34	67.43 ± 0.59	67.43 ± 0.59	66.65 ± 2.15	81.90 ± 9.76	75.90 ± 9.28	67.43 ± 0.59
Au4-2500	95.13 ± 0.73	83.45 ± 2.61	52.13 ± 9.35	64.38 ± 7.72	67.92 ± 4.82	67.92 ± 4.82	67.92 ± 4.82	62.96 ± 6.80	62.81 ± 7.92	81.84 ± 7.39
Au6-1000	99.65 ± 0.37	99.20 ± 0.74	94.38 ± 4.26	100.82 ± 4.36	88.47 ± 0.01	88.47 ± 0.01	88.47 ± 0.01	99.67 ± 3.16	100.64 ± 4.46	88.13 ± 2.58
Au6-250-drift-au6-cd1-500	99.81 ± 0.54	98.95 ± 1.65	95.38 ± 4.14	97.98 ± 4.88	91.22 ± 0.76	91.22 ± 0.76	91.22 ± 0.76	99.52 ± 2.11	98.83 ± 3.99	88.26 ± 4.02
Au6-cd1-400	99.51 ± 0.58	95.72 ± 3.69	77.36 ± 8.00	99.20 ± 9.43	87.01 ± 0.82	87.01 ± 0.82	87.01 ± 0.82	97.11 ± 6.08	97.61 ± 5.16	82.20 ± 7.92
Au7-300-drift-au7-cpd1-800	98.16 ± 0.61	92.66 ± 1.38	81.78 ± 4.66	86.32 ± 5.39	90.85 ± 2.08	91.77 ± 2.45	91.77 ± 2.45	88.86 ± 2.66	87.74 ± 3.17	95.23 ± 3.94
Au7-700	97.73 ± 1.32	91.56 ± 2.35	75.84 ± 5.04	96.84 ± 8.31	94.08 ± 4.32	95.37 ± 3.52	95.37 ± 3.52	90.41 ± 3.23	88.33 ± 3.98	82.94 ± 5.72
Au7-cpd1-500	98.68 ± 0.92	94.29 ± 2.51	70.29 ± 5.31	83.29 ± 7.52	82.49 ± 1.33	81.96 ± 2.13	81.96 ± 2.13	84.23 ± 5.60	82.95 ± 6.86	79.82 ± 6.44
Audiology-std	96.20 ± 0.35	31.94 ± 11.68	29.80 ± 8.89	38.14 ± 12.51	42.68 ± 9.22	37.28 ± 11.79	33.87 ± 9.68	25.65 ± 7.03	26.42 ± 5.98	60.90 ± 2.39
Audit-risk	58.08 ± 2.67	11.29 ± 4.10	0.27 ± 0.85	4.85 ± 2.54	3.24 ± 2.78	2.69 ± 2.83	2.42 ± 2.95	8.64 ± 4.01	8.72 ± 3.90	0.00 ± 0.00
Autism-adolescent-data	77.89 ± 5.47	9.38 ± 6.01	0.00 ± 0.00	21.64 ± 13.14	8.02 ± 10.37	9.94 ± 10.50	8.02 ± 10.37	21.09 ± 18.16	20.85 ± 18.51	0.00 ± 0.00
Autism-adult-data	74.39 ± 2.64	8.31 ± 4.77	0.00 ± 0.00	13.34 ± 9.74	1.81 ± 2.55	1.09 ± 2.45	1.45 ± 2.53	0.56 ± 0.31	0.44 ± 0.25	0.00 ± 0.00
Autism-child-data	79.33 ± 2.43	8.93 ± 3.35	0.00 ± 0.00	23.54 ± 7.65	0.69 ± 2.18	0.00 ± 0.00	0.00 ± 0.00	1.82 ± 1.73	1.95 ± 1.88	0.00 ± 0.00
Autos	75.47 ± 3.50	56.55 ± 14.32	26.24 ± 8.52	33.42 ± 12.55	84.46 ± 5.52	83.20 ± 6.66	83.20 ± 6.66	31.92 ± 7.36	31.38 ± 6.33	48.67 ± 12.86
Avila	51.66 ± 0.55	97.65 ± 2.37	8.75 ± 1.60	31.65 ± 3.48	41.49 ± 2.74	38.68 ± 2.40	36.83 ± 2.97	62.83 ± 2.84	62.56 ± 3.53	36.74 ± 3.26
Balance-scale	73.87 ± 2.73	56.05 ± 2.27	49.81 ± 6.48	33.52 ± 7.43	16.85 ± 2.67	12.64 ± 1.52	11.24 ± 2.34	21.20 ± 2.88	21.89 ± 3.60	76.65 ± 7.70
Balloons	81.25 ± 23.98	100.00 ± 0.00	91.06 ± 31.00	68.48 ± 22.88	57.00 ± 63.60	37.00 ± 62.55	37.00 ± 62.55	56.90 ± 46.90	54.78 ± 48.74	112.50 ± 77.28
Bank	95.82 ± 0.54	87.54 ± 4.70	70.70 ± 4.16	68.26 ± 6.54	51.27 ± 3.03	50.51 ± 2.78	49.86 ± 5.20	63.84 ± 5.63	65.32 ± 9.20	56.04 ± 3.01
Blood	96.32 ± 1.56	79.63 ± 5.50	83.64 ± 3.41	88.71 ± 11.09	59.27 ± 5.65	57.81 ± 4.24	59.28 ± 3.90	81.45 ± 4.21	81.29 ± 5.46	65.18 ± 4.59
Breast-cancer-wisc-diag	52.63 ± 4.65	14.91 ± 8.87	15.46 ± 8.53	9.03 ± 3.99	5.25 ± 4.74	4.51 ± 4.26	3.76 ± 2.50	8.15 ± 3.78	8.12 ± 4.00	23.28 ± 8.76
Breast-cancer-wisc-prog	97.66 ± 1.44	93.25 ± 26.65	79.33 ± 16.09	75.56 ± 24.57	56.86 ± 16.40	60.03 ± 25.23	61.33 ± 27.19	75.38 ± 25.65	73.70 ± 30.26	82.38 ± 22.18
Breast-cancer-wisc	30.02 ± 4.69	9.08 ± 3.74	16.30 ± 7.31	10.02 ± 6.58	6.33 ± 4.22	6.65 ± 4.34	7.28 ± 4.22	12.22 ± 7.57	10.45 ± 4.70	16.14 ± 10.05
Breast-cancer	93.44 ± 2.87	75.65 ± 9.99	87.28 ± 6.25	72.06 ± 18.00	62.49 ± 12.22	64.17 ± 12.75	66.71 ± 12.25	77.44 ± 13.67	78.80 ± 8.96	70.83 ± 11.74
Breast-tissue	67.58 ± 7.19	40.04 ± 13.68	41.08 ± 16.69	38.07 ± 15.80	50.18 ± 17.49	38.79 ± 19.93	36.37 ± 20.11	51.89 ± 8.28	51.91 ± 11.17	54.78 ± 11.74
Bupa	92.74 ± 3.01	94.88 ± 6.47	81.13 ± 16.90	78.64 ± 10.05	84.41 ± 4.54	83.81 ± 4.89	83.81 ± 4.89	76.65 ± 7.63	73.89 ± 7.61	93.87 ± 16.05
Caesarian	88.07 ± 11.68	84.08 ± 21.17	93.33 ± 11.98	96.10 ± 29.64	91.75 ± 12.23	76.84 ± 25.03	84.51 ± 35.13	91.04 ± 37.86	91.03 ± 36.15	105.03 ± 38.01
Car	94.52 ± 0.77	54.16 ± 2.40	6.32 ± 1.72	8.13 ± 2.30	5.69 ± 2.47	4.17 ± 2.52	3.66 ± 2.10	18.40 ± 2.78	17.59 ± 3.27	65.45 ± 0.26
Cardiotocography-10classes	81.53 ± 0.59	36.58 ± 2.75	23.93 ± 2.57	25.86 ± 3.51	22.35 ± 2.49	20.95 ± 3.05	20.11 ± 2.46	24.50 ± 3.11	25.72 ± 2.69	62.40 ± 2.72
Cardiotocography-3classes	87.86 ± 1.25	50.02 ± 8.43	23.36 ± 6.87	21.95 ± 4.73	24.26 ± 5.22	21.57 ± 5.64	21.19 ± 4.73	23.56 ± 2.74	22.59 ± 5.56	50.44 ± 8.62
Cervical-cancer	82.50 ± 7.36	23.68 ± 18.11	38.98 ± 28.40	30.08 ± 26.38	69.87 ± 5.37	62.97 ± 15.91	62.97 ± 15.91	18.32 ± 18.30	18.11 ± 18.48	52.95 ± 20.63
Chemicalcomposionofceramic	48.22 ± 4.83	0.00 ± 0.00	2.50 ± 7.91	2.46 ± 0.01	108.77 ± 4.62	108.77 ± 4.62	108.77 ± 4.62	1.03 ± 0.45	0.76 ± 0.36	0.00 ± 0.00
Chess-krvk	96.17 ± 0.23	90.50 ± 0.64	59.18 ± 2.87	56.30 ± 3.59	60.36 ± 2.90	57.89 ± 2.50	56.18 ± 3.27	74.77 ± 1.16	75.03 ± 1.79	83.17 ± 2.35
Chess-krvkp	94.95 ± 0.25	42.83 ± 3.05	2.14 ± 0.93	20.43 ± 2.78	2.63 ± 1.81	1.63 ± 1.26	1.63 ± 1.07	1.99 ± 1.11	1.82 ± 1.11	67.22 ± 2.90
Congressional-voting	98.94 ± 0.62	93.83 ± 8.29	96.58 ± 5.20	94.06 ± 9.83	80.94 ± 8.39	81.91 ± 11.00	82.87 ± 12.24	91.50 ± 5.95	91.17 ± 6.95	78.06 ± 5.53
Conn-bench-sonar-mines-rocks	90.85 ± 2.57	62.64 ± 17.90	57.49 ± 14.47	27.74 ± 13.91	31.80 ± 14.15	26.05 ± 15.63	23.14 ± 15.75	39.52 ± 18.34	36.71 ± 17.20	73.43 ± 21.12
Conn-bench-vowel-deterding	69.18 ± 0.74	47.05 ± 4.24	24.61 ± 6.91	3.07 ± 1.42	6.46 ± 2.08	2.71 ± 1.97	1.66 ± 1.64	22.53 ± 3.06	23.12 ± 3.66	71.04 ± 5.43
Connect-4	99.54 ± 0.11	100.96 ± 2.40	64.33 ± 4.35	75.45 ± 7.14	67.46 ± 0.66	63.17 ± 2.51	60.13 ± 4.59	70.08 ± 5.04	67.21 ± 4.00	67.23 ± 0.35
Connectionist	90.55 ± 2.58	64.31 ± 21.22	53.66 ± 14.58	26.82 ± 11.63	77.57 ± 17.04	60.81 ± 23.09	54.92 ± 24.46	34.74 ± 15.76	33.84 ± 15.80	74.66 ± 13.93
Contrac	96.44 ± 0.59	85.85 ± 3.75	79.77 ± 4.22	88.33 ± 5.68	70.39 ± 3.42	70.59 ± 5.45	71.54 ± 5.97	80.87 ± 3.06	80.04 ± 2.48	80.47 ± 3.12
Covid-19	82.03 ± 14.93	74.56 ± 43.25	59.63 ± 65.09	79.44 ± 49.06	62.70 ± 59.12	73.88 ± 73.34	59.34 ± 71.97	58.59 ± 61.89	56.92 ± 63.13	30.51 ± 52.42
Credit-approval	83.51 ± 2.47	45.92 ± 6.36	38.63 ± 6.36	36.30 ± 11.38	29.63 ± 11.00	30.21 ± 9.77	31.10 ± 9.29	31.95 ± 5.23	34.02 ± 7.16	29.34 ± 9.07
Crowdsource	73.96 ± 0.93	39.44 ± 4.39	26.14 ± 3.03	10.15 ± 1.97	50.77 ± 2.17	50.77 ± 2.17	50.77 ± 2.17	17.46 ± 3.77	17.68 ± 4.13	44.23 ± 4.55
Crx	82.23 ± 2.36	45.10 ± 5.62	38.94 ± 6.45	38.35 ± 9.75	89.49 ± 7.22	88.02 ± 8.61	85.38 ± 10.37	36.33 ± 8.22	33.45 ± 4.95	29.34 ± 9.07
Cryother	63.26 ± 9.90	36.78 ± 21.94	21.18 ± 14.92	22.05 ± 16.12	35.58 ± 25.91	33.34 ± 26.00	33.34 ± 26.00	27.40 ± 16.86	33.55 ± 18.89	37.93 ± 21.23
Cylinder-bands	93.48 ± 1.64	68.93 ± 11.88	60.97 ± 12.45	64.94 ± 15.13	50.87 ± 8.08	43.91 ± 9.21	39.39 ± 6.58	57.23 ± 10.13	55.07 ± 9.63	70.12 ± 9.03
Dbworld-bodies	97.83 ± 0.70	49.39 ± 31.18	42.52 ± 32.62	82.56 ± 20.22	91.52 ± 10.51	91.52 ± 10.51	72.85 ± 25.44	/	/	27.35 ± 26.75
Dbworld-bodies-stemmed	97.66 ± 0.79	47.11 ± 26.12	30.81 ± 26.46	73.31 ± 28.56	91.52 ± 10.51	82.41 ± 18.05	52.77 ± 26.76	/	/	51.81 ± 41.11
Dbworld-subjects	98.00 ± 0.72	34.71 ± 20.89	61.24 ± 20.24	50.30 ± 24.75	91.52 ± 10.51	91.52 ± 10.51	91.52 ± 10.51	26.89 ± 24.71	26.72 ± 27.31	75.22 ± 24.75
Dbworld-subjects-stemmed	98.01 ± 0.91	34.39 ± 22.86	54.43 ± 27.54	38.63 ± 18.38	91.52 ± 10.51	91.52 ± 10.51	91.52 ± 10.51	28.03 ± 23.32	28.47 ± 23.09	78.63 ± 20.43
Dermatology	72.96 ± 0.82	3.58 ± 3.29	6.40 ± 3.79	7.57 ± 5.23	3.06 ± 2.96	2.71 ± 3.10	2.71 ± 3.49	4.35 ± 1.62	4.10 ± 1.71	62.88 ± 2.99
Diabetes	86.31 ± 3.39	62.50 ± 7.63	69.45 ± 9.95	65.77 ± 10.36	76.77 ± 0.63	76.77 ± 0.63	76.77 ± 0.63	64.99 ± 5.29	63.83 ± 4.80	62.74 ± 11.34
Diabetic	92.32 ± 1.49	86.85 ± 4.44	76.21 ± 7.00	77.67 ± 9.51	83.89 ± 9.59	81.45 ± 12.08	81.27 ± 11.48	66.22 ± 6.30	65.45 ± 5.31	93.84 ± 11.41
Divorce	19.50 ± 5.15	4.71 ± 6.08	11.74 ± 11.75	5.11 ± 5.53	4.71 ± 6.08	4.71 ± 6.08	4.71 ± 6.08	4.07 ± 5.11	4.05 ± 5.17	9.41 ± 10.80
Dota2train	99.84 ± 0.08	94.52 ± 3.62	94.55 ± 8.29	89.94 ± 6.06	94.88 ± 0.69	92.95 ± 7.23	95.10 ± 6.60	99.93 ± 6.20	96.66 ± 4.21	89.49 ± 7.29
Dow-jones-index	89.78 ± 3.12	99.48 ± 6.00	57.59 ± 7.94	90.96 ± 12.70	96.02 ± 0.43	96.02 ± 0.43	96.02 ± 0.43	91.60 ± 6.27	89.30 ± 5.10	87.20 ± 6.17
Dry-bean-dataset	26.66 ± 0.80	12.10 ± 1.59	9.98 ± 1.33	6.74 ± 1.36	46.84 ± 3.31	46.84 ± 3.31	46.84 ± 3.31	11.68 ± 1.87	11.20 ± 1.54	38.29 ± 2.33
Early-stage-diabetes-data-upload	78.44 ± 2.75	31.47 ± 7.34	11.59 ± 5.57	4.37 ± 4.61	12.18 ± 8.77	10.97 ± 8.57	9.75 ± 7.94	8.41 ± 4.89	9.15 ± 6.25	37.37 ± 9.14
Echocardiogram	89.70 ± 4.98	50.08 ± 25.33	54.44 ± 22.21	59.82 ± 30.06	41.36 ± 18.41	43.30 ± 25.02	43.30 ± 25.02	54.38 ± 28.56	51.03 ± 28.13	32.62 ± 14.70
Ecoli	75.32 ± 1.74	23.54 ± 5.79	27.01 ± 9.08	29.22 ± 8.50	18.34 ± 6.25	18.34 ± 5.94	17.52 ± 4.81	26.48 ± 3.88	26.48 ± 4.78	45.16 ± 9.41
Eegeyesate	91.56 ± 0.57	107.84 ± 4.22	34.17 ± 1.96	33.05 ± 4.11	90.71 ± 0.06	90.63 ± 0.13	90.63 ± 0.13	98.31 ± 1.76	97.85 ± 2.01	75.59 ± 2.38
Electrical	68.31 ± 5.99	13.75 ± 5.86	0.00 ± 0.00	19.92 ± 16.10	38.93 ± 16.77	38.96 ± 14.64	36.80 ± 13.94	4.36 ± 3.42	3.90 ± 3.09	0.73 ± 2.31
Energy-y1	59.86 ± 1.20	30.85 ± 3.38	5.99 ± 2.46	39.08 ± 6.40	18.15 ± 2.23	16.28 ± 2.57	16.28 ± 4.49	20.68 ± 1.21	21.30 ± 2.18	24.83 ± 3.02
Energy-y2	55.82 ± 1.10	26.84 ± 3.05	20.89 ± 3.06	38.15 ± 5.31	16.43 ± 3.27	14.77 ± 2.47	13.94 ± 2.39	19.92 ± 2.78	18.35 ± 3.67	18.52 ± 4.92
Extentionofz-alizadehsani	93.03 ± 1.21	19.68 ± 9.03	1.59 ± 2.20	23.91 ± 12.40	69.98 ± 2.67	69.98 ± 2.67	69.98 ± 2.67	5.18 ± 5.21	4.99 ± 5.25	32.95 ± 11.46
Fertility	96.97 ± 4.64	92.36 ± 13.73	103.29 ± 16.87	80.50 ± 51.66	53.87 ± 10.40	48.98 ± 20.03	48.98 ± 30.55	57.42 ± 28.54	60.18 ± 42.97	53.87 ± 10.40
First-order	97.19 ± 1.01	393.57 ± 21.98	0.00 ± 0.00	3.81 ± 2.79	55.11 ± 1.16	26.10 ± 8.60	11.74 ± 6.33	1.72 ± 1.86	1.44 ± 1.83	53.29 ± 10.21
Flags	92.10 ± 1.78	66.51 ± 7.33	55.39 ± 10.35	70.24 ± 10.52	59.01 ± 11.69	56.97 ± 12.75	58.21 ± 16.34	69.74 ± 13.52	65.32 ± 12.69	53.20 ± 8.94
Foresttypes	61.01 ± 2.62	6.73 ± 5.55	8.70 ± 7.50	8.20 ± 4.86	93.73 ± 3.22	91.00 ± 4.76	91.00 ± 4.76	6.85 ± 4.62	6.50 ± 4.84	23.86 ± 11.45
Garments-worker-productivity	94.96 ± 0.57	97.33 ± 2.17	73.76 ± 5.31	97.73 ± 4.55	85.27 ± 3.86	84.50 ± 3.89	83.51 ± 3.30	86.04 ± 3.49	87.19 ± 3.55	89.36 ± 4.91
Gender-name-dataset	99.13 ± 0.82	86.75 ± 9.13	100.02 ± 0.10	86.20 ± 3.04	74.81 ± 2.89	74.62 ± 4.21	74.49 ± 3.78	100.20 ± 2.29	99.95 ± 2.89	82.34 ± 2.17
Gesture-a1-raw	43.80 ± 1.19	41.31 ± 3.68	13.74 ± 2.79	5.76 ± 1.65	88.48 ± 0.37	88.48 ± 0.37	88.48 ± 0.37	16.33 ± 2.58	15.27 ± 1.27	4.81 ± 1.98
Gesture-a1-va3	76.61 ± 2.00	60.43 ± 4.81	52.58 ± 6.74	38.36 ± 4.03	88.64 ± 0.21	88.64 ± 0.21	88.64 ± 0.21	51.35 ± 3.53	50.57 ± 3.20	56.61 ± 2.98
Gesture-a2-raw	44.81 ± 0.99	45.32 ± 3.76	16.78 ± 3.88	8.14 ± 4.32	87.07 ± 0.36	87.07 ± 0.36	87.07 ± 0.36	18.17 ± 2.42	16.61 ± 2.97	7.56 ± 3.75
Gesture-a2-va3	81.75 ± 2.12	82.33 ± 10.29	63.73 ± 3.73	46.37 ± 5.87	87.27 ± 0.34	87.27 ± 0.34	87.27 ± 0.34	64.21 ± 4.71	63.64 ± 5.66	65.65 ± 5.84
Gesture-a3-raw	34.69 ± 1.28	53.80 ± 3.19	10.48 ± 1.45	5.31 ± 1.33	86.11 ± 0.26	86.11 ± 0.26	86.11 ± 0.26	17.87 ± 1.71	18.09 ± 2.40	6.32 ± 1.56
Gesture-a3-va3	81.14 ± 0.83	67.45 ± 3.72	34.68 ± 4.42	13.90 ± 2.38	86.25 ± 0.28	86.25 ± 0.28	86.25 ± 0.28	57.22 ± 3.36	55.36 ± 3.35	66.08 ± 3.20
Gesture-b1-raw	41.41 ± 2.23	72.03 ± 4.67	11.04 ± 3.44	7.69 ± 3.91	83.92 ± 0.90	83.66 ± 1.05	83.66 ± 1.05	21.63 ± 3.76	17.96 ± 2.56	11.74 ± 3.07
Gesture-b1-va3	83.39 ± 2.35	90.45 ± 8.66	43.56 ± 5.25	13.47 ± 3.67	84.39 ± 0.47	84.39 ± 0.47	84.39 ± 0.47	60.78 ± 3.92	56.72 ± 5.54	78.87 ± 5.95
Gesture-b3-raw	48.32 ± 2.62	60.30 ± 3.59	12.84 ± 4.51	6.27 ± 1.15	87.61 ± 0.43	87.61 ± 0.43	87.61 ± 0.43	18.99 ± 4.84	18.83 ± 4.55	6.07 ± 3.08
Gesture-b3-va3	90.20 ± 1.63	75.18 ± 4.51	57.61 ± 4.64	29.08 ± 3.82	87.73 ± 0.25	87.73 ± 0.25	87.73 ± 0.25	60.88 ± 4.90	62.11 ± 4.47	78.51 ± 7.19
Gesture-c1-raw	47.07 ± 2.15	47.52 ± 3.53	14.73 ± 2.89	7.66 ± 2.13	94.19 ± 0.53	94.19 ± 0.53	94.19 ± 0.53	19.08 ± 3.35	19.07 ± 4.29	7.88 ± 3.30
Gesture-c1-va3	83.85 ± 2.07	62.77 ± 3.70	54.69 ± 4.34	37.89 ± 4.35	94.50 ± 0.42	94.50 ± 0.42	94.50 ± 0.42	50.38 ± 4.02	51.14 ± 3.08	74.23 ± 5.90
Gesture-c3-raw	44.71 ± 1.72	51.60 ± 5.30	16.74 ± 4.75	9.32 ± 2.97	93.71 ± 0.28	93.71 ± 0.28	93.71 ± 0.28	21.17 ± 2.88	23.33 ± 2.29	7.27 ± 1.72
Gesture-c3-va3	86.27 ± 1.65	69.93 ± 2.91	59.86 ± 5.09	48.48 ± 6.04	93.77 ± 0.25	93.77 ± 0.25	93.77 ± 0.25	62.21 ± 4.70	62.77 ± 5.26	73.69 ± 3.58
Glass	74.97 ± 3.67	71.06 ± 9.08	48.23 ± 10.02	42.06 ± 11.80	36.66 ± 8.94	37.99 ± 11.64	39.26 ± 11.50	53.34 ± 9.99	50.21 ± 5.68	58.02 ± 12.16
Go-track-tracks	67.60 ± 8.74	37.42 ± 9.95	27.13 ± 13.92	19.34 ± 16.96	56.18 ± 25.50	56.25 ± 23.12	56.25 ± 23.12	37.59 ± 11.74	36.30 ± 10.90	49.11 ± 27.29
Haberman-survival	94.64 ± 3.43	85.03 ± 6.71	93.09 ± 6.02	86.06 ± 18.01	70.33 ± 11.67	73.69 ± 7.19	73.69 ± 7.19	90.85 ± 6.56	90.66 ± 7.17	69.48 ± 7.26
Hayes-roth	62.76 ± 4.25	67.68 ± 4.69	30.93 ± 7.92	21.33 ± 8.68	21.04 ± 10.92	29.15 ± 10.03	30.33 ± 10.01	51.32 ± 15.37	49.42 ± 15.86	86.18 ± 9.07
Hcc-data	94.91 ± 2.00	70.55 ± 13.41	90.97 ± 11.91	77.33 ± 16.70	80.73 ± 3.96	80.73 ± 3.96	80.73 ± 3.96	75.16 ± 23.89	76.59 ± 20.02	65.20 ± 23.22
Hcvdat	80.27 ± 2.16	38.07 ± 12.46	33.72 ± 8.25	40.46 ± 5.94	53.59 ± 1.50	53.59 ± 1.50	53.59 ± 1.50	32.43 ± 10.46	34.86 ± 10.24	45.68 ± 6.81
Heart-cleveland	91.33 ± 2.49	71.33 ± 10.43	80.13 ± 9.05	70.80 ± 9.79	65.53 ± 12.65	63.49 ± 13.59	65.48 ± 14.24	72.20 ± 8.53	71.26 ± 7.75	73.19 ± 10.59
Heart-hungarian	78.79 ± 4.96	41.67 ± 13.98	59.37 ± 11.70	49.09 ± 17.48	39.56 ± 16.42	38.16 ± 13.73	36.72 ± 13.99	50.25 ± 16.24	50.26 ± 14.06	45.51 ± 20.32
Heart-switzerland	97.90 ± 3.28	92.11 ± 8.88	90.12 ± 15.16	94.39 ± 18.58	84.83 ± 8.69	83.77 ± 7.91	81.35 ± 9.02	84.47 ± 15.43	80.93 ± 13.60	94.32 ± 17.44
Heart-va	97.78 ± 1.25	91.24 ± 7.37	91.84 ± 6.28	85.06 ± 9.93	85.18 ± 12.46	88.43 ± 11.16	91.04 ± 12.31	93.90 ± 7.85	94.42 ± 10.22	94.29 ± 12.81
Heart-failure-clinical-records-dataset	93.16 ± 1.63	57.66 ± 10.11	54.22 ± 10.92	79.25 ± 15.41	73.52 ± 2.86	73.52 ± 2.86	73.52 ± 2.86	62.49 ± 10.67	54.54 ± 18.04	32.91 ± 14.29
Hepatitis	92.08 ± 3.55	54.48 ± 24.83	73.05 ± 20.64	54.33 ± 16.47	50.89 ± 18.80	47.08 ± 28.08	47.08 ± 28.08	61.88 ± 27.40	65.42 ± 34.33	78.56 ± 21.85
Hill-valley	100.16 ± 2.29	102.38 ± 4.86	100.08 ± 0.25	96.41 ± 6.94	95.70 ± 13.15	95.71 ± 14.88	93.73 ± 13.99	91.27 ± 4.53	90.95 ± 4.77	105.01 ± 9.86
Hiv1625data	49.77 ± 3.56	20.84 ± 5.20	36.62 ± 6.44	32.25 ± 3.53	22.02 ± 5.66	18.55 ± 6.01	17.69 ± 6.59	14.33 ± 5.04	18.96 ± 6.49	58.22 ± 4.93
Hiv746data	49.45 ± 3.09	21.20 ± 5.60	51.28 ± 6.19	33.32 ± 6.09	21.02 ± 7.46	18.32 ± 5.62	18.05 ± 6.56	15.19 ± 4.47	16.16 ± 6.28	38.28 ± 8.74
Horse-colic	87.65 ± 2.40	59.11 ± 12.16	33.81 ± 9.25	50.78 ± 16.06	35.25 ± 10.36	33.09 ± 11.30	32.37 ± 12.29	49.23 ± 10.99	47.76 ± 14.44	38.15 ± 11.72
Htru	37.39 ± 2.04	33.26 ± 2.70	20.41 ± 2.69	17.19 ± 2.29	53.21 ± 0.84	51.20 ± 1.14	51.77 ± 1.09	18.13 ± 2.96	17.91 ± 2.34	14.44 ± 2.41
Hypothyroid	93.55 ± 0.93	48.91 ± 5.21	4.16 ± 1.75	58.72 ± 10.61	50.73 ± 2.01	49.82 ± 4.91	50.00 ± 5.21	52.30 ± 32.65	56.91 ± 36.33	25.81 ± 6.37
Ibeacon-rssi-labeled	97.01 ± 0.23	83.22 ± 1.70	75.07 ± 2.27	64.94 ± 2.84	66.64 ± 1.72	64.22 ± 2.84	63.01 ± 3.17	84.54 ± 1.89	82.78 ± 2.15	96.65 ± 1.75
Ilpd-indian-liver	94.61 ± 3.03	108.16 ± 11.80	94.49 ± 8.25	86.13 ± 14.60	70.00 ± 1.38	71.70 ± 4.24	71.29 ± 6.12	84.04 ± 6.74	83.75 ± 7.20	83.01 ± 7.53
Image-segmentation	59.74 ± 3.02	26.23 ± 6.59	15.54 ± 9.18	18.04 ± 5.08	15.00 ± 3.75	15.00 ± 5.27	15.56 ± 5.11	15.24 ± 4.33	14.83 ± 4.43	47.78 ± 10.21
Immunotherapy	89.60 ± 4.21	92.94 ± 19.96	69.14 ± 23.77	91.35 ± 50.14	62.20 ± 8.35	62.20 ± 8.35	62.20 ± 8.35	57.96 ± 25.66	61.26 ± 26.96	45.99 ± 27.09
Impensdata	77.82 ± 2.69	47.91 ± 6.07	99.79 ± 0.00	58.34 ± 8.29	59.21 ± 0.74	59.21 ± 0.74	59.21 ± 0.74	38.09 ± 7.74	43.16 ± 10.61	58.80 ± 7.71
In-vehicle-coupon-recommendation	97.77 ± 0.12	83.15 ± 1.27	76.82 ± 1.34	73.01 ± 2.69	63.41 ± 2.68	62.33 ± 2.67	61.48 ± 2.69	60.94 ± 1.98	59.27 ± 2.63	80.46 ± 1.80
Indian	94.02 ± 2.54	107.75 ± 12.26	80.09 ± 10.71	87.84 ± 16.73	67.51 ± 3.81	69.19 ± 3.32	69.19 ± 3.32	84.54 ± 7.55	83.72 ± 7.19	83.42 ± 7.37
Ionosphere	51.47 ± 4.53	37.70 ± 10.11	20.34 ± 7.16	30.12 ± 9.79	12.34 ± 4.95	11.09 ± 6.88	9.24 ± 6.53	21.13 ± 3.91	20.94 ± 5.71	41.49 ± 16.32
Iris	37.75 ± 5.36	10.00 ± 5.76	7.87 ± 7.85	8.98 ± 8.04	5.00 ± 5.27	6.00 ± 6.99	5.00 ± 5.27	7.36 ± 6.20	6.64 ± 5.32	12.00 ± 9.19
Jain	21.84 ± 8.21	17.35 ± 7.28	1.39 ± 4.38	0.77 ± 0.01	0.00 ± 0.00	0.00 ± 0.00	0.00 ± 0.00	17.70 ± 6.28	17.64 ± 6.43	12.52 ± 8.66
Jsbach-chorals-harmony	80.38 ± 0.77	22.07 ± 2.53	26.32 ± 2.96	13.94 ± 2.18	18.19 ± 2.95	15.02 ± 2.18	13.64 ± 3.32	12.76 ± 2.82	14.35 ± 4.55	19.65 ± 3.00
Knowledge	76.90 ± 4.13	35.94 ± 11.06	11.39 ± 7.67	36.84 ± 11.45	22.75 ± 13.76	15.49 ± 12.38	11.42 ± 11.02	14.79 ± 6.92	14.60 ± 7.07	24.50 ± 13.45
Lasvegastripadvisorreviews	99.97 ± 0.25	98.75 ± 2.60	97.89 ± 4.52	98.63 ± 4.57	101.19 ± 3.93	103.28 ± 5.62	100.49 ± 5.64	99.59 ± 5.39	98.21 ± 4.86	100.23 ± 3.81
Leaf	82.76 ± 1.37	28.96 ± 7.37	42.06 ± 7.51	100.12 ± 3.03	85.83 ± 4.24	78.52 ± 5.33	77.00 ± 5.75	56.53 ± 5.59	57.23 ± 5.43	78.83 ± 5.06
Led-display	86.13 ± 0.43	42.75 ± 2.23	46.22 ± 2.64	42.55 ± 2.56	31.24 ± 4.55	31.46 ± 4.23	31.80 ± 4.07	42.19 ± 2.22	40.90 ± 2.41	89.39 ± 1.40
Lenses	78.24 ± 19.64	102.13 ± 12.97	43.55 ± 64.20	37.48 ± 34.78	36.36 ± 49.65	32.73 ± 47.28	32.73 ± 47.28	53.43 ± 45.66	55.71 ± 48.24	62.42 ± 68.75
Letter	89.09 ± 0.12	43.64 ± 0.59	14.28 ± 0.71	4.39 ± 0.31	5.33 ± 0.26	4.16 ± 0.31	3.63 ± 0.22	21.06 ± 1.04	20.96 ± 0.80	86.06 ± 0.38
Libras	79.37 ± 2.43	39.44 ± 9.81	33.97 ± 10.24	18.90 ± 5.10	20.23 ± 4.39	15.77 ± 4.66	13.98 ± 4.22	25.08 ± 4.49	23.87 ± 4.32	84.50 ± 6.46
Low-res-spect	74.95 ± 1.64	29.64 ± 5.91	27.01 ± 5.08	26.51 ± 5.94	15.95 ± 3.69	15.66 ± 4.15	14.24 ± 3.85	14.10 ± 4.78	14.10 ± 4.52	39.53 ± 7.99
Lung-cancer	92.89 ± 5.02	56.37 ± 24.31	87.48 ± 36.43	76.93 ± 50.20	70.31 ± 32.97	61.64 ± 32.47	73.12 ± 38.09	79.89 ± 33.05	79.31 ± 34.22	79.46 ± 27.00
Lymphography	84.63 ± 2.26	38.02 ± 11.23	52.62 ± 13.94	48.40 ± 24.02	26.31 ± 17.07	27.84 ± 18.24	31.39 ± 16.28	37.80 ± 14.21	37.20 ± 13.19	45.35 ± 14.88
Magic	74.32 ± 1.89	60.02 ± 4.33	48.72 ± 3.98	44.28 ± 5.09	33.12 ± 4.66	32.55 ± 3.78	32.55 ± 3.08	41.43 ± 4.15	42.65 ± 4.22	61.10 ± 9.66
Mammographic	72.02 ± 2.56	48.19 ± 5.74	49.33 ± 3.99	51.58 ± 9.61	34.73 ± 4.29	35.56 ± 5.22	35.15 ± 5.31	50.99 ± 3.11	52.18 ± 5.22	36.41 ± 2.52
Miniboone	77.60 ± 0.71	180.29 ± 3.52	36.81 ± 6.06	42.03 ± 4.01	39.34 ± 6.27	36.60 ± 5.36	34.65 ± 4.70	53.19 ± 7.06	54.03 ± 8.21	46.39 ± 4.51
Molec-biol-promoter	89.02 ± 4.01	26.91 ± 16.74	53.25 ± 20.10	55.99 ± 23.67	34.72 ± 26.91	30.72 ± 23.89	30.72 ± 23.89	46.07 ± 17.97	46.21 ± 18.16	60.51 ± 22.18
Molec-biol-splice	96.55 ± 0.23	16.95 ± 2.43	13.87 ± 2.87	60.03 ± 3.40	20.95 ± 3.21	20.69 ± 2.78	21.56 ± 2.12	26.61 ± 3.45	27.08 ± 2.93	59.63 ± 3.71
Monks-1	78.06 ± 6.44	73.39 ± 15.31	8.32 ± 13.87	61.26 ± 18.44	38.71 ± 24.21	33.84 ± 21.84	33.71 ± 19.18	27.39 ± 27.93	23.27 ± 32.81	53.45 ± 19.63
Monks-2	100.45 ± 2.64	101.79 ± 5.62	57.45 ± 20.60	82.85 ± 22.09	89.06 ± 15.27	82.81 ± 26.21	75.20 ± 25.61	61.57 ± 19.79	57.60 ± 17.00	87.86 ± 9.58
Monks-3	74.97 ± 6.43	53.25 ± 8.94	21.44 ± 15.42	50.96 ± 30.89	19.62 ± 15.12	21.41 ± 15.91	21.41 ± 15.91	33.73 ± 20.12	25.08 ± 12.86	44.24 ± 7.68
Mushroom	65.43 ± 0.94	24.96 ± 2.21	0.00 ± 0.00	0.03 ± 0.00	0.00 ± 0.00	0.00 ± 0.00	0.00 ± 0.00	0.14 ± 0.01	0.09 ± 0.01	2.96 ± 0.96
Musk-1	80.80 ± 2.69	52.32 ± 14.54	31.55 ± 13.33	31.05 ± 9.22	18.77 ± 8.74	15.39 ± 5.82	12.83 ± 6.08	14.69 ± 4.89	14.71 ± 4.77	76.50 ± 18.17
Musk-2	76.64 ± 1.26	55.96 ± 5.83	18.49 ± 4.90	14.48 ± 3.61	14.72 ± 4.51	9.70 ± 3.58	7.30 ± 2.85	5.64 ± 2.64	5.43 ± 3.23	38.67 ± 4.73
Newdiagnosis	48.01 ± 3.55	20.34 ± 6.58	0.00 ± 0.00	0.17 ± 0.02	0.00 ± 0.00	0.00 ± 0.00	0.00 ± 0.00	1.41 ± 0.18	1.02 ± 0.10	46.26 ± 14.11
Nursery	88.77 ± 0.15	31.12 ± 0.76	0.89 ± 0.39	17.22 ± 0.86	2.58 ± 0.32	1.94 ± 0.30	1.56 ± 0.29	7.45 ± 1.96	9.42 ± 2.55	42.53 ± 1.51
Obesitydataset-raw-and-data-sinthetic	78.81 ± 0.39	44.47 ± 1.69	8.71 ± 2.07	21.26 ± 2.88	12.01 ± 2.72	9.68 ± 2.62	8.80 ± 2.59	8.47 ± 3.07	8.33 ± 1.77	38.45 ± 4.18
Obs-network-dataset-2-aug27	45.55 ± 1.79	41.61 ± 7.45	0.52 ± 1.64	1.51 ± 0.92	3.45 ± 0.97	3.45 ± 0.97	3.45 ± 0.97	7.92 ± 2.39	5.39 ± 1.83	13.83 ± 3.08
Occupancy-data	18.67 ± 2.03	10.09 ± 2.20	3.99 ± 1.68	1.95 ± 1.08	41.13 ± 4.35	38.62 ± 4.99	38.62 ± 4.99	7.57 ± 1.99	7.12 ± 2.18	2.83 ± 1.67
Occupancy-data2	22.14 ± 1.37	11.92 ± 1.51	2.71 ± 0.55	1.64 ± 0.68	49.55 ± 1.50	43.21 ± 1.76	43.21 ± 1.76	2.78 ± 0.62	2.62 ± 0.53	1.88 ± 0.47
Occupancy-data3	14.30 ± 1.40	6.64 ± 1.54	2.00 ± 0.45	1.66 ± 0.67	51.10 ± 1.59	46.29 ± 1.83	46.25 ± 1.81	4.03 ± 0.84	3.97 ± 0.88	1.87 ± 0.53
Old	84.69 ± 0.53	15.52 ± 2.13	13.01 ± 2.86	5.28 ± 1.68	9.97 ± 1.88	9.73 ± 2.58	9.15 ± 2.41	6.60 ± 2.04	6.64 ± 2.48	22.91 ± 3.27
Online-shoppers-intention	92.63 ± 0.42	86.25 ± 4.57	53.53 ± 1.74	70.60 ± 2.19	59.24 ± 0.21	59.42 ± 0.49	59.64 ± 0.59	51.02 ± 5.28	52.07 ± 6.12	44.98 ± 3.77
Oocytes-merluccius-nucleus-4d	89.08 ± 2.38	90.22 ± 8.87	65.21 ± 8.13	62.94 ± 8.77	51.35 ± 6.60	44.93 ± 5.64	42.93 ± 6.77	45.77 ± 5.96	47.91 ± 7.66	75.00 ± 11.58
Oocytes-merluccius-states-2f	53.89 ± 4.04	32.92 ± 8.01	23.11 ± 7.17	19.73 ± 6.14	18.04 ± 5.61	17.19 ± 4.55	15.71 ± 4.28	19.18 ± 3.56	19.41 ± 4.66	39.27 ± 10.17
Oocytes-trisopterus-nucleus-2f	89.86 ± 2.47	94.11 ± 10.06	61.67 ± 11.17	52.72 ± 11.86	38.64 ± 7.51	33.25 ± 6.97	34.15 ± 5.66	37.00 ± 5.56	39.20 ± 6.51	83.14 ± 12.22
Oocytes-trisopterus-states-5b	61.02 ± 4.44	48.06 ± 8.10	26.01 ± 6.06	18.56 ± 7.37	16.39 ± 6.78	15.30 ± 5.46	14.42 ± 4.63	14.42 ± 5.03	14.44 ± 5.16	39.78 ± 5.50
Optdigits	89.32 ± 0.15	9.66 ± 1.12	11.34 ± 1.61	1.74 ± 0.71	29.85 ± 2.12	27.92 ± 2.31	27.92 ± 2.31	2.91 ± 0.67	2.43 ± 0.57	81.08 ± 1.37
Optical	88.77 ± 0.22	9.36 ± 1.35	12.64 ± 1.31	2.00 ± 0.63	1.57 ± 0.62	1.39 ± 0.67	1.42 ± 0.65	2.91 ± 0.68	2.93 ± 0.75	80.33 ± 1.44
Ozone	98.65 ± 0.75	514.31 ± 55.69	83.66 ± 10.30	87.49 ± 11.32	51.08 ± 1.86	51.08 ± 1.86	51.08 ± 1.86	67.47 ± 15.39	61.98 ± 13.01	55.98 ± 7.57
Page-blocks	67.45 ± 1.99	57.06 ± 7.90	21.18 ± 2.32	21.68 ± 3.22	20.05 ± 3.93	18.71 ± 3.95	18.52 ± 4.00	26.28 ± 3.56	25.37 ± 3.44	32.81 ± 3.16
Parkingbirmingham	21.04 ± 1.66	31.59 ± 1.11	0.00 ± 0.00	3.44 ± 0.69	42.84 ± 3.92	40.14 ± 4.05	40.14 ± 4.05	27.47 ± 1.45	23.68 ± 1.49	0.00 ± 0.00
Parkinsons	74.34 ± 7.36	82.45 ± 30.00	53.50 ± 10.07	11.04 ± 6.57	32.95 ± 16.92	27.47 ± 16.79	23.27 ± 15.50	31.68 ± 15.10	29.52 ± 19.55	37.10 ± 20.47
Pasture	81.94 ± 8.65	40.75 ± 34.25	35.21 ± 28.56	47.07 ± 37.59	107.02 ± 6.04	107.02 ± 6.04	107.02 ± 6.04	41.92 ± 35.99	43.88 ± 36.49	54.68 ± 39.33
Pbc	83.92 ± 3.21	50.57 ± 13.52	60.78 ± 11.94	85.44 ± 18.48	81.30 ± 1.38	81.30 ± 1.38	81.30 ± 1.38	61.73 ± 10.61	56.72 ± 6.29	58.61 ± 19.36
Pen	68.81 ± 0.36	16.90 ± 1.26	4.44 ± 0.53	0.81 ± 0.19	96.14 ± 0.95	95.06 ± 1.06	95.06 ± 1.06	6.74 ± 0.77	6.83 ± 0.43	67.85 ± 1.71
Pendigits	68.16 ± 0.40	14.41 ± 1.41	4.97 ± 0.79	0.78 ± 0.26	0.43 ± 0.21	0.37 ± 0.17	0.39 ± 0.19	6.10 ± 0.69	6.03 ± 0.60	66.97 ± 2.20
Pharynx	100.44 ± 3.94	107.48 ± 11.56	99.56 ± 0.04	100.09 ± 29.37	67.52 ± 22.16	73.58 ± 28.05	81.86 ± 28.07	80.91 ± 10.33	82.63 ± 43.90	201.57 ± 18.02
Phishingwebsites	87.61 ± 0.21	18.12 ± 1.15	11.49 ± 0.83	6.55 ± 1.11	11.14 ± 1.05	10.14 ± 1.02	9.60 ± 0.93	6.99 ± 0.83	6.88 ± 0.63	22.51 ± 1.14
Pima	86.45 ± 3.14	62.48 ± 7.65	69.45 ± 9.95	65.77 ± 10.36	53.56 ± 8.94	54.71 ± 8.81	55.00 ± 7.52	65.00 ± 5.29	63.83 ± 4.80	62.74 ± 11.34
Pittsburg-bridges-rel-l	85.63 ± 4.14	66.08 ± 15.18	76.14 ± 17.01	49.78 ± 27.10	54.48 ± 20.28	52.97 ± 20.36	45.14 ± 18.33	67.73 ± 16.66	64.05 ± 17.55	48.20 ± 17.58
Pittsburg-bridges-span	86.15 ± 3.47	64.47 ± 11.41	72.83 ± 17.00	72.74 ± 26.22	56.74 ± 24.06	58.65 ± 26.75	58.65 ± 26.75	58.68 ± 11.30	54.18 ± 17.94	73.56 ± 22.28
Pittsburg-bridges-t-or-d	92.43 ± 8.61	80.50 ± 21.12	86.75 ± 31.08	69.10 ± 43.95	53.74 ± 26.20	47.70 ± 33.01	43.14 ± 29.26	58.96 ± 44.70	65.23 ± 51.65	60.71 ± 42.49
Pittsburg-bridges-type	89.03 ± 3.97	67.18 ± 9.43	65.56 ± 7.28	58.61 ± 14.73	60.67 ± 16.06	50.95 ± 23.20	49.46 ± 17.71	60.92 ± 18.91	59.26 ± 20.97	57.79 ± 7.39
Pittsburg-bridgesmaterial	73.99 ± 3.79	45.14 ± 15.62	47.53 ± 13.35	42.97 ± 27.99	33.41 ± 14.32	35.53 ± 13.89	38.09 ± 22.43	45.06 ± 20.52	52.34 ± 19.88	31.28 ± 10.34
Planning	99.62 ± 2.38	104.13 ± 8.76	99.73 ± 0.02	83.73 ± 22.67	69.71 ± 2.82	72.56 ± 14.76	83.38 ± 17.31	101.14 ± 12.75	105.45 ± 20.71	92.84 ± 21.47
Plant-margin	94.95 ± 0.12	15.73 ± 2.46	54.50 ± 4.17	31.05 ± 2.63	17.36 ± 2.29	15.40 ± 2.83	15.02 ± 2.85	24.09 ± 1.81	23.12 ± 2.51	93.24 ± 2.19
Plant-shape	91.94 ± 0.11	47.32 ± 3.03	55.79 ± 3.25	39.84 ± 3.16	51.57 ± 1.63	43.68 ± 3.16	40.34 ± 3.39	43.86 ± 3.22	41.23 ± 3.03	92.35 ± 1.63
Plant-texture	95.39 ± 0.15	25.87 ± 3.18	50.29 ± 3.39	25.28 ± 2.68	16.86 ± 2.52	14.59 ± 2.31	14.08 ± 2.37	23.84 ± 1.60	22.80 ± 1.59	97.53 ± 0.91
Poker-hand-training-true	99.97 ± 0.01	99.98 ± 0.03	84.40 ± 2.91	93.48 ± 1.51	72.66 ± 1.76	74.66 ± 1.64	76.64 ± 1.96	96.31 ± 1.26	96.86 ± 1.79	88.07 ± 0.03
Post-operative	97.54 ± 2.88	103.29 ± 8.23	97.97 ± 0.37	101.82 ± 20.36	66.34 ± 9.65	71.48 ± 12.99	76.48 ± 17.86	97.20 ± 19.90	101.48 ± 28.67	71.48 ± 12.99
Primary-tumor	95.28 ± 0.69	68.67 ± 6.71	73.20 ± 6.01	76.00 ± 9.25	60.18 ± 12.24	62.24 ± 11.86	63.26 ± 13.42	70.17 ± 7.30	70.44 ± 7.80	81.40 ± 3.14
Qsarbioconcentration	100.17 ± 0.61	100.83 ± 4.02	102.60 ± 5.96	102.26 ± 15.18	66.97 ± 1.48	71.09 ± 4.12	78.25 ± 7.59	104.51 ± 6.05	104.62 ± 4.85	72.41 ± 8.44
Qsarbiodegradation	88.05 ± 1.73	53.37 ± 6.92	42.98 ± 6.42	36.83 ± 5.01	31.57 ± 5.87	30.73 ± 4.85	32.22 ± 5.57	32.16 ± 3.76	32.35 ± 3.67	52.10 ± 8.81
Qualitative-bankruptcy	26.63 ± 1.92	1.79 ± 3.29	4.80 ± 6.53	0.66 ± 0.54	2.46 ± 5.54	2.46 ± 5.54	0.81 ± 2.57	1.60 ± 3.70	1.48 ± 3.65	3.28 ± 5.74
Ringnorm	59.88 ± 2.39	3.92 ± 0.66	18.08 ± 0.37	49.70 ± 3.17	2.81 ± 0.83	3.05 ± 0.82	3.30 ± 0.97	16.74 ± 2.49	17.42 ± 1.69	71.14 ± 1.93
Risk-factors-cervical-cancer	96.16 ± 1.09	92.97 ± 11.30	49.02 ± 12.44	47.55 ± 18.02	52.88 ± 2.93	53.88 ± 5.59	49.80 ± 12.09	41.47 ± 12.51	45.32 ± 16.32	31.89 ± 16.03
Robotnavigation	47.91 ± 1.23	71.45 ± 2.17	0.69 ± 0.37	17.81 ± 1.83	15.05 ± 1.73	13.31 ± 1.48	12.09 ± 1.17	19.84 ± 2.42	19.17 ± 3.47	37.18 ± 2.86
Sapfile	95.19 ± 1.58	77.07 ± 9.48	92.26 ± 18.29	96.60 ± 13.95	79.45 ± 13.75	78.18 ± 10.63	78.15 ± 13.06	81.94 ± 12.13	82.67 ± 16.59	90.31 ± 19.72
Sat	51.32 ± 0.52	25.36 ± 1.49	18.48 ± 1.13	11.75 ± 0.97	91.15 ± 0.66	89.69 ± 0.58	89.69 ± 0.58	13.80 ± 1.71	14.23 ± 1.26	49.67 ± 1.61
Satelite	51.35 ± 0.60	25.36 ± 1.36	18.21 ± 0.85	11.72 ± 1.37	91.17 ± 0.71	89.59 ± 0.88	89.59 ± 0.88	13.49 ± 0.66	14.00 ± 1.03	49.40 ± 2.03
Scadi	84.82 ± 1.64	22.22 ± 11.10	32.14 ± 15.34	35.37 ± 17.03	32.36 ± 12.76	28.64 ± 18.71	24.89 ± 20.41	28.81 ± 14.85	28.22 ± 14.97	55.28 ± 13.83
Schillingdata	82.03 ± 2.00	42.45 ± 2.63	99.92 ± 0.00	63.68 ± 3.93	57.60 ± 0.40	47.78 ± 4.43	36.36 ± 2.87	32.39 ± 8.29	39.05 ± 12.63	58.26 ± 1.72
Seeds	42.07 ± 5.78	14.04 ± 6.27	14.78 ± 8.74	10.00 ± 2.96	9.29 ± 5.88	9.29 ± 5.88	9.29 ± 5.88	9.26 ± 3.26	8.33 ± 3.11	26.43 ± 15.08
Segment	53.80 ± 1.24	23.49 ± 1.80	4.27 ± 1.12	3.65 ± 0.72	40.40 ± 3.36	38.48 ± 2.80	38.54 ± 2.88	6.42 ± 1.05	5.63 ± 0.83	42.17 ± 3.24
Seismic-bumps	98.69 ± 0.40	115.78 ± 13.12	98.60 ± 3.58	86.32 ± 11.80	53.38 ± 0.06	53.38 ± 0.06	53.38 ± 0.06	78.60 ± 8.88	75.65 ± 8.05	57.15 ± 4.41
Semeion	96.78 ± 0.07	15.63 ± 3.03	29.94 ± 2.41	10.00 ± 1.86	4.88 ± 1.19	4.26 ± 0.77	4.33 ± 0.80	9.99 ± 1.85	9.37 ± 1.92	89.35 ± 0.53
Setapprocesst1	93.44 ± 5.93	96.50 ± 43.94	87.03 ± 39.95	81.33 ± 23.35	83.51 ± 18.75	72.54 ± 24.34	76.34 ± 21.09	82.46 ± 42.80	85.72 ± 44.19	64.52 ± 53.46
Setapprocesst10	98.97 ± 3.13	124.35 ± 32.41	94.02 ± 29.97	88.89 ± 36.54	74.50 ± 9.59	74.50 ± 9.59	74.50 ± 9.59	105.91 ± 54.99	105.17 ± 48.88	84.35 ± 17.77
Setapprocesst11	98.56 ± 4.45	101.50 ± 58.38	81.02 ± 38.41	93.61 ± 33.26	74.50 ± 9.59	74.50 ± 9.59	74.50 ± 9.59	86.46 ± 33.25	78.38 ± 37.24	95.80 ± 22.70
Setapprocesst2	97.76 ± 3.58	73.36 ± 30.55	59.92 ± 40.59	83.49 ± 46.02	74.50 ± 9.59	74.50 ± 9.59	74.50 ± 9.59	84.47 ± 38.01	78.61 ± 38.22	41.47 ± 37.02
Setapprocesst3	97.51 ± 3.17	78.93 ± 26.90	88.97 ± 34.49	76.68 ± 30.30	74.50 ± 9.59	74.50 ± 9.59	74.50 ± 9.59	77.26 ± 27.64	77.43 ± 19.43	104.81 ± 36.47
Setapprocesst4	99.87 ± 2.10	122.70 ± 60.90	110.95 ± 35.48	97.13 ± 42.87	68.66 ± 13.00	68.66 ± 13.00	68.66 ± 13.00	109.70 ± 45.59	105.27 ± 38.88	66.22 ± 27.24
Setapprocesst5	96.83 ± 4.53	118.50 ± 38.81	105.16 ± 36.94	90.15 ± 36.20	77.22 ± 14.45	79.94 ± 17.59	79.94 ± 17.59	91.72 ± 26.54	86.41 ± 25.92	77.05 ± 57.09
Setapprocesst6	97.09 ± 2.12	72.69 ± 34.89	78.35 ± 27.56	80.69 ± 31.47	74.50 ± 9.59	74.50 ± 9.59	74.50 ± 9.59	82.28 ± 31.96	83.55 ± 31.72	89.05 ± 46.01
Setapprocesst7	97.36 ± 4.02	57.12 ± 30.93	87.54 ± 43.22	80.30 ± 31.09	74.50 ± 9.59	74.50 ± 9.59	74.50 ± 9.59	62.70 ± 38.99	64.34 ± 39.59	105.09 ± 34.29
Setapprocesst8	96.38 ± 2.61	70.34 ± 32.11	67.05 ± 33.15	83.06 ± 34.00	74.50 ± 9.59	74.50 ± 9.59	74.50 ± 9.59	76.02 ± 35.53	74.51 ± 36.64	113.36 ± 28.85
Setapprocesst9	98.09 ± 2.65	78.22 ± 32.68	70.80 ± 35.69	93.07 ± 20.30	74.50 ± 9.59	74.50 ± 9.59	74.50 ± 9.59	64.42 ± 39.11	73.83 ± 36.60	110.64 ± 39.73
Shillbiddingdataset	64.59 ± 0.97	18.80 ± 1.93	2.83 ± 0.76	4.15 ± 1.48	2.40 ± 0.91	2.32 ± 1.09	1.82 ± 1.02	2.19 ± 0.88	1.92 ± 1.04	14.25 ± 2.80
Shuttle-landing-control	98.09 ± 98.47	153.52 ± 221.31	61.65 ± 13.48	81.09 ± 116.78	10.00 ± 31.62	10.00 ± 31.62	10.00 ± 31.62	94.74 ± 194.49	98.24 ± 206.94	90.00 ± 251.44
Somervillehappinesssurvey2015	93.67 ± 5.90	89.07 ± 11.60	82.68 ± 20.00	83.95 ± 20.26	80.34 ± 21.75	83.02 ± 20.63	82.95 ± 23.46	84.30 ± 21.18	85.77 ± 21.62	69.05 ± 31.84
Sonar	90.81 ± 2.64	62.61 ± 17.91	57.49 ± 14.47	27.74 ± 13.91	68.49 ± 10.87	59.72 ± 18.03	53.03 ± 19.12	38.08 ± 19.26	37.80 ± 18.79	75.34 ± 22.26
Soybean	88.59 ± 0.63	9.44 ± 5.73	15.32 ± 5.03	18.94 ± 4.92	10.70 ± 6.56	8.56 ± 4.87	9.26 ± 5.13	15.90 ± 6.44	15.33 ± 5.95	75.82 ± 2.65
Spambase	84.20 ± 0.69	43.63 ± 3.71	18.72 ± 2.09	19.36 ± 2.26	13.84 ± 1.95	13.61 ± 2.30	13.79 ± 1.93	22.48 ± 2.57	27.47 ± 12.10	45.33 ± 4.18
Speaker-accent	88.94 ± 2.95	65.19 ± 10.75	45.75 ± 13.13	29.60 ± 12.53	59.48 ± 5.54	53.85 ± 6.07	53.85 ± 6.07	36.25 ± 8.70	37.57 ± 9.12	70.82 ± 6.72
Spect	94.05 ± 5.88	70.52 ± 33.64	86.21 ± 21.82	111.23 ± 27.49	79.59 ± 25.42	80.41 ± 27.23	83.14 ± 22.23	105.25 ± 38.65	105.67 ± 38.35	62.49 ± 20.91
Spectf	93.47 ± 4.09	49.03 ± 24.59	56.85 ± 22.77	65.95 ± 36.62	65.00 ± 12.91	65.00 ± 12.91	47.50 ± 27.51	59.54 ± 28.00	57.60 ± 26.15	57.50 ± 16.87
Statlog-australian-credit	98.53 ± 0.69	105.50 ± 4.70	92.82 ± 10.37	101.28 ± 7.68	74.34 ± 4.06	81.65 ± 14.63	82.63 ± 12.80	93.88 ± 7.57	95.03 ± 7.04	79.67 ± 14.60
Statlog-german-credit	97.57 ± 0.32	68.80 ± 7.51	72.76 ± 9.80	76.73 ± 9.35	56.88 ± 9.81	54.02 ± 8.91	54.02 ± 10.53	70.24 ± 8.41	66.81 ± 7.40	69.26 ± 5.43
Statlog-heart	79.99 ± 3.41	37.70 ± 13.10	56.04 ± 16.74	50.66 ± 17.20	34.50 ± 10.72	40.50 ± 17.03	41.25 ± 19.44	45.54 ± 11.90	41.61 ± 15.50	58.49 ± 12.65
Statlog-image	50.97 ± 1.19	23.62 ± 1.87	4.25 ± 1.14	3.65 ± 0.72	6.67 ± 1.03	5.66 ± 1.11	5.30 ± 1.04	6.16 ± 1.00	6.14 ± 1.22	42.37 ± 2.87
Statlog-landsat	50.64 ± 0.88	25.41 ± 2.74	18.67 ± 2.16	12.33 ± 2.10	13.03 ± 1.96	12.13 ± 1.71	12.05 ± 1.99	14.70 ± 1.53	14.52 ± 2.13	48.93 ± 2.08
Statlog-shuttle	72.47 ± 0.54	27.12 ± 1.38	0.14 ± 0.07	0.21 ± 0.10	0.70 ± 0.13	0.57 ± 0.13	0.55 ± 0.12	1.28 ± 0.17	1.26 ± 0.14	14.83 ± 0.84
Statlog-vehicle	77.89 ± 1.57	74.64 ± 3.21	37.89 ± 5.61	40.51 ± 5.94	31.06 ± 5.18	26.33 ± 3.47	26.01 ± 3.15	26.89 ± 3.52	26.91 ± 4.84	65.11 ± 6.80
Steel-plates	74.89 ± 1.28	51.74 ± 4.59	32.78 ± 3.85	36.31 ± 3.49	31.44 ± 3.56	29.59 ± 2.90	29.72 ± 3.74	37.74 ± 2.85	37.67 ± 2.92	66.60 ± 4.99
Synthetic-control	67.81 ± 1.68	6.59 ± 3.88	10.39 ± 4.21	5.25 ± 2.55	0.80 ± 1.03	0.60 ± 0.97	0.60 ± 0.97	2.38 ± 1.17	2.10 ± 1.14	51.60 ± 4.60
Teaching	91.04 ± 3.59	85.30 ± 6.22	65.99 ± 14.73	56.33 ± 19.19	65.76 ± 22.04	57.76 ± 19.01	56.76 ± 18.56	80.76 ± 7.84	80.52 ± 7.84	80.45 ± 16.39
Thoraricsurgery	98.87 ± 1.05	111.80 ± 29.70	99.67 ± 0.22	89.90 ± 23.21	58.48 ± 0.00	60.99 ± 4.04	60.99 ± 4.04	92.81 ± 15.64	86.92 ± 17.56	65.17 ± 6.59
Thyroid	57.77 ± 8.05	7.98 ± 7.66	18.77 ± 13.14	8.02 ± 5.05	51.27 ± 10.32	48.41 ± 9.52	48.41 ± 9.52	9.32 ± 6.57	8.93 ± 6.43	19.81 ± 18.26
Thyroid-train	92.47 ± 1.02	47.14 ± 5.66	2.20 ± 2.26	55.74 ± 9.39	34.50 ± 4.86	30.39 ± 4.22	26.29 ± 4.44	30.70 ± 6.72	35.07 ± 10.96	24.80 ± 5.47
Tic-tac-toe	96.31 ± 0.52	82.35 ± 1.30	15.71 ± 4.65	0.18 ± 0.01	3.00 ± 3.10	1.62 ± 2.20	0.69 ± 1.57	6.34 ± 2.17	5.45 ± 2.62	66.37 ± 5.27
Titanic	83.80 ± 1.82	71.83 ± 3.90	71.99 ± 3.00	70.67 ± 2.81	49.24 ± 5.01	48.20 ± 4.60	48.20 ± 4.60	73.76 ± 4.95	73.97 ± 6.57	51.21 ± 4.63
Trains	84.82 ± 15.40	73.34 ± 94.66	51.94 ± 84.05	76.67 ± 77.46	73.33 ± 94.67	55.00 ± 88.56	55.00 ± 88.56	50.95 ± 67.29	49.70 ± 68.53	183.33 ± 0.00
Transfusion	96.33 ± 1.56	80.83 ± 5.96	83.64 ± 3.41	87.89 ± 11.10	68.12 ± 7.91	74.38 ± 8.43	77.33 ± 10.26	81.45 ± 4.21	81.29 ± 5.46	65.18 ± 4.59
Trial	68.74 ± 3.53	20.60 ± 7.26	0.00 ± 0.00	0.84 ± 1.75	1.92 ± 1.85	1.37 ± 1.45	1.37 ± 1.45	2.16 ± 1.92	1.78 ± 1.94	0.00 ± 0.00
Turkiye-student-evaluation	93.92 ± 0.34	27.54 ± 2.76	0.06 ± 0.13	17.88 ± 2.93	8.37 ± 0.97	6.93 ± 1.10	6.93 ± 1.10	0.20 ± 0.02	0.14 ± 0.02	0.06 ± 0.13
Unbalanced	95.88 ± 1.76	389.07 ± 168.32	95.69 ± 0.55	87.80 ± 59.75	47.45 ± 8.26	56.16 ± 18.15	63.68 ± 22.74	88.33 ± 22.73	82.28 ± 28.37	47.45 ± 8.26
Urbanlandcover	80.42 ± 3.34	23.92 ± 10.79	25.59 ± 7.52	30.66 ± 11.82	93.85 ± 2.26	93.85 ± 2.26	93.85 ± 2.26	27.55 ± 10.22	27.18 ± 10.37	57.61 ± 16.50
Userknowledgemodeling	75.33 ± 5.18	30.86 ± 5.69	11.70 ± 4.63	28.66 ± 14.08	31.26 ± 9.01	20.64 ± 6.01	20.09 ± 5.50	12.98 ± 4.39	11.14 ± 5.47	20.65 ± 11.01
Vehicle	77.45 ± 1.66	75.41 ± 3.51	37.74 ± 5.74	40.51 ± 5.94	92.71 ± 3.38	91.61 ± 4.09	91.61 ± 4.09	27.03 ± 4.00	26.45 ± 5.37	64.16 ± 6.72
Vertebral-column-2classes	78.84 ± 9.07	50.23 ± 15.73	47.01 ± 14.02	41.81 ± 11.55	33.18 ± 12.17	35.39 ± 14.25	34.65 ± 13.03	41.47 ± 9.67	40.32 ± 9.74	59.72 ± 17.53
Vertebral-column-3classes	66.41 ± 4.10	33.87 ± 5.65	32.10 ± 9.04	34.85 ± 7.69	25.81 ± 9.43	24.27 ± 11.17	25.30 ± 10.73	31.61 ± 5.37	30.66 ± 5.10	39.75 ± 11.17
Veteran	92.97 ± 1.93	75.26 ± 12.85	80.96 ± 8.67	95.26 ± 21.20	70.42 ± 1.88	70.42 ± 1.88	70.42 ± 1.88	93.54 ± 29.30	81.84 ± 28.68	63.59 ± 17.95
Vowel	80.68 ± 1.05	51.58 ± 3.76	21.88 ± 5.27	1.99 ± 0.90	16.44 ± 2.86	9.78 ± 2.21	7.78 ± 2.28	12.32 ± 3.37	10.10 ± 2.58	74.78 ± 4.63
Wall-following	48.03 ± 1.15	71.46 ± 1.43	0.60 ± 0.39	17.92 ± 1.83	16.60 ± 2.13	14.19 ± 2.39	13.06 ± 1.96	20.34 ± 2.17	19.91 ± 2.62	36.77 ± 2.15
Waveform-noise	82.77 ± 0.94	30.52 ± 2.17	38.30 ± 1.82	39.61 ± 1.90	20.79 ± 2.27	21.93 ± 1.92	22.65 ± 2.12	24.73 ± 2.35	25.51 ± 2.03	69.42 ± 4.12
Waveform	75.61 ± 0.91	29.23 ± 1.85	38.13 ± 1.95	34.70 ± 3.02	20.37 ± 2.04	21.51 ± 2.07	21.81 ± 2.15	24.51 ± 1.76	25.52 ± 1.87	71.20 ± 2.46
Wbc	21.48 ± 3.02	5.64 ± 3.13	19.31 ± 4.17	10.08 ± 4.61	5.15 ± 3.47	5.15 ± 3.47	4.83 ± 3.81	9.33 ± 3.05	8.55 ± 4.04	19.64 ± 6.41
Wdbc	52.63 ± 4.72	14.94 ± 8.88	15.44 ± 8.53	9.03 ± 3.99	79.67 ± 1.28	79.67 ± 1.28	79.67 ± 1.28	7.46 ± 3.60	8.46 ± 4.45	21.79 ± 6.79
Weathernominal	99.31 ± 34.07	89.36 ± 34.03	79.58 ± 76.74	105.12 ± 59.86	55.00 ± 59.86	80.00 ± 82.33	55.00 ± 83.17	55.18 ± 81.59	55.09 ± 82.12	115.00 ± 97.33
Weathernumeric	117.85 ± 31.20	94.85 ± 27.94	51.25 ± 72.28	53.86 ± 72.31	55.00 ± 59.86	100.00 ± 97.18	100.00 ± 97.18	57.19 ± 82.33	58.08 ± 82.85	115.00 ± 97.33
Website-phishingdata	77.13 ± 1.04	37.13 ± 3.15	22.20 ± 3.01	24.98 ± 3.42	25.55 ± 2.26	24.63 ± 2.13	22.00 ± 3.66	22.88 ± 3.32	21.51 ± 3.94	32.53 ± 2.92
Wholesalecustomersdata	52.14 ± 4.41	27.19 ± 6.34	30.44 ± 6.43	27.98 ± 10.93	73.76 ± 1.66	73.76 ± 1.66	73.76 ± 1.66	24.95 ± 5.71	24.87 ± 5.84	21.82 ± 8.40
Wifi-localization	53.31 ± 0.96	3.08 ± 0.75	4.59 ± 0.68	2.48 ± 1.18	27.93 ± 3.32	26.07 ± 3.43	26.07 ± 3.43	3.69 ± 0.48	3.36 ± 0.70	28.00 ± 5.94
Wilt	98.25 ± 1.77	169.09 ± 9.21	21.81 ± 4.42	50.33 ± 5.73	51.14 ± 1.29	51.14 ± 1.29	51.14 ± 1.29	26.92 ± 4.66	22.69 ± 3.57	54.17 ± 1.88
Wine-quality-red	85.83 ± 2.01	82.11 ± 3.21	63.71 ± 3.92	55.01 ± 4.21	57.83 ± 3.82	57.24 ± 4.11	57.05 ± 4.14	77.23 ± 3.54	75.38 ± 3.21	70.46 ± 4.77
Wine-quality-white	90.26 ± 0.40	89.15 ± 0.96	65.18 ± 3.20	51.32 ± 3.70	63.63 ± 3.38	62.36 ± 3.65	62.00 ± 3.90	82.93 ± 1.44	82.53 ± 1.38	80.28 ± 3.17
Wine	52.96 ± 3.13	5.09 ± 7.02	11.00 ± 7.84	9.36 ± 6.11	1.68 ± 3.55	2.58 ± 4.16	2.58 ± 4.16	5.66 ± 4.98	5.43 ± 5.11	34.99 ± 14.01
Yamilnaduelectricty	92.30 ± 0.69	100.00 ± 0.00	92.08 ± 0.89	83.42 ± 1.38	98.78 ± 0.58	98.61 ± 0.59	98.74 ± 0.64	99.98 ± 0.04	99.98 ± 0.05	97.94 ± 1.18
Yeast	91.42 ± 0.61	67.16 ± 2.46	65.36 ± 4.00	61.75 ± 3.05	51.13 ± 5.26	51.04 ± 5.46	50.87 ± 5.80	65.48 ± 2.40	65.15 ± 2.53	77.12 ± 4.38
Youtobe-kabita-preprocessing	92.32 ± 0.36	86.08 ± 0.85	83.77 ± 1.30	83.17 ± 1.38	73.12 ± 2.76	74.40 ± 2.56	75.21 ± 2.46	87.94 ± 0.65	87.78 ± 0.65	78.90 ± 2.34
Youtobe-nisha-preprocessing	91.64 ± 0.26	85.67 ± 0.84	81.03 ± 0.87	80.28 ± 1.97	69.48 ± 2.48	70.90 ± 2.45	71.38 ± 2.60	86.17 ± 0.67	86.08 ± 0.95	80.24 ± 1.53
Z-alizadehsani	95.90 ± 1.24	48.74 ± 14.11	57.45 ± 18.02	53.44 ± 16.65	69.98 ± 2.67	69.98 ± 2.67	69.98 ± 2.67	42.10 ± 17.85	43.58 ± 15.44	71.56 ± 12.76
Zoo	65.90 ± 3.25	5.23 ± 8.36	10.06 ± 11.09	8.68 ± 6.54	7.54 ± 10.51	7.54 ± 10.51	7.54 ± 10.51	8.97 ± 7.18	7.92 ± 7.07	34.76 ± 13.50
Average rae (rank)	79.08 (10)	60.74 (9)	44.76 (3)	45.24 (4)	49.04 (7)	47.95 (6)	47.48 (5)	43.61 (2)	43.27 (1)	56.74 (8)
Average rae std (rank)	3.21 (1)	10.91 (7)	9.45 (5)	11.66 (9)	7.55 (2)	8.53 (3)	8.75 (4)	10.86 (6)	11.51 (8)	11.75 (10)

**Table 11:** The detailed experimental results on root relative squared error and standard deviation.

Dataset	RN	NB	J48	KNN	SVM1	SVM2	SVM3	ANN1	ANN2	OneR
Abalone	87.04 ± 1.45	98.65 ± 3.48	96.11 ± 1.73	112.29 ± 3.33	100.03 ± 3.71	99.50 ± 3.56	99.58 ± 3.38	81.78 ± 1.93	82.19 ± 2.46	108.24 ± 2.48
Absenteeism-at-work	64.13 ± 1.29	65.75 ± 7.93	5.26 ± 9.63	81.19 ± 7.45	78.51 ± 8.03	78.51 ± 8.03	78.51 ± 8.03	29.02 ± 6.25	29.45 ± 8.61	43.21 ± 8.16
Acute-inflammation	49.79 ± 4.91	30.75 ± 6.75	0.00 ± 0.00	1.68 ± 0.08	0.00 ± 0.00	0.00 ± 0.00	0.00 ± 0.00	1.23 ± 0.11	0.89 ± 0.09	89.41 ± 19.34
Acute-nephritis	44.00 ± 2.74	28.44 ± 26.94	0.00 ± 0.00	1.69 ± 0.08	0.00 ± 0.00	0.00 ± 0.00	0.00 ± 0.00	1.23 ± 0.14	0.98 ± 0.16	44.83 ± 39.70
Adult	94.87 ± 0.89	94.07 ± 7.03	88.25 ± 6.56	108.54 ± 8.94	95.11 ± 11.86	94.77 ± 11.64	94.93 ± 11.80	89.31 ± 8.09	90.37 ± 7.98	103.82 ± 3.43
Aggregation	41.47 ± 7.37	5.51 ± 3.22	5.39 ± 8.69	2.73 ± 5.41	1.82 ± 5.75	3.62 ± 7.64	3.62 ± 7.64	16.58 ± 4.55	15.24 ± 6.30	101.51 ± 3.69
Algerianforest	67.39 ± 5.59	38.32 ± 28.13	32.55 ± 26.76	66.75 ± 19.67	129.31 ± 2.58	124.52 ± 6.88	124.52 ± 6.88	29.20 ± 20.27	23.61 ± 23.09	18.48 ± 30.00
Annealing	92.46 ± 0.55	110.20 ± 10.34	37.23 ± 13.89	71.37 ± 8.09	73.64 ± 10.23	64.64 ± 9.73	62.34 ± 8.38	58.54 ± 14.28	57.72 ± 12.39	90.27 ± 0.06
Arrhythmia	97.62 ± 0.51	105.27 ± 9.70	95.99 ± 10.56	115.41 ± 4.38	106.37 ± 3.39	100.94 ± 5.15	102.18 ± 6.11	89.64 ± 8.21	101.99 ± 11.41	109.41 ± 3.31
Au1-1000	99.16 ± 0.18	97.22 ± 2.15	97.74 ± 9.49	116.84 ± 8.88	116.17 ± 0.26	116.17 ± 0.26	115.48 ± 1.85	121.39 ± 7.93	116.95 ± 8.29	116.17 ± 0.26
Au4-2500	95.57 ± 0.69	98.30 ± 2.76	93.63 ± 8.56	113.00 ± 7.26	116.52 ± 4.28	116.52 ± 4.28	116.52 ± 4.28	101.91 ± 5.90	102.40 ± 6.97	127.86 ± 5.57
Au6-1000	100.19 ± 0.35	103.39 ± 0.83	130.38 ± 3.17	141.35 ± 3.10	133.03 ± 0.02	133.03 ± 0.02	133.03 ± 0.02	129.97 ± 2.01	131.94 ± 3.22	132.76 ± 1.94
Au6-250-drift-au6-cd1-500	100.42 ± 0.60	104.58 ± 1.76	130.17 ± 3.00	139.11 ± 3.50	135.09 ± 0.52	135.09 ± 0.52	135.09 ± 0.52	128.72 ± 1.87	128.09 ± 3.59	132.84 ± 3.07
Au6-cd1-400	100.02 ± 0.56	107.06 ± 3.95	116.33 ± 6.32	139.16 ± 6.89	131.99 ± 0.50	131.99 ± 0.50	131.99 ± 0.50	124.44 ± 5.63	125.69 ± 4.62	128.15 ± 6.26
Au7-300-drift-au7-cpd1-800	99.03 ± 0.63	97.68 ± 1.41	114.95 ± 4.82	130.95 ± 4.15	134.79 ± 1.52	135.47 ± 1.81	135.47 ± 1.81	106.21 ± 3.72	106.22 ± 3.32	137.99 ± 2.84
Au7-700	98.90 ± 1.36	98.55 ± 2.70	111.03 ± 4.59	138.48 ± 6.11	137.25 ± 3.13	138.19 ± 2.55	138.19 ± 2.55	113.58 ± 3.03	111.08 ± 3.93	128.82 ± 4.47
Au7-cpd1-500	99.46 ± 0.85	98.56 ± 2.77	102.46 ± 6.33	128.08 ± 5.92	128.48 ± 0.87	128.06 ± 1.63	128.06 ± 1.63	115.19 ± 4.86	115.27 ± 6.42	126.30 ± 5.00
Audiology-std	97.30 ± 0.37	71.56 ± 13.39	66.74 ± 15.49	72.36 ± 17.49	92.29 ± 10.19	85.55 ± 15.05	81.75 ± 12.99	55.26 ± 10.04	58.45 ± 7.30	110.81 ± 2.16
Audit-risk	62.32 ± 3.32	45.95 ± 9.60	2.31 ± 7.32	28.16 ± 11.64	20.84 ± 15.36	17.56 ± 15.99	15.22 ± 16.74	31.35 ± 11.06	32.12 ± 10.84	0.00 ± 0.00
Autism-adolescent-data	79.76 ± 5.66	21.72 ± 14.92	0.00 ± 0.00	55.68 ± 30.30	25.41 ± 32.81	31.66 ± 33.38	25.41 ± 32.81	47.83 ± 37.02	47.60 ± 37.80	0.00 ± 0.00
Autism-adult-data	76.53 ± 2.98	27.93 ± 13.51	0.00 ± 0.00	47.57 ± 19.04	11.90 ± 15.68	6.49 ± 13.93	9.17 ± 15.07	1.85 ± 1.12	1.61 ± 1.08	0.00 ± 0.00
Autism-child-data	81.09 ± 2.35	20.96 ± 7.07	0.00 ± 0.00	66.42 ± 11.51	3.72 ± 11.75	0.00 ± 0.00	0.00 ± 0.00	6.57 ± 7.91	7.57 ± 8.33	0.00 ± 0.00
Autos	79.44 ± 4.00	93.69 ± 15.49	59.87 ± 12.16	74.84 ± 18.73	130.14 ± 4.27	129.13 ± 5.18	129.13 ± 5.18	69.30 ± 10.55	69.54 ± 10.22	98.15 ± 12.45
Avila	55.95 ± 0.61	113.05 ± 2.00	36.89 ± 3.61	79.05 ± 4.37	91.07 ± 3.03	87.94 ± 2.73	85.79 ± 3.46	81.95 ± 1.65	81.87 ± 2.52	85.67 ± 3.79
Balance-scale	78.97 ± 2.17	64.09 ± 1.77	84.72 ± 6.53	74.91 ± 9.18	57.92 ± 4.57	50.23 ± 3.06	47.21 ± 5.01	46.13 ± 7.33	47.42 ± 7.76	123.73 ± 6.26
Balloons	84.28 ± 22.19	100.00 ± 0.00	101.11 ± 32.98	73.52 ± 19.16	73.13 ± 77.59	45.07 ± 73.01	45.07 ± 73.01	75.07 ± 65.85	73.43 ± 68.31	132.51 ± 76.43
Bank	96.18 ± 0.62	111.16 ± 4.14	97.70 ± 3.91	116.66 ± 5.44	101.26 ± 3.03	100.51 ± 2.80	99.77 ± 5.34	92.83 ± 4.15	94.47 ± 6.13	105.87 ± 2.83
Blood	97.90 ± 1.24	100.92 ± 5.35	93.54 ± 3.96	124.81 ± 9.96	108.82 ± 5.05	107.53 ± 4.02	108.89 ± 3.54	92.25 ± 2.87	92.58 ± 3.13	114.18 ± 4.07
Breast-cancer-wisc-diag	58.60 ± 4.87	49.18 ± 21.88	48.81 ± 21.01	40.40 ± 10.17	27.81 ± 17.56	25.83 ± 16.18	24.22 ± 13.55	35.55 ± 11.22	34.82 ± 15.56	67.21 ± 12.49
Breast-cancer-wisc-prog	98.67 ± 2.27	122.29 ± 24.41	111.32 ± 19.13	120.18 ± 22.65	105.85 ± 15.90	107.68 ± 24.75	108.63 ± 25.97	109.19 ± 20.51	111.40 ± 23.50	127.77 ± 17.95
Breast-cancer-wisc	42.39 ± 7.53	41.10 ± 9.69	49.02 ± 14.54	40.44 ± 18.99	31.30 ± 17.84	32.10 ± 18.23	35.28 ± 15.28	39.94 ± 20.14	38.95 ± 15.35	54.13 ± 18.22
Breast-cancer	94.83 ± 2.43	99.22 ± 10.73	101.11 ± 7.07	117.59 ± 16.06	111.32 ± 11.38	112.77 ± 11.87	115.11 ± 10.81	104.46 ± 11.90	110.64 ± 7.47	118.69 ± 9.24
Breast-tissue	76.84 ± 8.01	77.80 ± 20.83	79.52 ± 23.21	75.89 ± 27.92	98.27 ± 20.29	84.92 ± 24.48	78.96 ± 33.89	75.31 ± 11.32	77.58 ± 13.99	104.08 ± 11.32
Bupa	96.09 ± 2.82	103.16 ± 7.94	109.46 ± 13.07	124.65 ± 8.26	129.90 ± 3.37	129.43 ± 3.63	129.43 ± 3.63	94.47 ± 8.36	94.72 ± 8.45	136.60 ± 11.34
Caesarian	93.60 ± 11.35	96.05 ± 22.13	99.08 ± 10.67	116.36 ± 24.92	135.09 ± 8.43	122.67 ± 20.60	127.78 ± 26.66	122.23 ± 31.46	121.16 ± 27.44	142.18 ± 31.33
Car	95.36 ± 0.79	69.09 ± 2.61	29.29 ± 7.47	36.24 ± 6.01	32.85 ± 8.17	27.62 ± 8.94	26.08 ± 7.74	43.50 ± 5.29	44.30 ± 5.80	114.50 ± 0.15
Cardiotocography-10classes	84.03 ± 0.62	75.38 ± 3.86	60.30 ± 4.24	70.89 ± 4.93	66.78 ± 3.77	64.57 ± 4.93	63.32 ± 3.90	58.39 ± 5.07	60.51 ± 4.16	111.71 ± 2.42
Cardiotocography-3classes	89.05 ± 1.39	94.05 ± 9.53	59.47 ± 10.72	65.38 ± 7.50	69.32 ± 7.48	65.19 ± 8.80	64.76 ± 7.36	59.43 ± 4.46	58.51 ± 8.59	100.16 ± 8.65
Cervical-cancer	84.43 ± 6.46	51.34 ± 37.84	67.07 ± 48.21	57.33 ± 48.43	118.53 ± 2.39	111.60 ± 14.93	111.60 ± 14.93	36.13 ± 34.48	36.27 ± 35.10	101.32 ± 19.29
Chemicalcomposionofceramic	50.06 ± 4.87	0.00 ± 0.00	7.07 ± 22.36	2.46 ± 0.01	147.37 ± 3.13	147.37 ± 3.13	147.37 ± 3.13	1.52 ± 1.09	1.15 ± 0.90	0.00 ± 0.00
Chess-krvk	96.95 ± 0.21	95.51 ± 0.62	92.34 ± 3.10	105.32 ± 3.35	109.87 ± 2.65	107.60 ± 2.33	105.98 ± 3.07	88.64 ± 1.29	90.21 ± 1.38	128.99 ± 1.82
Chess-krvkp	95.07 ± 0.24	63.94 ± 3.46	14.61 ± 7.45	46.19 ± 4.46	21.81 ± 7.50	16.25 ± 8.28	17.13 ± 6.00	14.65 ± 6.53	14.17 ± 7.25	115.92 ± 2.50
Congressional-voting	99.08 ± 0.56	98.72 ± 8.37	100.02 ± 4.89	102.46 ± 10.65	127.09 ± 6.41	127.74 ± 8.38	128.44 ± 9.28	100.83 ± 7.17	100.21 ± 9.10	124.89 ± 4.38
Conn-bench-sonar-mines-rocks	91.67 ± 2.69	105.49 ± 14.07	103.66 ± 12.82	70.39 ± 20.51	77.67 ± 18.97	66.81 ± 28.73	62.39 ± 28.49	76.31 ± 27.58	72.57 ± 27.11	119.98 ± 18.01
Conn-bench-vowel-deterding	75.28 ± 0.72	71.22 ± 4.93	64.24 ± 9.74	8.49 ± 10.29	35.40 ± 6.57	20.36 ± 11.86	13.93 ± 12.42	52.47 ± 6.14	57.37 ± 6.61	119.12 ± 4.56
Connect-4	99.57 ± 0.06	109.71 ± 2.95	94.21 ± 4.89	122.65 ± 5.94	116.17 ± 0.57	112.39 ± 2.25	109.60 ± 4.18	108.75 ± 5.03	106.76 ± 4.24	115.97 ± 0.29
Connectionist	91.28 ± 2.65	106.31 ± 19.31	98.82 ± 14.67	69.96 ± 16.79	123.85 ± 13.85	107.55 ± 25.61	98.53 ± 37.54	72.27 ± 22.00	72.77 ± 21.46	121.69 ± 11.57
Contrac	97.07 ± 0.63	98.84 ± 3.76	101.01 ± 3.41	130.99 ± 4.40	118.62 ± 2.87	118.74 ± 4.60	119.53 ± 4.97	93.08 ± 2.38	93.39 ± 2.68	126.84 ± 2.43
Covid-19	87.72 ± 14.61	79.48 ± 43.86	73.28 ± 73.08	87.99 ± 54.30	82.96 ± 72.68	91.62 ± 83.90	75.86 ± 84.92	70.16 ± 68.24	69.60 ± 71.22	40.14 ± 65.67
Credit-approval	85.23 ± 2.41	87.36 ± 7.07	71.92 ± 8.63	83.63 ± 15.02	75.69 ± 14.75	76.80 ± 12.65	77.99 ± 12.32	71.42 ± 7.89	74.32 ± 8.93	75.63 ± 12.80
Crowdsource	78.25 ± 1.13	78.56 ± 5.38	65.54 ± 4.88	43.75 ± 4.47	100.86 ± 2.14	100.86 ± 2.14	100.86 ± 2.14	51.09 ± 6.51	51.84 ± 7.83	94.05 ± 4.77
Crx	84.30 ± 2.50	87.46 ± 6.08	66.22 ± 8.16	86.43 ± 12.22	133.68 ± 5.46	132.53 ± 6.48	130.46 ± 7.92	77.27 ± 10.24	74.64 ± 6.95	75.63 ± 12.80
Cryother	69.03 ± 11.06	61.61 ± 31.47	40.21 ± 30.25	52.56 ± 36.30	77.05 ± 35.77	74.27 ± 35.31	74.27 ± 35.31	55.57 ± 26.27	64.64 ± 24.75	84.20 ± 23.42
Cylinder-bands	94.25 ± 1.70	109.94 ± 11.74	102.49 ± 12.31	112.91 ± 13.07	100.60 ± 7.85	93.26 ± 9.81	88.51 ± 7.15	98.44 ± 10.03	97.40 ± 9.04	118.22 ± 7.65
Dbworld-bodies	97.96 ± 0.74	91.88 ± 39.78	72.99 ± 50.38	124.94 ± 15.83	134.97 ± 7.40	134.97 ± 7.40	118.84 ± 21.59	/	/	56.43 ± 50.38
Dbworld-bodies-stemmed	97.80 ± 0.83	90.04 ± 36.82	55.89 ± 44.18	115.89 ± 24.62	134.97 ± 7.40	127.54 ± 14.01	100.12 ± 24.08	/	/	88.13 ± 53.62
Dbworld-subjects	98.03 ± 0.74	55.45 ± 29.67	89.11 ± 19.44	84.40 ± 30.41	134.97 ± 7.40	134.97 ± 7.40	134.97 ± 7.40	49.62 ± 40.30	50.30 ± 45.60	120.97 ± 20.46
Dbworld-subjects-stemmed	98.04 ± 0.93	54.72 ± 31.84	78.89 ± 33.77	74.88 ± 28.64	134.97 ± 7.40	134.97 ± 7.40	134.97 ± 7.40	52.27 ± 39.33	54.64 ± 39.86	124.42 ± 15.79
Dermatology	75.32 ± 0.66	18.33 ± 14.16	25.26 ± 16.27	28.11 ± 19.83	18.90 ± 16.82	16.26 ± 17.56	16.01 ± 17.82	19.29 ± 8.81	19.56 ± 9.41	112.15 ± 2.54
Diabetes	90.33 ± 4.04	87.05 ± 8.50	93.05 ± 10.72	114.11 ± 9.22	123.93 ± 0.35	123.93 ± 0.35	123.93 ± 0.35	88.21 ± 5.91	88.85 ± 7.35	111.61 ± 10.35
Diabetic	94.35 ± 1.52	130.35 ± 3.28	102.47 ± 6.98	124.28 ± 7.54	129.33 ± 7.61	127.28 ± 9.96	127.18 ± 9.43	86.96 ± 7.13	87.10 ± 7.01	136.76 ± 8.48
Divorce	30.00 ± 11.71	19.40 ± 25.05	34.83 ± 28.26	17.58 ± 22.38	19.40 ± 25.05	19.40 ± 25.05	19.40 ± 25.05	16.16 ± 20.73	16.27 ± 21.08	32.64 ± 30.09
Dota2train	99.85 ± 0.08	104.85 ± 3.87	119.95 ± 7.33	123.48 ± 5.37	137.75 ± 0.47	136.25 ± 5.32	137.84 ± 4.80	130.90 ± 14.23	128.88 ± 16.59	133.68 ± 5.46
Dow-jones-index	93.85 ± 2.66	110.18 ± 6.50	92.93 ± 6.89	134.37 ± 9.24	138.58 ± 0.30	138.58 ± 0.30	138.58 ± 0.30	105.45 ± 6.32	107.80 ± 5.42	131.99 ± 4.61
Dry-bean-dataset	40.16 ± 1.36	46.24 ± 3.30	39.11 ± 2.97	36.00 ± 3.59	96.74 ± 3.46	96.74 ± 3.46	96.74 ± 3.46	37.71 ± 3.56	38.09 ± 3.06	87.48 ± 2.65
Early-stage-diabetes-data-upload	80.52 ± 2.36	64.82 ± 9.54	38.85 ± 12.41	21.90 ± 19.27	44.74 ± 22.01	42.04 ± 21.76	37.91 ± 23.87	30.79 ± 14.39	32.21 ± 16.84	85.87 ± 10.59
Echocardiogram	91.63 ± 5.20	85.00 ± 32.69	89.39 ± 24.07	105.49 ± 24.24	85.62 ± 32.14	86.34 ± 36.76	86.34 ± 36.76	89.68 ± 23.05	88.82 ± 26.16	78.84 ± 17.81
Ecoli	79.44 ± 1.57	53.34 ± 10.91	59.99 ± 16.71	71.62 ± 12.14	59.84 ± 10.81	59.94 ± 10.23	58.76 ± 8.76	56.03 ± 7.79	58.51 ± 7.77	94.81 ± 10.09
Eegeyesate	92.77 ± 0.51	140.59 ± 12.34	75.92 ± 2.30	81.12 ± 5.42	134.69 ± 0.04	134.63 ± 0.10	134.63 ± 0.10	100.04 ± 2.91	100.40 ± 3.71	122.94 ± 1.94
Electrical	74.59 ± 7.33	28.67 ± 13.76	0.00 ± 0.00	57.61 ± 24.10	86.54 ± 18.21	86.90 ± 16.65	84.50 ± 15.91	17.48 ± 14.95	16.37 ± 13.97	3.84 ± 12.15
Energy-y1	67.33 ± 1.11	66.60 ± 4.27	25.99 ± 7.16	87.63 ± 7.32	60.16 ± 3.73	56.88 ± 4.79	56.45 ± 8.82	53.37 ± 3.02	53.97 ± 4.10	70.36 ± 4.26
Energy-y2	62.98 ± 1.82	59.28 ± 5.49	49.15 ± 6.73	86.64 ± 6.10	57.05 ± 5.91	54.18 ± 4.53	52.63 ± 4.43	46.47 ± 5.18	45.91 ± 6.73	60.36 ± 8.18
Extentionofz-alizadehsani	93.28 ± 1.09	50.69 ± 15.96	4.91 ± 12.18	65.58 ± 17.91	118.38 ± 1.28	118.38 ± 1.28	118.38 ± 1.28	19.52 ± 19.64	19.47 ± 20.01	79.75 ± 16.62
Fertility	99.64 ± 2.50	103.14 ± 8.97	110.92 ± 23.57	116.09 ± 51.45	105.82 ± 2.00	95.33 ± 33.55	89.19 ± 48.84	87.80 ± 34.79	90.72 ± 44.89	105.82 ± 2.00
First-order	97.73 ± 1.16	278.38 ± 9.70	0.00 ± 0.00	21.63 ± 15.46	105.14 ± 0.20	71.37 ± 13.00	45.13 ± 19.14	6.20 ± 9.73	5.63 ± 9.67	103.00 ± 10.21
Flags	93.17 ± 1.73	103.09 ± 6.74	93.28 ± 12.85	114.36 ± 9.25	108.36 ± 10.53	106.34 ± 11.56	107.13 ± 14.89	103.62 ± 13.05	101.17 ± 9.90	103.01 ± 8.41
Foresttypes	65.19 ± 3.23	29.20 ± 21.71	28.54 ± 23.94	29.29 ± 19.57	136.89 ± 2.30	134.86 ± 3.38	134.86 ± 3.38	24.10 ± 16.45	24.09 ± 17.25	67.11 ± 17.23
Garments-worker-productivity	95.85 ± 0.53	105.03 ± 2.13	104.73 ± 4.85	139.38 ± 3.22	130.58 ± 3.01	129.99 ± 3.04	129.23 ± 2.59	99.04 ± 3.33	100.34 ± 3.44	133.66 ± 3.68
Gender-name-dataset	100.32 ± 0.82	124.83 ± 2.80	100.10 ± 0.15	99.11 ± 2.26	122.30 ± 2.35	122.12 ± 3.46	122.02 ± 3.10	100.56 ± 0.65	100.86 ± 0.87	128.32 ± 1.70
Gesture-a1-raw	54.41 ± 2.09	78.70 ± 4.61	48.63 ± 5.56	32.45 ± 5.22	133.07 ± 0.23	133.07 ± 0.23	133.07 ± 0.23	47.38 ± 6.30	47.42 ± 2.85	30.33 ± 6.80
Gesture-a1-va3	83.35 ± 1.72	104.11 ± 4.61	95.81 ± 6.90	87.08 ± 4.64	133.19 ± 0.13	133.19 ± 0.13	133.19 ± 0.13	82.05 ± 2.58	84.43 ± 4.15	106.40 ± 2.71
Gesture-a2-raw	55.12 ± 1.09	86.90 ± 5.39	53.71 ± 7.04	37.23 ± 12.58	132.00 ± 0.25	132.00 ± 0.25	132.00 ± 0.25	49.13 ± 4.49	48.19 ± 6.09	37.91 ± 9.21
Gesture-a2-va3	87.85 ± 2.04	117.67 ± 10.25	105.65 ± 3.66	95.63 ± 6.11	132.16 ± 0.24	132.16 ± 0.24	132.16 ± 0.24	92.42 ± 4.18	94.44 ± 5.19	114.52 ± 5.11
Gesture-a3-raw	47.60 ± 1.73	91.15 ± 3.28	41.91 ± 3.44	31.34 ± 3.97	131.25 ± 0.15	131.25 ± 0.15	131.25 ± 0.15	48.29 ± 2.93	49.41 ± 5.23	35.29 ± 4.54
Gesture-a3-va3	85.41 ± 0.92	111.26 ± 3.87	77.48 ± 6.03	51.91 ± 4.77	131.35 ± 0.20	131.35 ± 0.20	131.35 ± 0.20	83.48 ± 3.53	82.86 ± 2.97	114.95 ± 2.80
Gesture-b1-raw	51.48 ± 2.62	108.43 ± 4.39	43.24 ± 7.92	36.13 ± 10.17	129.61 ± 0.72	129.41 ± 0.81	129.41 ± 0.81	51.59 ± 6.43	48.95 ± 4.28	48.07 ± 6.62
Gesture-b1-va3	87.25 ± 2.47	128.32 ± 7.15	87.85 ± 5.85	50.33 ± 7.39	129.95 ± 0.31	129.95 ± 0.31	129.95 ± 0.31	87.35 ± 3.96	86.40 ± 6.04	125.54 ± 4.73
Gesture-b3-raw	57.69 ± 2.80	93.30 ± 4.39	46.77 ± 8.60	34.08 ± 3.49	132.38 ± 0.32	132.38 ± 0.32	132.38 ± 0.32	50.93 ± 9.12	52.43 ± 8.73	33.85 ± 8.71
Gesture-b3-va3	93.98 ± 1.41	115.66 ± 3.94	101.47 ± 4.61	75.58 ± 4.98	132.48 ± 0.15	132.48 ± 0.15	132.48 ± 0.15	89.32 ± 4.80	94.06 ± 5.10	125.20 ± 5.78
Gesture-c1-raw	57.89 ± 2.51	86.46 ± 3.70	50.93 ± 5.17	37.44 ± 5.75	137.26 ± 0.36	137.26 ± 0.36	137.26 ± 0.36	50.18 ± 7.09	51.27 ± 8.28	38.92 ± 8.27
Gesture-c1-va3	88.63 ± 2.11	102.77 ± 4.22	98.51 ± 4.20	86.33 ± 5.11	137.48 ± 0.28	137.48 ± 0.28	137.48 ± 0.28	85.44 ± 4.29	89.37 ± 2.79	121.76 ± 4.82
Gesture-c3-raw	56.15 ± 2.12	90.95 ± 5.48	53.79 ± 8.13	41.72 ± 7.02	136.91 ± 0.21	136.91 ± 0.21	136.91 ± 0.21	54.09 ± 5.48	59.42 ± 3.46	37.86 ± 4.86
Gesture-c3-va3	91.31 ± 1.49	110.23 ± 2.36	102.38 ± 4.96	97.90 ± 6.11	136.95 ± 0.18	136.95 ± 0.18	136.95 ± 0.18	91.04 ± 3.87	92.53 ± 5.78	121.37 ± 2.94
Glass	82.19 ± 3.64	102.03 ± 7.89	87.40 ± 12.52	87.15 ± 14.20	85.22 ± 10.46	86.50 ± 12.86	88.00 ± 12.60	80.26 ± 10.18	79.87 ± 6.72	107.44 ± 10.85
Go-track-tracks	73.67 ± 8.97	76.28 ± 12.00	60.07 ± 27.30	48.88 ± 36.66	103.74 ± 22.81	104.08 ± 21.33	104.08 ± 21.33	64.23 ± 18.60	64.32 ± 17.64	95.42 ± 28.22
Haberman-survival	97.97 ± 2.89	98.54 ± 7.58	98.53 ± 3.60	128.48 ± 14.74	118.32 ± 10.07	121.39 ± 5.88	121.39 ± 5.88	97.51 ± 6.19	97.41 ± 6.44	117.85 ± 5.87
Hayes-roth	73.71 ± 3.96	77.38 ± 4.16	59.28 ± 10.77	46.15 ± 10.19	60.55 ± 24.53	75.26 ± 13.64	76.81 ± 13.70	73.09 ± 20.45	72.97 ± 21.55	131.15 ± 6.96
Hcc-data	95.40 ± 2.24	109.37 ± 15.30	110.61 ± 12.20	122.62 ± 13.45	127.07 ± 2.32	127.07 ± 2.32	127.07 ± 2.32	111.77 ± 22.45	114.42 ± 17.38	111.74 ± 24.68
Hcvdat	85.09 ± 2.36	78.59 ± 16.68	76.01 ± 11.37	87.36 ± 7.19	104.58 ± 0.23	104.58 ± 0.23	104.58 ± 0.23	64.82 ± 15.36	71.08 ± 13.04	96.31 ± 6.54
Heart-cleveland	92.89 ± 2.29	94.58 ± 10.47	110.50 ± 10.30	117.14 ± 8.26	114.18 ± 10.92	112.30 ± 11.70	114.03 ± 11.93	105.31 ± 8.01	106.11 ± 8.97	120.91 ± 8.17
Heart-hungarian	81.71 ± 5.53	81.94 ± 15.60	85.06 ± 14.95	96.81 ± 17.91	87.03 ± 19.27	85.86 ± 16.91	83.96 ± 18.04	86.49 ± 16.07	87.92 ± 11.64	92.81 ± 23.08
Heart-switzerland	98.89 ± 3.07	106.19 ± 8.26	118.09 ± 14.35	133.59 ± 13.68	130.40 ± 6.78	129.62 ± 6.35	127.68 ± 7.19	111.78 ± 13.06	111.48 ± 11.46	137.20 ± 13.31
Heart-va	98.75 ± 1.41	106.74 ± 8.88	121.36 ± 6.10	128.15 ± 7.90	130.27 ± 9.54	132.80 ± 8.65	134.71 ± 9.31	119.46 ± 7.43	121.70 ± 8.19	137.11 ± 9.38
Heart-failure-clinical-records-dataset	94.23 ± 2.04	87.18 ± 8.48	88.56 ± 11.78	124.71 ± 13.39	121.30 ± 1.49	121.30 ± 1.49	121.30 ± 1.49	102.12 ± 11.40	96.17 ± 18.48	79.54 ± 16.67
Hepatitis	93.31 ± 3.52	91.15 ± 25.26	102.30 ± 29.81	101.25 ± 17.53	99.62 ± 19.31	86.24 ± 47.29	86.24 ± 47.29	95.75 ± 28.21	102.81 ± 31.56	125.06 ± 18.48
Hill-valley	102.00 ± 2.42	139.88 ± 9.03	100.30 ± 0.67	138.51 ± 5.04	138.07 ± 9.33	137.99 ± 10.62	136.58 ± 10.06	96.94 ± 3.62	97.73 ± 3.97	144.77 ± 6.83
Hiv1625data	58.63 ± 3.99	51.93 ± 8.51	64.10 ± 7.23	64.25 ± 5.31	65.88 ± 8.70	60.27 ± 9.60	58.62 ± 10.89	47.93 ± 11.52	57.93 ± 12.01	107.86 ± 4.69
Hiv746data	60.56 ± 5.01	52.90 ± 9.46	73.66 ± 8.01	67.27 ± 8.03	63.74 ± 12.49	59.78 ± 10.06	59.09 ± 11.45	50.21 ± 9.03	51.82 ± 11.75	86.83 ± 11.35
Horse-colic	89.18 ± 2.62	97.44 ± 13.26	70.82 ± 16.01	98.76 ± 16.26	82.97 ± 13.38	80.16 ± 14.45	78.96 ± 16.19	90.78 ± 10.49	87.85 ± 19.79	86.54 ± 12.50
Htru	55.10 ± 3.61	78.10 ± 3.57	47.16 ± 5.91	58.46 ± 3.99	103.17 ± 0.83	101.20 ± 1.13	101.76 ± 1.07	44.44 ± 4.82	44.93 ± 4.79	53.58 ± 4.38
Hypothyroid	93.63 ± 1.15	72.24 ± 6.74	19.79 ± 9.48	107.70 ± 9.64	100.96 ± 1.88	99.96 ± 4.87	100.12 ± 5.11	81.22 ± 19.04	87.35 ± 21.40	71.52 ± 8.75
Ibeacon-rssi-labeled	97.31 ± 0.22	107.16 ± 1.47	94.52 ± 2.03	89.22 ± 2.04	115.45 ± 1.48	113.31 ± 2.53	112.23 ± 2.83	91.45 ± 1.53	91.23 ± 1.74	139.03 ± 1.26
Ilpd-indian-liver	96.22 ± 2.81	144.80 ± 8.99	103.60 ± 6.40	130.56 ± 11.37	118.37 ± 0.65	119.76 ± 3.59	119.36 ± 5.14	93.07 ± 3.12	93.10 ± 3.39	128.80 ± 6.22
Image-segmentation	65.23 ± 2.86	69.31 ± 9.03	45.94 ± 22.03	53.06 ± 10.03	54.39 ± 6.81	53.90 ± 10.25	54.96 ± 10.01	39.77 ± 8.76	42.53 ± 9.91	97.18 ± 11.10
Immunotherapy	93.17 ± 5.79	97.62 ± 21.85	91.37 ± 30.22	124.97 ± 54.46	111.97 ± 4.45	111.97 ± 4.45	111.97 ± 4.45	91.06 ± 29.58	95.88 ± 31.63	89.90 ± 37.42
Impensdata	83.51 ± 2.93	70.69 ± 8.24	100.00 ± 0.00	90.49 ± 9.22	108.93 ± 0.21	108.93 ± 0.21	108.93 ± 0.21	80.25 ± 9.64	85.97 ± 11.92	108.35 ± 6.83
In-vehicle-coupon-recommendation	97.92 ± 0.12	95.44 ± 1.65	92.34 ± 1.19	112.19 ± 2.23	112.59 ± 2.38	111.63 ± 2.38	110.86 ± 2.41	104.27 ± 1.75	103.05 ± 2.88	126.84 ± 1.42
Indian	95.31 ± 2.20	144.46 ± 9.23	105.80 ± 8.41	131.74 ± 12.56	116.21 ± 3.38	117.66 ± 3.01	117.66 ± 3.01	94.53 ± 4.15	94.71 ± 3.40	129.12 ± 6.10
Ionosphere	62.23 ± 4.81	81.13 ± 12.78	59.31 ± 12.13	75.60 ± 13.65	48.69 ± 10.28	43.50 ± 18.93	39.50 ± 17.90	60.43 ± 6.34	59.15 ± 11.10	88.58 ± 22.85
Iris	59.06 ± 8.72	30.32 ± 17.48	22.67 ± 26.19	26.88 ± 26.91	22.36 ± 23.57	24.21 ± 26.11	22.36 ± 23.57	20.87 ± 18.68	21.64 ± 19.95	40.57 ± 28.95
Jain	45.29 ± 13.71	41.53 ± 12.81	5.23 ± 16.55	0.68 ± 0.01	0.00 ± 0.00	0.00 ± 0.00	0.00 ± 0.00	39.92 ± 11.04	40.90 ± 10.57	44.01 ± 25.18
Jsbach-chorals-harmony	81.57 ± 0.79	50.79 ± 3.84	54.21 ± 4.56	45.20 ± 4.47	60.15 ± 4.88	54.68 ± 4.07	51.89 ± 6.48	45.78 ± 5.80	50.58 ± 8.14	62.55 ± 4.68
Knowledge	85.87 ± 4.18	59.22 ± 16.30	34.90 ± 22.51	81.75 ± 13.88	64.23 ± 21.32	47.97 ± 29.57	38.60 ± 29.57	39.56 ± 17.94	41.39 ± 16.90	67.95 ± 17.68
Lasvegastripadvisorreviews	100.32 ± 0.25	111.50 ± 3.13	121.51 ± 3.80	139.33 ± 3.30	142.23 ± 2.77	143.67 ± 3.93	141.71 ± 4.01	128.63 ± 4.12	129.31 ± 4.45	141.56 ± 2.71
Leaf	86.01 ± 1.17	68.95 ± 10.66	83.50 ± 8.86	135.31 ± 2.22	130.96 ± 3.21	125.23 ± 4.31	124.00 ± 4.63	85.62 ± 6.01	88.69 ± 5.82	125.49 ± 3.94
Led-display	88.07 ± 0.42	66.81 ± 3.97	70.14 ± 4.33	68.69 ± 4.89	78.85 ± 5.79	79.17 ± 5.30	79.60 ± 5.09	68.26 ± 4.39	68.70 ± 4.07	133.70 ± 1.05
Lenses	82.99 ± 20.68	103.60 ± 12.86	60.43 ± 78.78	51.16 ± 53.78	53.59 ± 72.68	55.38 ± 78.02	55.38 ± 78.02	75.56 ± 66.81	79.26 ± 70.36	93.86 ± 84.38
Letter	90.04 ± 0.12	72.31 ± 0.65	47.09 ± 1.30	29.17 ± 1.05	32.64 ± 0.80	28.83 ± 1.07	26.93 ± 0.84	56.10 ± 1.91	58.02 ± 1.50	131.19 ± 0.29
Libras	82.82 ± 2.25	85.98 ± 11.79	74.79 ± 12.39	53.22 ± 10.15	63.27 ± 6.84	55.56 ± 8.53	52.31 ± 8.10	58.94 ± 6.85	58.09 ± 6.86	129.89 ± 5.00
Low-res-spect	79.46 ± 1.62	76.21 ± 7.57	68.59 ± 7.60	69.06 ± 8.72	56.34 ± 6.62	55.72 ± 7.39	53.10 ± 7.30	43.58 ± 7.72	46.68 ± 8.00	88.79 ± 8.96
Lung-cancer	93.99 ± 4.88	96.56 ± 31.00	117.28 ± 29.32	103.21 ± 56.95	110.93 ± 42.92	103.54 ± 41.58	112.79 ± 45.74	114.07 ± 24.85	114.63 ± 25.64	124.19 ± 22.45
Lymphography	87.03 ± 2.34	70.67 ± 17.62	91.72 ± 19.36	88.84 ± 36.07	67.15 ± 29.90	72.22 ± 22.02	77.76 ± 18.30	75.23 ± 17.11	75.45 ± 19.22	94.63 ± 15.89
Magic	84.07 ± 1.78	101.85 ± 4.24	78.74 ± 4.82	93.84 ± 5.47	81.20 ± 5.88	80.57 ± 4.71	80.60 ± 3.86	71.56 ± 5.36	74.11 ± 5.40	110.22 ± 8.88
Mammographic	79.84 ± 2.61	82.69 ± 7.12	72.33 ± 4.82	94.63 ± 9.68	83.20 ± 5.26	84.13 ± 6.19	83.62 ± 6.41	73.77 ± 4.47	75.48 ± 6.23	85.30 ± 2.93
Miniboone	82.25 ± 1.18	189.93 ± 1.93	81.34 ± 7.18	91.38 ± 4.32	88.45 ± 7.42	85.36 ± 6.44	83.07 ± 5.95	75.70 ± 4.34	76.58 ± 4.28	96.24 ± 4.74
Molec-biol-promoter	89.39 ± 4.02	54.41 ± 28.47	96.63 ± 20.34	101.51 ± 23.11	79.01 ± 27.86	71.19 ± 34.52	71.19 ± 34.52	84.66 ± 19.60	85.23 ± 19.65	108.30 ± 20.19
Molec-biol-splice	96.61 ± 0.21	43.89 ± 4.68	47.20 ± 6.16	109.44 ± 3.15	64.55 ± 5.12	64.20 ± 4.41	65.59 ± 3.29	64.73 ± 4.94	65.33 ± 4.39	109.16 ± 3.42
Monks-1	79.68 ± 6.35	85.58 ± 16.00	18.01 ± 25.67	107.12 ± 19.53	77.61 ± 43.67	72.46 ± 41.02	76.16 ± 32.30	43.91 ± 43.45	36.89 ± 47.79	101.45 ± 20.94
Monks-2	100.90 ± 2.75	103.08 ± 4.57	90.54 ± 20.46	126.56 ± 16.70	133.06 ± 10.97	127.05 ± 21.06	121.08 ± 19.59	92.51 ± 20.24	90.88 ± 13.73	132.43 ± 7.25
Monks-3	78.01 ± 7.23	64.86 ± 11.40	40.50 ± 31.32	94.43 ± 30.54	51.83 ± 37.08	57.14 ± 33.62	57.14 ± 33.62	64.71 ± 32.51	55.86 ± 24.15	93.71 ± 8.47
Mushroom	67.75 ± 1.00	65.00 ± 3.45	0.00 ± 0.00	0.03 ± 0.00	0.00 ± 0.00	0.00 ± 0.00	0.00 ± 0.00	0.29 ± 0.04	0.22 ± 0.04	23.99 ± 4.20
Musk-1	82.69 ± 2.50	98.13 ± 14.21	75.59 ± 17.22	77.25 ± 12.78	59.52 ± 15.23	54.34 ± 11.78	49.30 ± 12.26	41.77 ± 10.93	42.38 ± 10.08	122.88 ± 14.88
Musk-2	79.29 ± 2.02	102.70 ± 5.93	56.26 ± 8.90	53.27 ± 6.39	53.67 ± 8.49	43.30 ± 8.56	37.44 ± 8.13	26.11 ± 9.61	26.31 ± 10.98	87.81 ± 5.55
Newdiagnosis	50.72 ± 3.08	30.73 ± 7.33	0.00 ± 0.00	0.18 ± 0.02	0.00 ± 0.00	0.00 ± 0.00	0.00 ± 0.00	1.51 ± 0.17	1.10 ± 0.10	94.98 ± 16.20
Nursery	89.23 ± 0.16	50.15 ± 1.52	10.57 ± 3.75	47.61 ± 1.53	22.67 ± 1.42	19.67 ± 1.54	17.60 ± 1.60	27.33 ± 4.63	31.85 ± 5.47	92.21 ± 1.63
Obesitydataset-raw-and-data-sinthetic	80.81 ± 0.44	72.54 ± 2.04	36.38 ± 5.59	64.50 ± 4.47	48.72 ± 5.55	43.66 ± 5.84	41.54 ± 6.13	33.04 ± 7.20	34.19 ± 4.07	87.58 ± 4.75
Obs-network-dataset-2-aug27	52.23 ± 2.24	86.99 ± 8.34	2.58 ± 8.15	14.70 ± 8.16	26.00 ± 4.09	26.00 ± 4.09	26.00 ± 4.09	26.01 ± 7.81	21.90 ± 6.73	52.27 ± 6.35
Occupancy-data	35.36 ± 3.13	41.97 ± 5.20	22.80 ± 6.37	18.52 ± 5.69	90.59 ± 4.83	87.73 ± 5.66	87.73 ± 5.66	26.40 ± 5.83	25.76 ± 5.83	22.91 ± 6.78
Occupancy-data2	35.32 ± 2.07	46.60 ± 3.32	17.76 ± 2.81	17.59 ± 3.65	99.54 ± 1.49	92.95 ± 1.88	92.95 ± 1.88	17.73 ± 3.34	17.88 ± 2.64	19.26 ± 2.59
Occupancy-data3	28.48 ± 3.36	33.40 ± 4.77	16.77 ± 1.68	17.60 ± 3.81	101.09 ± 1.58	96.21 ± 1.89	96.17 ± 1.87	21.45 ± 3.16	21.58 ± 2.94	19.14 ± 3.05
Old	85.14 ± 0.57	42.84 ± 3.78	39.55 ± 6.28	26.61 ± 5.34	44.47 ± 4.32	43.75 ± 5.86	42.45 ± 5.52	30.94 ± 5.80	30.90 ± 6.98	67.54 ± 4.81
Online-shoppers-intention	93.64 ± 0.25	107.81 ± 3.48	82.63 ± 2.45	118.80 ± 1.82	108.85 ± 0.19	109.02 ± 0.45	109.22 ± 0.54	78.72 ± 2.32	80.45 ± 4.54	94.78 ± 4.01
Oocytes-merluccius-nucleus-4d	92.39 ± 2.57	126.11 ± 8.34	93.80 ± 4.53	111.77 ± 7.42	101.17 ± 6.69	94.63 ± 5.96	92.41 ± 7.33	80.60 ± 6.86	83.56 ± 8.49	122.17 ± 9.10
Oocytes-merluccius-states-2f	63.53 ± 4.90	79.46 ± 9.81	61.47 ± 11.02	61.45 ± 9.52	59.47 ± 9.30	58.27 ± 7.35	55.66 ± 7.29	53.41 ± 5.97	54.99 ± 7.37	88.04 ± 11.26
Oocytes-trisopterus-nucleus-2f	94.00 ± 2.91	130.24 ± 8.25	98.36 ± 10.84	101.83 ± 11.84	87.53 ± 8.52	81.11 ± 8.90	82.39 ± 6.95	72.86 ± 8.59	78.50 ± 8.77	128.61 ± 9.79
Oocytes-trisopterus-states-5b	71.13 ± 6.12	95.75 ± 8.13	64.57 ± 8.79	58.95 ± 12.75	56.09 ± 12.28	54.47 ± 10.42	53.04 ± 9.19	46.27 ± 9.36	45.98 ± 10.04	89.06 ± 6.25
Optdigits	89.77 ± 0.15	42.09 ± 2.70	43.53 ± 3.37	17.13 ± 4.00	77.23 ± 2.78	74.66 ± 3.13	74.66 ± 3.13	18.30 ± 3.43	16.68 ± 3.29	127.34 ± 1.08
Optical	89.27 ± 0.22	41.47 ± 3.05	45.83 ± 2.96	18.20 ± 3.46	17.43 ± 3.32	16.17 ± 4.42	16.45 ± 3.97	17.67 ± 3.15	18.78 ± 3.43	126.75 ± 1.13
Ozone	99.38 ± 0.13	318.38 ± 16.15	119.39 ± 9.97	131.98 ± 8.63	101.46 ± 0.10	101.46 ± 0.10	101.46 ± 0.10	108.58 ± 14.16	103.74 ± 12.33	106.04 ± 6.86
Page-blocks	73.74 ± 2.77	95.47 ± 6.48	54.63 ± 3.52	64.31 ± 5.03	63.14 ± 6.33	60.92 ± 6.85	60.60 ± 6.90	56.36 ± 6.09	57.36 ± 4.93	81.05 ± 3.97
Parkingbirmingham	49.50 ± 2.79	56.99 ± 1.85	0.00 ± 0.00	22.73 ± 2.99	92.47 ± 4.19	89.49 ± 4.52	89.49 ± 4.52	46.52 ± 1.78	43.82 ± 2.02	0.00 ± 0.00
Parkinsons	80.16 ± 7.40	124.32 ± 25.45	98.43 ± 12.59	36.91 ± 24.59	78.74 ± 21.53	68.60 ± 30.39	62.99 ± 28.27	63.95 ± 25.07	60.75 ± 32.61	83.06 ± 25.05
Pasture	85.52 ± 8.61	70.69 ± 49.92	62.84 ± 47.61	70.45 ± 55.58	145.90 ± 3.85	145.90 ± 3.85	145.90 ± 3.85	63.21 ± 50.80	65.25 ± 51.66	91.28 ± 53.06
Pbc	87.75 ± 3.46	88.39 ± 13.99	84.26 ± 14.91	129.68 ± 13.55	127.52 ± 0.80	127.52 ± 0.80	127.52 ± 0.80	101.73 ± 9.69	98.13 ± 6.58	106.95 ± 18.06
Pen	72.38 ± 0.38	53.31 ± 2.25	26.98 ± 1.93	11.80 ± 1.59	138.66 ± 0.69	137.88 ± 0.77	137.88 ± 0.77	32.79 ± 1.90	33.46 ± 1.55	116.48 ± 1.48
Pendigits	71.98 ± 0.40	49.64 ± 2.71	28.47 ± 2.79	11.05 ± 2.40	8.97 ± 2.47	8.09 ± 3.11	8.23 ± 3.21	30.62 ± 1.93	31.40 ± 1.82	115.72 ± 1.90
Pharynx	101.25 ± 2.56	108.18 ± 9.07	100.00 ± 0.04	139.58 ± 22.58	115.08 ± 16.84	119.68 ± 22.09	126.61 ± 20.52	115.62 ± 9.70	119.82 ± 28.27	201.32 ± 11.50
Phishingwebsites	87.95 ± 0.21	46.34 ± 2.22	37.25 ± 2.06	28.94 ± 2.65	47.16 ± 2.25	44.97 ± 2.23	43.78 ± 2.11	32.02 ± 2.06	32.09 ± 1.80	67.07 ± 1.70
Pima	90.47 ± 3.59	87.25 ± 8.42	93.05 ± 10.72	114.11 ± 9.22	103.18 ± 8.64	104.30 ± 8.58	104.66 ± 7.23	88.21 ± 5.91	88.85 ± 7.35	111.61 ± 10.35
Pittsburg-bridges-rel-l	89.50 ± 5.08	89.37 ± 18.30	97.38 ± 18.60	89.14 ± 38.62	102.52 ± 20.85	101.05 ± 20.80	92.90 ± 21.20	99.94 ± 19.10	96.28 ± 20.16	96.57 ± 19.22
Pittsburg-bridges-span	89.67 ± 4.40	84.73 ± 15.52	94.81 ± 16.21	113.97 ± 26.05	104.74 ± 21.91	105.75 ± 26.40	105.75 ± 26.40	91.20 ± 12.11	91.85 ± 17.20	120.35 ± 16.75
Pittsburg-bridges-t-or-d	95.87 ± 7.30	88.10 ± 22.04	106.33 ± 31.57	106.73 ± 46.76	98.57 ± 35.87	88.59 ± 50.29	84.28 ± 46.41	83.77 ± 58.07	89.37 ± 63.44	98.71 ± 59.34
Pittsburg-bridges-type	92.48 ± 4.29	88.86 ± 10.41	91.56 ± 10.94	101.39 ± 15.88	109.49 ± 15.61	98.66 ± 23.87	98.12 ± 19.29	90.60 ± 19.32	95.59 ± 19.39	107.61 ± 7.39
Pittsburg-bridgesmaterial	80.30 ± 5.59	80.47 ± 16.73	68.43 ± 21.17	81.75 ± 38.26	80.98 ± 15.65	83.67 ± 14.88	82.05 ± 34.00	79.77 ± 19.53	86.96 ± 19.61	78.92 ± 11.95
Planning	100.69 ± 2.28	109.01 ± 8.59	100.00 ± 0.02	127.40 ± 17.80	118.23 ± 1.26	120.18 ± 11.79	128.81 ± 13.19	124.95 ± 13.76	133.30 ± 19.79	135.77 ± 15.55
Plant-margin	95.45 ± 0.12	53.64 ± 4.45	97.15 ± 4.59	70.29 ± 3.67	58.80 ± 4.03	55.28 ± 5.16	54.59 ± 5.18	50.54 ± 3.17	51.14 ± 3.91	136.54 ± 1.61
Plant-shape	93.38 ± 0.12	94.55 ± 3.05	98.45 ± 3.28	81.84 ± 3.95	101.54 ± 1.61	93.41 ± 3.39	89.74 ± 3.79	73.32 ± 4.17	74.65 ± 3.18	135.89 ± 1.20
Plant-texture	95.78 ± 0.15	70.64 ± 4.59	93.55 ± 3.26	61.49 ± 4.34	57.93 ± 4.25	53.86 ± 4.30	52.90 ± 4.50	51.11 ± 2.22	52.36 ± 2.09	139.65 ± 0.65
Poker-hand-training-true	100.00 ± 0.01	100.01 ± 0.04	112.92 ± 2.44	136.31 ± 1.12	120.55 ± 1.45	122.21 ± 1.34	123.81 ± 1.58	99.37 ± 0.59	100.33 ± 1.10	132.74 ± 0.01
Post-operative	100.05 ± 3.10	107.98 ± 7.49	99.93 ± 0.31	135.74 ± 14.49	116.16 ± 4.71	120.44 ± 8.71	124.20 ± 12.12	120.66 ± 17.21	128.98 ± 20.86	120.44 ± 8.71
Primary-tumor	95.96 ± 0.67	92.08 ± 8.27	99.11 ± 5.53	114.86 ± 7.80	109.30 ± 11.30	111.22 ± 10.70	112.01 ± 12.02	102.94 ± 7.17	104.08 ± 7.15	127.72 ± 2.50
Qsarbioconcentration	100.25 ± 0.61	102.46 ± 2.87	103.67 ± 8.00	142.45 ± 10.81	115.78 ± 0.99	119.26 ± 3.67	125.03 ± 6.00	108.72 ± 6.30	110.13 ± 5.28	120.21 ± 6.76
Qsarbiodegradation	89.51 ± 1.69	100.09 ± 6.60	80.51 ± 7.36	85.38 ± 5.91	79.18 ± 7.31	78.18 ± 6.39	79.99 ± 7.23	69.56 ± 5.11	70.77 ± 6.78	101.76 ± 8.68
Qualitative-bankruptcy	32.35 ± 3.73	5.51 ± 10.58	17.29 ± 22.81	1.33 ± 2.38	9.79 ± 21.04	9.79 ± 21.04	4.03 ± 12.74	4.33 ± 11.38	4.13 ± 11.31	13.86 ± 22.80
Ringnorm	74.09 ± 2.09	19.74 ± 2.98	56.68 ± 0.70	99.62 ± 3.20	23.46 ± 3.65	24.51 ± 3.38	25.43 ± 3.77	53.40 ± 4.65	53.76 ± 2.92	119.27 ± 1.63
Risk-factors-cervical-cancer	94.94 ± 1.15	126.72 ± 13.05	77.04 ± 17.52	95.64 ± 19.78	103.33 ± 0.35	104.24 ± 5.08	99.45 ± 11.46	80.68 ± 15.46	87.01 ± 18.10	77.93 ± 21.08
Robotnavigation	55.25 ± 1.48	107.44 ± 1.88	10.13 ± 3.43	59.46 ± 3.13	54.79 ± 3.15	51.52 ± 2.86	49.12 ± 2.39	55.42 ± 4.28	55.26 ± 6.04	86.18 ± 3.25
Sapfile	96.14 ± 1.48	100.24 ± 9.39	115.93 ± 15.89	129.08 ± 8.60	125.60 ± 11.63	124.81 ± 8.63	124.58 ± 10.95	116.65 ± 10.66	117.63 ± 13.94	133.77 ± 14.89
Sat	60.61 ± 0.60	69.58 ± 2.17	56.20 ± 2.03	48.23 ± 2.01	135.02 ± 0.49	133.93 ± 0.43	133.93 ± 0.43	46.77 ± 3.44	49.11 ± 2.28	99.66 ± 1.62
Satelite	60.65 ± 0.61	69.61 ± 2.16	55.92 ± 1.59	48.11 ± 2.83	135.03 ± 0.52	133.86 ± 0.65	133.86 ± 0.65	46.50 ± 1.04	48.25 ± 2.28	99.38 ± 2.04
Scadi	87.09 ± 1.43	61.49 ± 23.59	62.84 ± 26.02	66.13 ± 26.97	79.45 ± 15.60	70.12 ± 31.33	61.52 ± 37.62	67.24 ± 19.91	67.29 ± 20.46	104.98 ± 13.00
Schillingdata	84.94 ± 1.12	62.10 ± 2.63	100.00 ± 0.00	91.64 ± 3.69	107.37 ± 0.09	97.69 ± 4.54	85.26 ± 3.41	71.97 ± 9.52	85.21 ± 12.03	107.98 ± 1.36
Seeds	58.92 ± 5.93	44.58 ± 18.36	45.28 ± 20.77	40.64 ± 6.55	38.07 ± 21.30	38.07 ± 21.30	38.07 ± 21.30	30.45 ± 10.31	29.24 ± 9.57	70.34 ± 19.38
Segment	59.50 ± 1.21	65.40 ± 2.80	25.83 ± 4.34	25.64 ± 2.84	89.82 ± 3.71	87.68 ± 3.17	87.74 ± 3.25	27.25 ± 3.48	25.77 ± 2.60	91.78 ± 3.52
Seismic-bumps	99.02 ± 0.35	138.24 ± 9.17	100.18 ± 0.57	131.00 ± 9.20	103.46 ± 0.01	103.46 ± 0.01	103.46 ± 0.01	100.79 ± 3.96	102.92 ± 4.94	106.98 ± 4.05
Semeion	96.86 ± 0.07	53.86 ± 5.51	70.01 ± 3.45	42.40 ± 4.21	31.05 ± 3.77	29.08 ± 2.48	29.31 ± 2.58	35.85 ± 4.06	35.19 ± 4.68	133.68 ± 0.39
Setapprocesst1	95.41 ± 5.81	130.37 ± 39.71	120.89 ± 28.33	123.25 ± 19.31	128.77 ± 13.55	118.98 ± 21.01	122.56 ± 17.65	116.55 ± 45.33	116.16 ± 44.96	98.22 ± 61.05
Setapprocesst10	99.97 ± 4.17	154.43 ± 21.86	131.27 ± 23.89	127.99 ± 32.23	122.05 ± 5.22	122.05 ± 5.22	122.05 ± 5.22	133.00 ± 42.66	133.55 ± 36.13	129.59 ± 14.46
Setapprocesst11	99.41 ± 4.33	134.13 ± 42.31	116.67 ± 34.31	132.74 ± 23.21	122.05 ± 5.22	122.05 ± 5.22	122.05 ± 5.22	121.08 ± 25.91	114.69 ± 28.33	137.85 ± 16.93
Setapprocesst2	98.71 ± 4.31	114.63 ± 26.50	90.85 ± 49.65	117.85 ± 48.89	122.05 ± 5.22	122.05 ± 5.22	122.05 ± 5.22	118.79 ± 31.46	113.52 ± 32.85	77.95 ± 49.39
Setapprocesst3	99.05 ± 4.05	122.25 ± 23.50	126.61 ± 30.35	119.20 ± 27.49	122.05 ± 5.22	122.05 ± 5.22	122.05 ± 5.22	117.27 ± 24.34	118.37 ± 16.90	143.18 ± 26.50
Setapprocesst4	100.78 ± 1.89	146.77 ± 47.89	141.95 ± 29.31	133.29 ± 34.37	116.99 ± 7.71	116.99 ± 7.71	116.99 ± 7.71	136.51 ± 38.59	136.70 ± 30.51	113.48 ± 23.96
Setapprocesst5	97.92 ± 5.06	150.96 ± 26.50	134.24 ± 31.44	129.29 ± 27.69	124.00 ± 9.11	125.95 ± 11.41	125.95 ± 11.41	121.69 ± 19.30	120.29 ± 18.53	113.68 ± 55.27
Setapprocesst6	98.36 ± 2.96	114.55 ± 30.62	116.30 ± 25.27	122.44 ± 27.05	122.05 ± 5.22	122.05 ± 5.22	122.05 ± 5.22	117.72 ± 37.58	119.04 ± 33.16	130.04 ± 35.03
Setapprocesst7	98.65 ± 4.55	98.83 ± 35.72	118.41 ± 49.74	121.67 ± 25.15	122.05 ± 5.22	122.05 ± 5.22	122.05 ± 5.22	97.17 ± 40.62	98.87 ± 40.41	143.69 ± 23.95
Setapprocesst8	97.57 ± 3.64	113.36 ± 28.07	103.27 ± 30.86	120.07 ± 43.69	122.05 ± 5.22	122.05 ± 5.22	122.05 ± 5.22	109.43 ± 40.65	107.74 ± 43.23	150.24 ± 22.05
Setapprocesst9	98.89 ± 3.20	117.62 ± 26.75	110.54 ± 31.35	133.62 ± 14.38	122.05 ± 5.22	122.05 ± 5.22	122.05 ± 5.22	97.88 ± 43.95	108.83 ± 43.44	147.47 ± 28.50
Shillbiddingdataset	68.39 ± 0.58	55.61 ± 4.28	19.11 ± 3.70	28.07 ± 5.14	21.45 ± 4.75	20.85 ± 5.67	18.40 ± 5.37	15.59 ± 4.50	15.05 ± 7.07	53.18 ± 5.20
Shuttle-landing-control	105.16 ± 98.60	166.55 ± 227.78	62.34 ± 15.65	84.84 ± 117.72	10.69 ± 33.80	10.69 ± 33.80	10.69 ± 33.80	102.61 ± 199.52	106.67 ± 212.35	90.69 ± 251.48
Somervillehappinesssurvey2015	97.70 ± 6.04	103.40 ± 12.60	96.69 ± 20.43	111.71 ± 21.10	125.77 ± 16.48	128.01 ± 15.36	127.58 ± 18.64	117.91 ± 19.08	118.43 ± 20.09	114.71 ± 26.82
Sonar	91.62 ± 2.77	105.49 ± 14.10	103.66 ± 12.82	70.39 ± 20.51	116.68 ± 9.43	108.18 ± 16.25	101.43 ± 18.65	74.27 ± 29.19	74.33 ± 29.17	121.44 ± 18.90
Soybean	89.33 ± 0.66	36.08 ± 16.54	47.74 ± 8.71	49.93 ± 10.10	44.37 ± 13.94	39.92 ± 11.59	41.48 ± 12.26	39.47 ± 11.30	39.60 ± 9.78	123.24 ± 2.12
Spambase	85.27 ± 0.75	93.02 ± 3.90	52.34 ± 4.65	62.03 ± 3.75	52.49 ± 3.69	52.01 ± 4.34	52.40 ± 3.66	54.04 ± 3.55	59.05 ± 14.74	95.13 ± 4.38
Speaker-accent	91.78 ± 1.83	94.38 ± 11.13	88.13 ± 14.93	72.21 ± 16.93	109.16 ± 4.98	103.82 ± 5.74	103.82 ± 5.74	67.13 ± 9.34	72.38 ± 10.63	119.12 ± 5.82
Spect	95.44 ± 7.39	102.14 ± 40.33	103.21 ± 21.86	133.24 ± 23.52	124.73 ± 22.08	125.35 ± 24.79	128.37 ± 18.79	128.96 ± 32.08	128.60 ± 34.38	110.24 ± 19.55
Spectf	94.23 ± 3.59	91.65 ± 27.14	98.75 ± 23.79	108.64 ± 30.80	113.48 ± 11.61	113.48 ± 11.61	90.20 ± 38.92	96.18 ± 37.11	96.91 ± 27.49	106.06 ± 16.70
Statlog-australian-credit	98.96 ± 0.48	107.03 ± 5.64	108.70 ± 11.95	142.02 ± 5.56	121.92 ± 3.24	127.38 ± 11.14	128.23 ± 10.04	113.34 ± 6.96	115.26 ± 7.19	125.81 ± 11.36
Statlog-german-credit	97.75 ± 0.31	91.18 ± 9.26	101.98 ± 10.77	123.48 ± 7.63	106.29 ± 9.64	103.65 ± 8.60	103.51 ± 10.34	111.43 ± 6.66	108.76 ± 7.25	117.63 ± 4.61
Statlog-heart	82.60 ± 3.30	72.29 ± 14.04	91.43 ± 18.07	97.58 ± 22.29	82.16 ± 12.91	88.01 ± 19.85	88.40 ± 22.00	85.72 ± 11.79	83.24 ± 17.25	107.56 ± 12.07
Statlog-image	57.69 ± 1.16	65.61 ± 2.80	25.80 ± 4.36	25.64 ± 2.84	36.41 ± 2.85	33.47 ± 3.49	32.41 ± 3.37	26.62 ± 3.57	27.11 ± 4.36	92.01 ± 3.11
Statlog-landsat	60.00 ± 1.20	69.60 ± 3.86	56.29 ± 3.34	49.16 ± 4.47	50.91 ± 3.91	49.15 ± 3.50	48.94 ± 4.15	47.79 ± 2.41	48.96 ± 4.41	98.91 ± 2.10
Statlog-shuttle	75.97 ± 0.52	55.93 ± 2.89	3.96 ± 2.30	5.32 ± 2.37	11.79 ± 1.08	10.62 ± 1.27	10.45 ± 1.18	12.91 ± 0.97	12.44 ± 1.44	54.45 ± 1.53
Statlog-vehicle	82.35 ± 1.47	105.79 ± 3.29	77.33 ± 7.49	89.18 ± 7.10	78.56 ± 6.68	72.42 ± 4.86	72.01 ± 4.41	60.55 ± 4.46	63.31 ± 6.52	113.97 ± 6.04
Steel-plates	81.03 ± 1.11	95.55 ± 4.13	73.83 ± 4.89	84.63 ± 4.10	79.20 ± 4.55	76.86 ± 3.76	76.98 ± 4.79	75.69 ± 4.31	76.97 ± 4.56	115.36 ± 4.37
Synthetic-control	71.91 ± 1.54	33.55 ± 10.45	43.08 ± 8.09	26.76 ± 11.35	8.00 ± 10.33	6.00 ± 9.66	6.00 ± 9.66	10.17 ± 6.45	9.86 ± 6.70	101.50 ± 4.44
Teaching	94.76 ± 3.91	96.13 ± 6.24	98.63 ± 12.16	101.23 ± 17.58	113.25 ± 18.91	106.11 ± 17.95	105.23 ± 17.49	94.76 ± 8.62	97.42 ± 6.28	126.23 ± 13.02
Thoraricsurgery	99.45 ± 0.57	109.78 ± 18.45	102.63 ± 4.26	132.67 ± 17.50	108.40 ± 0.00	110.64 ± 3.62	110.64 ± 3.62	116.89 ± 12.79	114.78 ± 17.81	114.29 ± 5.75
Thyroid	68.47 ± 12.02	26.24 ± 23.77	48.57 ± 29.18	27.35 ± 21.99	101.08 ± 10.29	98.28 ± 9.98	98.28 ± 9.98	29.59 ± 19.61	29.79 ± 20.14	53.58 ± 35.33
Thyroid-train	93.14 ± 1.53	70.78 ± 7.61	14.42 ± 13.66	105.06 ± 8.86	83.03 ± 6.05	77.92 ± 5.45	72.39 ± 6.23	61.02 ± 11.32	67.36 ± 7.95	70.09 ± 8.43
Tic-tac-toe	96.73 ± 0.39	90.24 ± 1.41	47.77 ± 9.33	0.19 ± 0.01	19.64 ± 15.44	12.30 ± 13.80	5.20 ± 11.16	28.86 ± 7.00	26.86 ± 10.01	115.14 ± 4.59
Titanic	88.41 ± 1.91	89.75 ± 4.30	84.88 ± 3.28	84.21 ± 2.86	99.13 ± 5.01	98.09 ± 4.62	98.09 ± 4.62	86.21 ± 3.78	86.83 ± 3.79	101.12 ± 4.52
Trains	84.82 ± 15.40	73.34 ± 94.66	51.94 ± 84.05	76.67 ± 77.46	73.33 ± 94.67	55.00 ± 88.56	55.00 ± 88.56	50.95 ± 67.29	49.70 ± 68.53	183.33 ± 0.00
Transfusion	97.89 ± 1.28	101.29 ± 5.76	93.54 ± 3.96	123.86 ± 10.03	116.61 ± 6.74	121.88 ± 6.71	124.21 ± 8.00	92.25 ± 2.87	92.58 ± 3.13	114.18 ± 4.07
Trial	71.22 ± 2.99	61.56 ± 12.59	0.00 ± 0.00	3.58 ± 10.41	15.05 ± 13.28	11.72 ± 12.36	11.72 ± 12.36	12.98 ± 13.25	10.62 ± 13.25	0.00 ± 0.00
Turkiye-student-evaluation	94.38 ± 0.27	65.14 ± 3.64	1.60 ± 3.37	59.50 ± 4.97	40.85 ± 2.39	37.12 ± 3.05	37.12 ± 3.05	0.40 ± 0.05	0.29 ± 0.04	1.60 ± 3.37
Unbalanced	99.74 ± 0.37	235.69 ± 70.87	99.98 ± 0.04	124.01 ± 60.40	100.62 ± 0.20	108.95 ± 17.51	115.38 ± 19.81	111.48 ± 11.91	113.44 ± 19.54	100.62 ± 0.20
Urbanlandcover	82.34 ± 3.57	66.22 ± 17.19	65.92 ± 14.08	68.91 ± 17.55	136.97 ± 1.66	136.97 ± 1.66	136.97 ± 1.66	60.94 ± 18.91	61.47 ± 19.17	106.26 ± 15.89
Userknowledgemodeling	84.76 ± 4.06	54.15 ± 11.90	36.41 ± 14.46	70.68 ± 20.81	78.40 ± 11.21	63.64 ± 9.44	62.85 ± 8.75	39.38 ± 12.61	36.40 ± 17.04	61.92 ± 18.43
Vehicle	81.91 ± 1.54	106.66 ± 3.74	77.14 ± 7.64	89.18 ± 7.10	136.15 ± 2.49	135.32 ± 3.04	135.32 ± 3.04	*60.31 ± 5.77*	62.30 ± 8.02	113.14 ± 6.02
Vertebral-column-2classes	85.52 ± 8.10	90.62 ± 18.71	78.76 ± 15.04	89.82 ± 12.51	79.79 ± 17.55	82.11 ± 19.52	81.50 ± 18.11	*68.63 ± 12.67*	68.66 ± 13.18	108.06 ± 17.67
Vertebral-column-3classes	78.56 ± 3.93	62.09 ± 8.69	68.73 ± 9.58	81.65 ± 9.57	70.69 ± 13.71	68.08 ± 15.71	69.92 ± 13.91	*57.42 ± 7.72*	57.94 ± 7.70	88.43 ± 12.33
Veteran	*95.55 ± 1.99*	102.19 ± 14.10	99.16 ± 13.68	136.01 ± 16.45	118.84 ± 0.89	118.84 ± 0.89	118.84 ± 0.89	119.03 ± 25.78	112.21 ± 25.61	111.85 ± 15.81
Vowel	84.58 ± 1.12	72.78 ± 4.98	59.07 ± 7.83	*9.27 ± 8.77*	57.14 ± 5.18	43.98 ± 4.85	39.05 ± 5.81	36.65 ± 7.58	33.50 ± 6.25	122.24 ± 3.70
Wall-following	55.39 ± 1.48	107.47 ± 1.49	*9.26 ± 4.25*	59.65 ± 3.10	57.51 ± 3.68	53.11 ± 4.42	50.98 ± 3.82	57.18 ± 3.46	56.64 ± 4.85	85.73 ± 2.46
Waveform-noise	86.71 ± 1.01	71.40 ± 3.04	83.64 ± 2.55	88.91 ± 2.16	*64.39 ± 3.61*	66.17 ± 2.97	67.24 ± 3.23	66.72 ± 3.80	68.45 ± 3.21	117.79 ± 3.50
Waveform	82.03 ± 0.70	69.94 ± 2.73	81.03 ± 2.77	83.15 ± 3.65	*63.76 ± 3.21*	65.52 ± 3.16	65.98 ± 3.25	64.30 ± 3.07	67.02 ± 2.96	119.31 ± 2.07
Wbc	37.11 ± 6.99	29.48 ± 13.02	49.19 ± 5.07	38.62 ± 12.77	28.23 ± 16.15	28.23 ± 16.15	*25.70 ± 18.48*	39.65 ± 7.43	37.50 ± 9.78	62.02 ± 9.71
Wdbc	58.62 ± 4.93	49.24 ± 21.86	48.65 ± 21.03	40.40 ± 10.17	126.24 ± 0.73	126.24 ± 0.73	126.24 ± 0.73	*33.32 ± 11.34*	35.81 ± 14.00	65.33 ± 10.09
Weathernominal	104.96 ± 33.71	91.92 ± 35.51	89.15 ± 79.96	112.45 ± 59.89	69.39 ± 73.26	94.39 ± 88.11	65.79 ± 91.09	*65.58 ± 89.13*	65.63 ± 89.79	125.02 ± 96.40
Weathernumeric	120.03 ± 26.66	99.85 ± 31.34	*56.97 ± 76.30*	59.08 ± 76.89	69.39 ± 73.26	105.66 ± 96.79	105.66 ± 96.79	60.65 ± 85.06	61.50 ± 85.55	125.02 ± 96.40
Website-phishingdata	79.99 ± 1.06	64.11 ± 4.29	*50.91 ± 5.65*	57.76 ± 5.77	71.44 ± 3.16	70.14 ± 3.00	66.14 ± 5.56	58.22 ± 5.13	57.40 ± 6.15	80.60 ± 3.65
Wholesalecustomersdata	63.07 ± 4.98	63.19 ± 10.85	61.88 ± 9.32	72.69 ± 15.03	121.49 ± 0.83	121.49 ± 0.83	121.49 ± 0.83	*54.15 ± 9.90*	54.77 ± 10.03	64.87 ± 13.39
Wifi-localization	59.41 ± 1.29	*19.68 ± 4.03*	26.37 ± 2.75	20.62 ± 5.50	74.62 ± 4.57	72.06 ± 4.85	72.06 ± 4.85	21.39 ± 2.98	21.94 ± 3.18	74.44 ± 8.05
Wilt	98.84 ± 1.18	121.71 ± 6.00	55.88 ± 7.46	100.06 ± 5.77	101.22 ± 1.27	101.22 ± 1.27	101.22 ± 1.27	54.61 ± 11.19	*51.12 ± 9.33*	104.16 ± 1.59
Wine-quality-red	*89.86 ± 1.73*	97.64 ± 3.22	101.93 ± 3.86	104.40 ± 4.03	107.55 ± 3.49	107.00 ± 3.74	106.82 ± 3.78	92.25 ± 3.08	92.41 ± 3.62	118.72 ± 3.95
Wine-quality-white	*92.27 ± 0.45*	103.72 ± 1.36	102.88 ± 3.21	101.09 ± 3.72	112.80 ± 3.01	111.66 ± 3.27	111.33 ± 3.51	93.01 ± 1.49	93.72 ± 1.18	126.72 ± 2.49
Wine	58.31 ± 4.34	19.42 ± 21.36	37.09 ± 22.77	32.53 ± 22.18	*8.20 ± 17.29*	12.45 ± 20.05	12.45 ± 20.05	19.20 ± 17.00	19.17 ± 17.76	82.06 ± 17.14
Yamilnaduelectricty	*97.44 ± 0.59*	100.01 ± 0.01	115.13 ± 0.78	128.98 ± 1.07	140.56 ± 0.41	140.44 ± 0.42	140.53 ± 0.46	100.05 ± 0.02	100.08 ± 0.02	139.95 ± 0.85
Yeast	93.16 ± 0.64	85.84 ± 2.25	96.02 ± 4.56	110.39 ± 2.76	101.06 ± 5.16	100.97 ± 5.39	100.78 ± 5.69	*85.35 ± 2.58*	86.19 ± 2.76	124.22 ± 3.50
Youtobe-kabita-preprocessing	94.81 ± 0.39	106.30 ± 0.92	101.09 ± 1.73	114.12 ± 1.29	120.91 ± 2.29	121.97 ± 2.09	122.63 ± 2.01	*94.26 ± 0.77*	94.43 ± 0.79	125.61 ± 1.86
Youtobe-nisha-preprocessing	94.07 ± 0.26	102.51 ± 1.20	99.08 ± 1.55	112.16 ± 1.84	117.86 ± 2.11	119.07 ± 2.06	119.47 ± 2.18	*93.15 ± 0.91*	93.46 ± 0.93	126.67 ± 1.21
Z-alizadehsani	96.14 ± 1.07	85.75 ± 15.52	95.62 ± 20.20	101.44 ± 15.72	118.38 ± 1.28	118.38 ± 1.28	118.38 ± 1.28	*82.76 ± 19.42*	85.13 ± 15.70	119.29 ± 10.99
Zoo	69.25 ± 4.04	*17.28 ± 26.77*	29.70 ± 31.08	18.10 ± 20.91	24.24 ± 31.96	24.24 ± 31.96	24.24 ± 31.96	21.01 ± 21.73	20.30 ± 22.40	82.11 ± 16.46
Average rrse (rank)	83.25 (5)	85.51 (6)	70.59 (2)	80.47 (4)	90.03 (9)	88.26 (8)	87.19 (7)	69.93 (1)	70.66 (3)	98.86 (10)
Average rrse std (rank)	3.40 (1)	12.11 (5)	12.26 (6)	13.81 (9)	9.34 (2)	10.67 (3)	11.056 (4)	13.39 (8)	13.99 (10)	12.36 (7)

**Table 12:** Comparison of accuracy.

	RN	NB	J48	KNN	SVM1	SVM2	SVM3	ANN1	ANN2
NB	1.0								
J48	0.14	*0.01*							
KNN	0.7	0.17	1.0						
SVM1	1.0	1.0	*0.01*	0.15					
SVM2	1.0	1.0	*0.03*	0.35	1.0				
SVM3	1.0	1.0	0.06	0.48	1.0	1.0			
ANN1	0.29	*0.03*	1.0	1.0	*0.03*	0.09	0.14		
ANN2	0.32	*0.04*	1.0	1.0	*0.03*	0.1	0.17	1.0	
OneR	*0.01*	0.15	*0.0*	*0.0*	0.17	0.06	*0.03*	*0.0*	*0.0*

**Table 13:** Comparison of kappa statistic.

	RN	NB	J48	KNN	SVM1	SVM2	SVM3	ANN1	ANN2
NB	0.11								
J48	*0.0*	0.06							
KNN	*0.0*	0.59	0.99						
SVM1	1.0	0.12	*0.0*	*0.0*					
SVM2	1.0	0.56	*0.0*	*0.0*	1.0				
SVM3	0.97	0.82	*0.0*	*0.01*	0.97	1.0			
ANN1	*0.0*	0.06	1.0	0.98	*0.0*	*0.0*	*0.0*		
ANN2	*0.0*	0.07	1.0	0.99	*0.0*	*0.0*	*0.0*	1.0	
OneR	1.0	*0.02*	*0.0*	*0.0*	1.0	0.93	0.74	*0.0*	*0.0*

**Table 14:** Comparison of root mean squared error.

	RN	NB	J48	KNN	SVM1	SVM2	SVM3	ANN1	ANN2
NB	1.0								
J48	*0.03*	*0.01*							
KNN	1.0	1.0	0.13						
SVM1	0.54	0.79	*0.0*	0.22					
SVM2	0.83	0.96	*0.0*	0.51	1.0				
SVM3	0.95	0.99	*0.0*	0.71	1.0	1.0			
ANN1	*0.02*	*0.01*	1.0	0.1	*0.0*	*0.0*	*0.0*		
ANN2	*0.04*	*0.01*	1.0	0.16	*0.0*	*0.0*	*0.0*	1.0	
OneR	*0.0*	*0.01*	*0.0*	*0.0*	0.59	0.28	0.14	*0.0*	*0.0*

**Table 15:** Comparison of mean absolute error.

	RN	NB	J48	KNN	SVM1	SVM2	SVM3	ANN1	ANN2
NB	*0.0*								
J48	*0.0*	*0.04*							
KNN	*0.0*	0.1	1.0						
SVM1	*0.0*	0.65	0.94	0.99					
SVM2	*0.0*	0.45	0.99	1.0	1.0				
SVM3	*0.0*	0.3	1.0	1.0	1.0	1.0			
ANN1	*0.0*	*0.04*	1.0	1.0	0.95	0.99	1.0		
ANN2	*0.0*	*0.03*	1.0	1.0	0.91	0.98	1.0	1.0	
OneR	*0.0*	1.0	0.09	0.2	0.84	0.67	0.5	0.1	0.07

**Table 16:** Comparison of relative absolute error.

	RN	NB	J48	KNN	SVM1	SVM2	SVM3	ANN1	ANN2
NB	*0.0*								
J48	*0.0*	*0.0*							
KNN	*0.0*	*0.0*	1.0						
SVM1	*0.0*	*0.0*	0.84	0.94					
SVM2	*0.0*	*0.0*	0.97	0.99	1.0				
SVM3	*0.0*	*0.0*	0.99	1.0	1.0	1.0			
ANN1	*0.0*	*0.0*	1.0	1.0	0.71	0.91	0.97		
ANN2	*0.0*	*0.0*	1.0	1.0	0.63	0.86	0.94	1.0	
OneR	*0.0*	0.93	*0.0*	*0.0*	0.27	0.11	0.06	*0.0*	*0.0*

**Table 17:** Comparison of root relative squared error.

	RN	NB	J48	KNN	SVM1	SVM2	SVM3	ANN1	ANN2
NB	1.0								
J48	*0.0*	*0.0*							
KNN	1.0	0.84	0.05						
SVM1	0.48	0.92	*0.0*	0.07					
SVM2	0.85	1.0	*0.0*	0.28	1.0				
SVM3	0.96	1.0	*0.0*	0.5	1.0	1.0			
ANN1	*0.0*	*0.0*	1.0	*0.03*	*0.0*	*0.0*	*0.0*		
ANN2	*0.0*	*0.0*	1.0	0.06	*0.0*	*0.0*	*0.0*	1.0	
OneR	*0.0*	*0.0*	*0.0*	*0.0*	0.13	0.03	*0.01*	*0.0*	*0.0*

## Data Availability

The data used to support the findings of this study are included within the article.
